# Photovermellogens: Minimalistic Pyridinium-Based Acylhydrazones with Photoswitchable Basicity for Operation in Aqueous Media

**DOI:** 10.1021/acs.joc.5c02962

**Published:** 2026-05-04

**Authors:** Alejandro Vila, Francisco G. Blandón-Cumbreras, Mike Pauls, Mauro Díaz-Abellás, Arturo Blanco-Gómez, Carlos Peinador, Patricia Remón, Christoph Bannwarth, Uwe Pischel, Marcos D. García

**Affiliations:** † CICA − Centro Interdisciplinar de Química e Bioloxía and Departamento de Química, Facultade de Ciencias, 16737 Universidade da Coruña, A Coruña 15071, Spain; ‡ CIQSO − Center for Research in Sustainable Chemistry and Department of Chemistry, 16743 University of Huelva, Huelva 21071, Spain; § Institute of Physical Chemistry, RWTH Aachen University, Aachen 52074, Germany

## Abstract

Examples of a new class of synthetically straightforward and hydrolytically inert photoswitchable acylhydrazones, named photovermellogens, have been successfully developed and comprehensively studied using state-of-the-art spectroscopic and computational techniques. These compounds present photoswitchable basicity, with irradiation promoting *E*→*Z* photoisomerizations, which result in the generation of a less acidic species. The observed Δp*K*_a_ values > 1.5 within the biologically relevant window underscore the potential of photovermellogens as versatile platforms for light-triggered pH control.

## Graphical abstract

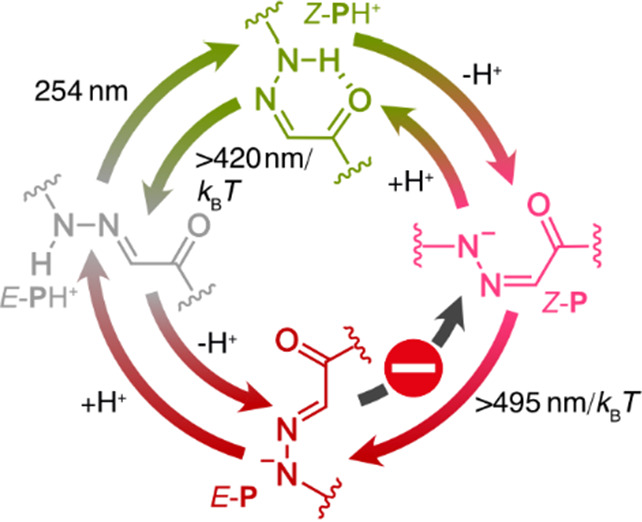

## 1 Introduction

Molecular photoswitches (MPs)^1^ are key tools in modern chemistry,^2^ with light stimulation enabling the precise spatiotemporal and remote noninvasive manipulation of matter.^3^ However, analogues capable of efficiently operating in biological milieus remain underdeveloped.^4^ Considering the different strategies for enhancing aqueous solubility, the introduction of ionizable groups is of particular interest, as such systems can also undergo a p*K*_a_ shift upon photoisomerization, showing photoswitchable acidity/basicity,^5^ opening the door for applications that rely on light-induced pH modulations.^6^

Among the popular families of MPs,^1–6^ (acyl)­hydrazones hold particular promise as extensively proven by Aprahamian et al.^7^ First, their appropriate functionalization leads to controlled photoisomerization of the C=N bond, and/or fine-tuning of the *E*/*Z* populations. Furthermore, the ability of these species to establish multifaceted noncovalent interactions, allows for further adjustment of the responsiveness by hydrogen bonding (HB) or anion/cation coordination.^8^ Finally, the photochemical properties have been thoroughly investigated, demonstrating favorable photostationary distributions (PSDs), high fatigue resistance, significant quantum yields and modular thermal half-lives. Despite these desirable features, the often limited and pH-dependent hydrolytic stability of imine derivatives^9^ poses a significant drawback and thus, only a few examples of (acyl)­hydrazone photoswitching in aqueous environments have been reported.^10^

In this context, some of us have reported the development of vermellogens (Scheme 1),^11^ pH-responsive analogues of the well-known viologens,^12^ that feature the insertion of a hydrazone function between two pyridinium rings. Previously, we found that the positively charged heterocycles not only enable water solubility, but also act as electron density sinks for the hydrazone group, increasing the hydrolytic stability of the C=N bond,^9^ and producing unusually low p*K*_a_ values for the NH motif in the range of 6–12.^11,13,14^ Herein we present the evolution of the functionality of vermellogens into the proposed dual pH-/light-responsiveness of photovermellogens **P**_a–d_H^+^.^15^ As shown in Scheme 1, the high acidity of the hydrazone in vermellogens is proposed to be potentially preserved by using a 4-hydrazinylpyridinium-like scaffold in the readily accessible analogues **H**_a–d_^+^ that were used as synthetic precursors.^16^ In turn, condensation of these intermediates with phenyl glyoxal in acidic aqueous medium introduces a carbonyl group in α-position to the C=N bond, aimed to stabilize the otherwise minor *Z* isomers by HB and thereby promote *E* to *Z* photoswitching.^7^ Hence, our designed compounds can potentially exist in four different states *E*/*Z*-[**P**_a–d_/**P**_a–d_H^+^], depending on the degree of protonation of the NH group and the *E*/*Z* isomerization of the C=N bond.

**Scheme 1:**
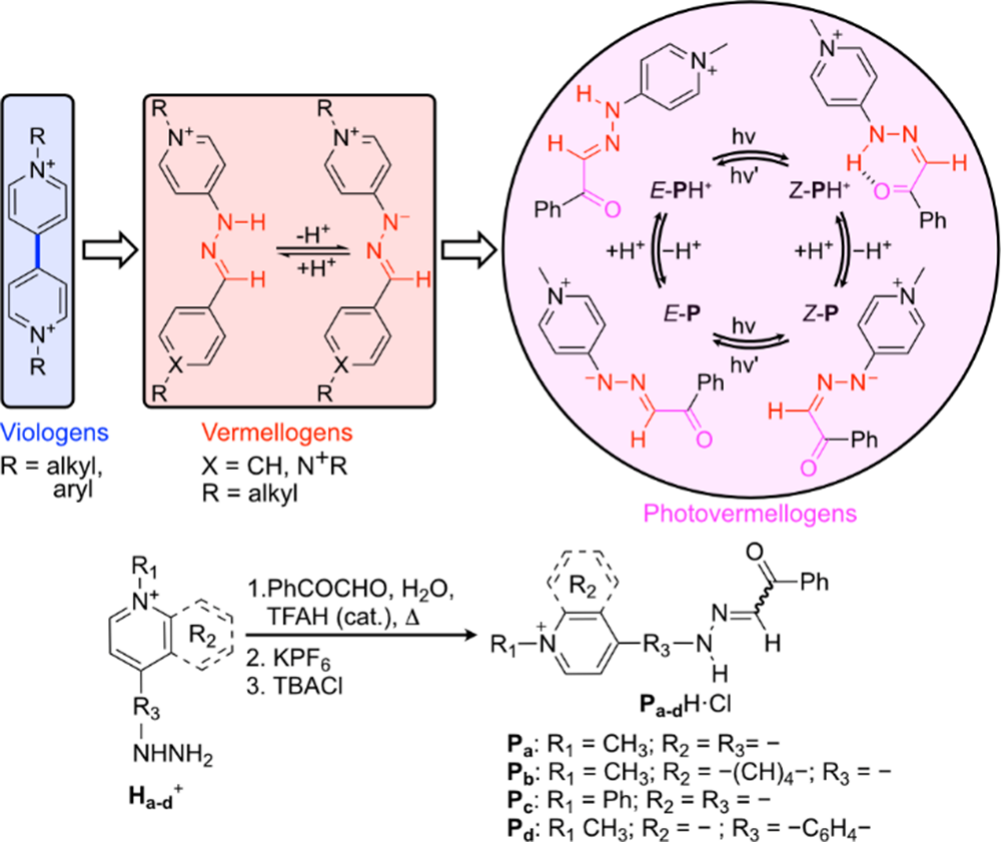
From Viologens to Photovermellogens^a^

a Top: Structural evolution into photovermellogens. Bottom: Synthesis of photovermellogens.

## 2 Results and Discussion

### 2.1 Synthesis and Structural Characterization

Regarding the synthesis of the intended compounds, the employed reaction conditions induced in all cases the quantitative self-assembly of the reactants into single products. This end was corroborated by^1^H NMR monitoring, which conveniently showed the disappearance of the reactants, and the advent of the diagnostic HC=N resonances for the acylhydrazone moiety at δ = 7.0–8.5 ppm (Figure S1, S1). The targeted water-soluble salts **P**_a–d_H·Cl were obtained in good yields, by precipitating the cations from the reaction mixture with KPF_6_, and subsequent ion metathesis with Bu_4_NCl. All compounds “as-synthesized” were extensively characterized by 1D/2D NMR (Figure 1a) and HR-ESI MS.^16^ Except for **P**_d_H·PF_6_/Cl (isolated as an 89/11 mixture of *E*/*Z* isomers), the other salts were obtained solely as the corresponding *E* forms, with NOESY NMR experiments in organic media showing defined NOE peaks between the *syn*-periplanar protons on the HC=N–NH moieties (Figure 1b). Further structural evidence for the identity of all compounds but **P**_d_H·PF_6_/Cl, was obtained by SC-XRD, with the cations in the solid state showing both the *E* configuration of the C=N bond, and a preferred *syn* disposition of the carbonyl group regarding the HC=N proton (Figure 1a). All obtained compounds were stored as solids in the refrigerator, protected from light. No signs of degradation were observed after long time storage (1 year). In solution, the compounds remained stable in the dark with no significant changes of the spectroscopic features being observed after 1 week at pD 5.5 (Figures S126–S129, SI1). This implies high hydrolytic stability, as inferred from our design rationale.

**Figure 1:**
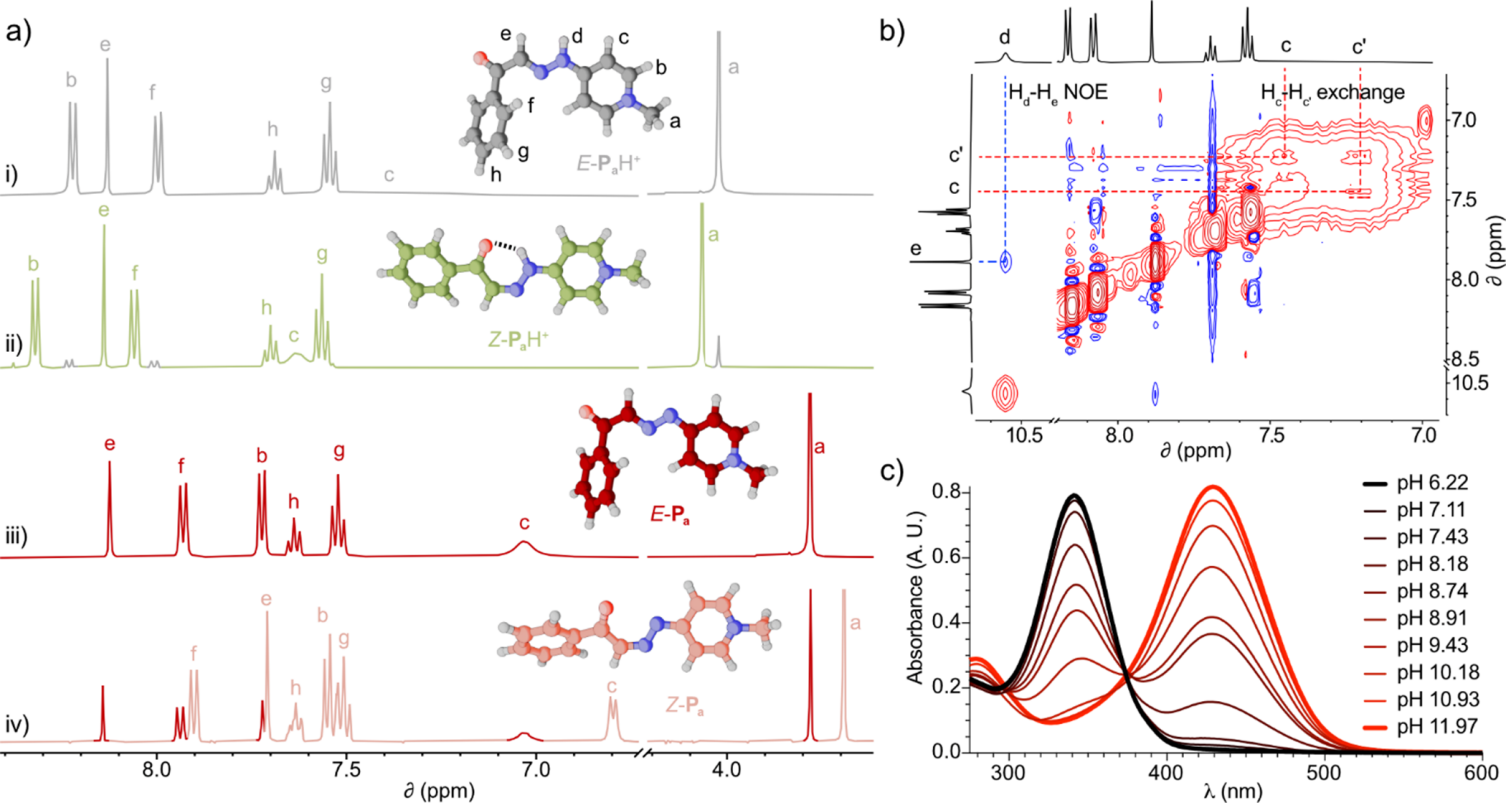
Spectroscopic features of photovermellogens, exemplified for **P**_a_H·Cl: (a) ^1^H NMR spectra (500 MHz, D_2_O) for 5 mM *E*-**P**_a_H·Cl: (i) pD = 6; (ii) irradiation at 254 nm, pD 6; (iii) pD 12; and (iv) irradiation at 254 nm, followed by adjustment of pD to 13.^17^ The main species in each spectrum are represented by representative geometries obtained from (i): SC-XRD data and (ii–iv): optimization at the density functional theory level (PBEh-3c+CPCM).^18,19^ Color code: H (light gray), N (blue), and O (red). Carbon atoms are color-coded according to isomer and protonation state as gray [*E*-**P**_a_H^+^], green [*Z*-**P**_a_H^+^], dark red [*E*-**P**_a_], and salmon [*Z*-**P**_a_]. (b) Partial EXSY/NOESY NMR spectra (CD_3_CN, 500 MHz) for *E*-**P**_a_H·PF_6_. (c) UV–vis spectra of 20 μM *E*-**P**_a_H·Cl in buffered solutions of varying pH.

### 2.2 Stimuli-Responsiveness

With the photovermellogens synthesized and fully characterized, their acid–base properties were next investigated by UV–vis spectroscopy to determine whether the pH-responsiveness characteristic of parent vermellogens was retained. Indeed, **P**_a–d_H·Cl reproduced the archetypical absorption changes of vermellogens (Figures 1c and S130–S137, SI1).^11,13,14^ In essence, the characteristic intense π*–*π* absorption bands of the cations undergo a considerable bathochromic shift upon deprotonation (Table 1), as also confirmed by our quantum chemical calculations (Figure S2, S2),^16^ causing a notable color change from yellow to red. This pronounced shift allowed the estimation of p*K*_a_ values for *E*-**P**_a–d_H^+^ *via* UV–vis absorption titrations, which, as for other vermellogens, fall within the biologically relevant pH range (Figure 1c, Table 1 and Figures S130–S137, SI1). Changes in the protonation state were also easily monitored by ^1^H NMR spectroscopy, with the gain in electronic density translating in a substantial shielding of resonances of the corresponding conjugate bases *E*/*Z*-**P**_a–d_ (Figure 1a).^17^ As already observed for *E*-**P**_a–d_H^+^, the deprotonated species were also thermally stable in the absence of light.

**Table 1:** Relevant Physicochemical Data for **P**_a–d_H·Cl in water

	p*K*_a_ (*E*-**P**H^+^)^a^	p*K*_a_ (PSD)^b^	λ_max_/ nm^c^ (*E*-**P**H^+^) [*ε*/ M^–1^·cm^–1^]^d^	λ_max_/nm^c^ (*Z*-**P**H^+^) [*ε*/ M^–1^·cm^–1^]^d^	λ_max_/ nm^c^ (*E*-**P**) [*ε*/ M^–1^·cm^–1^]^d^	λ_max_/ nm^c^ (*Z*-**P**) [*ε*/ M^–1^·cm^–1^]^d^	Φ_*E*→*Z*_ ^e^	PSD^f^ *E*→*Z*	Φ_*Z*→*E*_ ^e^	PSD^f^ *Z*→*E*	*k*_*Z*→*E*_ ^g^ (10^–4^ s^–1^)
**P**_a_H^+^	8.8 ± 0.2	10.4 ± 0.2	342 [41,000]	352 [43,000]	429 [39,000]	420 [18,000]	0.20	93% *Z*	0.001	67% *E*	4.1
**P**_b_H^+^	7.2 ± 0.1	8.8 ± 0.2	378 [42,000]	384, [36000]	456 [42,000]	445 [13,000]	0.38	93% *Z*	0.002	57% *E*	9.8
**P**_c_H^+^	7.8 ± 0.1	9.9 ± 0.1	349 [49,000]	359 [43,000]	432 [46,000]	420 [15,000]	0.34	98% *Z*	0.008	55% *E*	2.4
**P**_d_H^+^	11.2 ± 0.1	–^h^	411 [66,000]	–^h^	500 [69,000]	–^h^	–^h^	–^h^	–^h^	–^h^	–^h^

a Acidity constant of the cationic *E* form.

b Apparent acidity constant of the cationic *Z* form, as present in the photostationary state (88–97% *Z*).

c Maximum of the longest-wavelength absorption band.

d Molar absorption coefficient; error ± 5%.

e Photoisomerization quantum yield for irradiation at 254 nm (*E*→*Z*) or 440 nm (*Z*→*E*) at pH 6; error ± 20%. The quantum yields for *E*→*Z* isomerization upon irradiation at 365 nm are very similar: **P**_a_H^+^ – 0.22, **P**_b_H^+^ – 0.35, **P**_c_H^+^ – 0.34.

f Photostationary state distribution (PSD) upon irradiation at 254 nm (*E*→*Z*) or >420 nm (*Z*→*E*) at pD 6. The PSD for upon irradiation at 365 nm (*E*→*Z*) are marginally smaller: **P**_a_H^+^ – 87% *Z*, **P**_b_H^+^ – 92% *Z*, **P**_c_H^+^ – 93% *Z*.

g Unimolecular rate constant for the thermal back-isomerization upon heating to 60 °C (**P**_a_H^+^ at pD 6) or 70 °C (**P**_b_H^+^ at pD 5; **P**_c_H^+^ at pD 6).

h No data obtained due to the lack of photoisomerization.

The knowledge of the p*K*_a_ values enabled the decoupling of acid–base effects from the investigation of the light-responsiveness of photovermellogens. These effects were separately examined for **P**_a–d_H^+^ and their conjugate bases, each appropriately prepared by dissolving the “as-synthesized” salts in buffered solutions at pH = p*K*_a_ ± 2.

Photoisomerization studies were subsequently carried out in acidic medium (pH = p*K*_a_ – 2) in order to assess the photoresponse of the protonated species. Under these conditions, the anticipated photoisomerization of the thermally stable *E*-**P**_a–d_H^+^ species was investigated by UV–vis absorption spectroscopy under irradiation at 254 nm. Except for *E*-**P**_d_H^+^, which showed no detectable changes, irradiation promoted the formation of *Z*-**P**_a–c_H^+^ isomers, evidenced by a gradual red shift of the π–π* absorption band and the expected first-order kinetic behavior of the processes (Figures 2a and S138–S143, SI1).^16^ Consistent with related (acyl)­hydrazone-based switches,^7–10^ the photoreactions displayed quantum yield of 0.2–0.4 and reached excellent photostationary-state distributions (≈95%), as verified by relative integration of diagnostic ^1^H NMR signals (Figure 1a and S149–S154, SI1). It is noteworthy that the irradiation wavelength can be extended to 365 nm, yielding virtually the same observations as for 254 nm irradiation (see footnotes of Table 1).^16^ Irradiation also produced clear NMR spectral changes that support the occurrence of the *E*→*Z* isomerization. In essence, the signals H_f–h_ of the phenyl moiety were only slightly affected, whereas those of the pyridinium and acylhydrazone fragments shifted markedly, consistent with the formation of an intramolecular hydrogen bond in the *Z*-**P**_a–c_H^+^ species. The reverse *Z*→*E* transformation of the protonated forms **P**_a–c_H^+^ was also achieved upon irradiation at λ > 420 nm, giving lower quantum yields and less favorable PSDs (Figures S145–S147, S149–154, S1). Thermal reversion can occur as well, however, in a significantly slower time scale as indicated by the measured rate constants (Table 1 and Figures S155–S159, SI1). For the example of *Z*-**P**_a_H^+^ the activation energy was determined as *E*_A_ = 17.4 kcal·mol^–1^ and a half-life of the *Z* form of ca. 9 h at room temperature (298 K) was extrapolated (Figures S156–S157, SI1).

**Figure 2:**
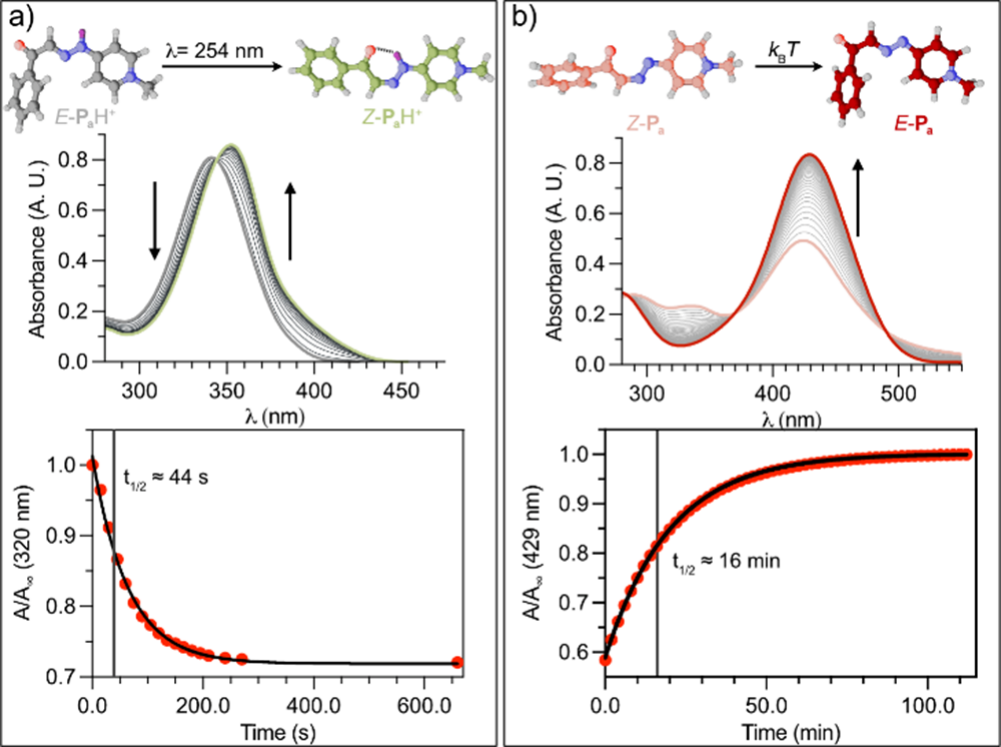
(a) Variation over time of the absorption spectrum of *E*-**P**_a_H·Cl (21 μM, pH = 6) upon irradiation at 254 nm, and fitting of the absorbance ratio at 320 nm vs time to first-order kinetics. (b) Variation over time of the absorption spectrum at r.t. of a *Z*-**P**_a_-enriched solution at pH 13 (prepared as described in the main text), and fitting of the absorbance ratio at 429 nm vs time to a first-order kinetics.

Having confirmed the photochemical behavior of the protonated forms, we next explored their light-responsiveness in the deprotonated state. Following the same procedure used for their cationic counterparts, the neutral *E*-**P**_a–d_ species were investigated in basic media (pH = p*K*_a_ + 2), where photoisomerization studies showed a markedly different behavior. While *E*-**P**_a–c_H^+^ exhibited the dual photochemical features of P-/T-type switches (see above),^1^ the corresponding conjugate base *E*-**P**_a–c_ showed no detectable *E*→*Z* conversion upon irradiation (Figures S160–S162, SI1).^20^ To probe whether *Z*-**P**_a–c_ could nonetheless be generated indirectly, we explored a consecutive photoisomerization–deprotonation sequence. Thus, *E*-**P**_a–c_H^+^ was first irradiated at 254 nm and pH 6 to enrich the *Z* population, followed by immediate adjustment of the solution to pH 13. Under these conditions, transient spectroscopic features were observed, consistent with the kinetic trapping of the metastable *Z*-**P**_a–c_ species. UV–vis spectra displayed similar maxima and bathochromic shifts to those of the deprotonated *E*-isomers (Table 1 and Figures S163–S168, S1).^20^ More revealingly, the ^1^H NMR spectra exhibited two distinct sets of signals in slow exchange on the NMR time scale, consistent with *E*/*Z* mixtures of the corresponding conjugate bases (Figure 1a). The signals attributable to the new species were assigned by COSY experiments, which confirmed both the expected multiplicity and relative integration for *Z*-**P**_a–c_ (Figures S41, S75 and S105 SI1) and revealed a clear deprotonation-induced shielding compared to *Z*-**P**_a–c_H^+^. These spectroscopic observations enabled the estimation of virtual p*K*_a_ values for the *Z*-enriched solutions (Table 1 and Figures S163–168, SI1), which confirmed the reduced acidity of the NH group in the *Z*-isomers, presumably arising from intramolecular hydrogen-bond formation.

To gain further insight into the behavior of the metastable *Z*-bases, absorbance changes of the kinetically trapped state were monitored over time in the dark for the whole **P**_a–c_ series. In all cases, the data fitted well to the expected unimolecular kinetics for thermally induced *Z*→*E* processes (Figures 2b, S169, S171–S172, SI1). Notably the thermal half-life increased dramatically upon substitution, from ca. 16 min for **P**_a_ to several hours for **P**_b_ and **P**_c_. Using **P**_a_H·Cl as a representative model, the photochemically induced back-isomerization was additionally investigated under irradiation at λ > 495 nm (Figure S170, SI1), revealing that the photo- and thermally induced *Z*→*E* processes operate on comparable time scales, with the thermal pathway being slightly slower.

Overall, the experimental results for the **P**_a–c_H·Cl series reveal a behavior characteristic of photoswitches with light-responsive basicity, where *E*→*Z* photoisomerization drives a reversible shift in p*K*_a_ between two ground-state forms.^5,6^ In acidic medium, the protonated species **P**_a–c_H^+^ display the typical features of acylhydrazone-type photoswitches, combining both P-/T-type responses.^1^ Upon deprotonation, however, the conjugate bases **P**_a–c_ lose their ability to undergo the forward *E*→*Z* photoisomerization. This contrasting photoresponse is assigned to the intramolecular C=O^···^H–N HB motif, which is essential for an efficient *E*→*Z* conversion.

### 2.3 Computational Study

To further rationalize the photochemical properties of photovermellogens at the molecular level we performed quantum-mechanical calculations on **P**_a_H^+^ as a representative model (see SI2 for details and expanded discussion). We first investigated the photoisomerization mechanism of the acidic form of the species (Figure 3). As shown, at the experimental irradiation conditions (λ = 254 nm), the bright S_2_ state (π→π*) of *E*-**P**_a_H^+^ becomes populated. From there, an S_1_/S_2_ minimum energy conical intersection (MECI) that involves a barrier of about 5.0 kcal·mol^**–**1^ with respect to a local S_2_ minimum, enables progression to the *n*→π* S_1_ excited state (θ ∼ 133°). The *E*-isomer can either relax to the *n*→π* S_1_, which eventually relaxes back to the ground state, or convert to the *Z*- form passing a S_0_/S_1_ MECI (θ ∼ 90°, schematic red path in Figure 3). This step involves a negligible barrier of just 0.3 kcal·mol^**–**1^ relative to the S_1_ minimum, and poses one path for *E*→*Z* photoisomerization. Since the n→π* state is essentially dark, with a computed oscillator strength of f_01_ = 0.00, it is unlikely to be efficiently populated by irradiation, and the excited-state trajectory is expected to start from S_2_. One should note that the relative ground-state stability of the *Z*/*E*-isomers in Figure 3 is inverted when a variational, solvation-inclusive theory level is used (Table 1, SI2). In that case, the *E*-form is more stable by about 2 kcal·mol^–1^. Nevertheless, the inverted ground-state energetics from the hh-TDA^21,22^ calculations (Figure 3), is not expected to affect the interpretation of the photoisomerization mechanism based on the S_1_ and S_2_ state surfaces.

**Figure 3:**
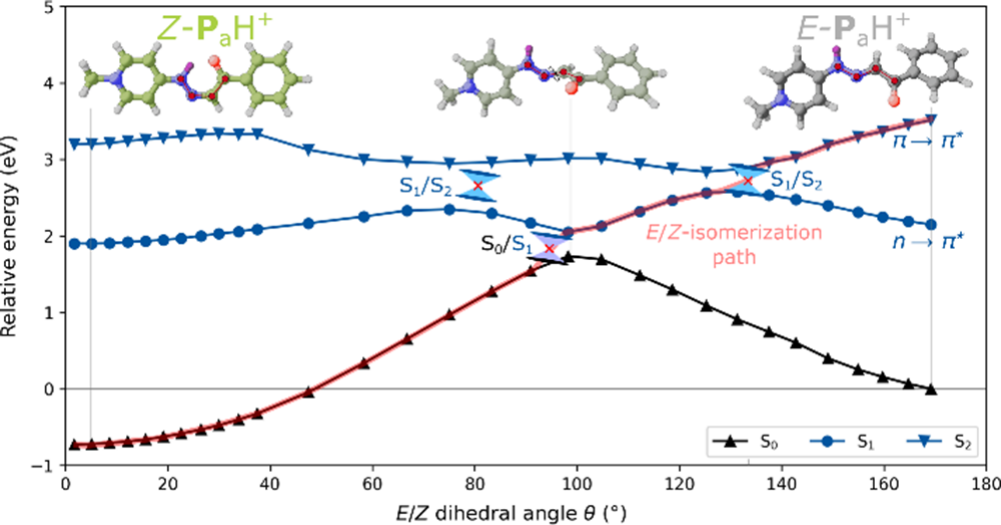
Potential energy curve (hh-TDA^21,22^ -PBEh-3c^18,23^ //GFN2-xTB^24^+ALPB^25^) along the *E*/*Z*-isomerization coordinate θ of **P**_a_H^+^ for the ground state S_0_ (black upper triangles) and the singlet excited states S_1_ (blue circles) and S_2_ (blue lower triangles). Relevant minimum energy conical intersections (see SI2 for details) and a possible photoisomerization pathway (red, dashed line) are displayed.

We performed an analogous set of calculations to characterize the deprotonated form **P**_a_.^16^ Associated with the observed red shift is the fact that now the visible π→π* becomes lowest in energy. This state is mostly comprised of contributions from the highest occupied molecular orbital (HOMO) and lowest unoccupied molecular orbital (LUMO). Given that the HOMO–LUMO energy gap is notably smaller for the deprotonated form **P**_a_, this rationalizes its pronounced red-shifted absorption as compared to the protonated form. These changes in the π→π* states are also recognizable looking at the natural transition orbitals (NTOs)^26^ given in Figures S2–S3 in SI2. We identified MECIs comparable to those of **P**_a_H^+^, which suggest photoisomerization to be feasible. However, there exists no structural motif which would prevent back isomerization to *E*-**P**_a_. While the calculated barrier for thermal *E*/*Z*-isomerization Δ*G* ^‡^ are clearly very high in free energy, we still find a notable decrease for **P**_a_ (33.0 kcal·mol^**–**1^) over **P**_a_H^+^ (43.6 kcal·mol^**–**1^). Δ*G* ^‡^ of **P**_a_ is similar in size to results known for other hydrazone photoswitches in literature,^27,28^ and resembles an inversion-type double bond rotation. On the other hand, thermal isomerization in the protonated form **P**_a_H^+^ is associated with an adiabatic rotation-type transition state. While this is more similar to azobenzene, the possibility of a nonadiabatic rotation-type isomerization as found for azobenzene,^28^ was not explored in the present work, but certainly deserves more attention in the future.

## 3 Conclusions

The experimental and computational results converge into a coherent picture, in which protonation controls the efficiency and metastability of photovermellogens. Protonated species **P**_a–c_H^+^ behave as P/T-type acylhydrazone switches, while deprotonation disrupts the hydrogen-bond motif that enables *E*→*Z* photoisomerization, allowing only *Z*→*E* relaxation. Calculations support this mechanism, showing low-barrier excited-state crossings for **P**_a_H^+^ and destabilization of the *Z*-form upon deprotonation. Overall, photovermellogens constitute a new class of synthetically straightforward accessible and modular acylhydrazone-based photoswitches, which uniquely combine hydrolytic stability with tunable photochemical and acid–base properties. Their high resistance to hydrolysis, conferred by the electron-withdrawing pyridinium framework, ensures reliable operation in aqueous environments—a feature that remains challenging for many (acyl)­hydrazone systems.^9^ Displaying photoswitchable basicity, they provide a distinct platform for light-induced pH modulation in water through *E*→*Z* photoisomerization, extending aqueous photobase chemistry beyond spiropyrans^29^ and related families.^4^ Owing to their favorable properties, photovermellogens emerge as promising, water-compatible scaffolds for next-generation molecular photoswitches.

## 4 Safety Statement

No unexpected or significant hazards beyond those commonly encountered in routine synthetic chemistry were identified during this work. The compounds described herein should be handled with care, avoiding inhalation and skin or eye contact. All procedures were conducted in a well-ventilated fume hood. No unexpected or significant hazards beyond those commonly encountered in routine synthetic chemistry were identified.

## Supplementary Material







## Data Availability

The data underlying this study are available in the published article and its Supporting Information.

## Notes

SI1: Experimental details, synthetic procedures and characterization data for new compounds, hydrolytic stabilities, titration data for the determination of p*K*_a_ values, isomerization photochemical data, crystallographic data for *E*-**P**_a_H·PF_6_, *E*-**P**_b_H·I and *E*-**P**_c_H·PF_6_ (CCDC: 2486929, 2487028 and 2486961, respectively), and additional figures (PDF)
SI2: Computation of free energies, absorption spectra, and thermal and complementary data for thermal isomerization and photoisomerization (PDF)
SI3: Coordinates for all computed structures and outputs of quantum chemical calculations (ZIP)
