# Supplementary material for: Photovermellogens: Minimalistic Pyridinium-Based Acylhydrazones with Photoswitchable Basicity for Operation in Aqueous Media
Source: J Org Chem. 2026 May 4;91(19):6559–65. doi: 10.1021/acs.joc.5c02962 (PMC13185115; doi:10.1021/acs.joc.5c02962)
Supplement: Supplementary file 1 [file jo5c02962_si_001.pdf]

Supporting Information

Photovermellogens: Minimalistic Pyridinium-Based Acylhydrazones with Photoswitchable Basicity for Operation in Aqueous Media

Alejandro Vila,[a] Francisco G. Blandón-Cumbreras,[b] Mike Pauls,[c] Mauro Díaz-Abellás,[a] Arturo Blanco-Gómez,[a] Carlos Peinador,[a] Patricia Remón,[b] Christoph Bannwarth,[c]* Uwe Pischel,[b]*and Marcos D. García.[a]*

[a] CICA-Centro Interdisciplinar de Química e Bioloxía and Departamento de Química, Facultade de Ciencias, Universidade da Coruña, 15071 A Coruña, Spain.

[b] CIQSO – Center for Research in Sustainable Chemistry and Department of Chemistry, University of Huelva, 21071 Huelva, Spain.

[c] Institute of Physical Chemistry, RWTH Aachen University, Aachen 52074, Germany.

TABLE OF CONTENTS:

1. General procedures....S4
2. Synthetic procedures and characterization data. S6
2.1. Synthesis and characterization data of $\mathbf{P}_a$H·Cl/PF_6....S6
2.1.1. NMR data for the species assigned as *E*-$\mathbf{P}_a H^+$. S9
2.1.1.1. Aqueous medium....S9
2.1.1.2. Organic medium. S14
2.1.2. NMR data for the species assigned as *Z*-$\mathbf{P}_a H^+$. S18
2.1.2.1. Aqueous medium....S18
2.1.2.2. Organic medium. S23
2.1.3. NMR data for the species assigned as *E*-$\mathbf{P}_a$. S27
2.1.4. NMR data for the species assigned as *Z*-$\mathbf{P}_a$....S31
2.2 Synthesis and characterization data of **1**·I S33
2.3 Synthesis and characterization data of $\mathbf{H}_b$·I S36
2.4 Synthesis and characterization data of $\mathbf{P}_b$H·Cl/PF_6....S39
2.4.1.1. Aqueous medium....S41
2.4.1.2. Organic medium. S45
2.4.2. NMR data of the species assigned as *Z*-$\mathbf{P}_b H^+$. S47
2.4.2.1. Aqueous medium....S47
2.4.2.2. Organic medium. S51
2.4.3. NMR data for the species assigned as *E*-$\mathbf{P}_b$. S53
2.4.4. NMR data for the species assigned as *Z*-$\mathbf{P}_b$....S55
2.5 Synthesis and characterization data of $\mathbf{H}_c$·Cl S57
2.6 Synthesis and characterization data of $\mathbf{P}_c$H·Cl/PF_6....S60
2.6.1. NMR data for the species assigned as *E*-$\mathbf{P}_c H^+$. S62
2.6.1.1. Aqueous medium....S62
2.6.1.2. Organic medium. S66
2.6.2 NMR data for the species assigned as *Z*-$\mathbf{P}_c H^+$. S68
2.6.2.1. Aqueous medium....S68
2.6.2.2. Organic medium. S72
2.6.3. NMR data for the species assigned as *E*-$\mathbf{P}_c$. S74
2.6.4. NMR data for the species assigned as *Z*-$\mathbf{P}_c$....S76
2.7 Synthesis and NMR data of **2**·I S78
2.8. Synthesis and NMR data of $\mathbf{H}_d$·I S81
2.9. Synthesis and characterization data of $\mathbf{P}_d$H·Cl. S84

2.9.1. NMR data for the species assigned as E-$\mathbf{P}_d$H$^+$. S86
2.9.1.1. Aqueous medium. S86
2.9.1.2. Organic medium. S90
2.9.2. NMR data of the species assigned as E-$\mathbf{P}_d$. S92
3. Hydrolytic stability of $\mathbf{P}_{a\text{-}d}$H·Cl compounds. S93
4. Determination of pK_a values for E-$\mathbf{P}_{a\text{-}d}$H$^+$ by UV-Vis spectroscopy. S96
4.1. E-$\mathbf{P}_a$H$^+$. S97
4.2. E-$\mathbf{P}_b$H$^+$. S98
4.3. E-$\mathbf{P}_c$H$^+$. S99
4.4. E-$\mathbf{P}_d$H$^+$. S100
5. Isomerization Photochemical Data S101
5.1. E-$\mathbf{P}_{a\text{-}c}$H$^+$→Z-$\mathbf{P}_{a\text{-}c}$H$^+$ photoisomerization kinetics. S101
5.1.1. E-$\mathbf{P}_a$H$^+$→Z-$\mathbf{P}_a$H$^+$ S101
5.1.2. E-$\mathbf{P}_b$H$^+$→Z-$\mathbf{P}_b$H$^+$ S102
5.1.3. E-$\mathbf{P}_c$H$^+$→Z-$\mathbf{P}_c$H$^+$ S103
5.1.4. E-$\mathbf{P}_d$H$^+$→Z-$\mathbf{P}_d$H$^+$ S104
5.2. Z-$\mathbf{P}_{a\text{-}c}$H$^+$→E-$\mathbf{P}_{a\text{-}c}$H$^+$ photoisomerization kinetics. S105
5.2.1. Z-$\mathbf{P}_a$H$^+$→E-$\mathbf{P}_a$H$^+$ S105
5.2.2. Z-$\mathbf{P}_b$H$^+$→E-$\mathbf{P}_b$H$^+$ S105
5.2.3. Z-$\mathbf{P}_c$H$^+$→E-$\mathbf{P}_c$H$^+$ S106
5.3. Fatigue resistance. S107
5.4. Photostationary state distributions. S108
5.4.1. $\mathbf{P}_a$H·Cl S108
5.4.2. $\mathbf{P}_b$H·Cl S110
5.4.3. $\mathbf{P}_c$H·Cl S112
5.5. Z-$\mathbf{P}_{a\text{-}c}$H$^+$→E-$\mathbf{P}_{a\text{-}c}$H$^+$ thermal back-isomerization kinetics. S114
5.5.1. Z-$\mathbf{P}_a$H$^+$→E-$\mathbf{P}_a$H$^+$ S114
5.5.2. Z-$\mathbf{P}_b$H$^+$→E-$\mathbf{P}_b$H$^+$ S117
5.5.3. Z-$\mathbf{P}_c$H$^+$→E-$\mathbf{P}_c$H$^+$ S118
5.6. E-$\mathbf{P}_{a\text{-}c}$→Z-$\mathbf{P}_{a\text{-}c}$ photoisomerization kinetics. S119
5.6.1. E-$\mathbf{P}_a$→Z-$\mathbf{P}_a$ S119
5.6.2. E-$\mathbf{P}_b$→Z-$\mathbf{P}_b$ S120
5.6.3. E-$\mathbf{P}_c$→Z-$\mathbf{P}_c$ S121
6. Determination of pK_a values for the PSS by UV-Vis spectroscopy S122
6.1. $\mathbf{P}_a$H·Cl. S123

6.2. $\mathbf{P}_b$H·Cl. S124
6.3. $\mathbf{P}_c$H·Cl. S125
7. Thermal Z-$\mathbf{P}_{a\text{-}c}$→E-$\mathbf{P}_{a\text{-}c}$ isomerizations from the PSS S126
8. X-RAY crystallographic data. S128
8.1. Obtention of X-Ray crystals of $\mathbf{P}_a$·H·PF_6, P_b·H·I, P_c·H·PF_6. ....S128
8.1.1. X-Ray crystallographic data of $\mathbf{P}_a$·H·PF_6 (CCDC: 2486929). S128
9.1.2 X-Ray crystallographic data of $\mathbf{P}_b$·H·I (CCDC: 2487028).............................S131
9.1.3. X-Ray crystallographic data of $\mathbf{P}_c$·H·PF_6 (CCDC: 2486961)........................S134

1. General procedures.

Starting materials were purchased from commercial suppliers and used without further purification. Compounds 4-hydrazineyl-1-methylpyridinium iodide (**H**$_a$·I) and 4-chloro-1-phenylpyridin-1-ium were prepared according to published procedures.[1] Reactions requiring microwave irradiation were carried out using an Anton Paar Monowave 300 instrument. Milli-Q water was purified with a Millipore Gradient A10 apparatus. Merck 60 F254 foils were used for thin layer chromatography.

NMR spectra were recorded on a Bruker Advance 300, 400 or 500 MHz for ^{1}H, and 75, 101 or 126 MHz for ^{13}C, equipped with a dual cryoprobe. The solvents used for NMR experiments were D_2O, DMSO-d6 or CD_3CN. Structural assignments were made with additional information from gCOSY, gHSQC, and gHMBC experiments. HPLC-MS analysis were performed using a Thermo Scientific UltiMate 3000 connected to a photo-diode array (PDA) detector using a Luna® Omega analytical column from Phenomenex (Polar-C18 stationary phase, 3 μm, 100 Å pore size, 150 × 2.1 mm). Mass spectrometry experiments were carried out in a LCQ-q-TOF Applied Biosystems QSTAR Elite spectrometer for low- and high-resolution ESI.

UV/Vis spectra for pK_a measurements were recorded on a Jasco V-650 spectrometer, and those for the photochemical or thermal kinetics on an Agilent CARY 5000 equipment. The measurements were carried out in both cases at room temperature (298 K), with the compounds dissolved in air-equilibrated phosphate buffered aqueous solutions of appropriate pH, contained in quartz cuvettes with 1 cm optical pathlength.

[1] a) Uno, K.; Sugimoto, N.; Sato, Y. *N*-aryl pyrido cyanine derivatives are nuclear and organelle DNA markers for two-photon and super-resolution imaging. *Nat. Commun.* **2021**, *12*, 2650. b) Katrizky, A. R.; Murugan, R.; Sakizadeh, K. The Reactions of Arylamines with Chelidonic Acid. *J. Heterocycl Chem.* **1984**, *21*, 1465–1467. c) Hünig, S.; Köbrich, G. Azofarbstoffe durch oxydative Kupplung, V. Synthese von 1-substituierten Pyridon-(4)-hydrazonen. *Liebigs Ann. Chem.* **1958**, *617*, 181–202. d) Blanco-Gómez, A.; Neira, I.; Barriada J. L.; Melle-Franco, M.; Peinador, C.; García, M. D. Thinking outside the "Blue Box": from molecular to supramolecular pH-responsiveness. *Chem. Sci.* **2019**, *10*, 10680–10686.

To carry out the E→Z isomerization experiments, a handheld UV lamp (Vilber Lourmat-6.LC, 254 nm and 365 nm) or a 150-W xenon lamp from Oriel with a 420-nm long-pass filter or a 440-nm band-pass filter were employed. The E-isomers were irradiated at 254 nm, 365 nm, or 440 nm, and potassium trioxalatoferrate(III) trihydrate was used as the actinometer (Φr (254 nm) = 1.25 in a 0.23 M CH3COONa / 0.05 M H2SO4 buffer solution; Φr (365 nm) = 1.21 in a 0.23 M CH3COONa / 0.05 M H2SO4 buffer solution; Φr (440 nm) = 1.01 in a 0.23 M CH3COONa / 0.05 M H2SO4 buffer solution)[2]. After determining the photon flux, the isomerization process was monitored under the same irradiation conditions by measuring the absorbance after 0, 5, 10, and 15 seconds of irradiation. The quantum yield was calculated using the following equation:[3,4]

$$\Phi_R = \frac{3m}{1000 \cdot I_{abs} \cdot \Delta\varepsilon}$$

where:

Φ_R= quantum yield of isomerization.

m = slope of the linear fit obtained by plotting absorbance changes versus time.

I_{abs}= average absorbed light intensity during irradiation.

$\Delta\varepsilon$ = change in molar extinction coefficient.

The photostationary state distributions (PSDs), were estimated by following the corresponding photochemical reaction by ^{1}H NMR spectroscopy.

[2] Hatchard, C. G.; Parker, C. A.; Bowen, E. J. A new sensitive chemical actinometer - II. Potassium ferrioxalate as a standard chemical actinometer. *Proc. Royal Soc. London* **1956**, *235*, 518-536

[3] Kuhn, H.; Braslavsky, S.; Schmidt, R. Chemical actinometry (IUPAC Technical Report). *Pure Appl. Chem.* **2004**, *76*, 2105-2146.

[4] Montalti, M.; Credi, A.; Prodi, L.; Gandolfi, M.T. (2006). Handbook of Photochemistry. (3rd ed.). CRC Press.

2. Synthetic procedures and characterization data.

2.1. Synthesis and characterization data of $P_aH{\cdot}Cl/PF_6$.

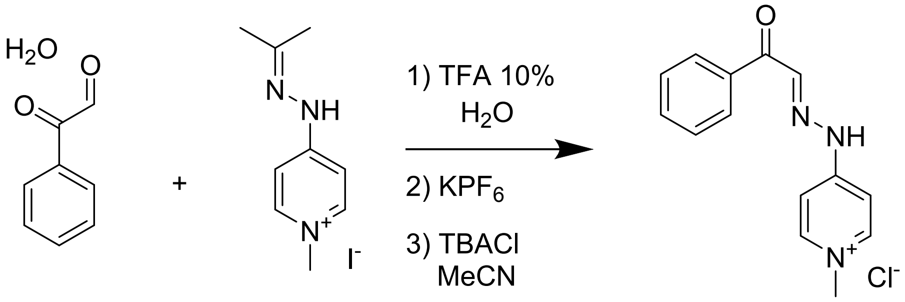

A mixture of phenylglyoxal monohydrate (144 mg, 0.947 mmol, 1.1 equiv) and 4-hydrazineyl-1-methylpyridinium iodide (**H**$_a$·I) (0.250 mg, 0.859 mmol, 1 equiv) in 35 mL of H_2O was stirred until complete solution. Then, 6.6 µL of trifluoroacetic acid were added and the solution was heated in a magnetic hot plate stirrer at 60 ºC for 16 hours. When the reaction was completed, KPF_6 (632 mg, 3.436 mmol, 4 equiv) was added to the solution. The resulting precipitate was filtered, washed with H_2O (3 × 10 mL) and Et_2O (3 × 10 mL) and dried under vacuum to yield the product **P**$_a$H·PF_6 as a yellow solid (340 mg, 92%). The solid was dissolved in acetone and a saturated solution of TBACl in acetone was added. After 1h under stirring the precipitate was filtered and washed with acetone (2 × 10 mL) and Et_2O (2 × 10 mL) and dried under vacuum, to obtain the product **P**$_a$H·Cl as a pale-yellow solid (214 mg, 58%).

HRMS (ESI) m/z: Calcd for $C_{14}H_{14}N_3O^+$ 240.1131; found 240.1126.

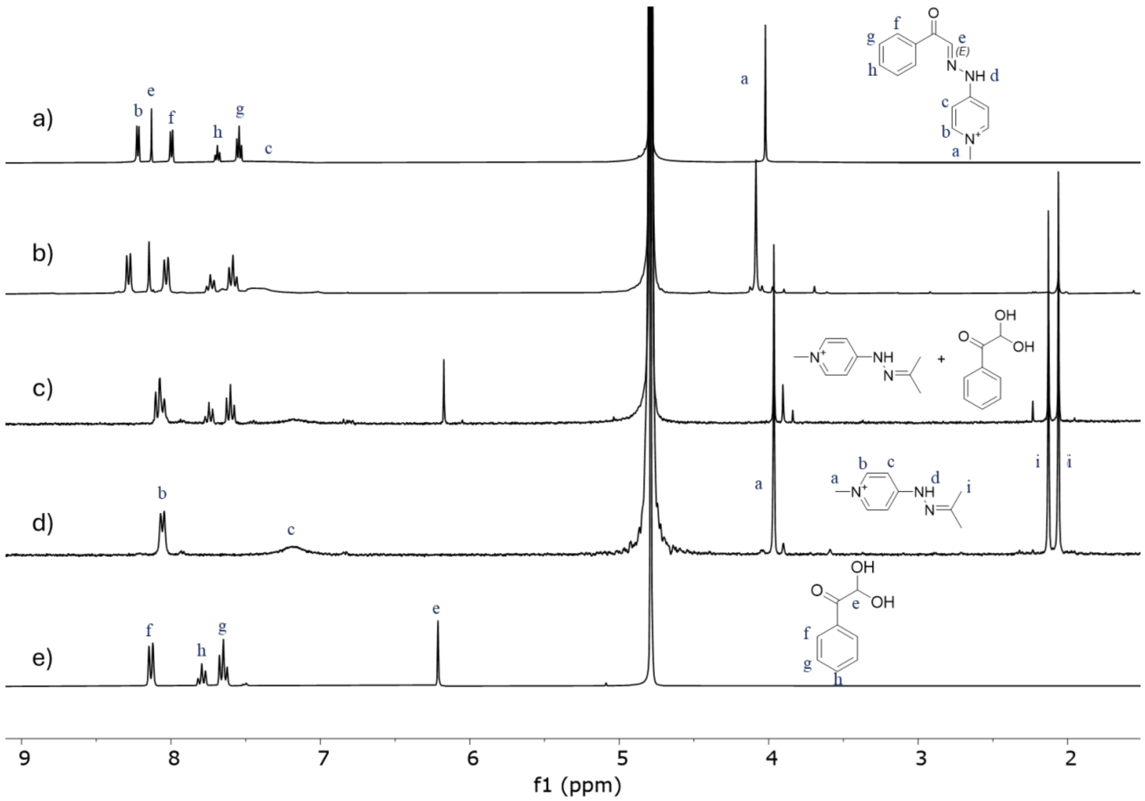

Figure S1. ^{1}H NMR stacked spectra (300 MHz, D_2O) monitoring the synthesis of $\mathbf{P}_a$H·Cl: a) isolated product as the chloride salt after redissolution. b) crude reaction mixture after 16 h at 60 °C; c) crude reaction mixture at t = 0; d) starting hydrazone reactant; e) starting phenylglyoxal reagent.

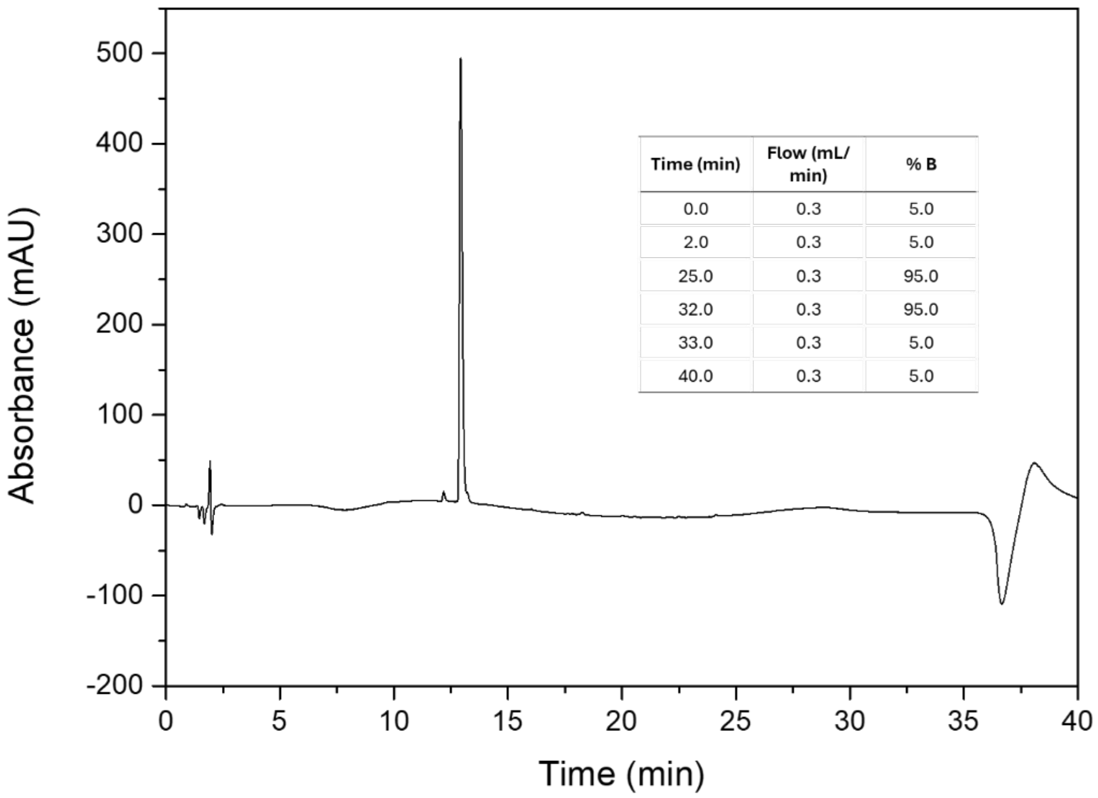

Figure S2. HPLC chromatogram (220 nm) of $\mathbf{P}_a$H·Cl at t_R = 12.93 min (Inset: separation method; A = H_2O + 0.04 % TFA, B = CH_3CN + 0.04 % TFA).

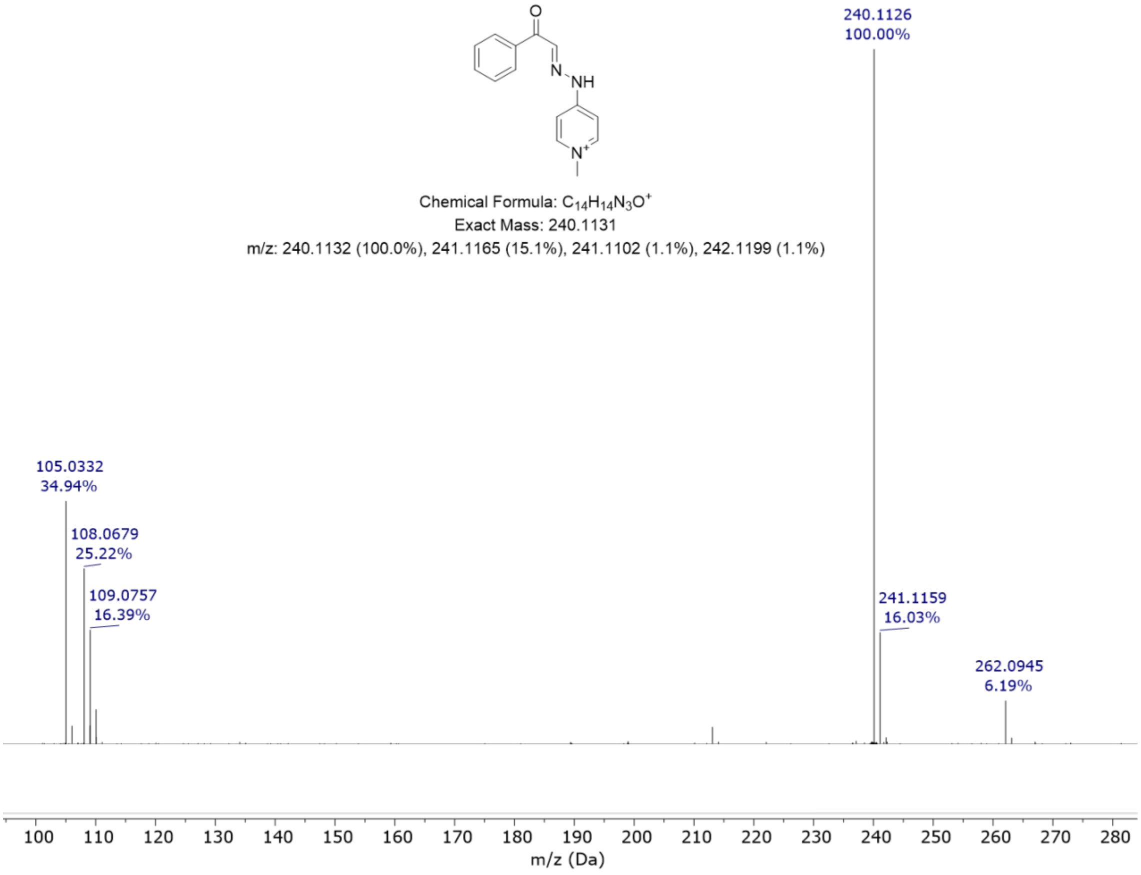

Figure S3. HRMS-ESI spectrum of $\mathbf{P}_a$H·Cl.

2.1.1. NMR data for the species assigned as *E*-P_aH^+.

2.1.1.1. Aqueous medium.

$\mathbf{P_a}$H·Cl salt was dissolved at 5 mM in a D_2O phosphate buffer solution (20 mM, pD 6).

^{1}H NMR (500 MHz, D_2O) δ (*ppm*): 8.22 (d, J = 7.2 Hz, 2H), 8.13 (s, 1H), 8.00 (d, J = 6.8 Hz, 2H), 7.69 (t, J = 7.5 Hz, 1H), 7.54 (t, J = 7.8 Hz, 2H), 7.47 – 7.21 (m, 2H), 4.02 (s, 3H).

^{13}C{^{1}H} NMR (126 MHz, D_2O) δ (*ppm*): 191.5 (C=O), 154.5 (C_{Ar}), 144.6 (CH_{Ar}), 141.5 (CH=N), 135.5 (C_{Ar}), 134.2 (CH_{Ar}), 129.7 (CH_{Ar}), 128.7 (CH_{Ar}), 110.0 (CH_{Ar}), 45.5 (CH_3).

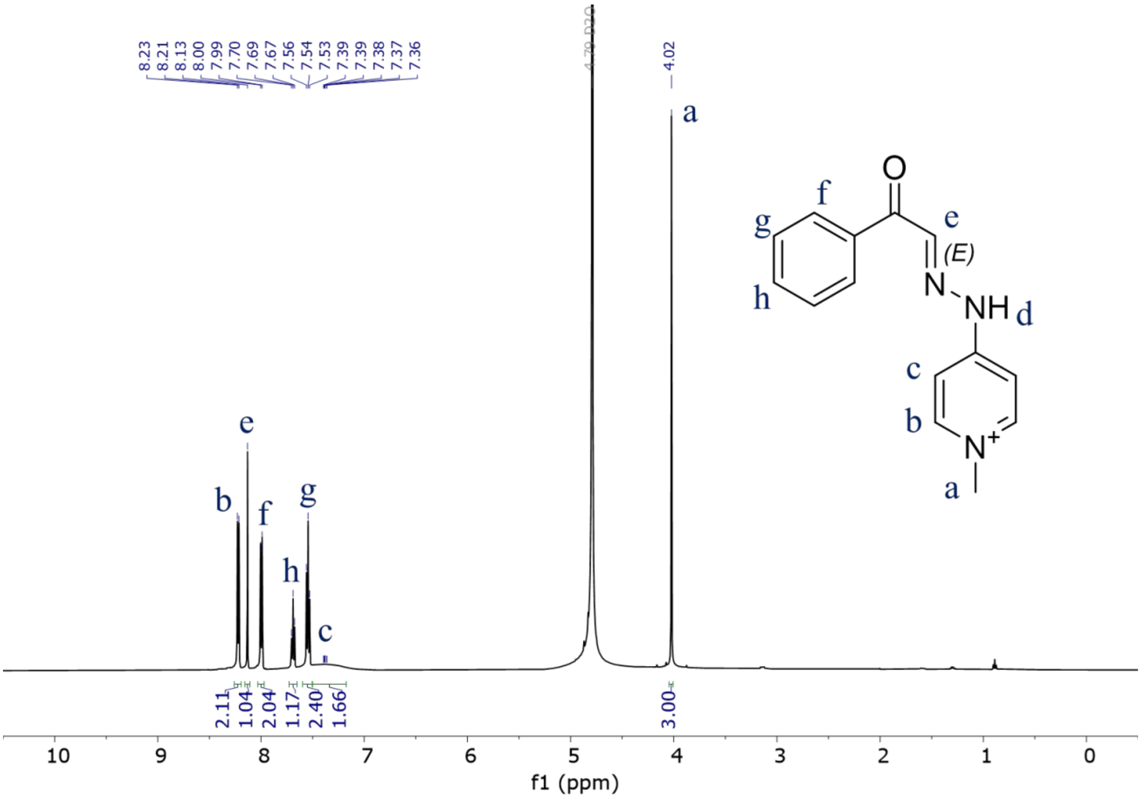

Figure S4. ^{1}H NMR (500 MHz, D_2O) spectrum of the compound $\mathbf{P_a}$H·Cl as synthesized.

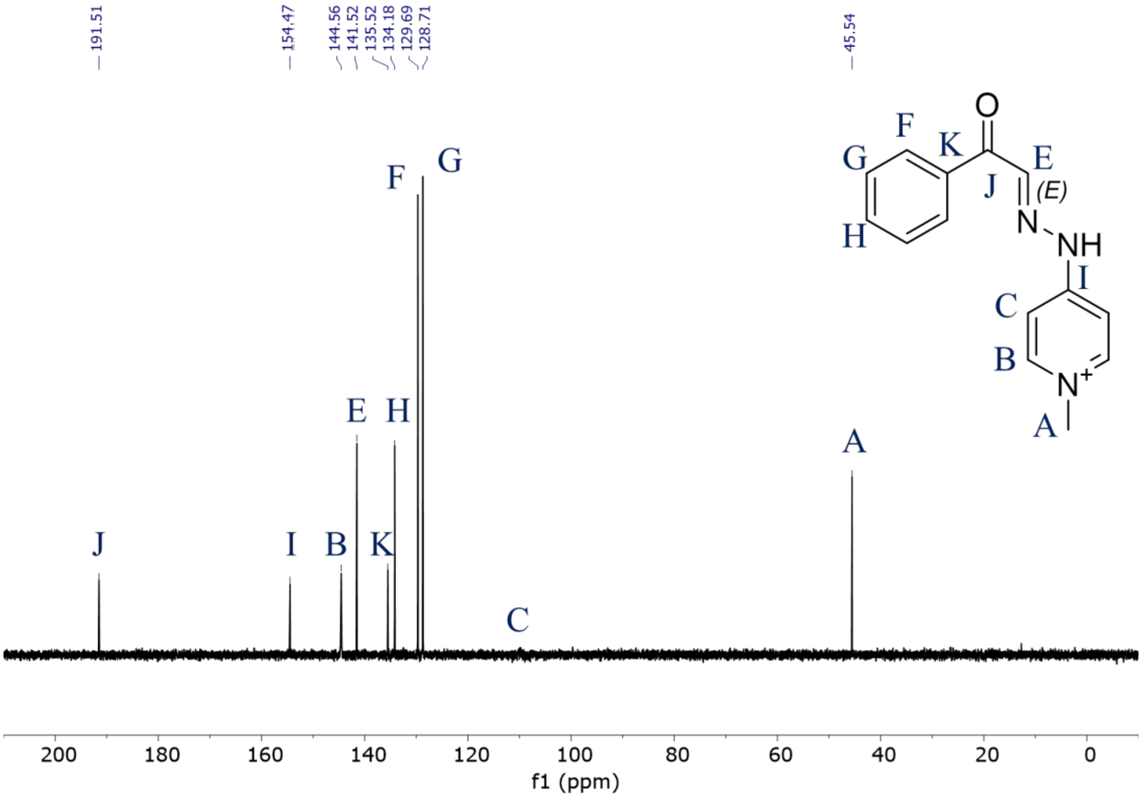

Figure S5. $^{13}C\{^1H\}$ NMR (126 MHz, D_2O) spectrum of the compound $\mathbf{P}_a$H·Cl as synthesized.

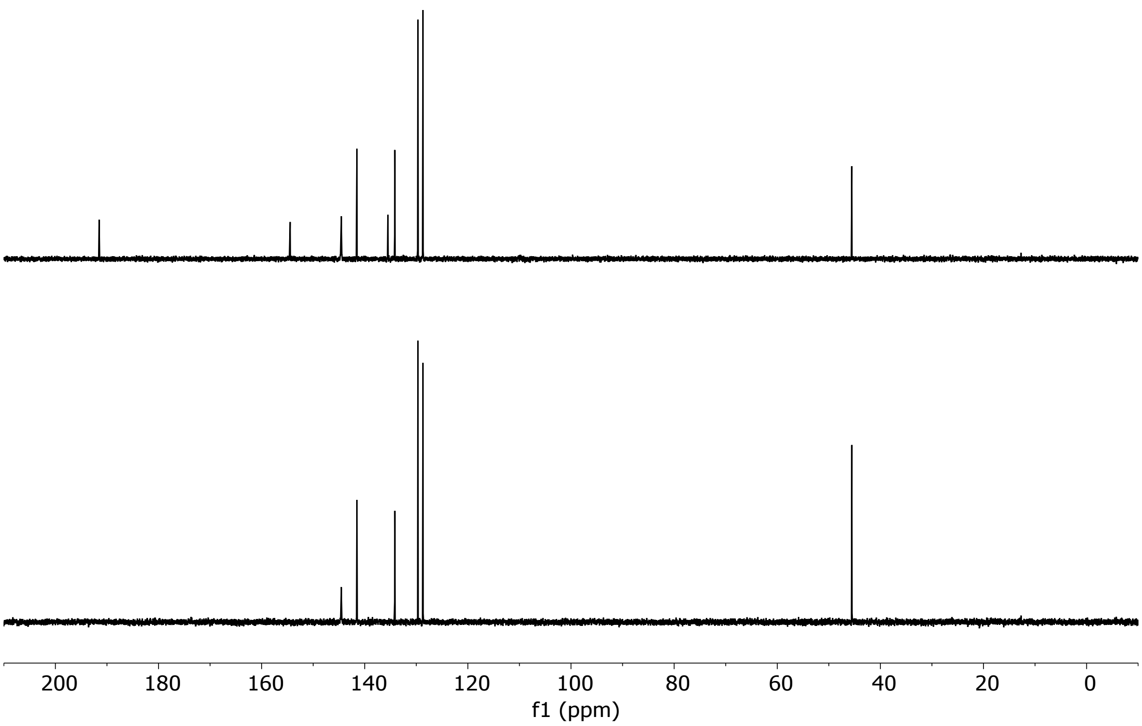

Figure S6. DEPT-135 NMR (126 MHz, D_2O) and $^{13}C\{^1H\}$ NMR (126 MHz, D_2O) stacked spectra of the compound $\mathbf{P}_a$H·Cl as synthesized.

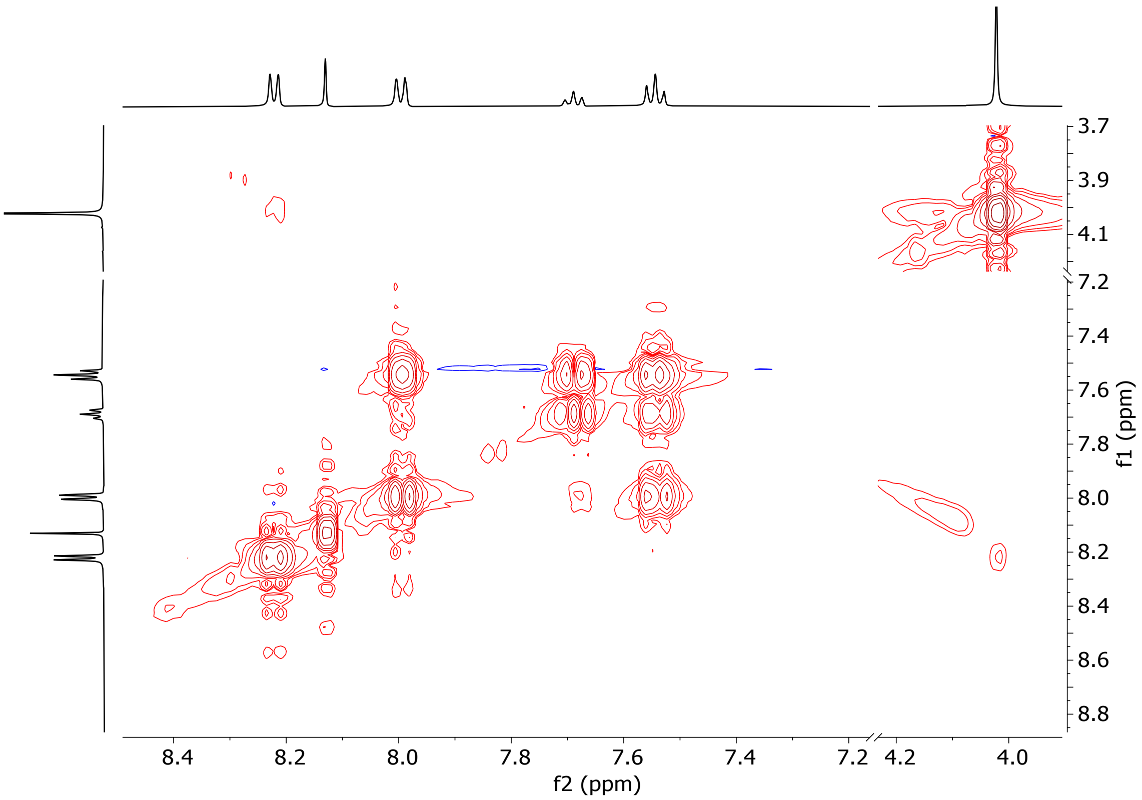

Figure S7. ^1H-^1H COSY (500 MHz, D_2O) partial spectrum of the compound $\mathbf{P}_a$H·Cl as synthesized.

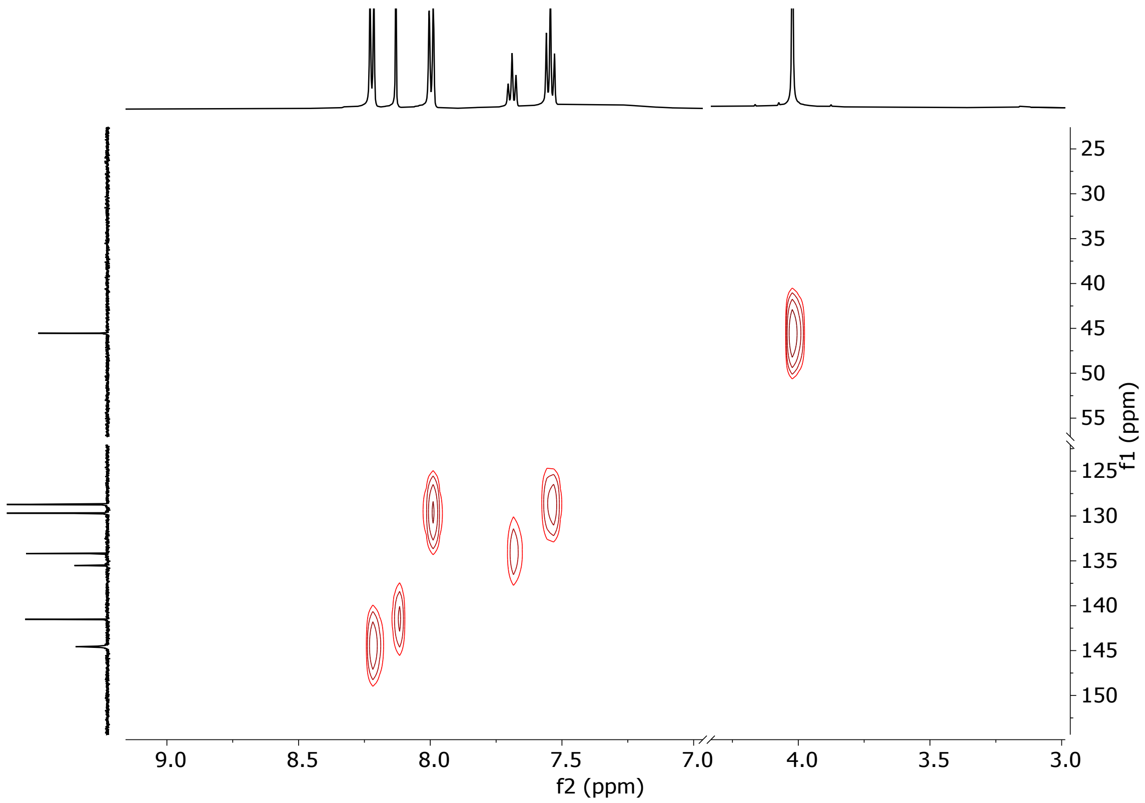

Figure S8. ^{1}H-^{13}C HSQC (500 MHz, D_2O) partial spectrum of the compound $\mathbf{P}_a$H·Cl as synthesized.

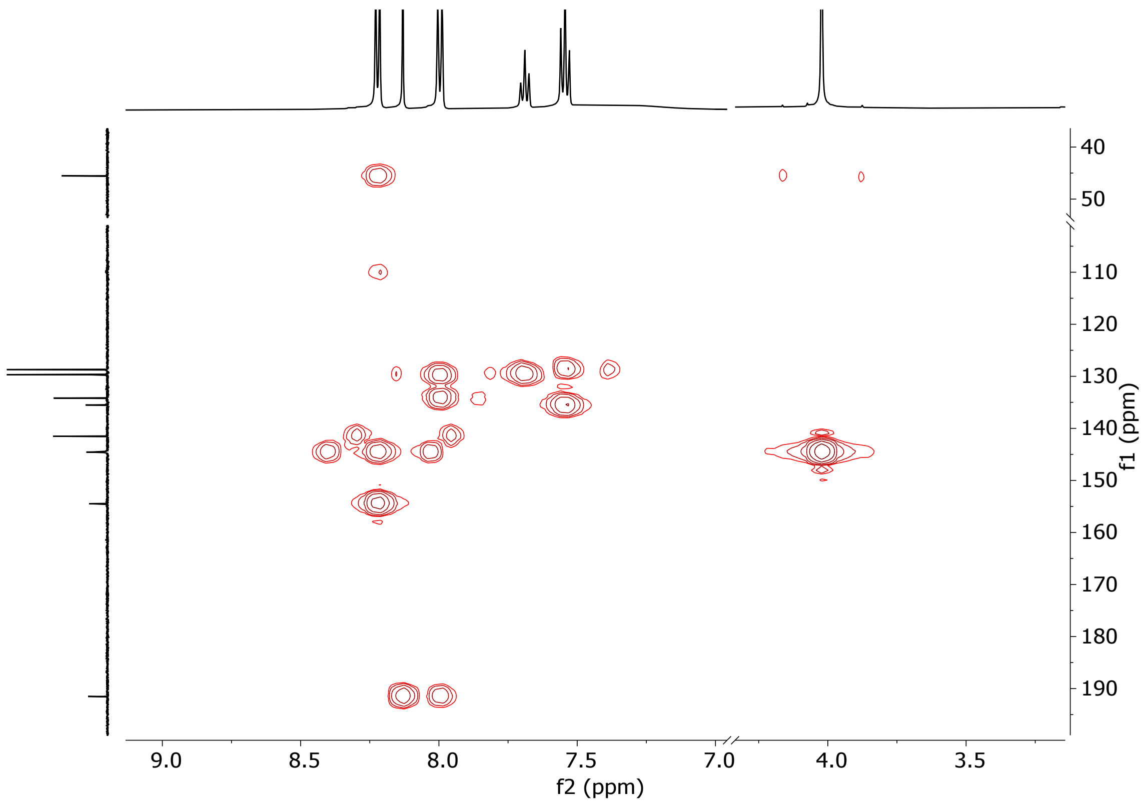

Figure S9. ^{1}H-^{13}C HMBC (500 MHz, D_2O) partial spectrum of the compound $\mathbf{P}_a$H·Cl as synthesized.

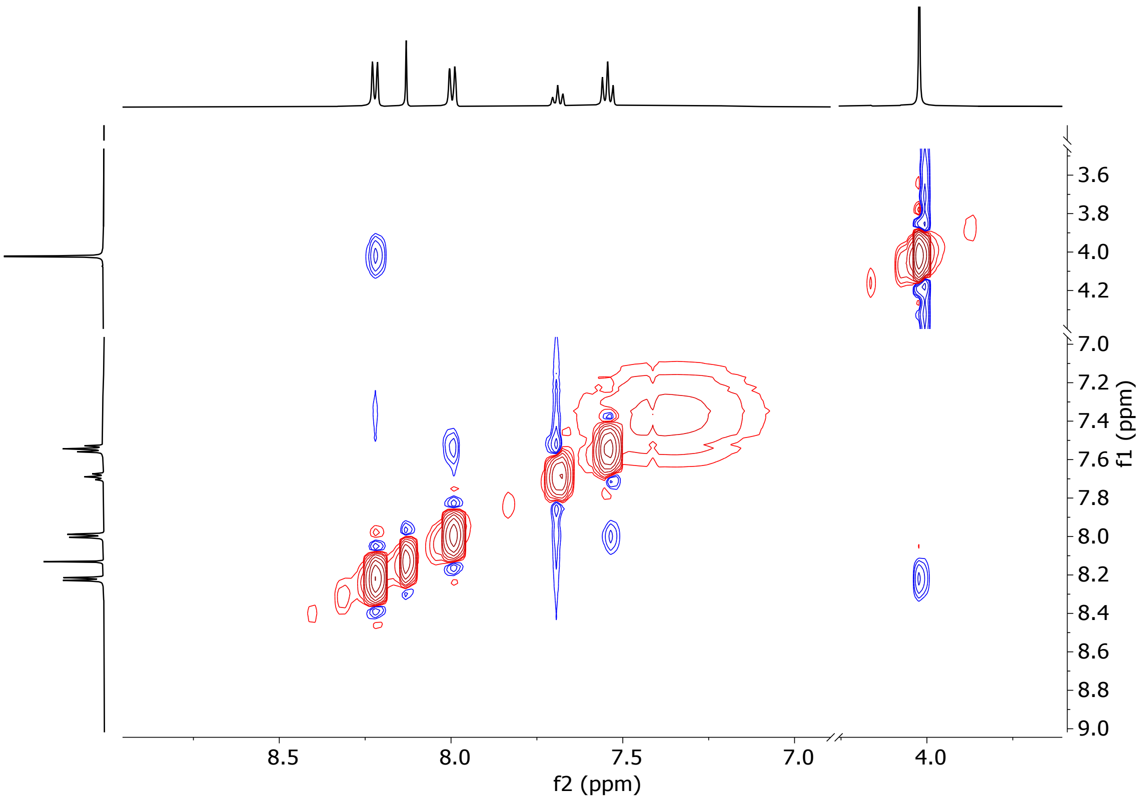

Figure S10. ^{1}H-^{1}H NOESY (500 MHz, D_2O) partial spectrum of the compound $\mathbf{P}_a$H·Cl as synthesized. Blue cross peaks indicate NOE correlations.

2.1.1.2. Organic medium.

P$_a$H·PF$_6$ salt was dissolved at 5 mM in CD$_3$CN.

^{1}**H NMR** (500 MHz, CD$_3$CN) δ (*ppm*): 10.55 (s, 1H), 8.16 (d, J = 7.8 Hz, 2H), 8.08 (d, J = 6.9 Hz, 2H), 7.89 (s, 1H), 7.70 (t, J = 7.5 Hz, 1H), 7.57 (t, J = 7.8 Hz, 2H), 7.51 – 7.12 (m, 2H), 4.02 (s, 3H).

^{13}**C{^{1}H} NMR** (126 MHz, CD$_3$CN) δ (*ppm*): 190.0 (C=O), 155.5 (C$_{Ar}$), 148.0 – 145.2 (m, CH$_{Ar}$), 144.6 (CH=N), 136.9 (C$_{Ar}$), 134.4 (CH$_{Ar}$), 130.8 (CH$_{Ar}$), 129.6 (CH$_{Ar}$), 110.8 (CH$_{Ar}$), 110.8 (CH$_{Ar}$), 46.9 (CH$_3$).

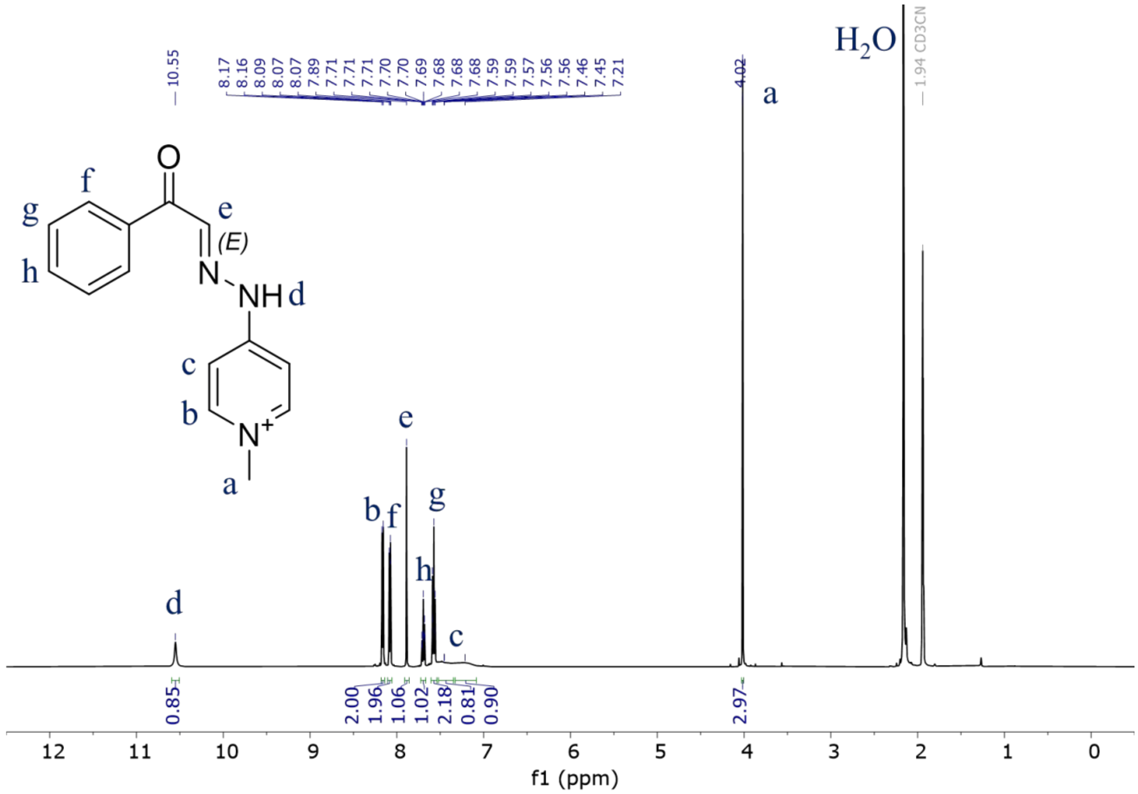

Figure S11. ^{1}H NMR (500 MHz, CD$_3$CN) spectrum of the compound **P**$_a$H·PF$_6$ as synthesized.

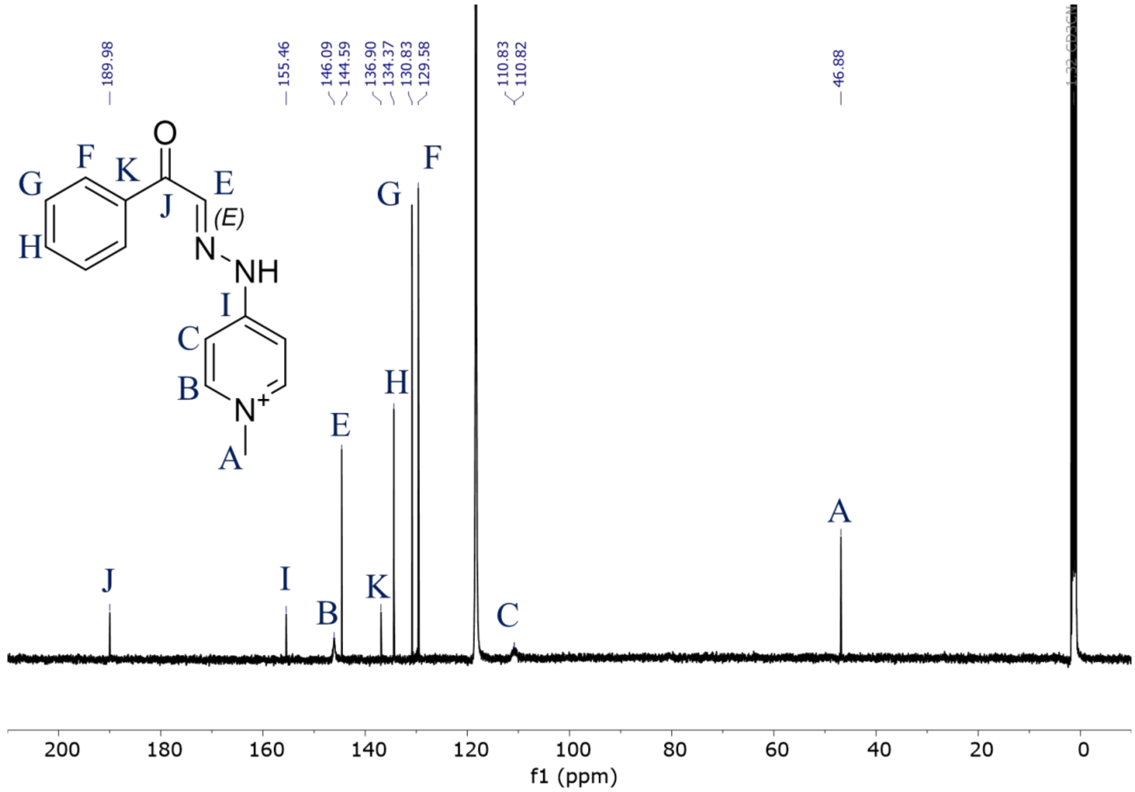

Figure S12. $^{13}C\{^1H\}$ NMR (126 MHz, CD_3CN) spectrum of the compound $\mathbf{P}_a$H·PF_6 as synthesized.

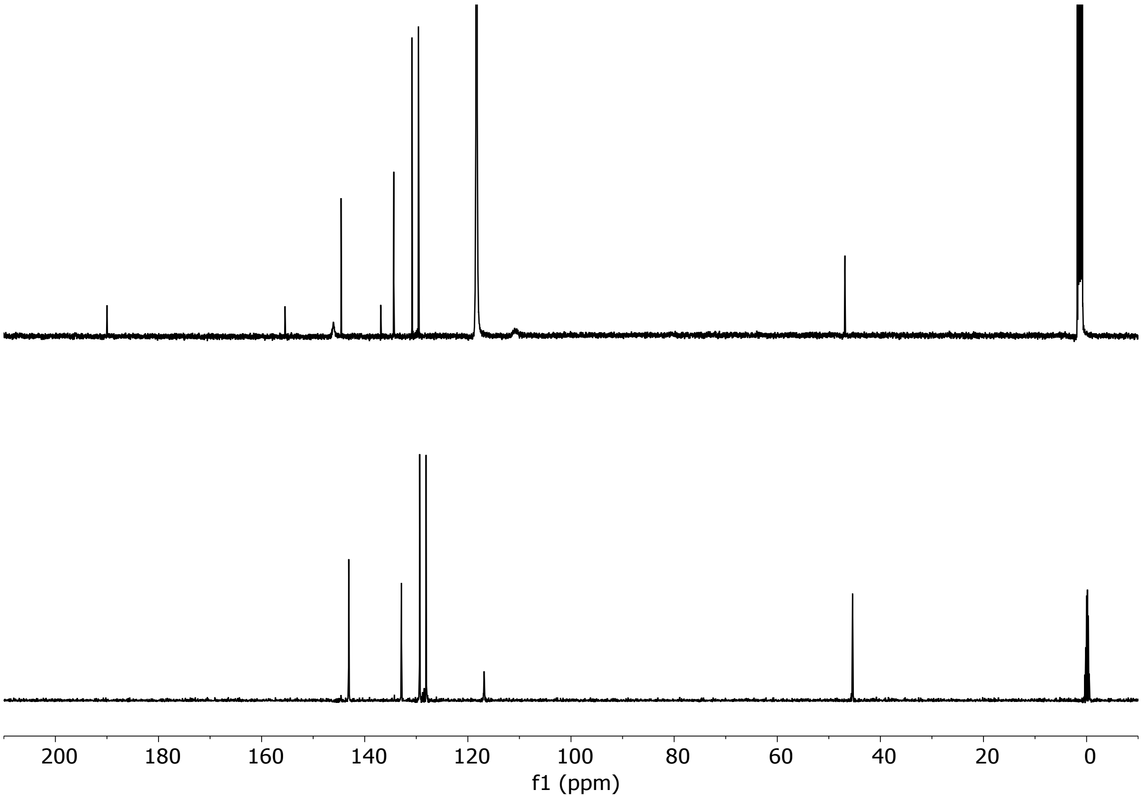

Figure S13. DEPT-135 NMR (126 MHz, CD_3CN) and $^{13}C\{^1H\}$ NMR (126 MHz, CD_3CN) stacked spectra of the compound $\mathbf{P}_a$H·PF_6 as synthesized.

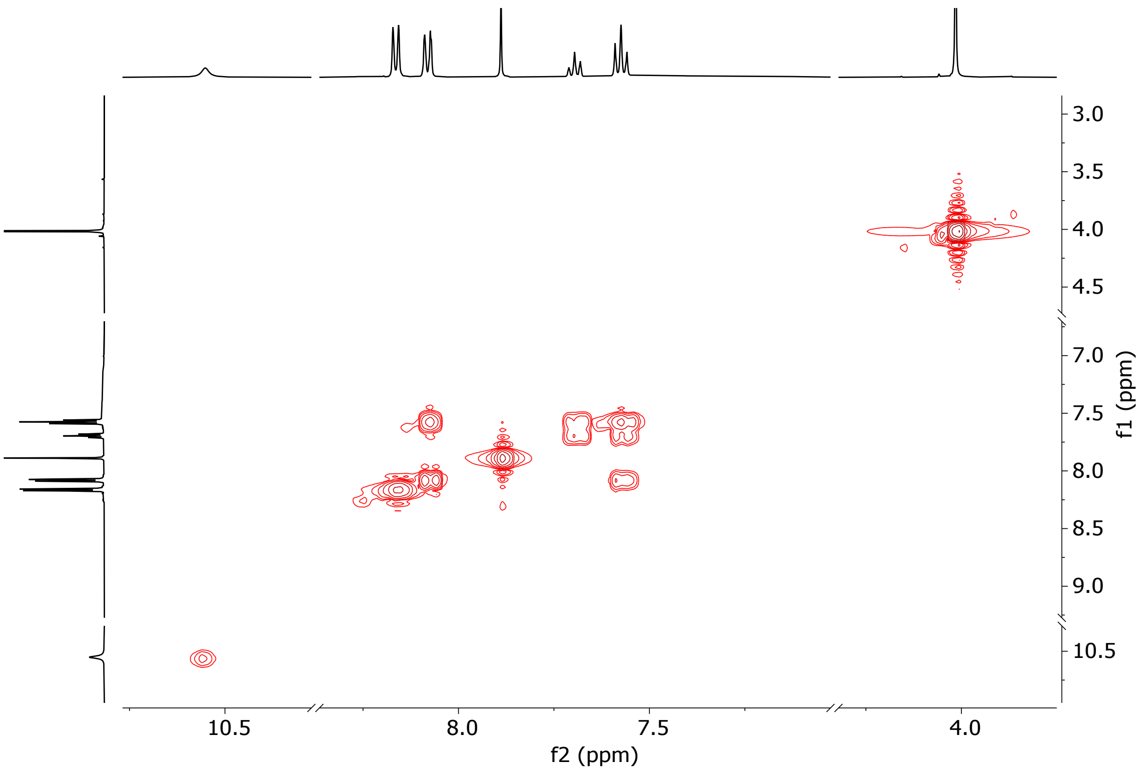

Figure S14. ^{1}H-^{1}H COSY (500 MHz, CD_3CN) partial spectrum of the compound **P**$_a$H·PF_6 as synthesized**.**

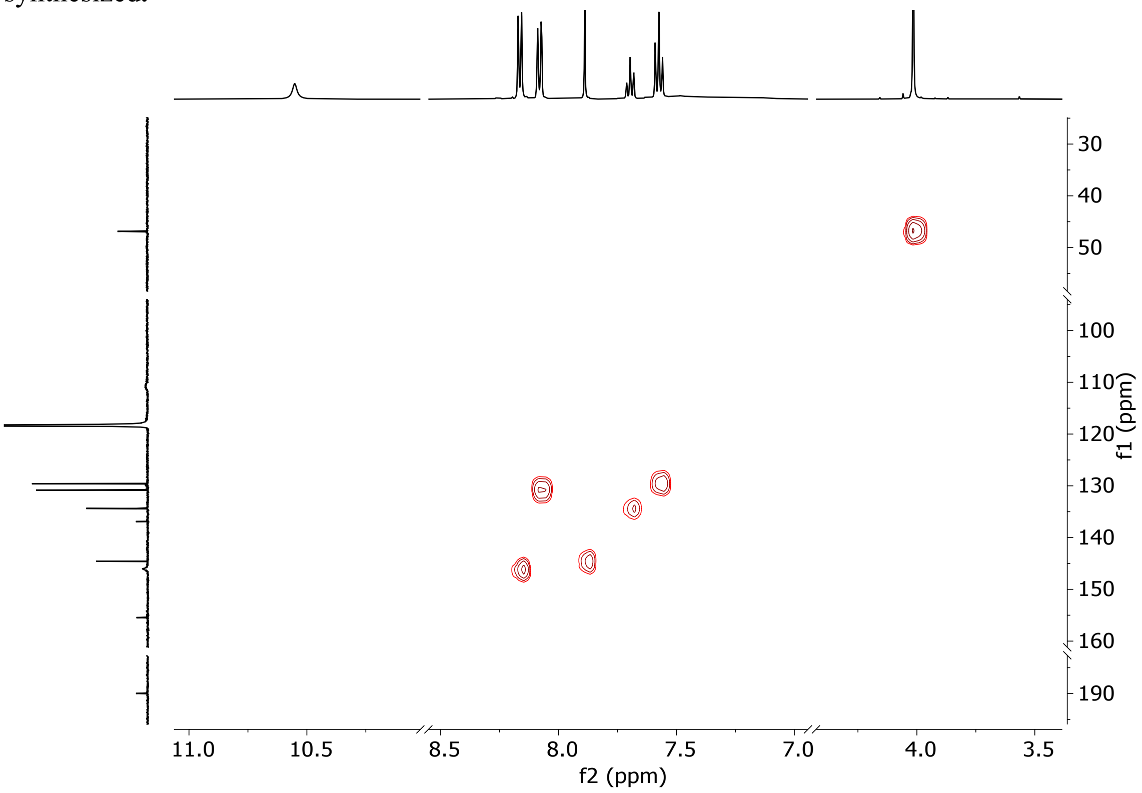

Figure S15. ^{1}H-^{13}C HSQC (500 MHz, CD_3CN) partial spectrum of the compound **P**$_a$H·PF_6 as synthesized.

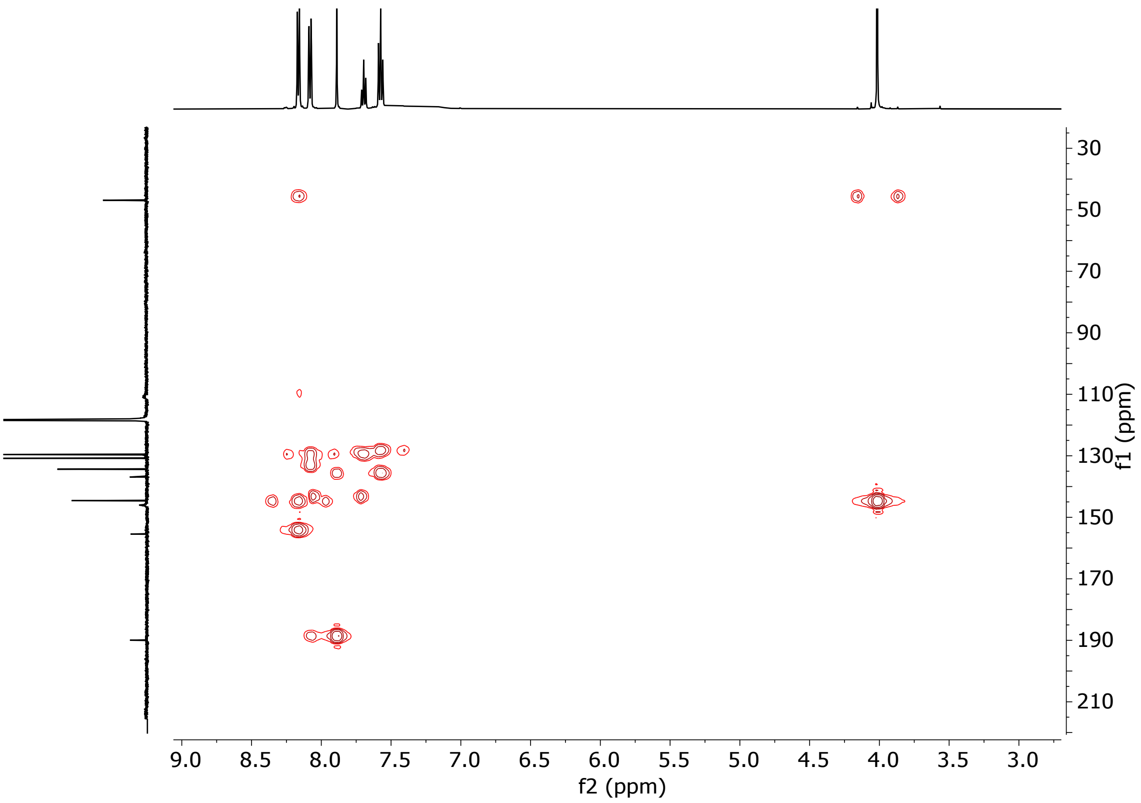

Figure S16. ^{1}H-^{13}C HMBC (500 MHz, CD_3CN) spectrum of the compound $\mathbf{P}_aH \cdot PF_6$ as synthesized.

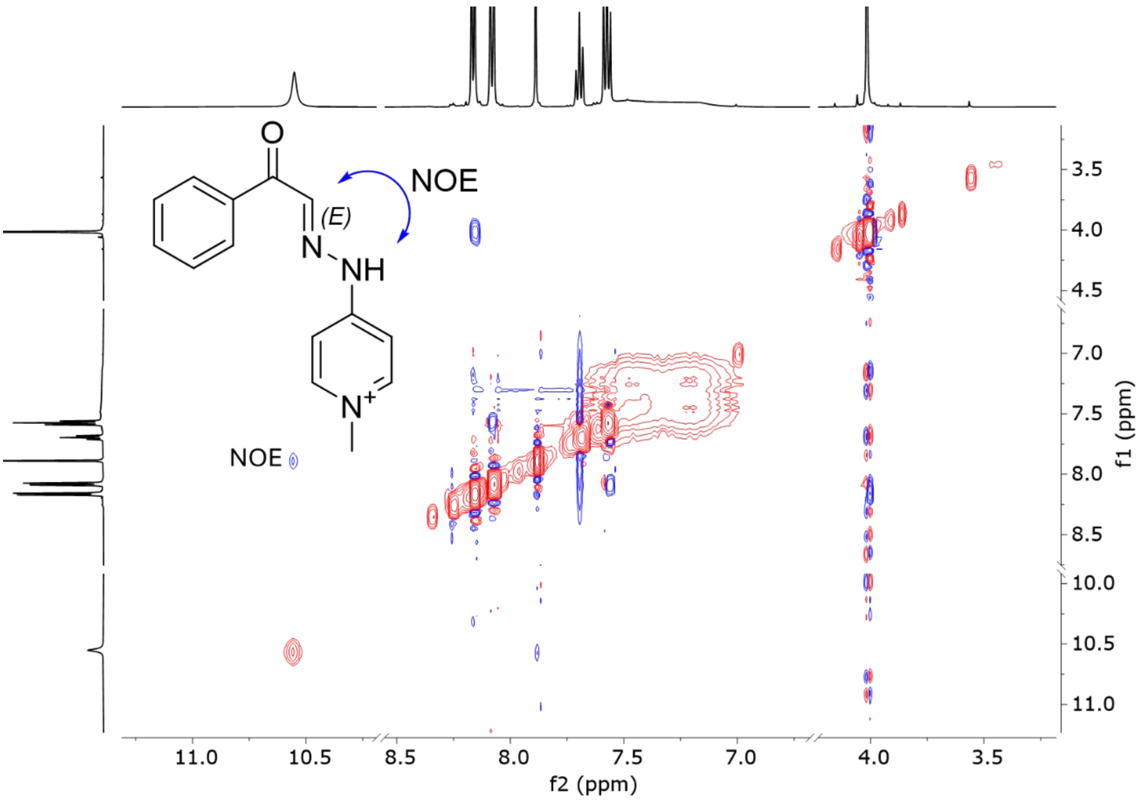

Figure S17. ^{1}H-^{1}H NOESY (500 MHz, CD_3CN) partial spectrum of the compound $\mathbf{P}_aH \cdot PF_6$ as synthesized. Blue cross peaks indicate NOE correlations. The observed NOE between the NH and the imine proton is diagnostic of the *E* isomer.

2.1.2. NMR data for the species assigned as *Z*-P_aH^+.

2.1.2.1. Aqueous medium.

P_aH·Cl salt was dissolved at 5 mM in a D_2O phosphate buffer solution (20 mM, pD 6). The NMR tube containing the compound solution was irradiated for 16 h at 254 nm, after which all NMR experiments were acquired.

^{1}H NMR (500 MHz, D_2O) δ (*ppm*): 8.39 (d, J = 7.1 Hz, 2H), 8.21 (s, 1H), 8.13 (d, J = 7.4 Hz, 2H), 7.77 (t, J = 7.5 Hz, 1H), 7.73 – 7.66 (m, 1H), 7.63 (t, J = 7.8 Hz, 2H), 4.14 (s, 3H).

^{13}C{^{1}H} NMR (126 MHz, D_2O) δ (*ppm*): 188.5 (C=O), 154.6 (C_{Ar}), 144.9 (CH_{Ar}), 135.8 (C_{Ar}), 134.8 (CH_{Ar}), 132.2 (CH=N), 129.2 (CH_{Ar}), 128.9 (CH_{Ar}), 110.7 (CH_{Ar}), 45.8 (CH_3).

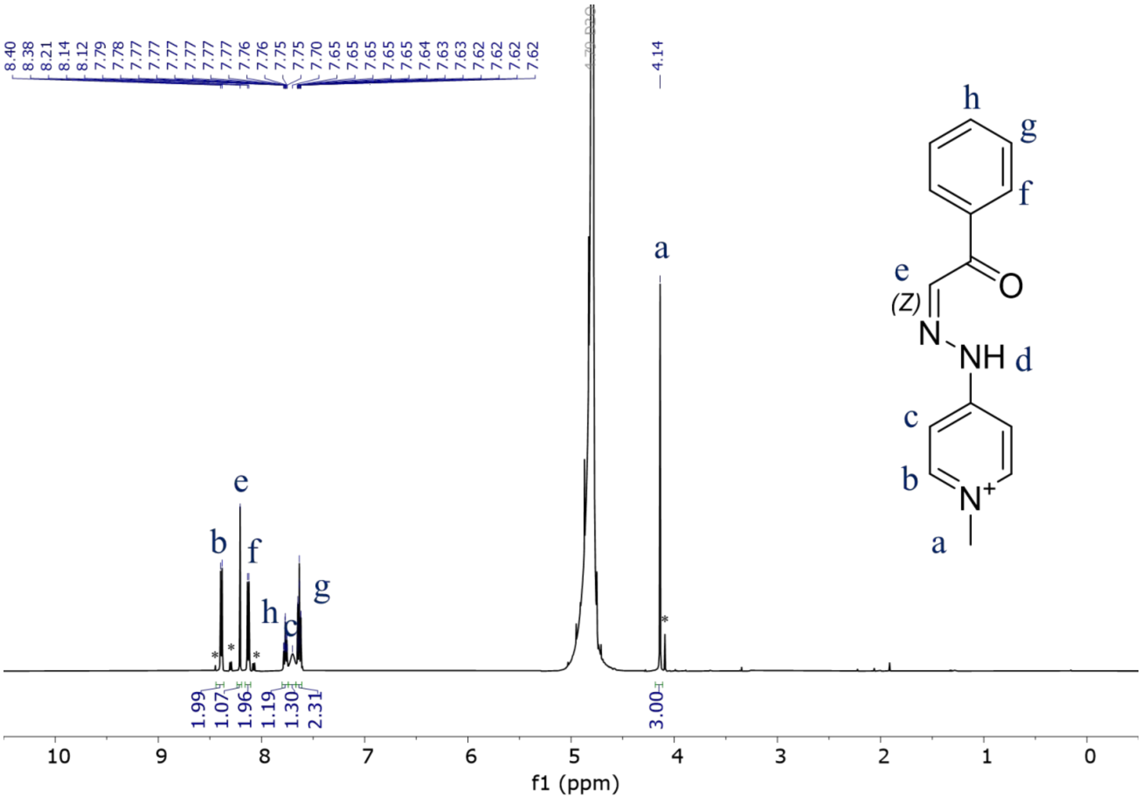

Figure S18. ^{1}H NMR (500 MHz, D_2O) spectrum of the 254 nm PSS for the compound **P_a**H·Cl. Signals marked with an asterisk correspond to the *E*-isomer.

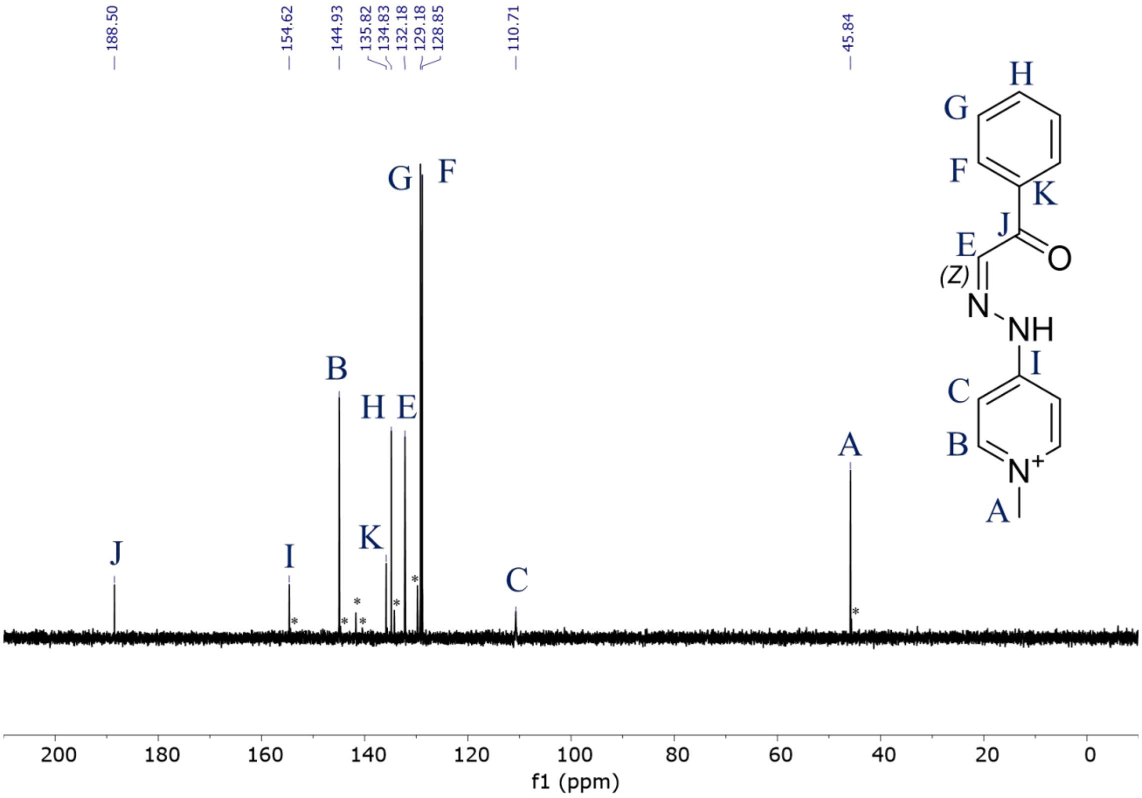

Figure S19. $^{13}C\{^1H\}$ NMR (126 MHz, D_2O) spectrum of the 254 nm PSS for the compound $\mathbf{P}_a H{\cdot}Cl$. Signals marked with an asterisk correspond to the *E*-isomer.

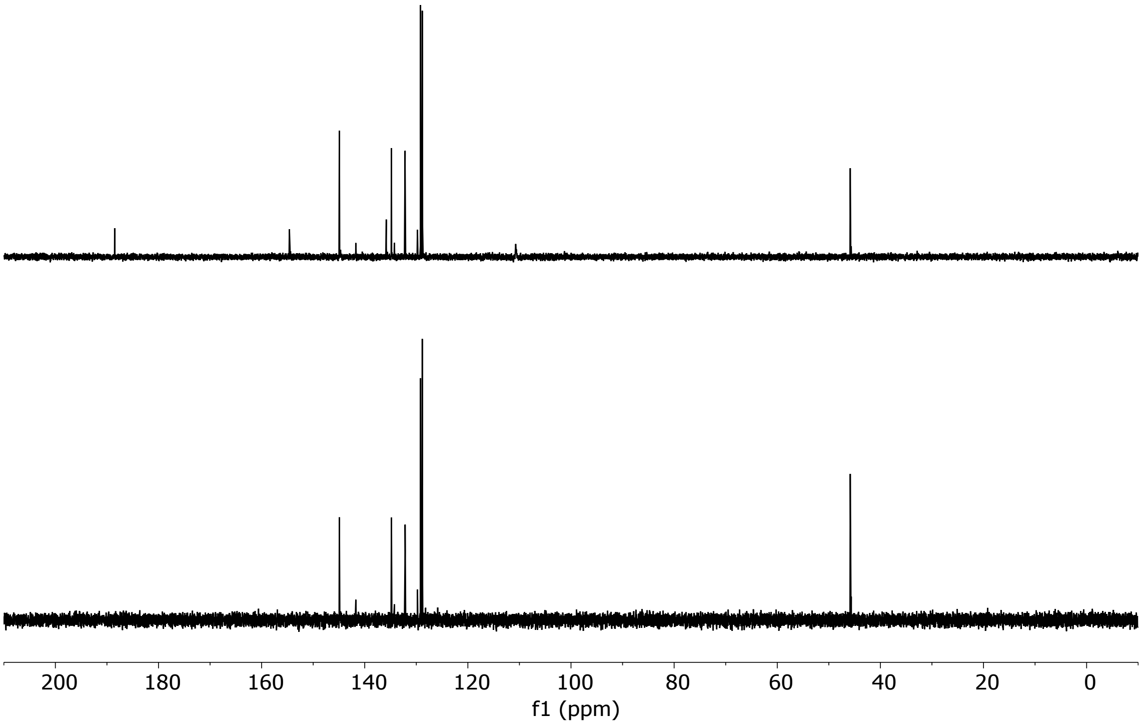

Figure S20. DEPT-135 NMR (126 MHz, D_2O) and $^{13}C\{^1H\}$ NMR (126 MHz, D_2O) stacked spectra of the 254 nm PSS for the compound $\mathbf{P}_a H{\cdot}Cl$.

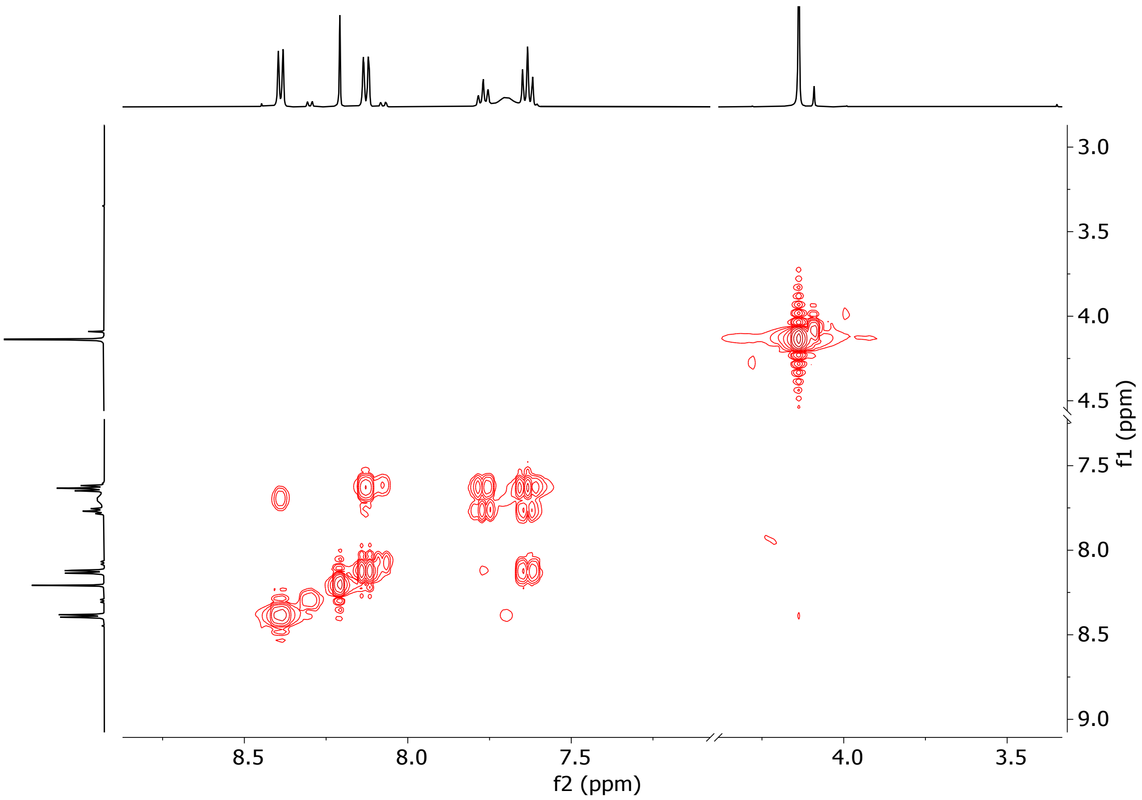

Figure S21. ^{1}H-^{1}H COSY (500 MHz, D_2O) partial spectrum of the 254 nm PSS for the compound **P**$_a$H·Cl.

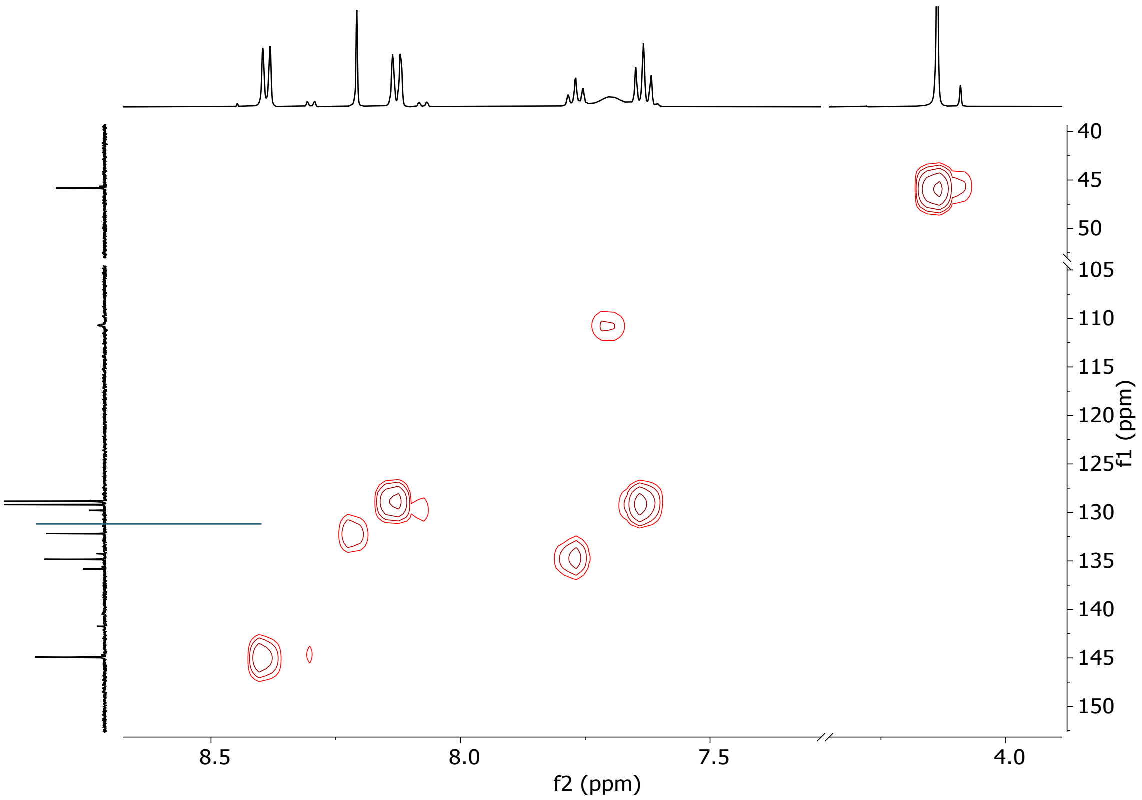

Figure S22. ^{1}H-^{13}C HSQC (500 MHz, D_2O) partial spectrum of the 254 nm PSS for the compound **P**$_a$H·Cl.

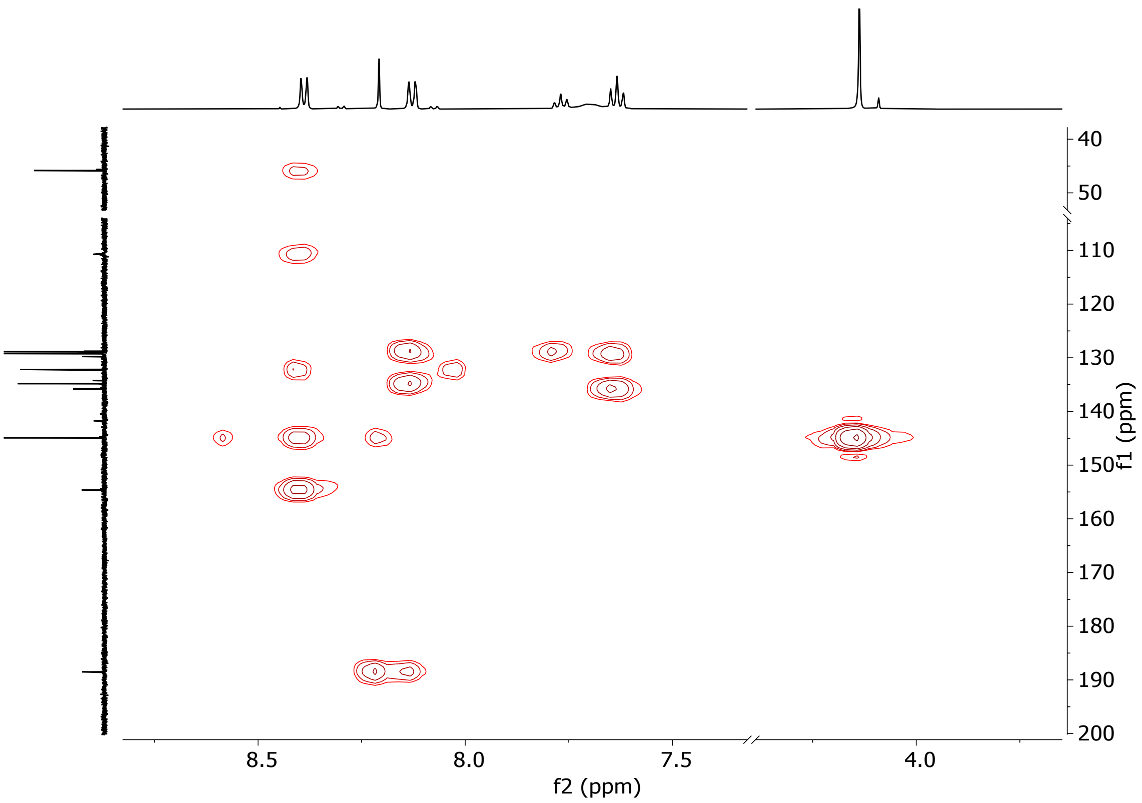

Figure S23. ^{1}H-^{13}C HMBC (500 MHz, D_2O) partial spectrum of the 254 nm PSS for the compound $\mathbf{P}_a$H·Cl.

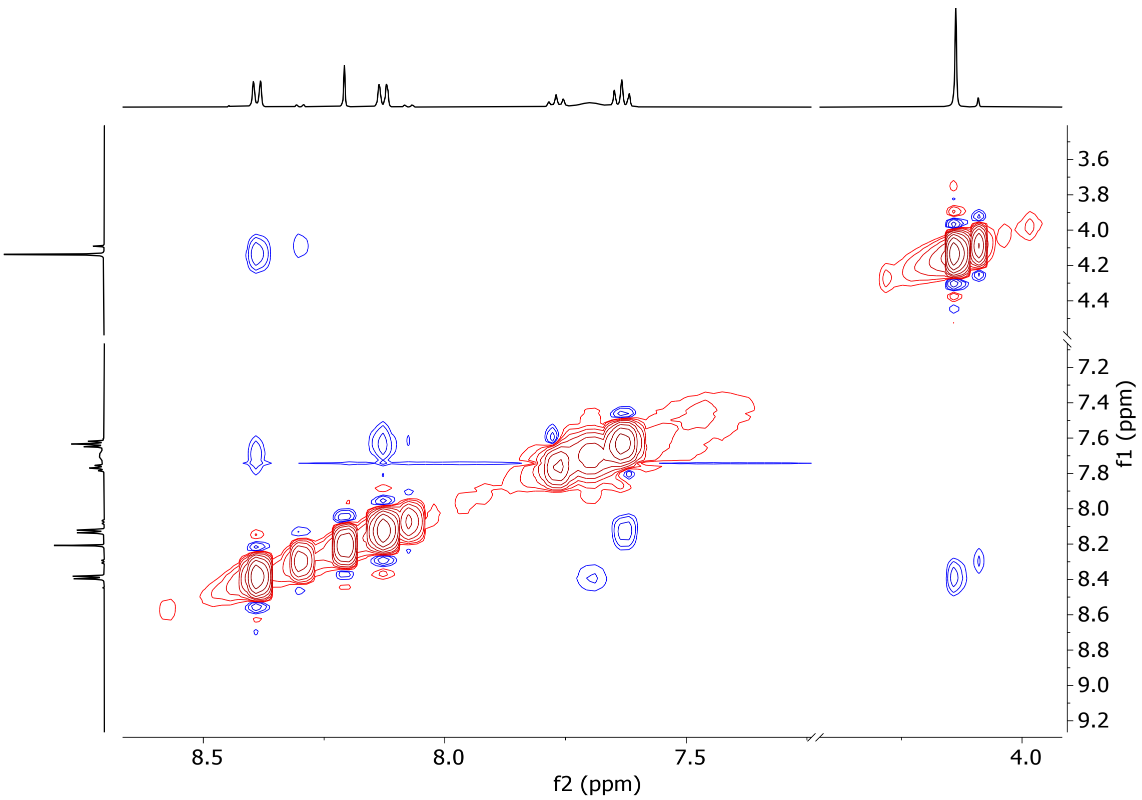

Figure S24. ^{1}H-^{1}H NOESY (500 MHz, D_2O) partial spectrum of the 254 nm PSS for the compound $\mathbf{P}_a$H·Cl. Blue cross peaks indicate NOE correlations.

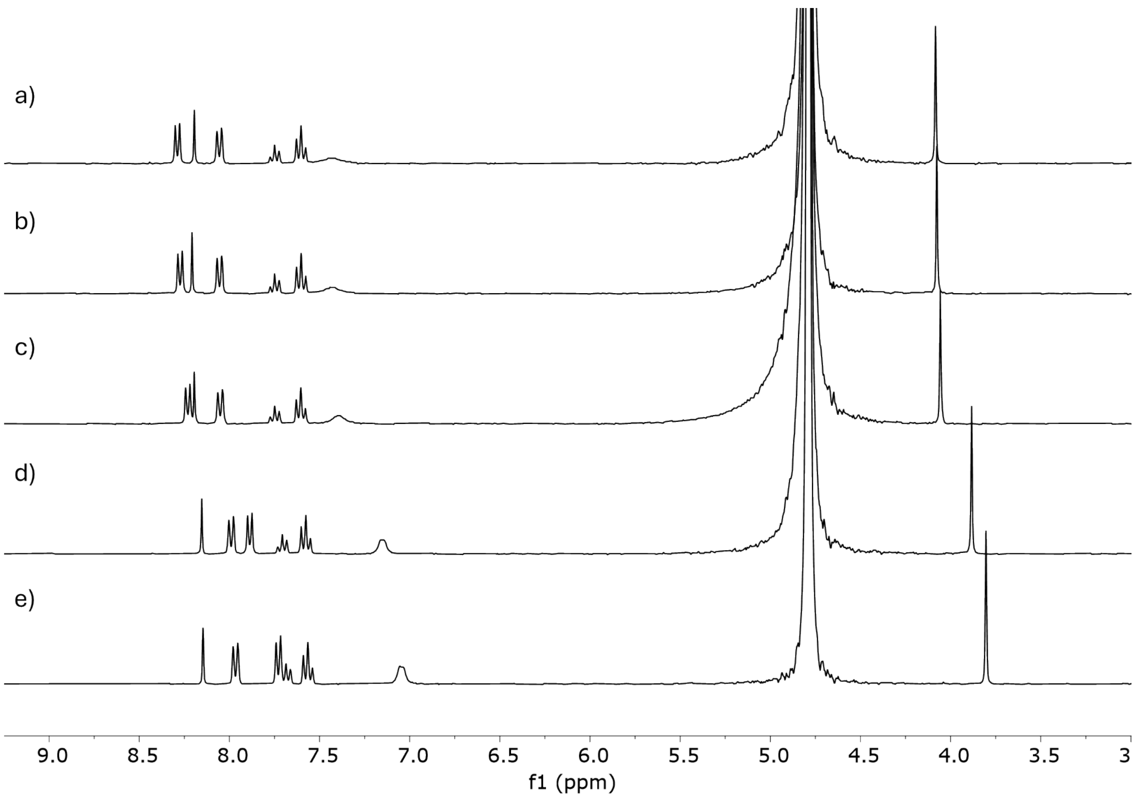

Figure S25. Partial stacked ¹H NMR spectra (300 MHz, D_2O) of $\mathbf{P}_a$H·Cl (5 mM) as a function of pD: a) pD 6.5, b) pD 7.5, c) pD 8.5, d) pD 9.8, e) pD 11.4.

2.1.2.2. Organic medium.

$\mathbf{P_a}H\cdot PF_6$ salt was dissolved at 5 mM in CD_3CN. The NMR tube containing the compound solution was irradiated for 16 h at 254 nm, after which all NMR experiments were acquired.

^{1}H NMR (500 MHz, CD_3CN) δ (*ppm*): 13.94 (s, 1H), 8.26 (d, *J* = 7.1 Hz, 2H), 8.19 (s, 1H), 8 8.14 (d, *J* = 7.1 Hz, 2H), 7.75 (t, *J* = 7.5 Hz, 1H), 7.75 – 7.69 (m, 2H), 7.62 (t, *J* = 7.8 Hz, 2H), 4.06 (s, 3H).

^{13}C{^{1}H} NMR (126 MHz, CD_3CN) δ (*ppm*): 187.3 (C=O), 154.6 (C_{Ar}), 145.0 (CH_{Ar}), 135.7 (C_{Ar}), 134.4 (CH_{Ar}), 131.8 (CH=N), 128.9 (CH_{Ar}), 128.6 (CH_{Ar}), 110.4 (CH_{Ar}), 45.8 (CH_3).

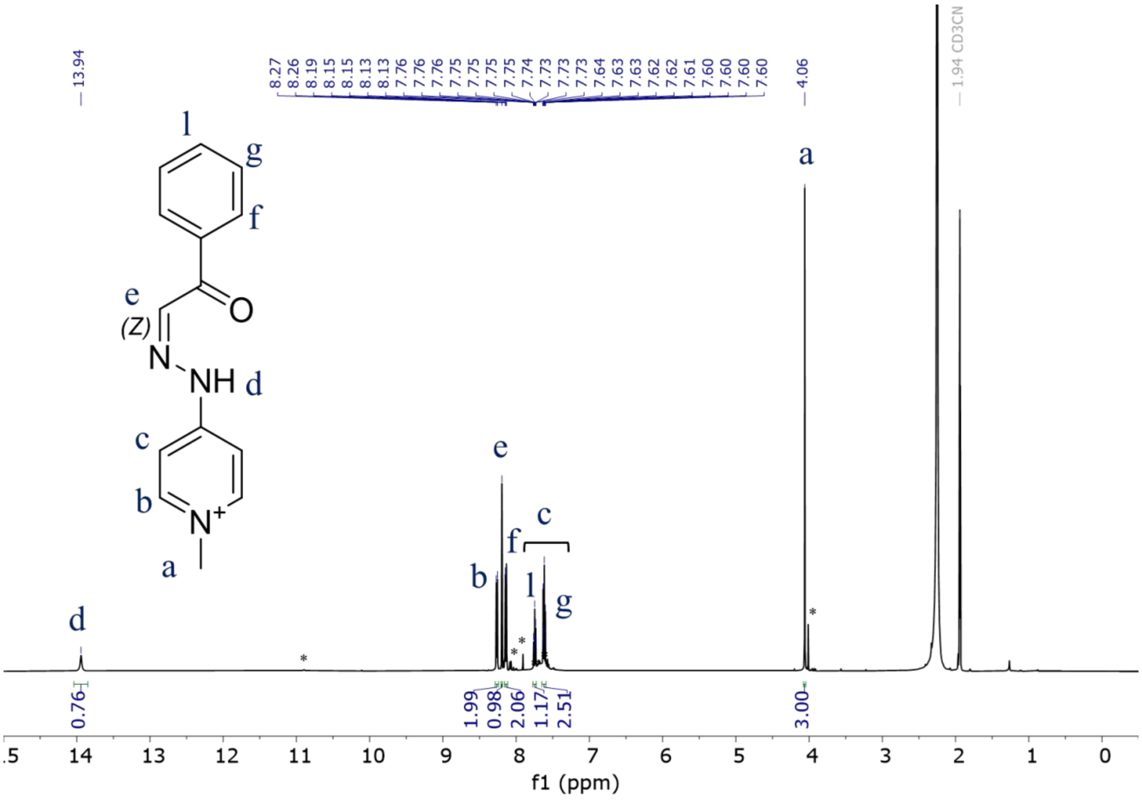

Figure S26. ^{1}H NMR (500 MHz, CD_3CN) spectrum of the 254 nm PSS for the compound $\mathbf{P_a}H\cdot PF_6$. Signals marked with an asterisk correspond to the *E*-isomer.

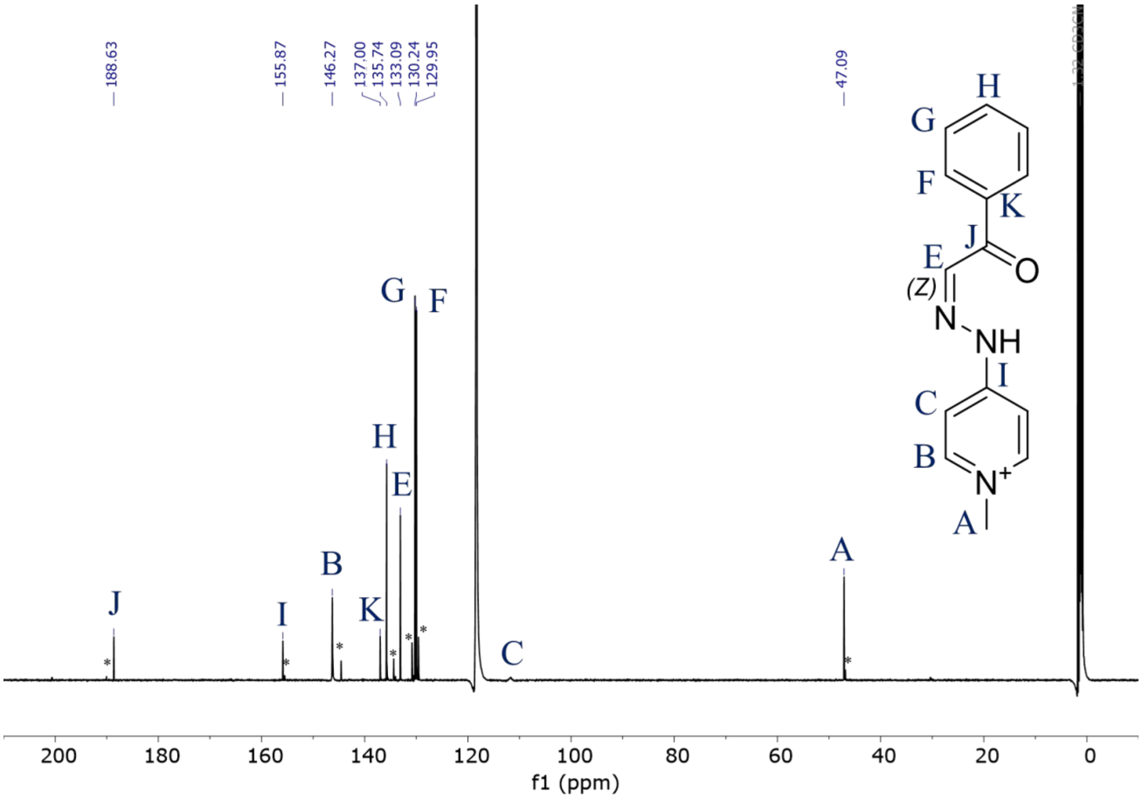

Figure S27. $^{13}C\{^{1}H\}$ NMR (126 MHz, CD_3CN) spectrum of the 254 nm PSS for the compound $\mathbf{P}_a H \cdot PF_6$**.** Signals marked with an asterisk correspond to the *E*-isomer.

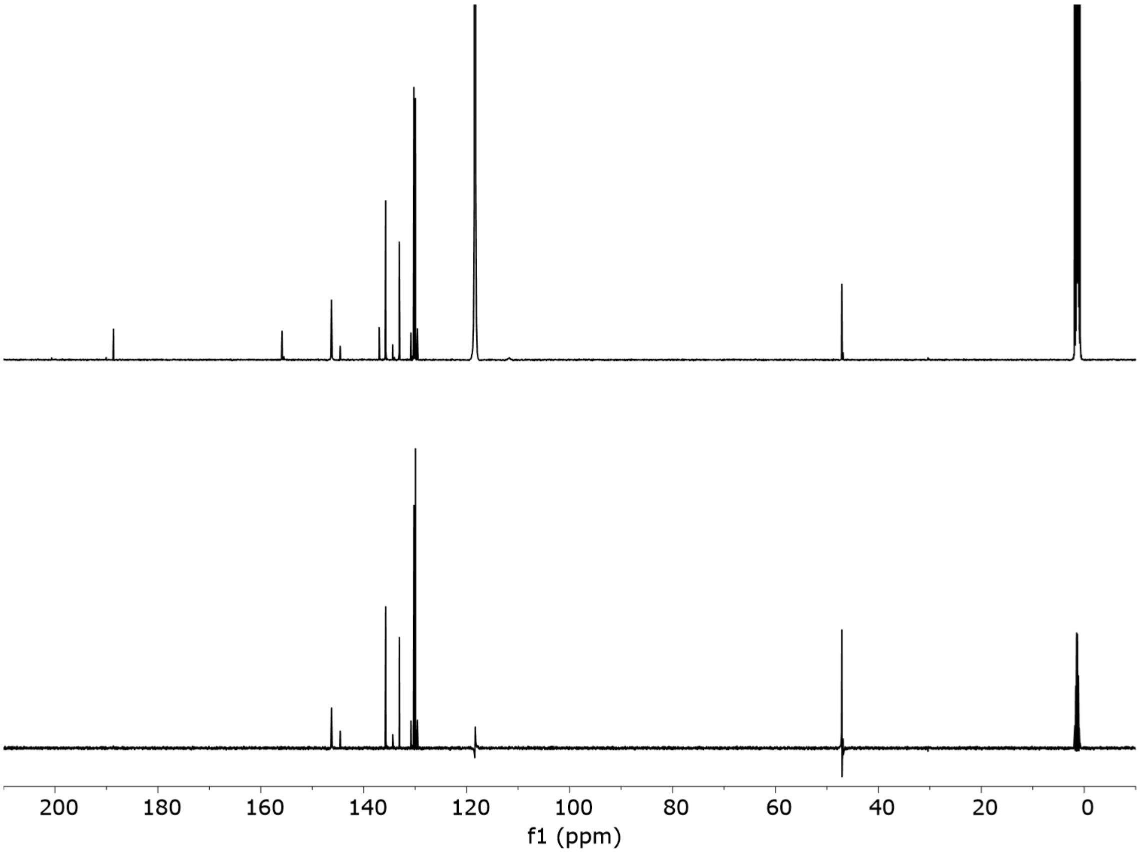

Figure S28. DEPT-135 NMR (126 MHz, CD_3CN) and $^{13}C\{^{1}H\}$ NMR (126 MHz, CD_3CN) stacked spectra of the 254 nm PSS for the compound $\mathbf{P}_a H \cdot PF_6$.

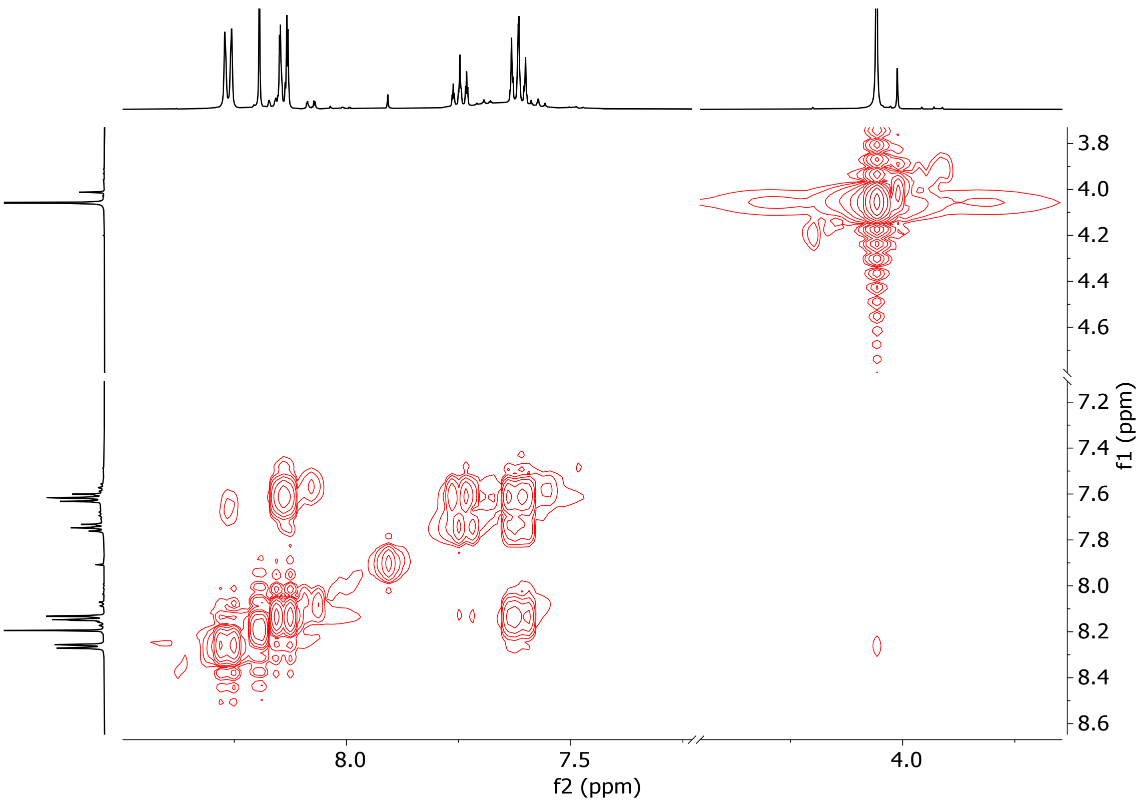

Figure S29. ^{1}H-^{1}H COSY (500 MHz, CD_3CN) partial spectrum of the 254 nm PSS for the compound $\mathbf{P}_a H \cdot PF_6$.

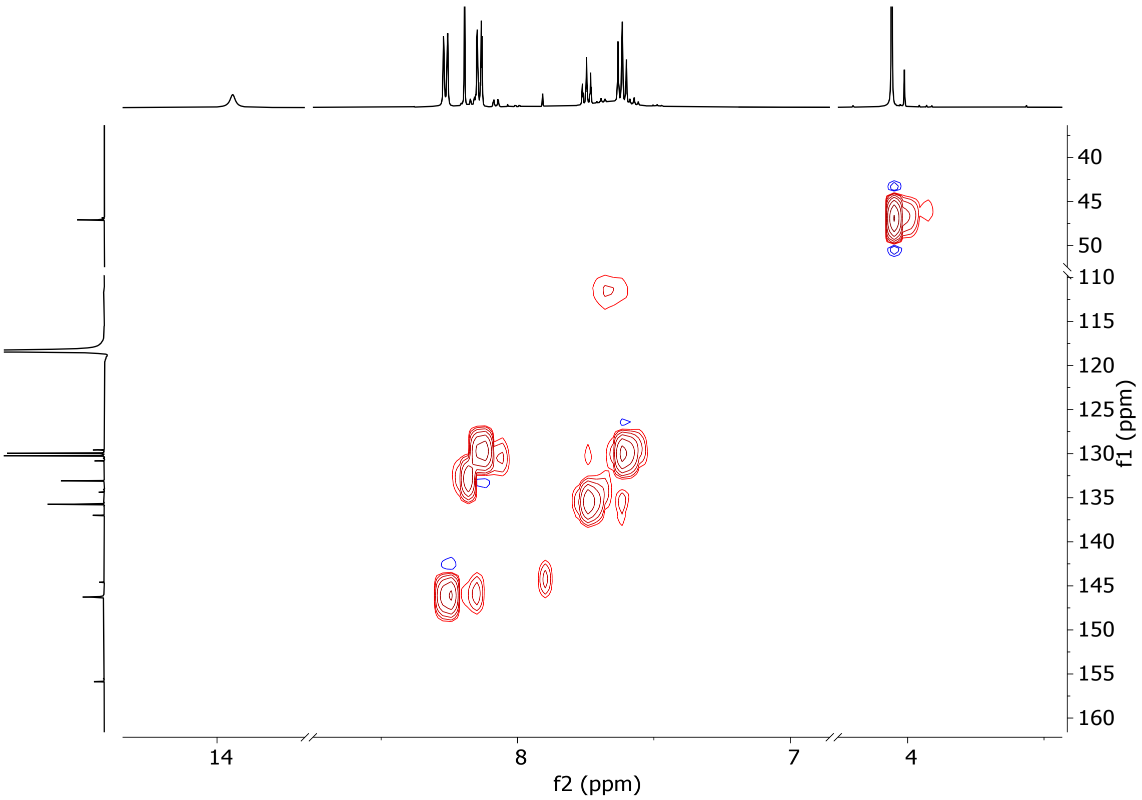

Figure S30. ^{1}H-^{13}C HSQC (500 MHz, CD_3CN) partial spectrum of the 254 nm PSS for the compound $\mathbf{P}_a H \cdot PF_6$.

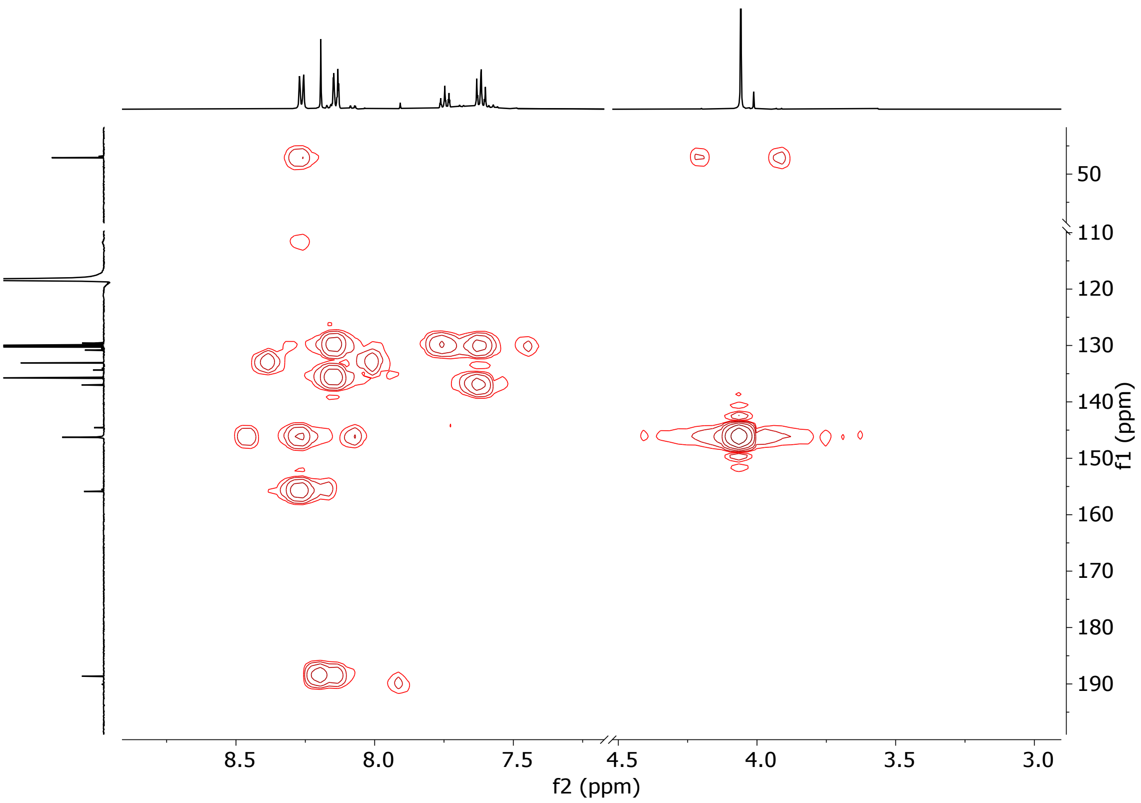

Figure S31. ^{1}H-^{13}C HMBC (500 MHz, CD_3CN) partial spectrum of the 254 nm PSS for the compound $\mathbf{P}_a$H·PF_6.

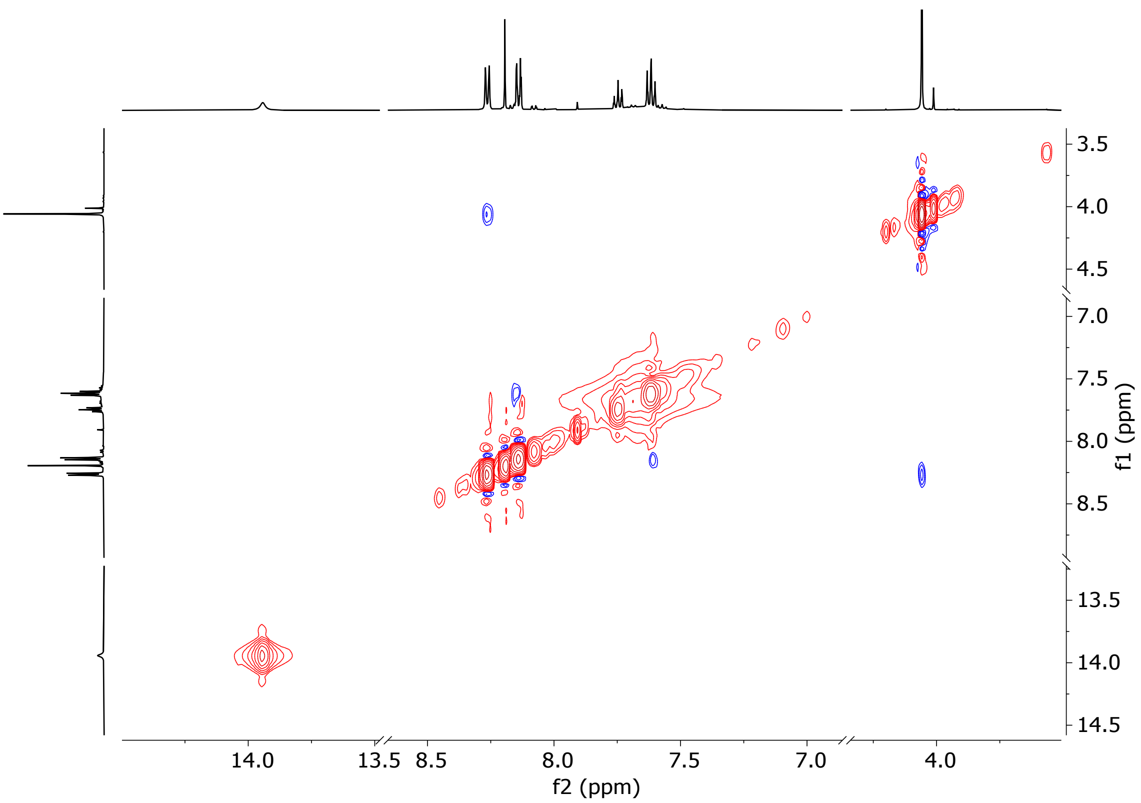

Figure S32. ^{1}H-^{1}H NOESY (500 MHz, CD_3CN) partial spectrum of the 254 nm PSS for the compound $\mathbf{P}_a$H·PF_6. Blue cross peaks indicate NOE correlations. The absence of NOE coupling between NH and the imine signals is diagnostic of the *Z* isomer.

2.1.3. NMR data for the species assigned as *E*-P_a.

P_aH·Cl salt was dissolved at 5 mM in a D_2O phosphate buffer solution (20 mM, pD 11).

^{1}H NMR (500 MHz, D_2O) δ (*ppm*): 8.12 (s, 1H), 7.93 (d, J = 7.1 Hz, 2H), 7.72 (d, J = 7.2 Hz, 2H), 7.64 (t, J = 7.5 Hz, 1H), 7.52 (t, J = 7.8 Hz, 2H), 7.13 – 6.89 (m, 2H), 3.78 (s, 3H).

^{13}C{^{1}H} NMR (126 MHz, D_2O) δ (*ppm*): 193.6 (C=O), 164.6 (C_{Ar}), 142.1 (CH_{Ar}), 141.1 (CH=N), 137.2 (C_{Ar}), 133.1 (CH_{Ar}), 129.4 (CH_{Ar}), 128.4 (CH_{Ar}), 111.7 (CH_{Ar}), 44.0 (CH_3).

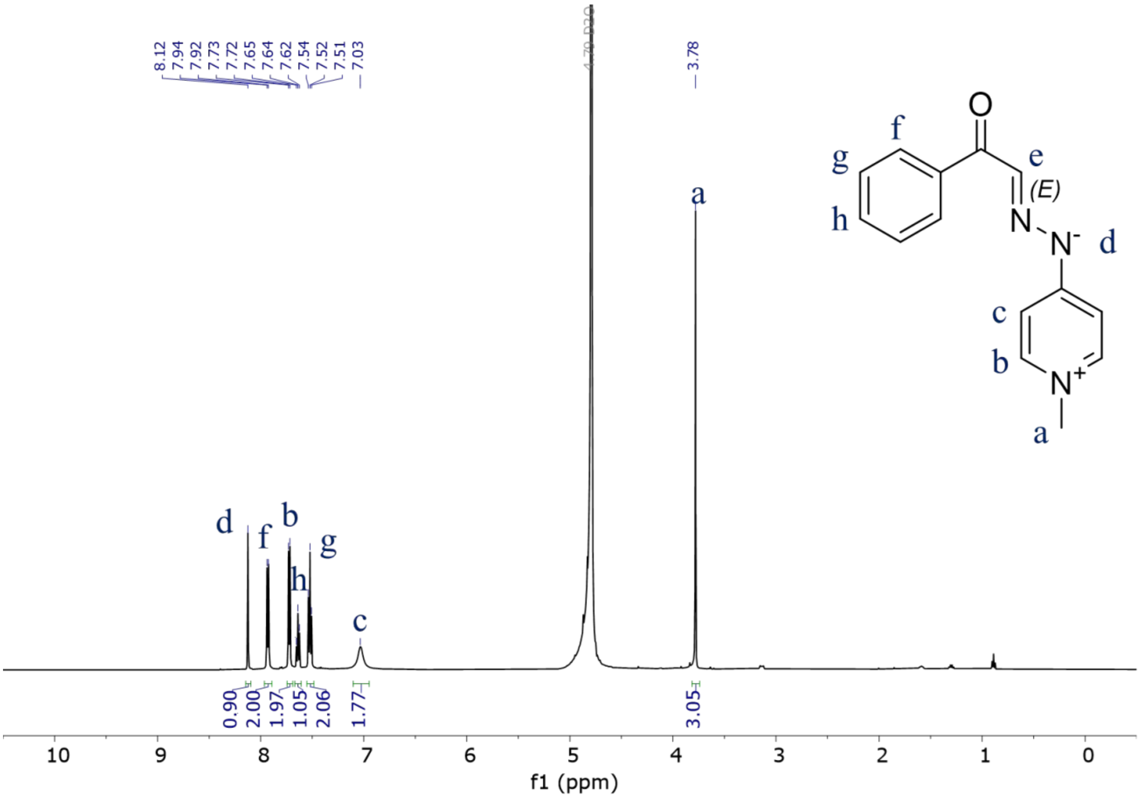

Figure S33. ^{1}H NMR (500 MHz, D_2O) spectrum of the compound P_aH·Cl as synthesized.

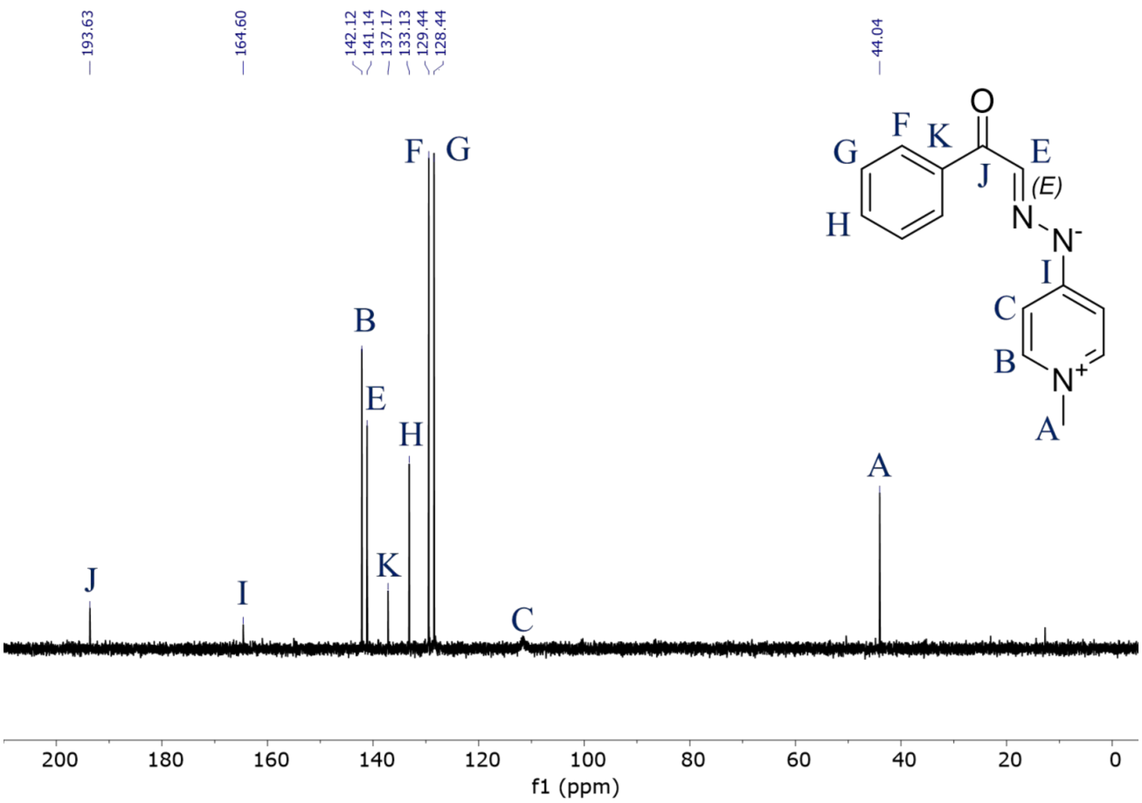

Figure S34. $^{13}C\{^{1}H\}$ NMR (126 MHz, D_2O) spectrum of the compound $\mathbf{P}_a$H·Cl as synthesized.

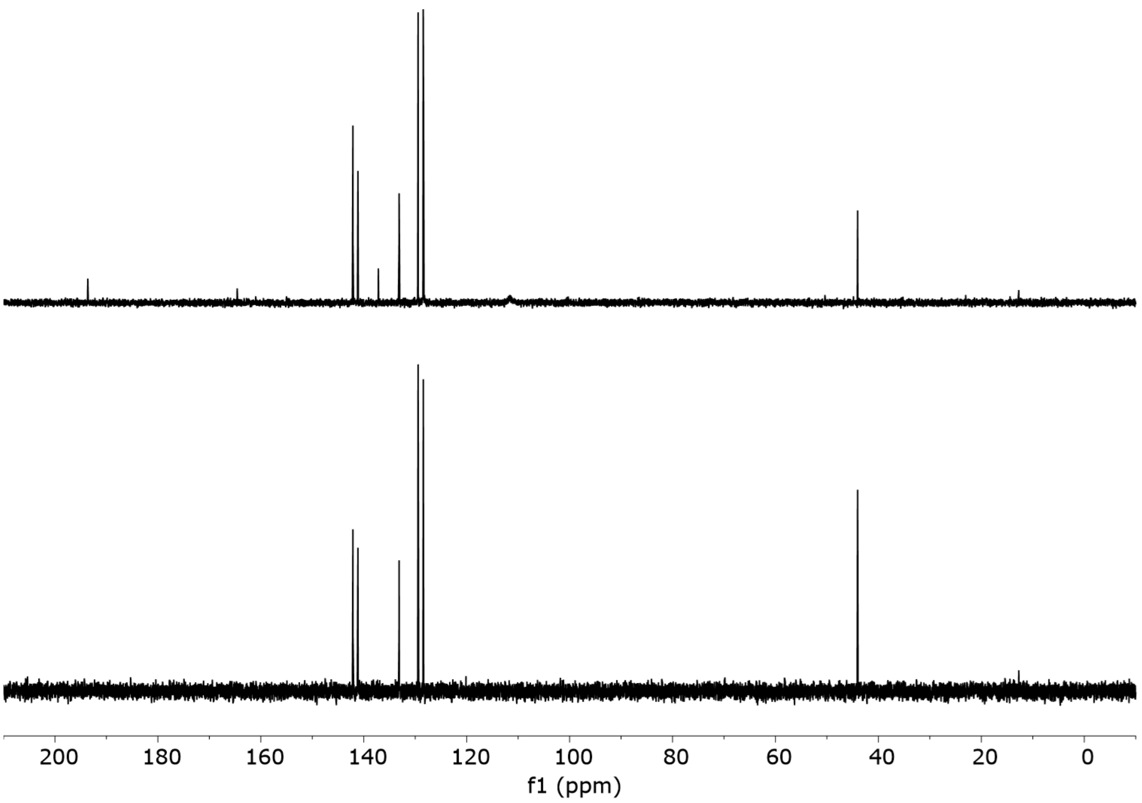

Figure S35. DEPT-135 NMR (126 MHz, D_2O) and $^{13}C\{1H\}$ NMR (126 MHz, D_2O) stacked spectra of the compound $\mathbf{P}_a$H·Cl as synthesized.

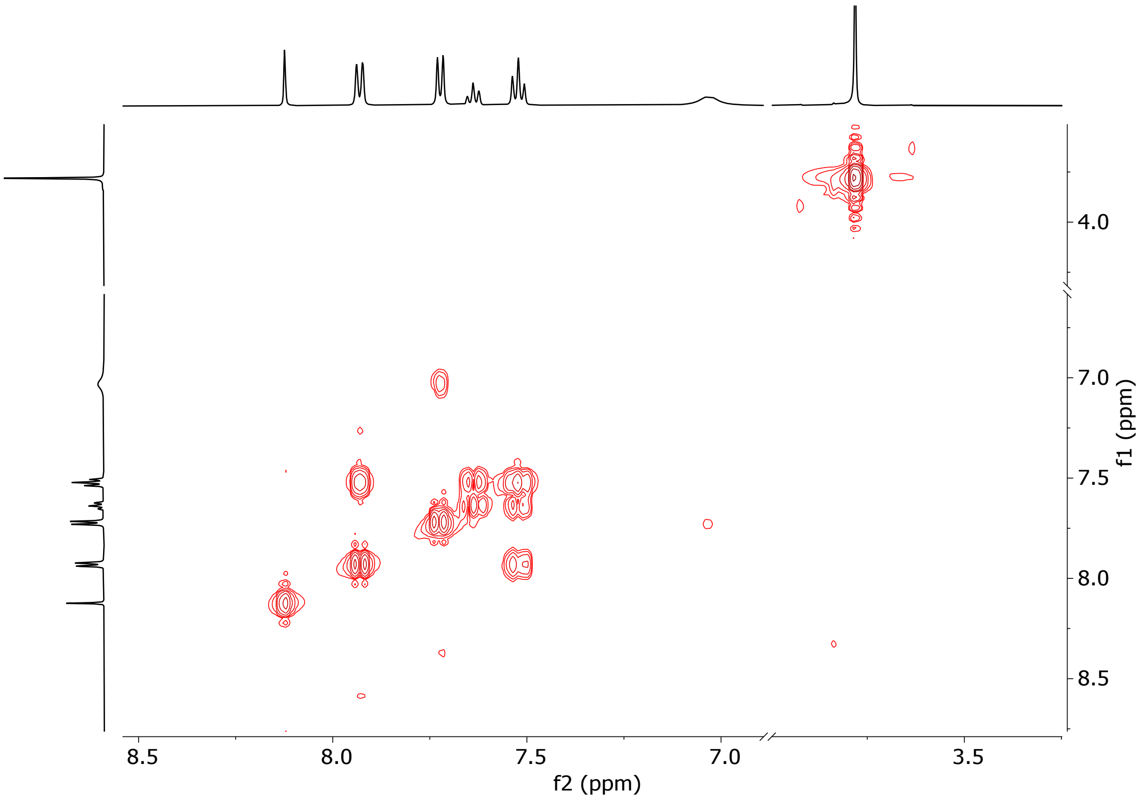

Figure S36. ^{1}H-^{1}H COSY (500 MHz, D_2O) partial spectrum of the compound $\mathbf{P}_a$H·Cl as synthesized.

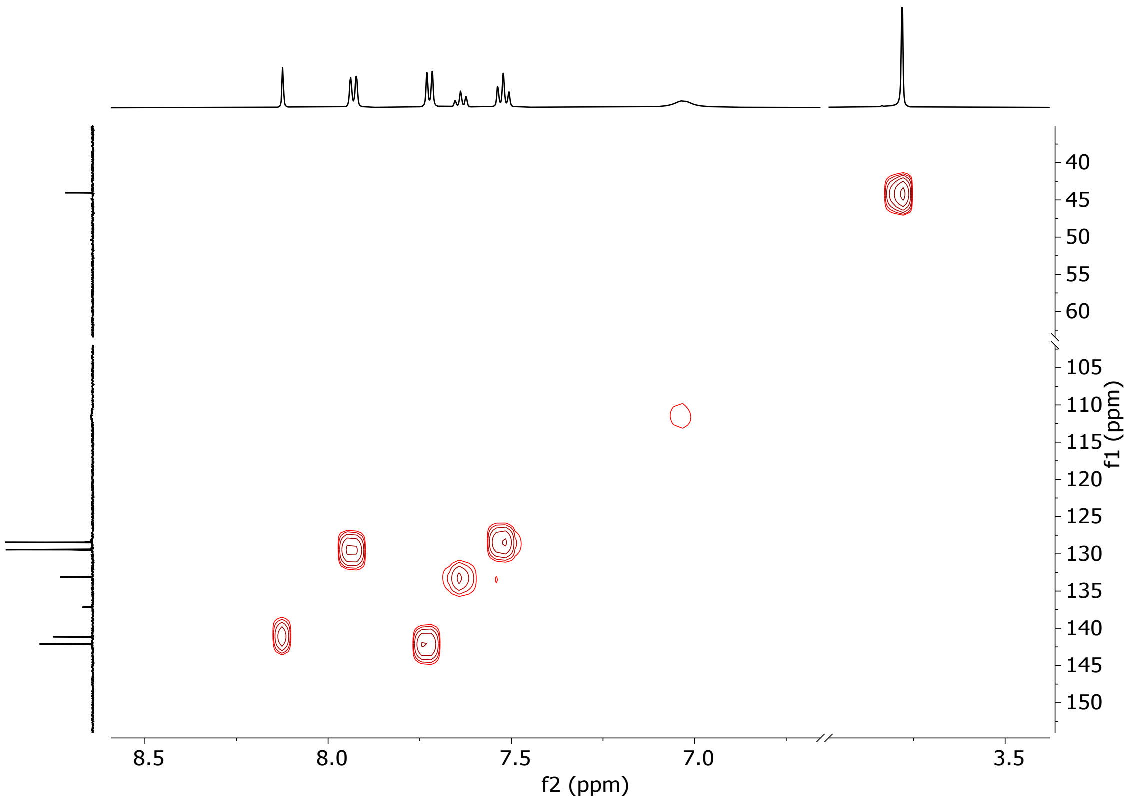

Figure S37. ^{1}H-^{13}C HSQC (500 MHz, D_2O) partial spectrum of the compound $\mathbf{P}_a$H·Cl as synthesized.

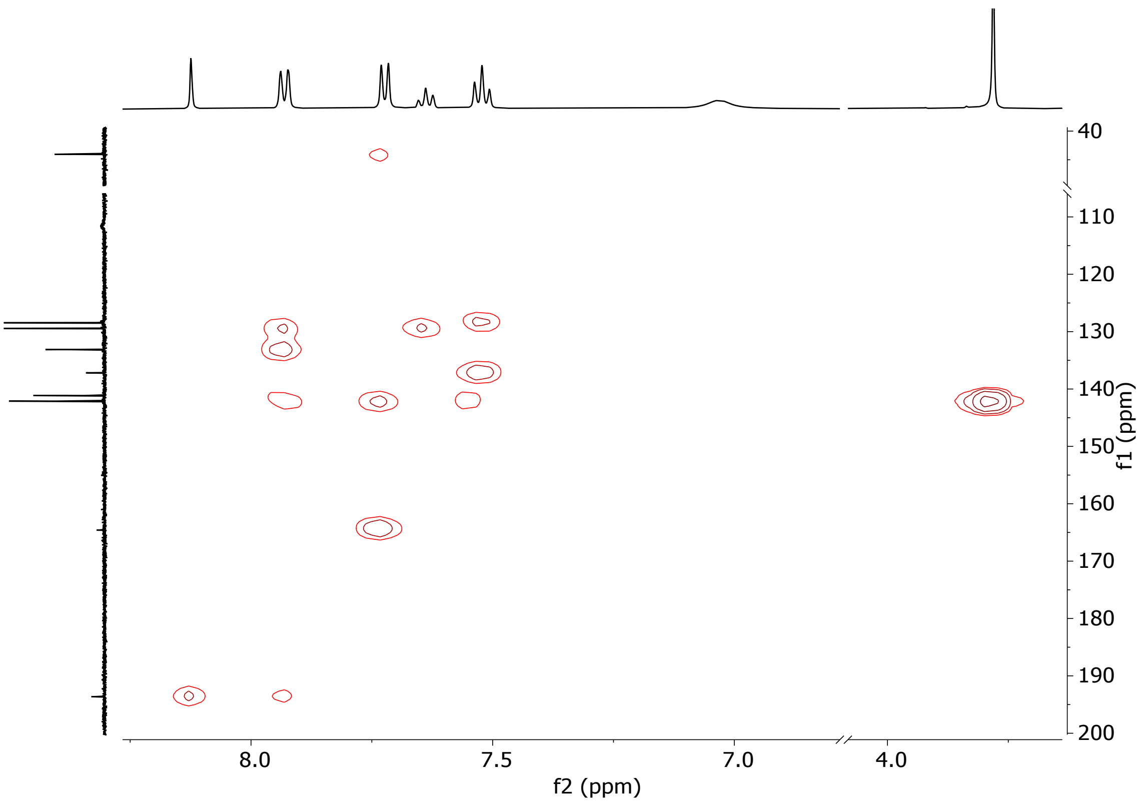

Figure S38. ^{1}H-^{13}C HMBC (500 MHz, D_2O) partial spectrum of the compound **P**$_a$H·Cl as synthesized.

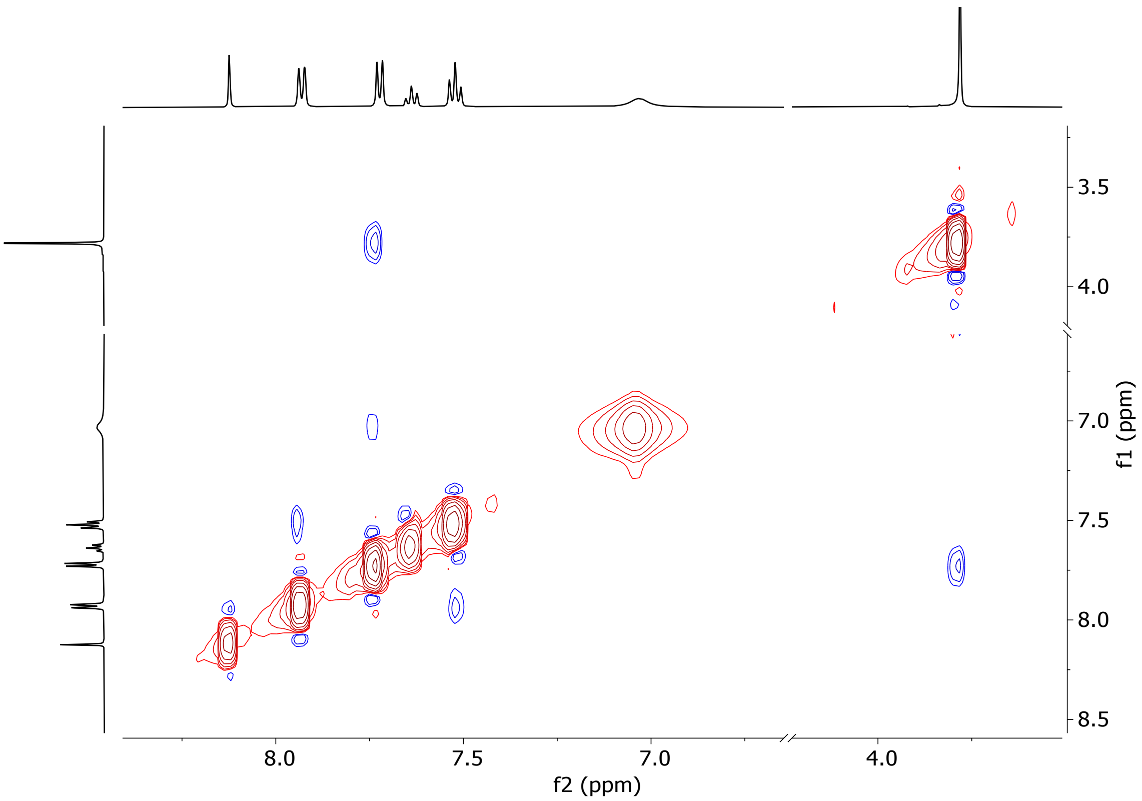

Figure S39. ^{1}H-^{1}H NOESY (500 MHz, D_2O) partial spectrum of the compound **P**$_a$H·Cl as synthesized. Blue cross peaks indicate NOE correlations**.**

2.1.4. NMR data for the species assigned as *Z*-P_a.

$P_aH·Cl$ salt was dissolved at 5 mM in a D_2O phosphate buffer solution (20 mM, pD 6). The NMR tube containing the compound solution was irradiated for 16 h at 254 nm. Immediately before data acquisition, the sample was basified to pD 13 using phosphate buffer (40 mM), resulting in a final compound concentration of 2.5 mM. The interval between basification and acquisition was approximately 1 min. For the ^1H and COSY experiments, 4 scans were applied in both cases, with acquisition times of ~1 min and ~60 min, respectively.

^{1}H NMR (500 MHz, D_2O) δ (*ppm*): 7.97 (d, J = 7.3 Hz, 2H), 7.77 (s, 1H), 7.70 (t, J = 6.9 Hz, 1H), 7.62 (d, J = 7.6 Hz, 2H), 7.57 (t, J = 7.6 Hz, 2H), 6.86 (d, J = 7.2 Hz, 2H), 3.76 (s, 3H).

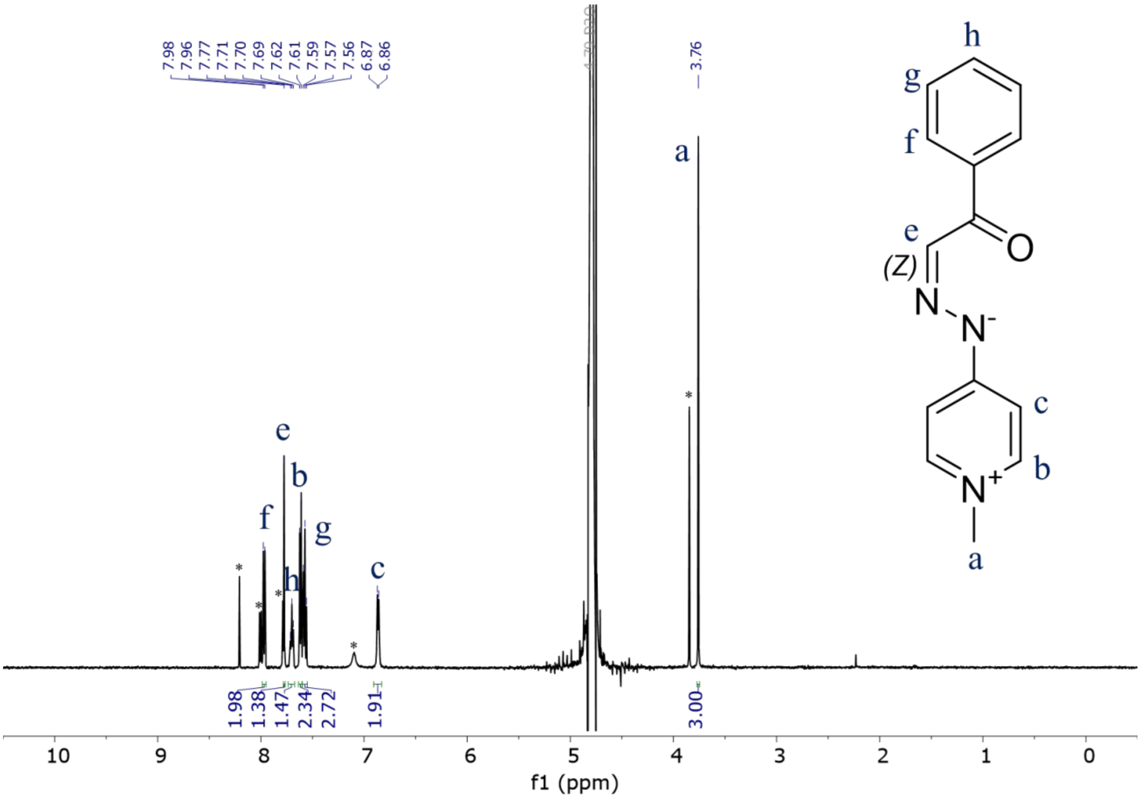

Figure S40. ^{1}H NMR (500 MHz, D_2O) spectrum of the basification after reaching the 254 nm PSS for the compound $P_aH·Cl$. Signals marked with an asterisk correspond to the *E*-isomer.

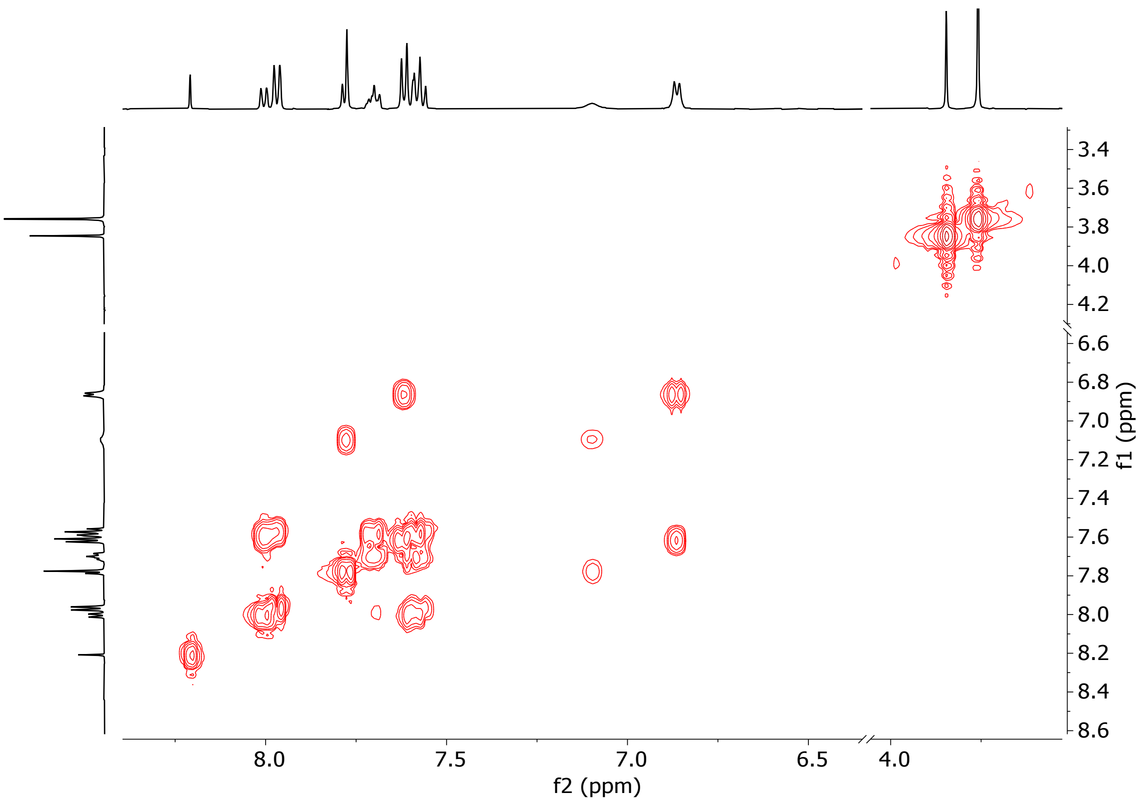

Figure S41. ¹H-¹H COSY (500 MHz, D_2O) partial spectrum of the basification after reaching the 254 nm PSS for the compound $\mathbf{P}_a$H·Cl. Due to the acquisition time of the experiment and the fast thermal reversion, cross-peaks of *E*-isomer exhibit a greater intensity than observed in the ¹H NMR. Signals marked with an asterisk correspond to the *E*-isomer.

2.2 Synthesis and characterization data of **1**·I.

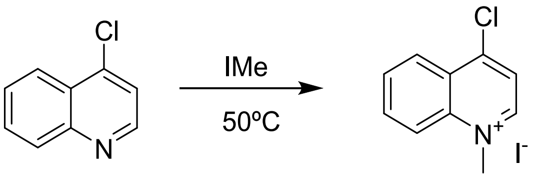

4-Chloroquinoline (2 g, 12.3 mmol, 1 equiv) was dissolved in 3.8 mL of iodomethane in a Schlenk flask. The reaction mixture was heated at 50ºC in a magnetic hot plate stirrer for 6h. After the reaction was completed, 25 mL of Et_2O was added, and the resulting precipitate was filtered, washed with Et_2O (3 x 15 mL), and dried under vacuum to yield precursor **1**·I as a yellow solid (3.32 g, 89%).

^{1}H NMR (400 MHz, D_2O) δ (*ppm*): 9.16 (d, J = 6.4 Hz, 1H), 8.67 (dd, J = 8.6, 1.4 Hz, 1H), 8.43 (d, J = 8.9 Hz, 1H), 8.33 (ddd, J = 8.8, 7.0, 1.4 Hz, 1H), 8.20 (d, J = 6.4 Hz, 1H), 8.13 (ddd, J = 8.3, 7.0, 1.0 Hz, 1H), 4.64 (s, 3H).

^{13}C{^{1}H} NMR (101 MHz, D_2O) δ (*ppm*): 154.0 (C_{Ar}), 148.7 (CH_{Ar}), 139.3 (C_{Ar}), 136.5 (CH_{Ar}), 131.0 (CH_{Ar}), 127.8 (C_{Ar}), 126.7 (CH_{Ar}), 122.1 (CH_{Ar}), 119.0 (CH_{Ar}), 45.3 (CH_3).

HRMS (ESI) m/z: Calcd for $C_{10}H_9ClN^+$ 178,0418; found 178.0417.

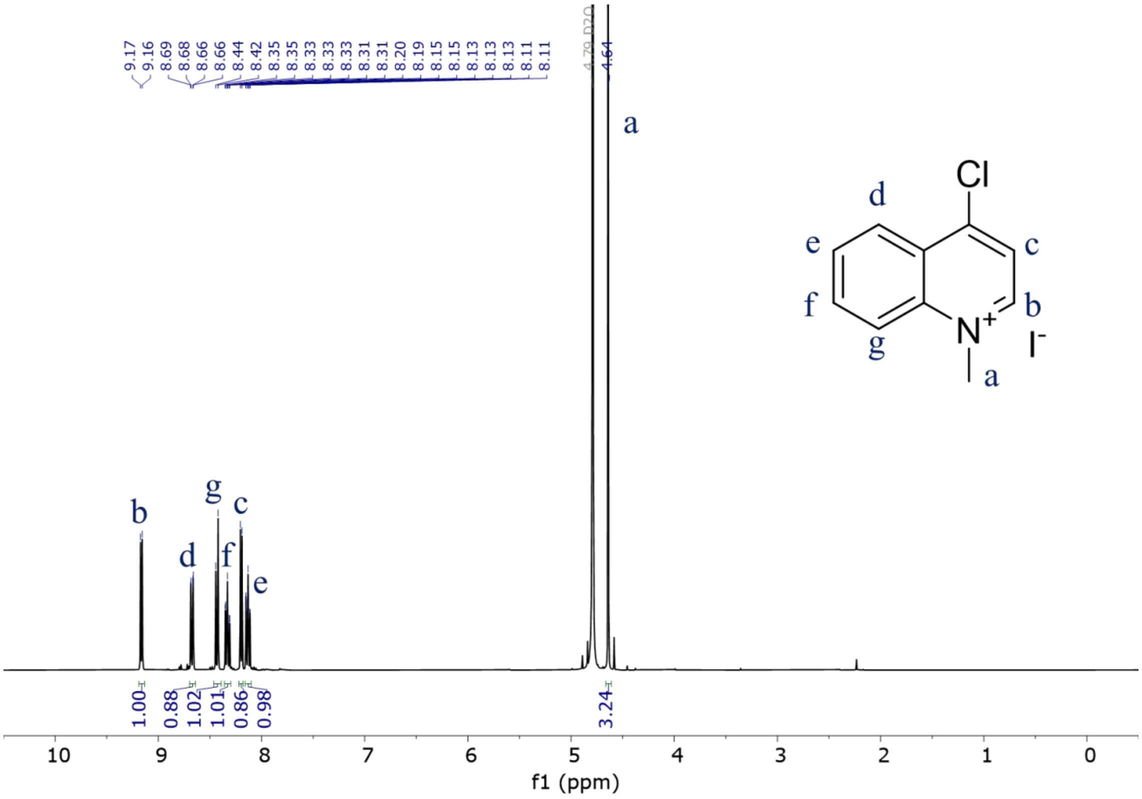

Figure S42. ^{1}H NMR (400 MHz, D_2O) spectrum of **1**·I.

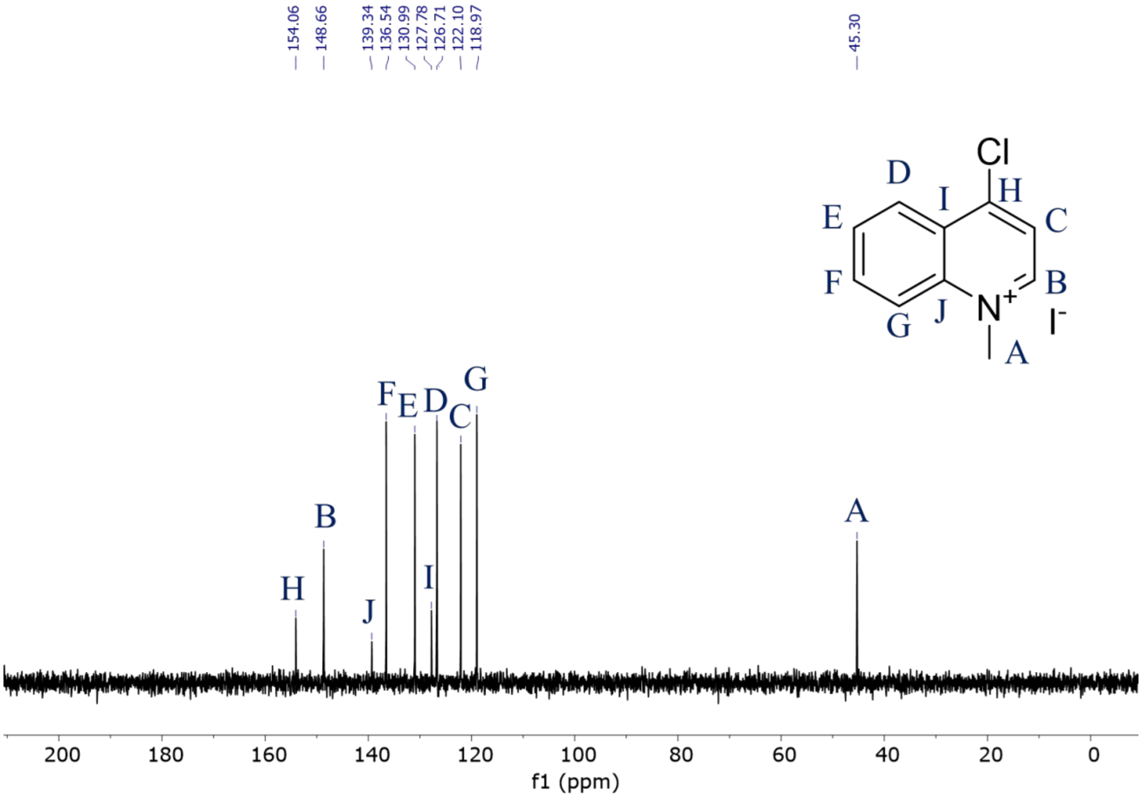

Figure S43. $^{13}C\{^1H\}$ NMR (101 MHz, D_2O) spectrum of **1**·I.

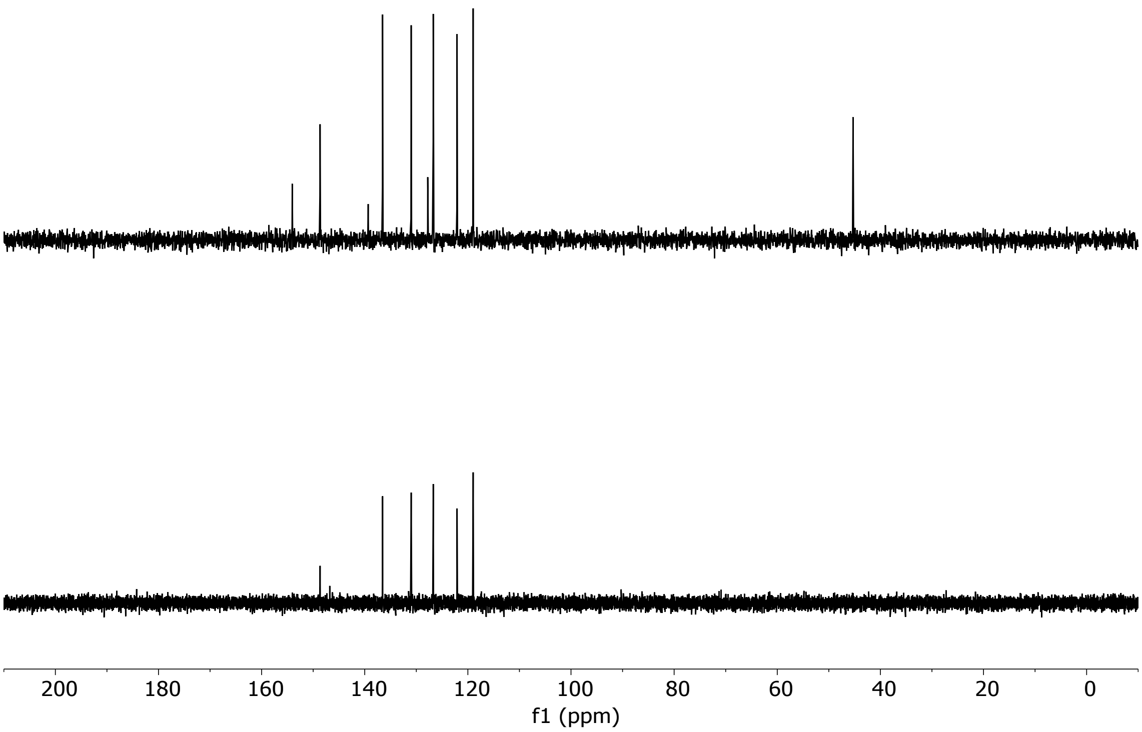

Figure S44. DEPT-135 NMR (101 MHz, D_2O) and $^{13}C\{^1H\}$ NMR (101 MHz, D_2O) stacked spectra of **1**·I.

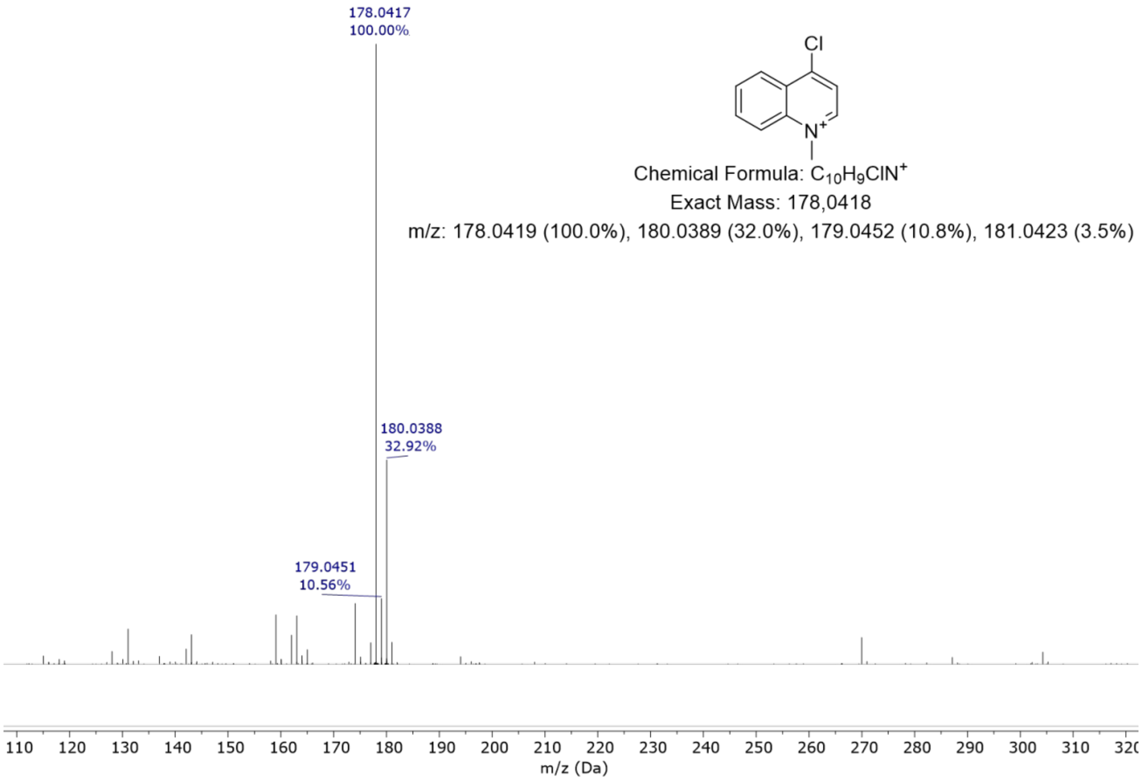

Figure S45. HRMS-ESI spectrum of **1**·I.

2.3 Synthesis and characterization data of H_b·I.

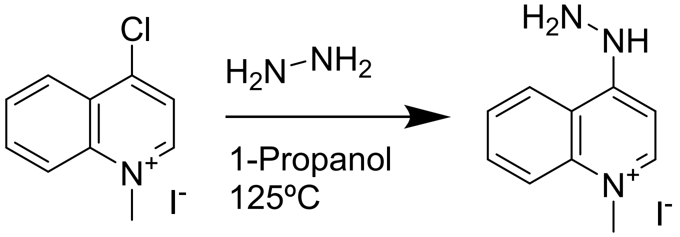

1·I (1 g, 3.3 mmol, 1 equiv) was dissolved in 200 mL of hot 1-propanol in a round bottom flask. Then, 2mL of hydrazine hydrate (40-60 %, 32.7 mmol, 10 equiv) were added, and the reaction mixture was heated in a magnetic hot plate stirrer at 125 ºC for 1.5h. The resulting precipitate was filtered, washed with propanol (3 x 10 mL) and Et_2O (3 x 10 mL), and dried under vacuum to yield $\mathbf{H_b}$·I as a pink solid (960 mg, 98%).

^{1}H NMR (500 MHz, DMSO-d6) δ (*ppm*): 10.68 (s, 1H), 8.54 (d, *J* = 7.4 Hz, 1H), 8.45 (d, *J* = 8.4 Hz, 1H), 8.06 – 7.99 (m, 2H), 7.75 – 7.71 (m, 1H), 7.14 (d, *J* = 7.4 Hz, 1H), 5.25 (s, 2H), 4.08 (s, 3H).

^{13}C{^{1}H} NMR (126 MHz, DMSO-d6) δ (*ppm*): 155.8 (C_{Ar}), 147.0 (CH_{Ar}), 138.2 (C_{Ar}), 133.7 (CH_{Ar}), 126.5 (CH_{Ar}), 123.2 (CH_{Ar}), 118.4 (CH_{Ar}), 115.6 (C_{Ar}), 97.7 (CH_{Ar}), 41.8 (CH_3).

HRMS (ESI) m/z: Calcd for $C_{10}H_{12}N_3^+$ 174.1026; found 174.1025.

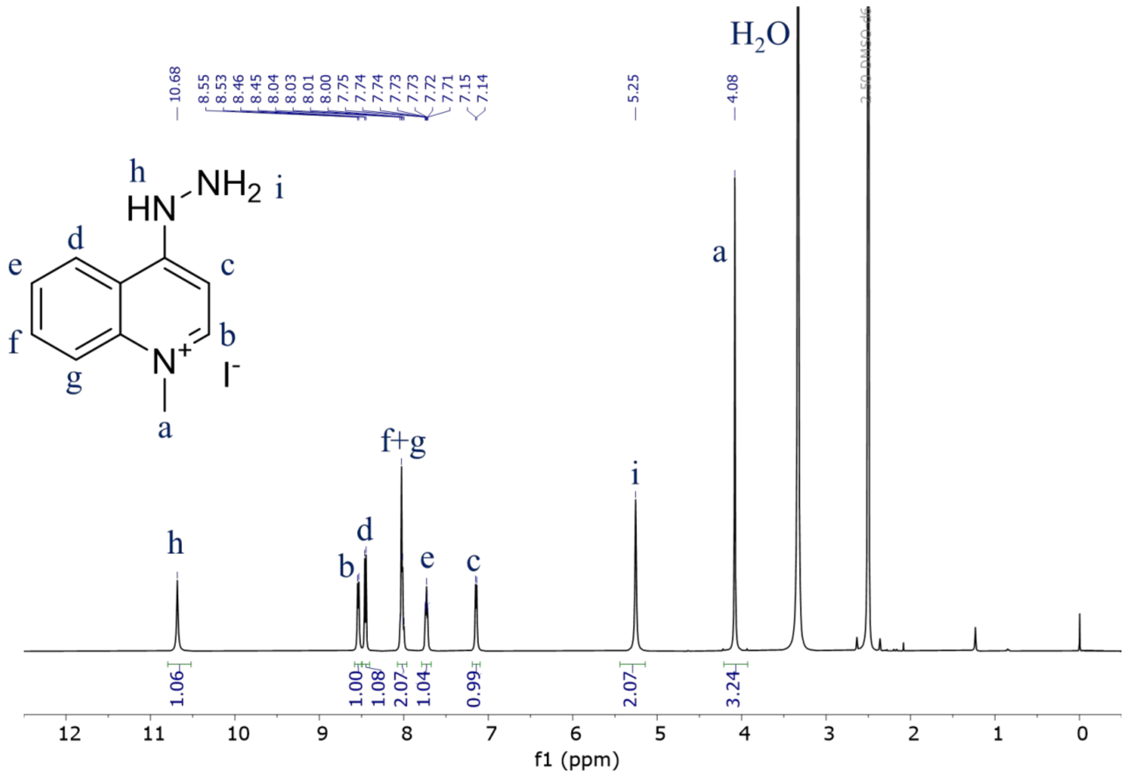

Figure S46. ^{1}H NMR (500 MHz, DMSO-d6) spectrum of $\mathbf{H_b}$·I.

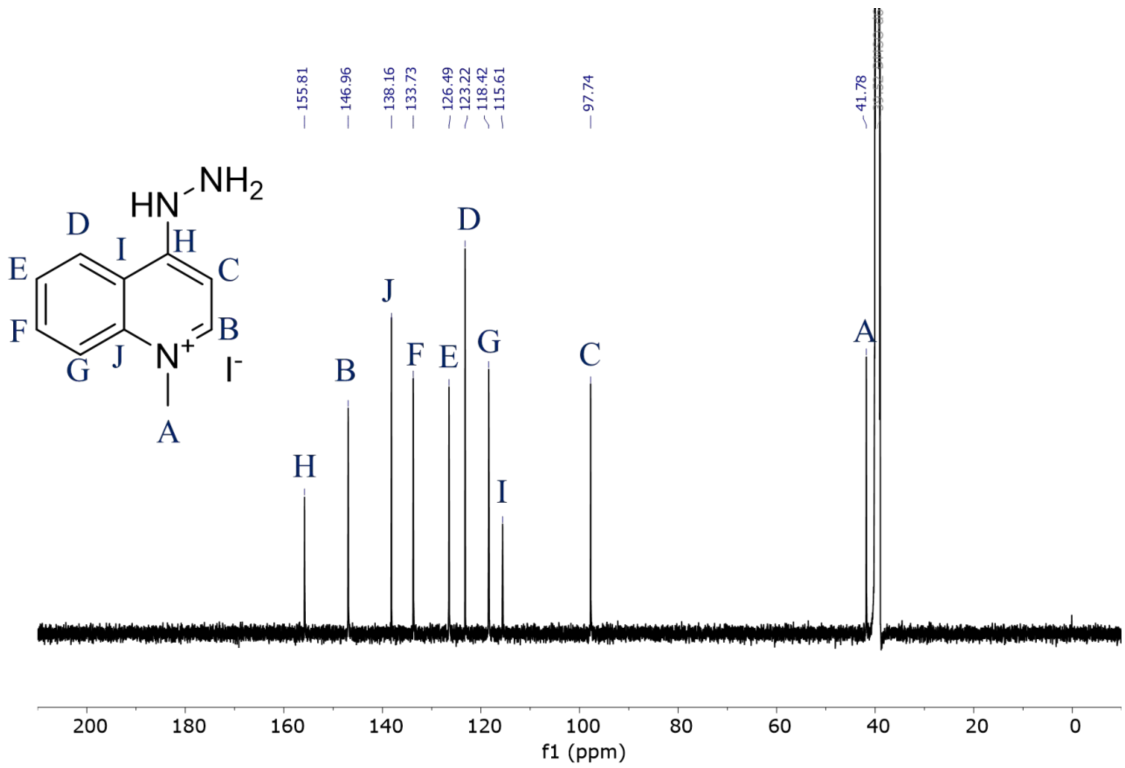

Figure S47. $^{13}C\{^{1}H\}$ NMR (126 MHz, DMSO-d6) spectrum of $\mathbf{H}_b$·I.

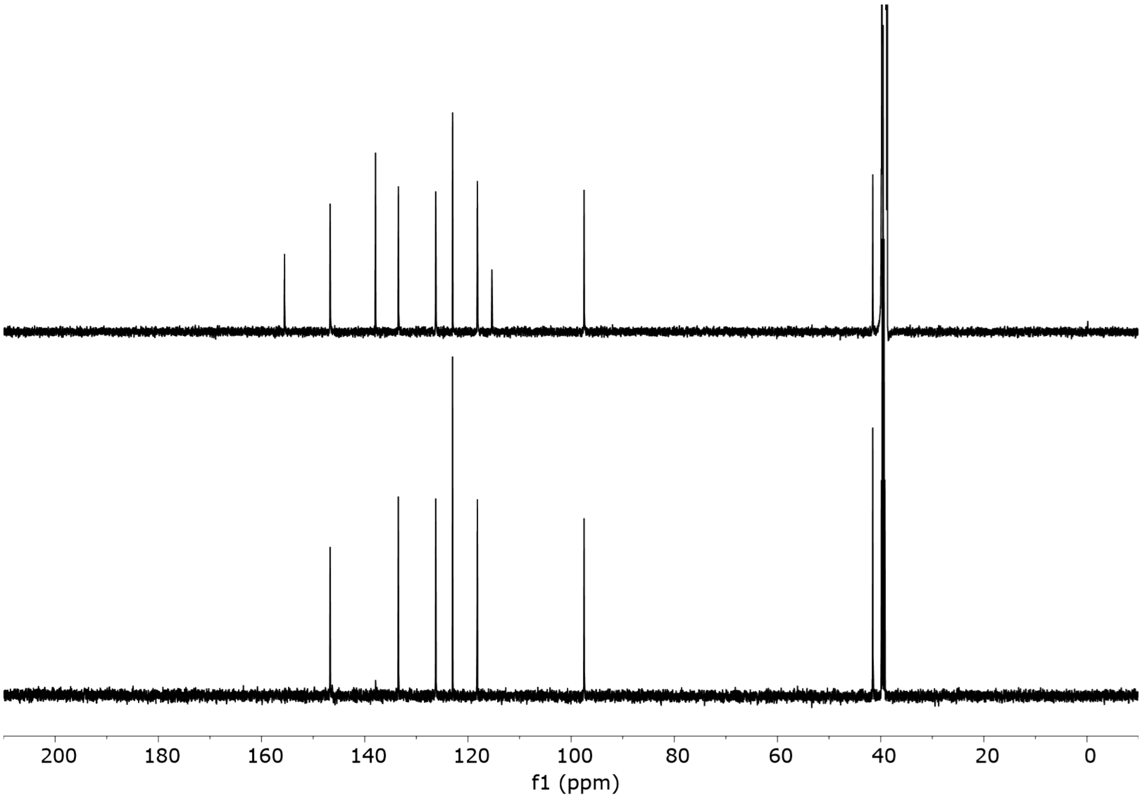

Figure S48. DEPT-135 NMR (126 MHz, DMSO-d6) and $^{13}C\{^{1}H\}$ NMR (126 MHz, DMSO-d_6) stacked spectra of $\mathbf{H}_b$·I.

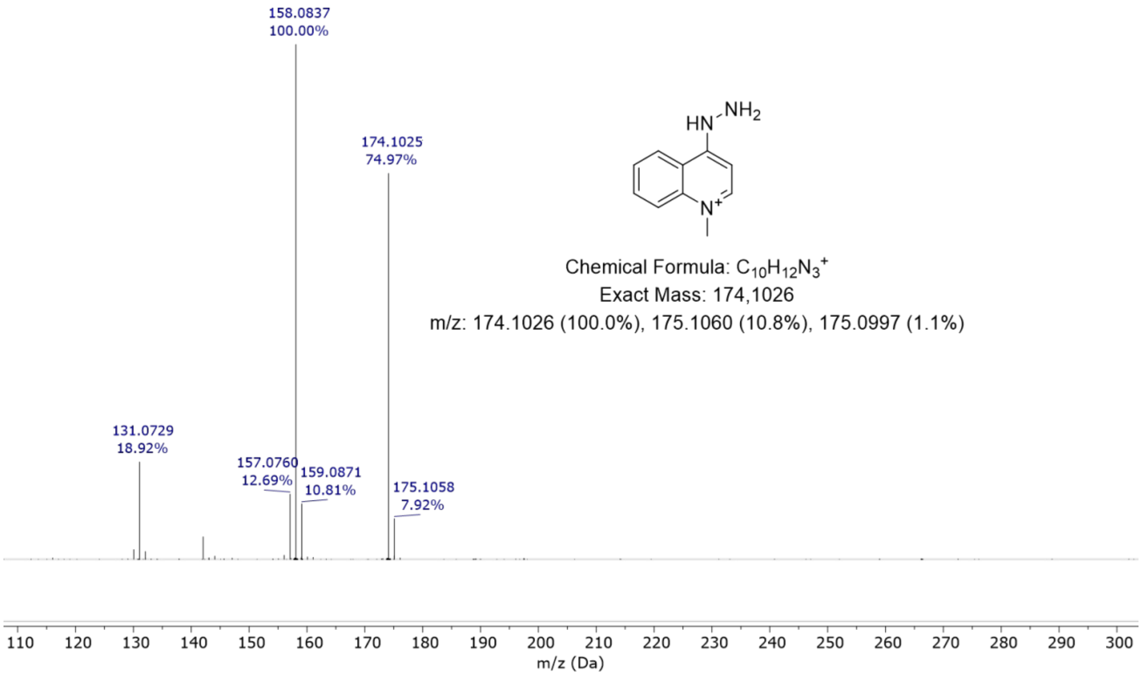

Figure S49. HRMS-ESI spectrum of $\mathbf{H}_b$·I.

2.4 Synthesis and characterization data of $P_bH{\cdot}Cl/PF_6$.

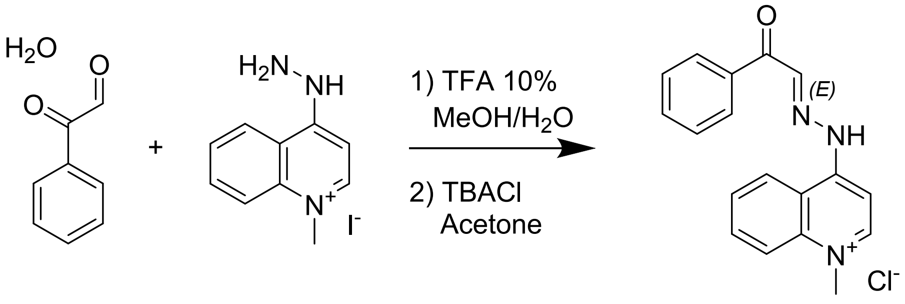

H$_b$·I (690 mg, 2.29 mmol, 1 equiv) was dissolved in 120 mL of H_2O and 40 mL of MeOH, and then, TFA (17.5 µL, 0.229 mmol, 0.1 equiv) was added. Then, phenylglyoxal monohydrate (383 mg, 2.52 mmol, 1.1 equiv) was added, and the reaction mixture was heated in a magnetic hot plate stirrer at 60 ºC for 4h in the absence of light. After reaction completion, the solvent was removed under reduced pressure, and the resulting residue was washed with H_2O (2 x 10 mL), Et_2O (2 x 10 mL) and dried under vacuum. The solid was dissolved in acetone and a saturated solution of TBACl in acetone was added. After stirring for 1 h, the resulting precipitate was filtered and washed with acetone (2 x 10 mL) and Et_2O (2 x 10 mL), and dried under vacuum to yield **P**$_b$H·Cl as a yellow solid (509 mg, 53 %).

In order to obtain the compound as a PF_6^- salt, **P**$_b$H·Cl (200 mg, 0.6 mmol, 1 equiv) was dissolved in 50 mL of H_2O. KPF_6 (440 mg, 2.4 mmol, 4 equiv) was added to the solution and the resulting precipitate was filtered, washed with H_2O (3 × 10 mL) and Et_2O (3 × 10 mL), and dried under vacuum to yield the product **P**$_b$H·PF_6 as a yellow solid (250 mg, 95%).

HRMS (ESI) m/z: Calcd for $C_{18}H_{16}N_3O^+$ 290.1288; found 290.1288.

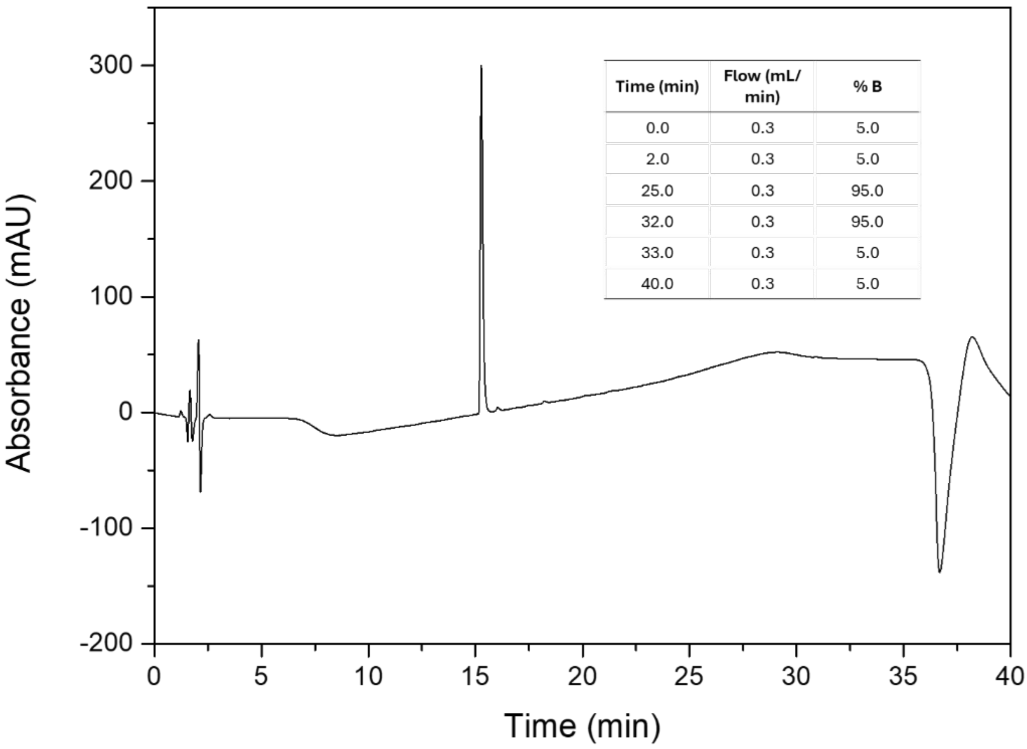

Time (min)	Flow (mL/ min)	% B
0.0	0.3	5.0
2.0	0.3	5.0
25.0	0.3	95.0
32.0	0.3	95.0
33.0	0.3	5.0
40.0	0.3	5.0

Figure S50. HPLC chromatogram (220 nm) of $\mathbf{P}_b$H·Cl at t_R = 15.27 min (Inset: separation method; A = H_2O + 0.04 % TFA, B = CH_3CN + 0.04 % TFA).

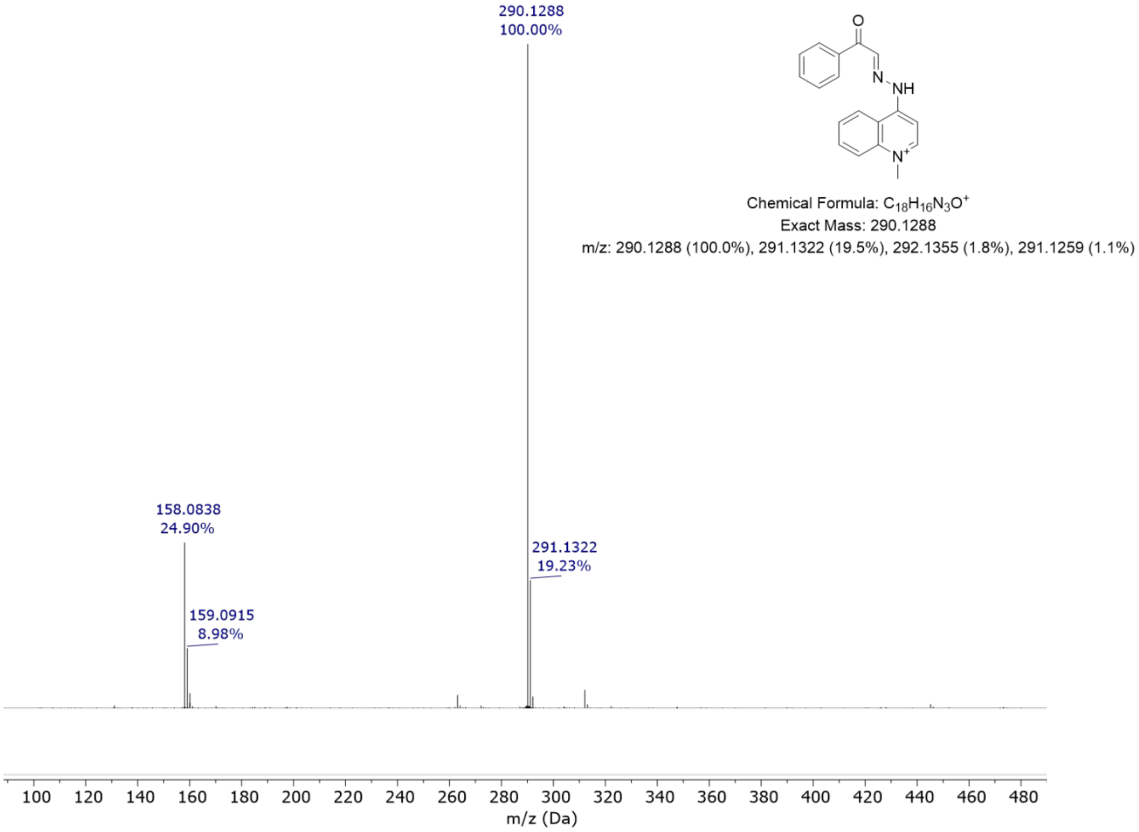

Figure S51. HRMS-ESI spectrum of $\mathbf{P}_b$H·Cl.

2.4.1. NMR data for the species assigned as *E*-P_bH^+.

2.4.1.1. Aqueous medium.

P_bH·Cl salt was dissolved at 5 mM in a D_2O phosphate buffer solution (20 mM, pD 5).

^{1}H NMR (500 MHz, D_2O) δ (*ppm*): 8.49 (d, J = 7.0 Hz, 1H), 8.33 (s, 1H), 8.25 (d, J = 8.4 Hz, 1H), 7.98 (t, J = 7.8 Hz, 1H), 7.92 (d, J = 8.8 Hz, 1H), 7.77 (dd, J = 8.3, 2.8 Hz, 3H), 7.62 (t, J = 7.4 Hz, 1H), 7.42 (q, J = 7.0 Hz, 3H), 4.13 (s, 3H).

^{13}C{^{1}H} NMR (126 MHz, D_2O) δ (*ppm*): 189.3 (C=O), 152.5 (C_{Ar}), 147.6 (CH_{Ar}), 142.9 (CH=N), 138.7 (C_{Ar}), 135.1 (CH_{Ar}), 134.7 (C_{Ar}), 134.6 (CH_{Ar}), 129.4 (CH_{Ar}), 128.7 (CH_{Ar}), 128.4 (CH_{Ar}), 122.8 (CH_{Ar}), 118.3 (CH_{Ar}), 116.4 (C_{Ar}), 102.0 (CH_{Ar}), 43.1 (CH_3).

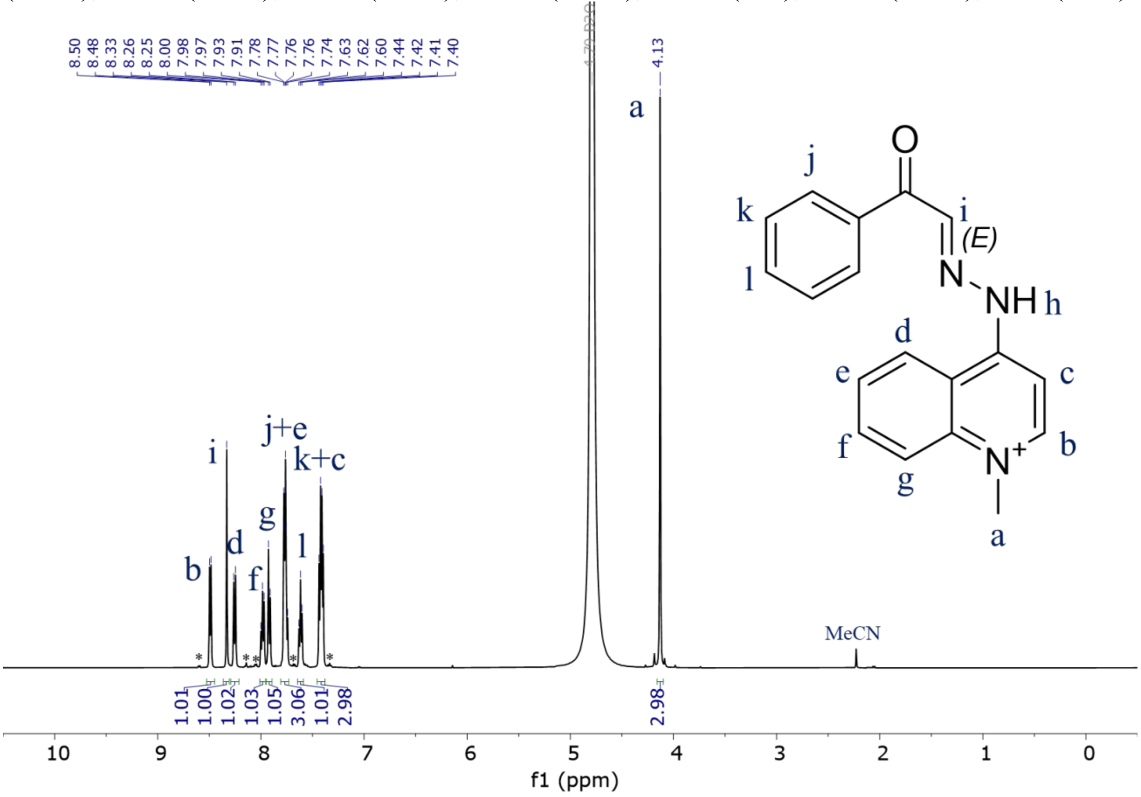

Figure S52. ^{1}H NMR (500 MHz, D_2O) spectrum of the compound P_bH·Cl as synthesized. Signals marked with an asterisk correspond to the *Z*-isomer.

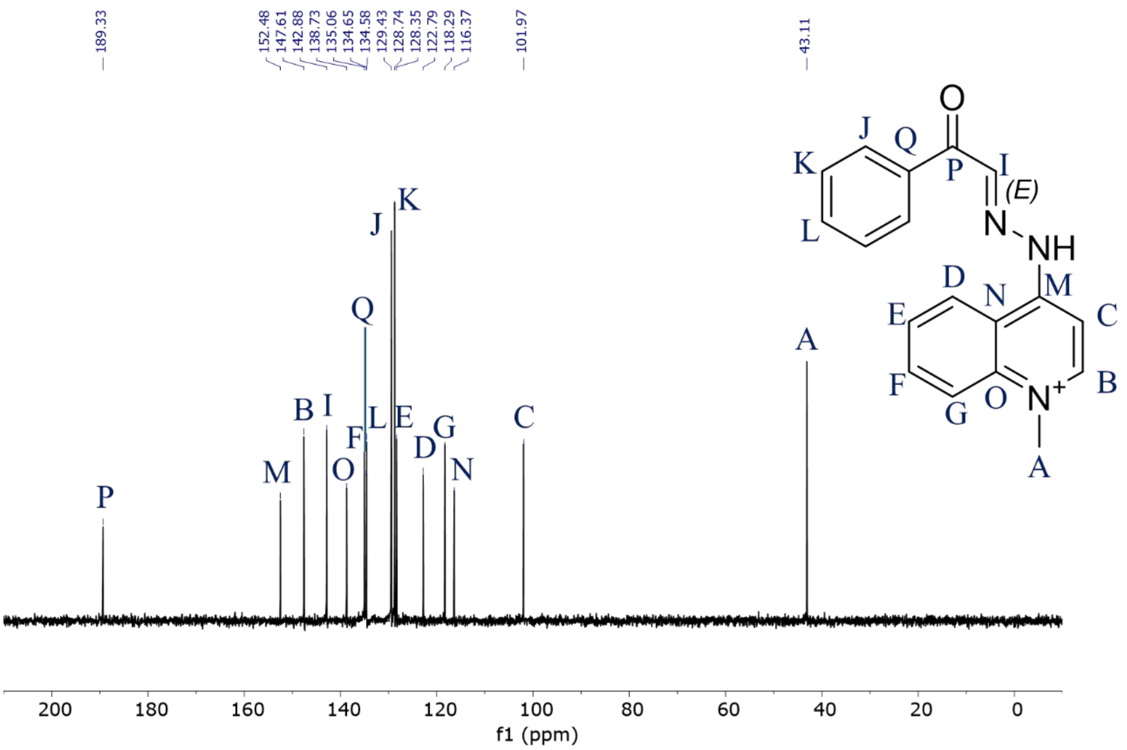

Figure S53. $^{13}C\{^{1}H\}$ NMR (126 MHz, D_2O) spectrum of the compound $\mathbf{P}_b$H·Cl as synthesized.

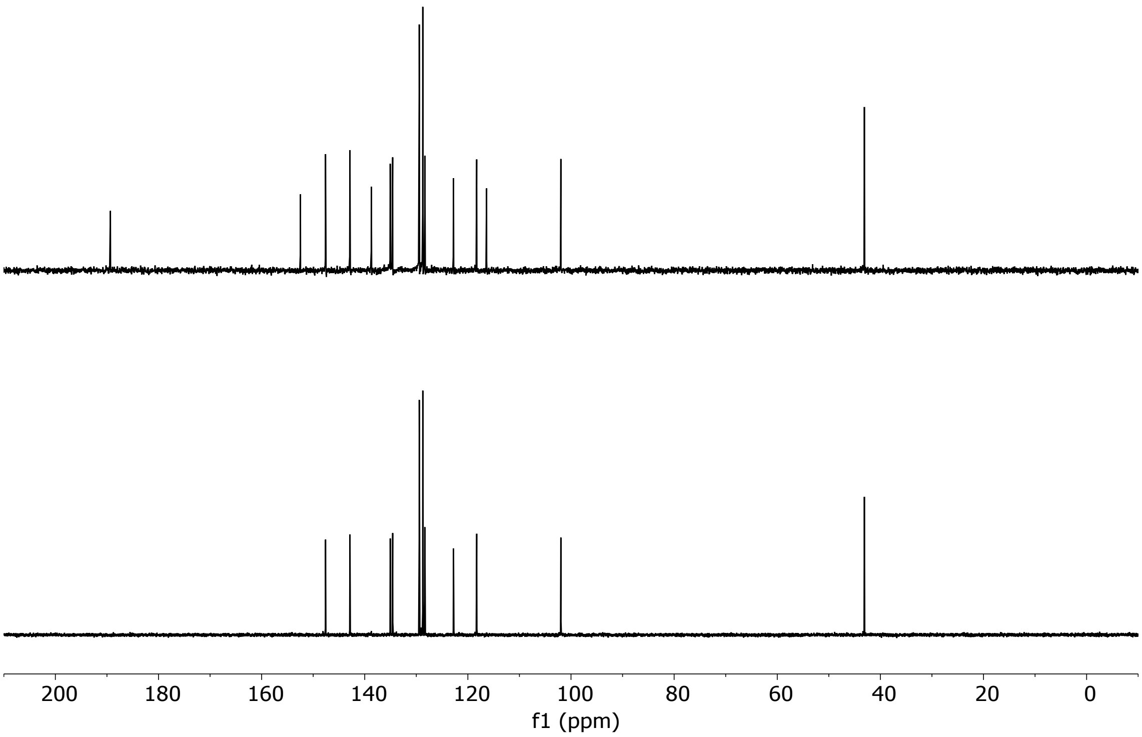

Figure S54. DEPT-135 NMR (126 MHz, D_2O) and $^{13}C\{^{1}H\}$ NMR (126 MHz, D_2O) stacked spectra of the compound $\mathbf{P}_b$H·Cl as synthesized.

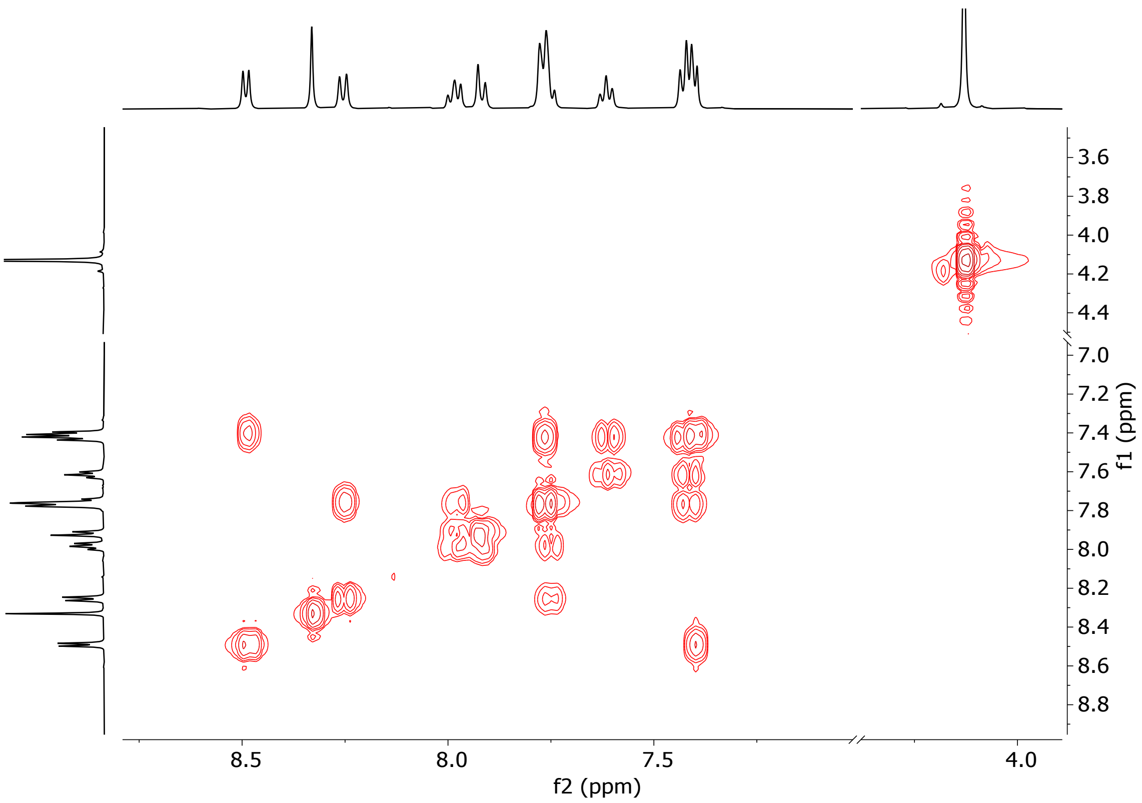

Figure S55. ^{1}H-^{1}H COSY (500 MHz, D_2O) partial spectrum of the compound $\mathbf{P}_b$H·Cl as synthesized.

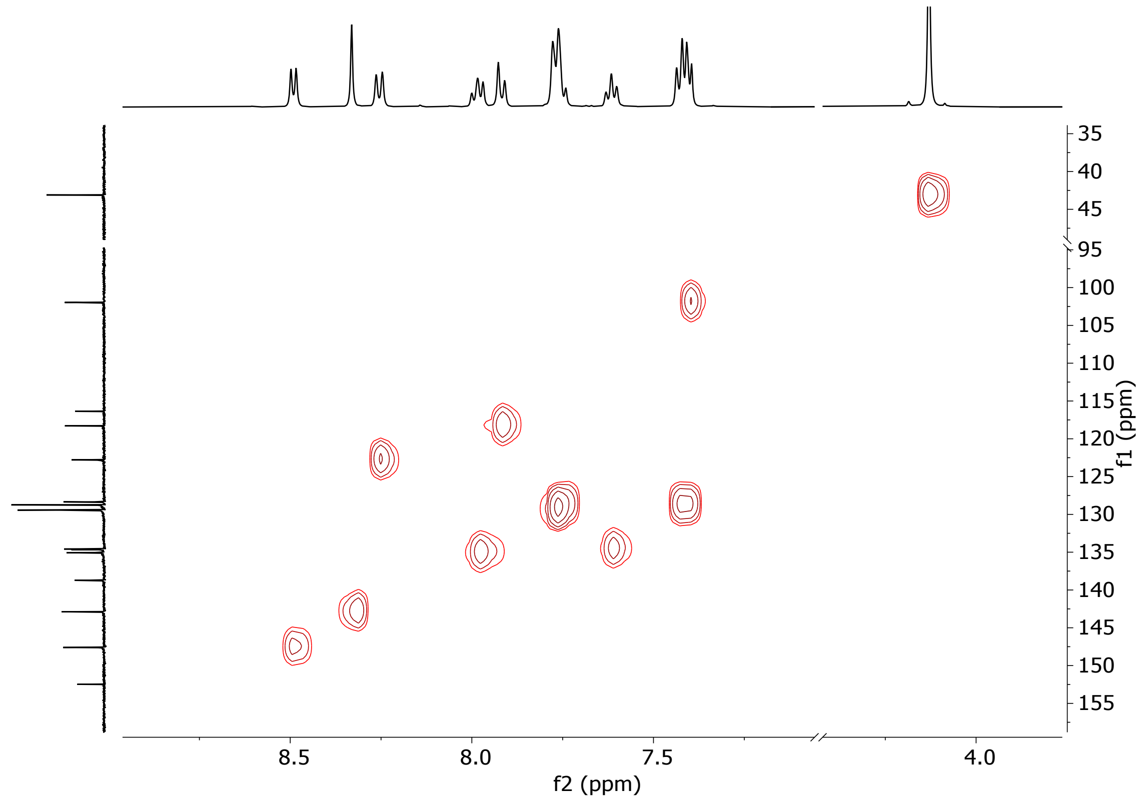

Figure S56. ^{1}H-^{13}C HSQC (500 MHz, D_2O) partial spectrum of the compound $\mathbf{P}_b$H·Cl as synthesized.

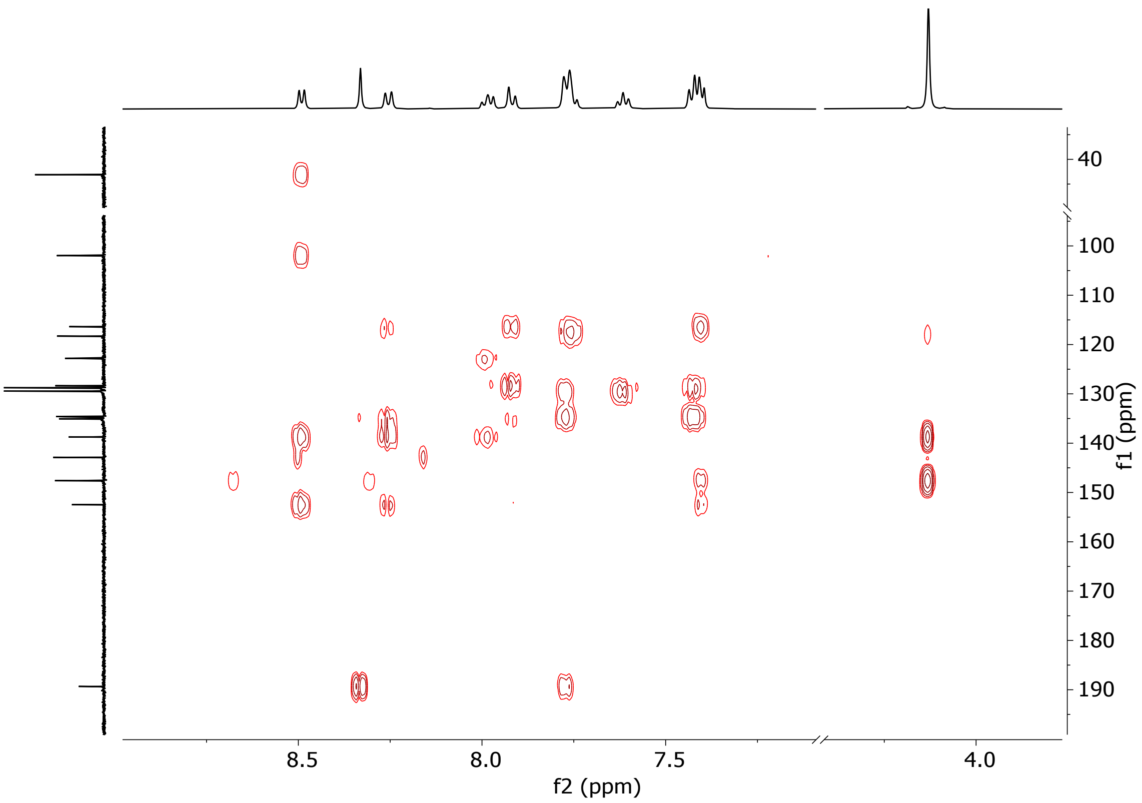

Figure S57. ^{1}H-^{13}C HMBC (500 MHz, D_2O) partial spectrum of the compound $\mathbf{P_b}H{\cdot}Cl$ as synthesized.

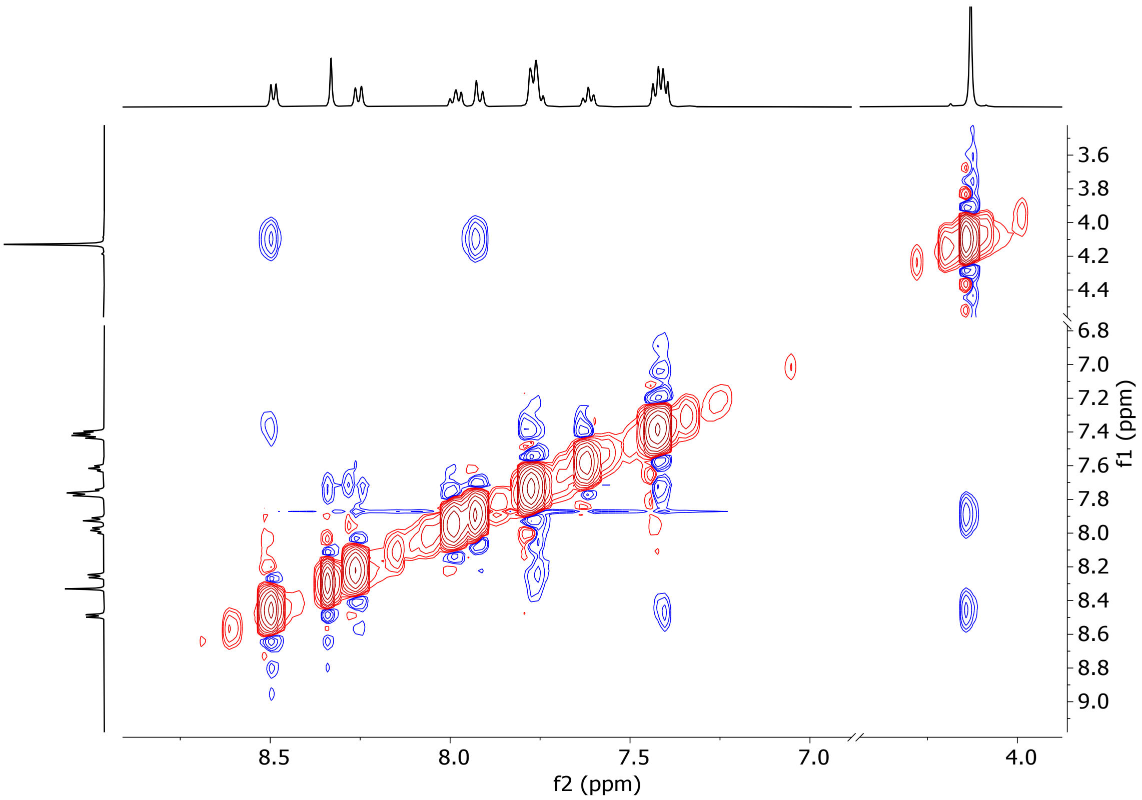

Figure S58. ^{1}H-^{1}H NOESY (500 MHz, D_2O) partial spectrum of the compound $\mathbf{P_b}H{\cdot}Cl$ as synthesized. Blue cross peaks indicate NOE correlations.

2.4.1.2. Organic medium.

P$_b$H·PF$_6$ salt was dissolved at 5 mM in CD_3CN.

^{1}H NMR (500 MHz, CD_3CN) *δ* (*ppm*): 10.93 (s, 1H), 8.54 (d, *J* = 7.1 Hz, 1H), 8.43 (d, *J* = 8.4 Hz, 1H), 8.29 (s, 1H), 8.18 – 8.16 (m, 2H), 8.14 (d, *J* = 7.2 Hz, 2H), 7.95 (ddd, *J* = 8.3, 5.0, 3.1 Hz, 1H), 7.73 (t, *J* = 7.4 Hz, 1H), 7.62 (t, *J* = 7.8 Hz, 1H), 7.55 (d, *J* = 7.1 Hz, 1H), 4.26 (s, 3H).

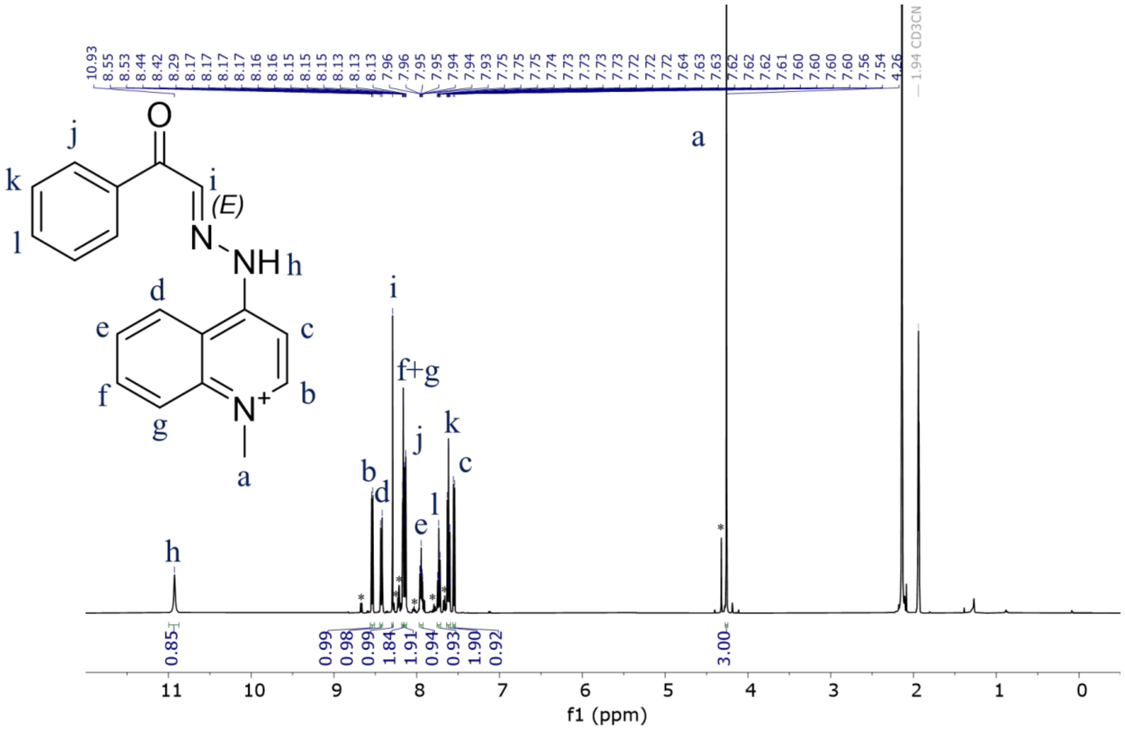

Figure S59. ^{1}H NMR (500 MHz, CD_3CN) spectrum of the compound **P**$_b$H·PF$_6$ as synthesized. Signals marked with an asterisk correspond to the *Z*-isomer.

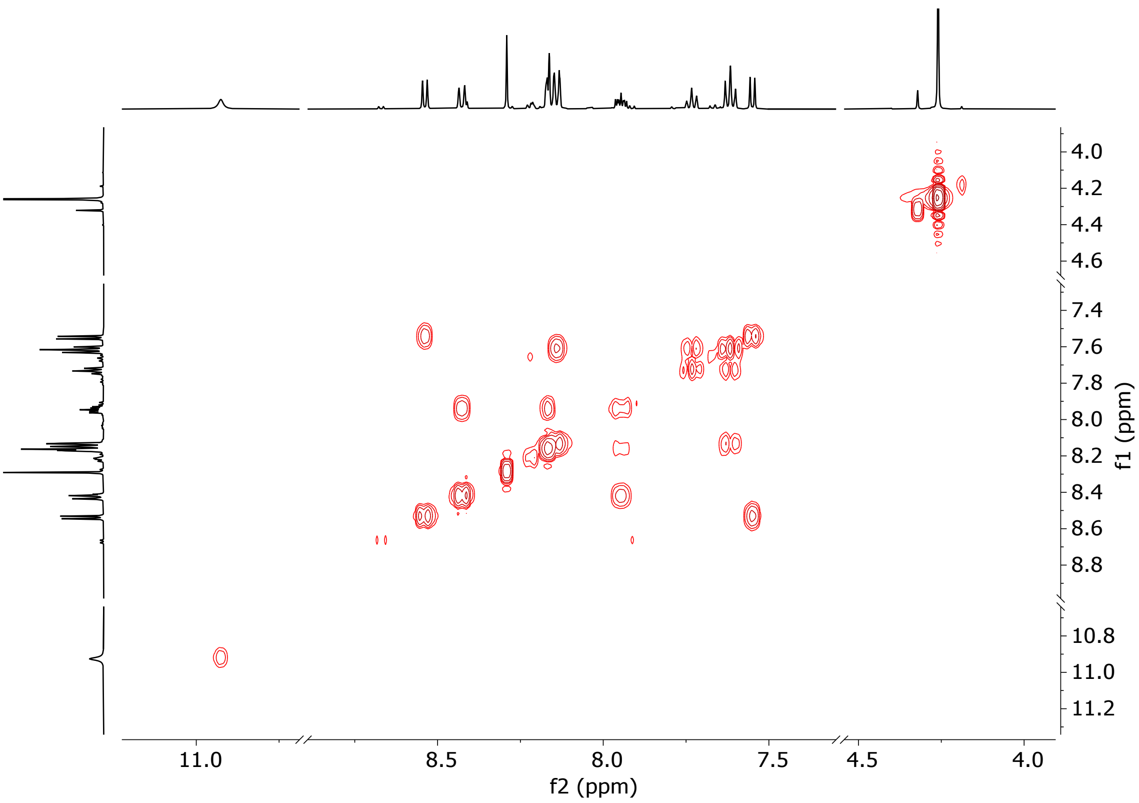

Figure S60. ^{1}H-^{1}H COSY (500 MHz, CD_3CN) partial spectrum of the compound $\mathbf{P}_b$H·PF_6 as synthesized.

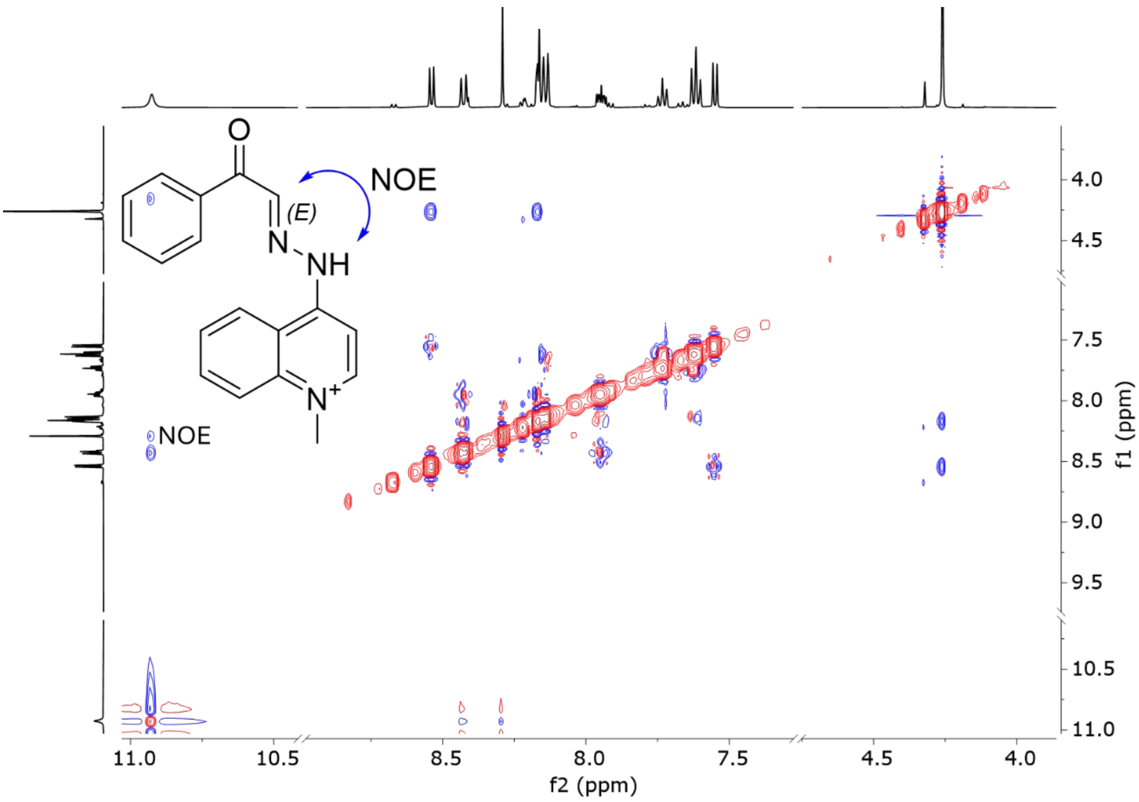

Figure S61. ^{1}H-^{1}H NOESY (500 MHz, CD_3CN) partial spectrum of the compound $\mathbf{P}_b$H·PF_6 as synthesized. Blue cross peaks indicate NOE correlations. The observed NOE between the NH and the imine proton is diagnostic of the *E*-isomer.

2.4.2. NMR data of the species assigned as *Z*-P_bH^+.

2.4.2.1. Aqueous medium.

P_bH·Cl salt was dissolved at 5 mM in a D_2O phosphate buffer solution (20 mM, pD 5). The NMR tube containing the compound solution was irradiated for 16 h at 254 nm, after which all NMR experiments were acquired.

^{1}H NMR (500 MHz, D_2O) δ (*ppm*): 8.67 (d, J = 7.0 Hz, 1H), 8.23 (s, 1H), 8.11 (dd, J = 8.7, 7.0 Hz, 1H), 8.06 (d, J = 8.7 Hz, 2H), 7.92 (t, J = 7.7 Hz, 1H), 7.87 (d, J = 7.9 Hz, 2H), 7.79 (d, J = 7.0 Hz, 1H), 7.66 (t, J = 7.4 Hz, 1H), 7.44 (t, J = 7.7 Hz, 2H), 4.24 (s, 3H).

^{13}C{^{1}H} NMR (126 MHz, D_2O) δ (*ppm*): 188.5 (C=O), 151.9 (C_{Ar}), 148.2 (CH_{Ar}), 139.0 (C_{Ar}), 135.3 (CH_{Ar}), 135.3 (CH_{Ar}), 134.8 (C_{Ar}), 133.2 (CH=N), 129.2 (CH_{Ar}), 129.0 (CH_{Ar}), 128.9 (CH_{Ar}), 121.9 (CH_{Ar}), 118.7 (CH_{Ar}), 116.4 (C_{Ar}), 101.9 (CH_{Ar}), 43.4 (CH_3).

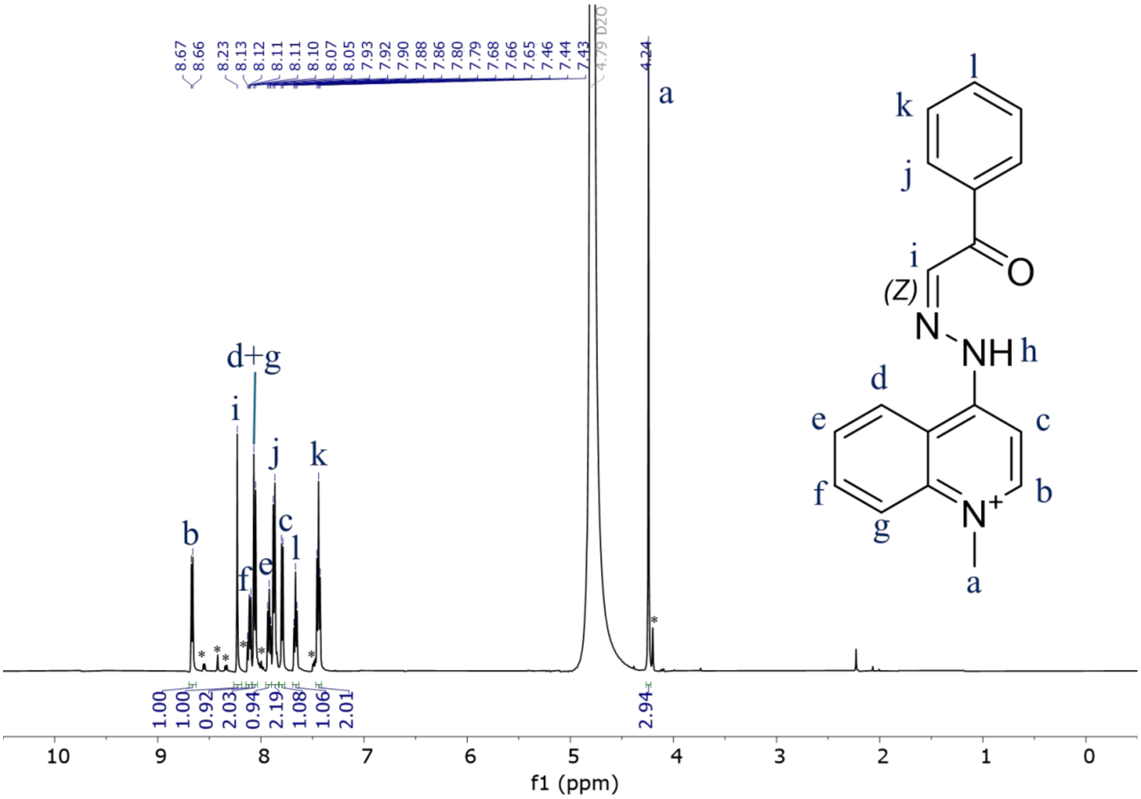

Figure S62. ^{1}H NMR (500 MHz, D_2O) spectrum of the 254 nm PSS for the compound **P_b**H·Cl. Signals marked with an asterisk correspond to the *E*-isomer.

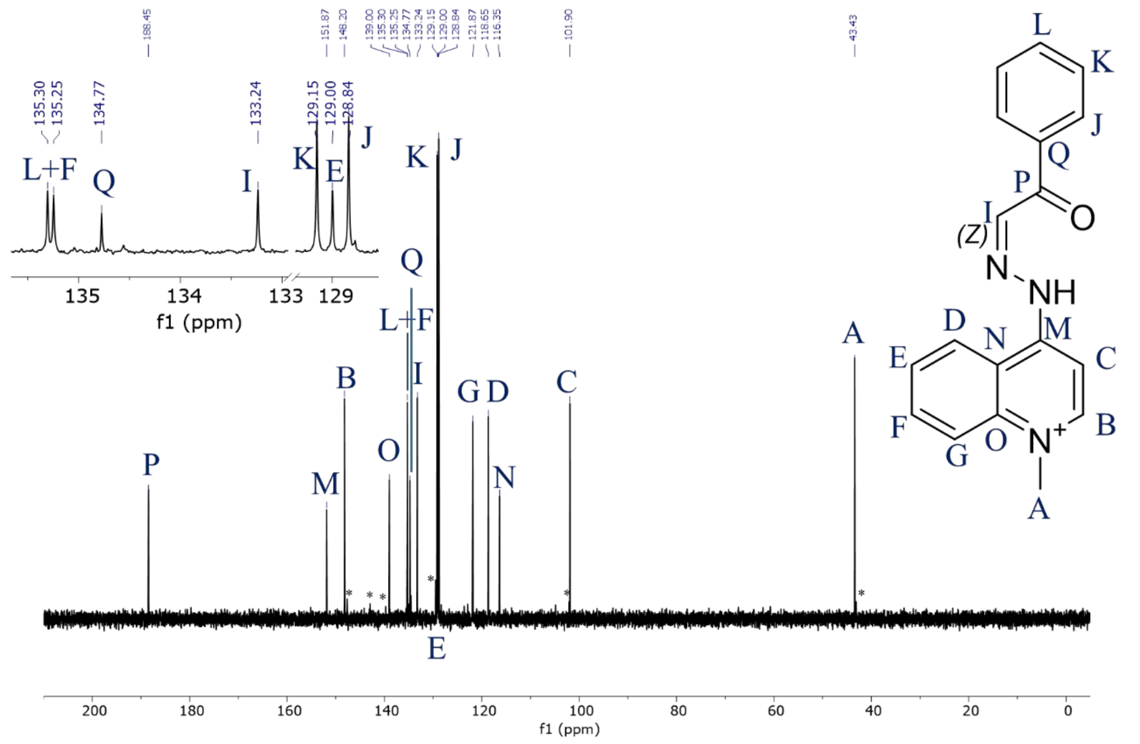

Figure S63. $^{13}C\{^{1}H\}$ NMR (126 MHz, D_2O) spectrum of the 254 nm PSS for the compound $\mathbf{P_b}$H·Cl. Signals marked with an asterisk correspond to the *E*-isomer. *Inset*: Partial region of the spectrum between 136-128 ppm.

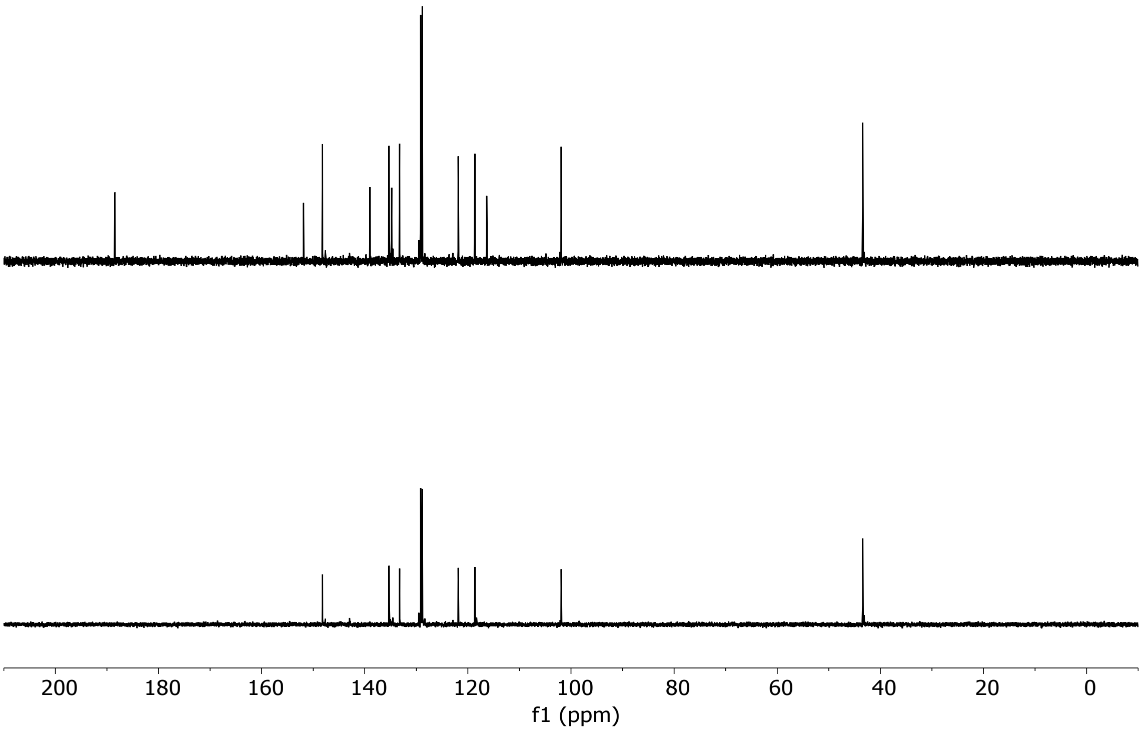

Figure S64. DEPT-135 NMR (126 MHz, D_2O) and $^{13}C\{^{1}H\}$ NMR (126 MHz, D_2O) stacked spectra of the 254 nm PSS for the compound $\mathbf{P_b}$H·Cl.

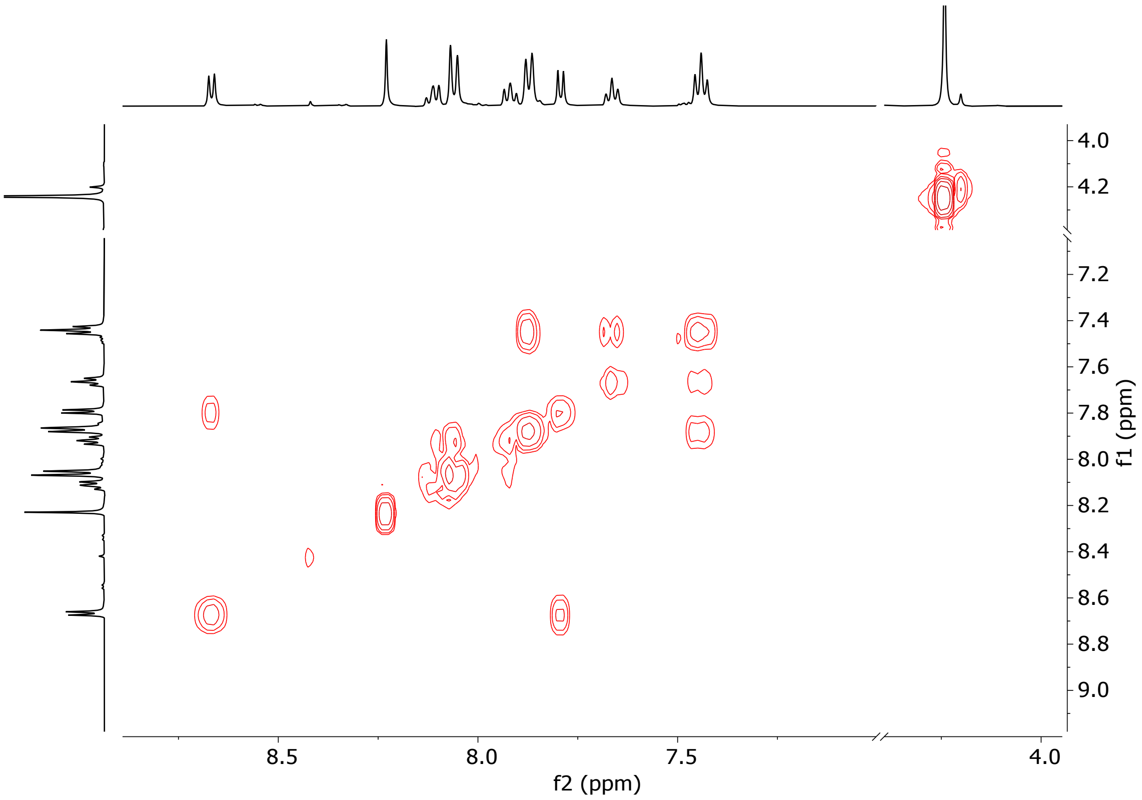

Figure S65. ^{1}H-^{1}H COSY (500 MHz, D_2O) partial spectrum of the 254 nm PSS for the compound **P**$_b$H·Cl.

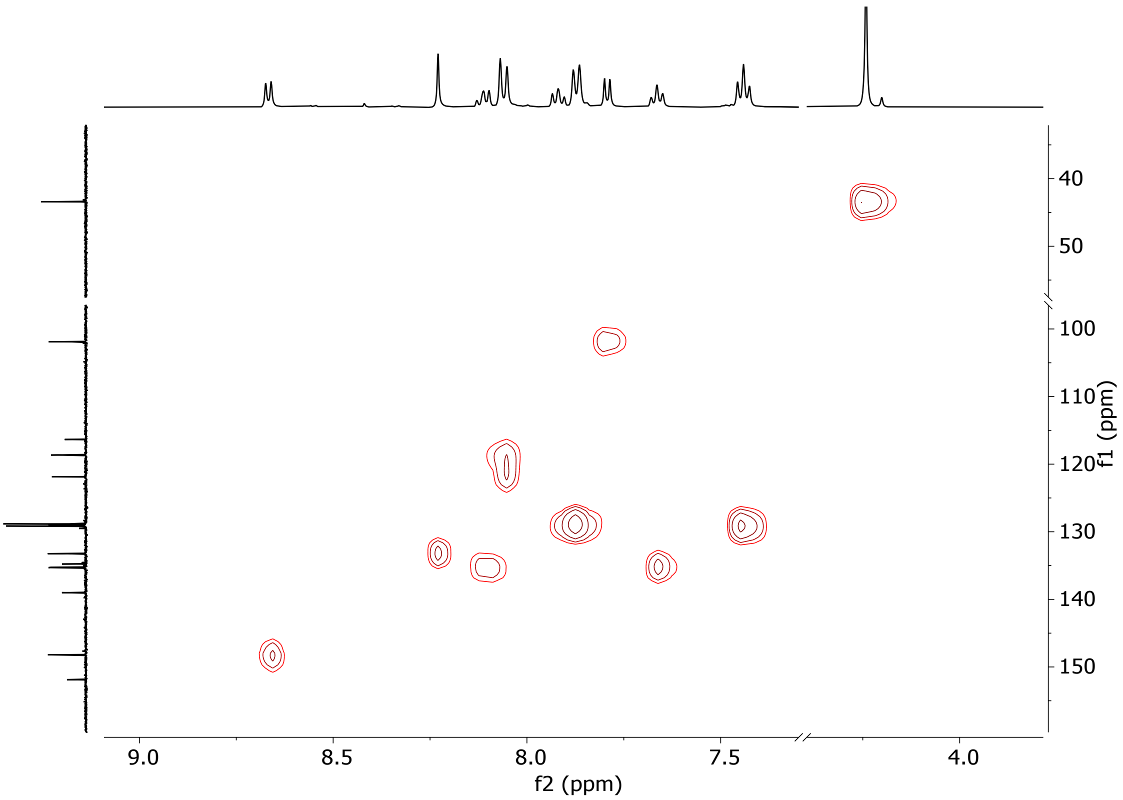

Figure S66. ^{1}H-^{13}C HSQC (500 MHz, D_2O) partial spectrum of the 254 nm PSS for the compound **P**$_b$H·Cl.

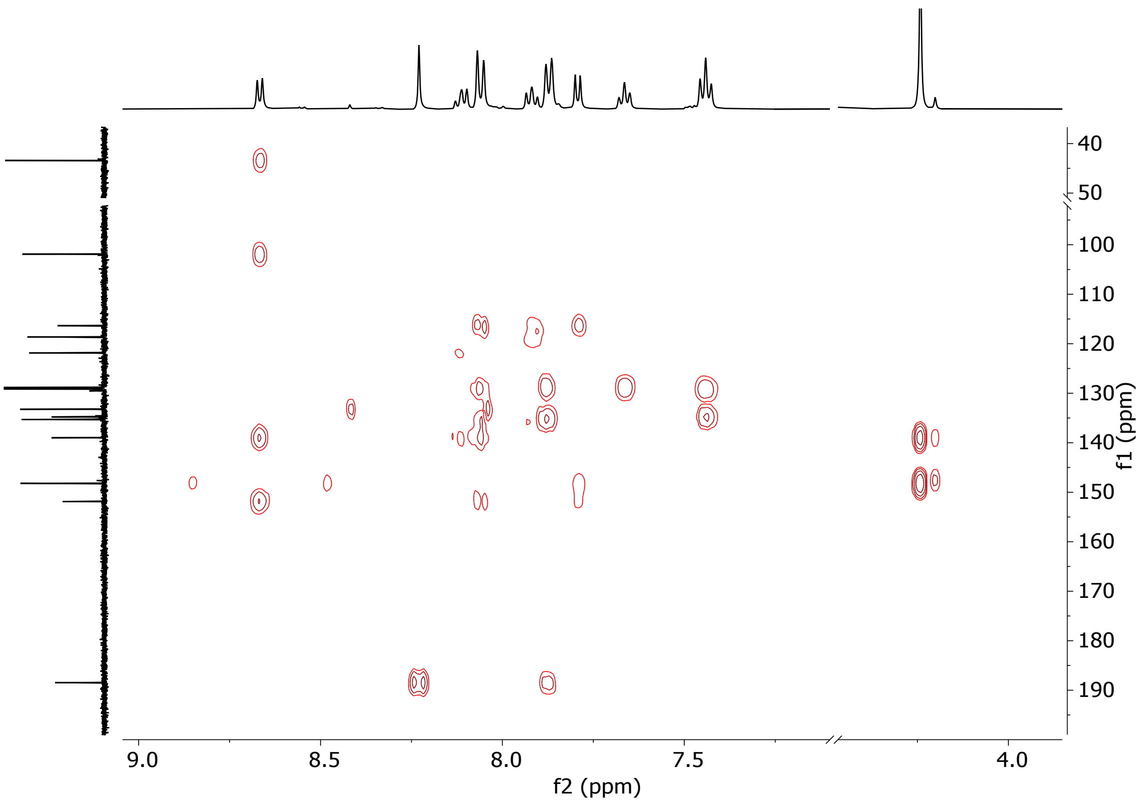

Figure S67. ^{1}H-^{13}C HMBC (500 MHz, D_2O) partial spectrum of the 254 nm PSS for the compound $\mathbf{P}_bH{\cdot}Cl$.

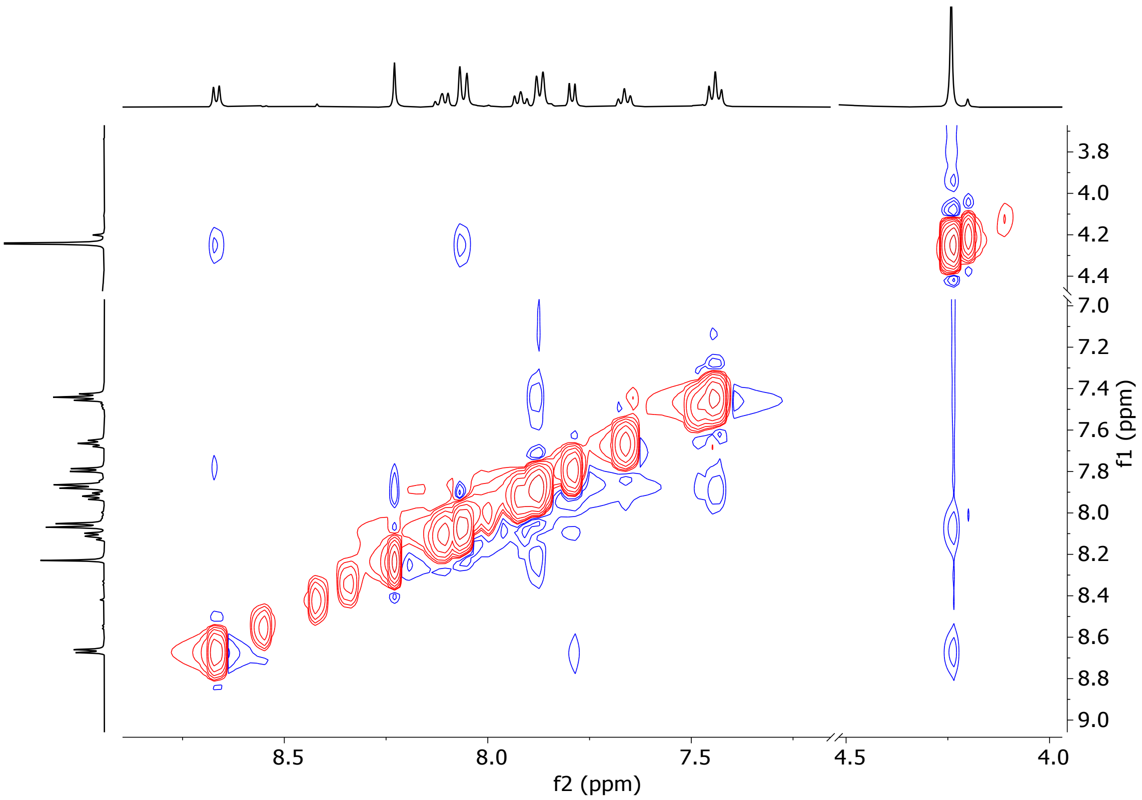

Figure S68. ^{1}H-^{1}H NOESY (500 MHz, D_2O) partial spectrum of the 254 nm PSS for the compound $\mathbf{P}_bH{\cdot}Cl$. Blue cross peaks indicate NOE correlations.

2.4.2.2. Organic medium.

P$_b$H·PF$_6$ salt was dissolved at 5 mM in CD_3CN. The NMR tube containing the compound solution was irradiated for 16 h at 254 nm, after which all NMR experiments were acquired.

^{1}H NMR (400 MHz, CD_3CN) δ (*ppm*): 15.28 (s, 1H), 8.70 (d, J = 7.0 Hz, 1H), 8.44 (s, 1H), 8.31 (dt, J = 8.7, 1.0 Hz, 1H), 8.28 – 8.21 (m, 4H), 8.06 (ddd, J = 8.2, 5.1, 3.0 Hz, 1H), 7.94 (d, J = 7.1 Hz, 1H), 7.86 – 7.79 (m, 1H), 7.74 – 7.65 (m, 2H), 4.35 (s, 3H).

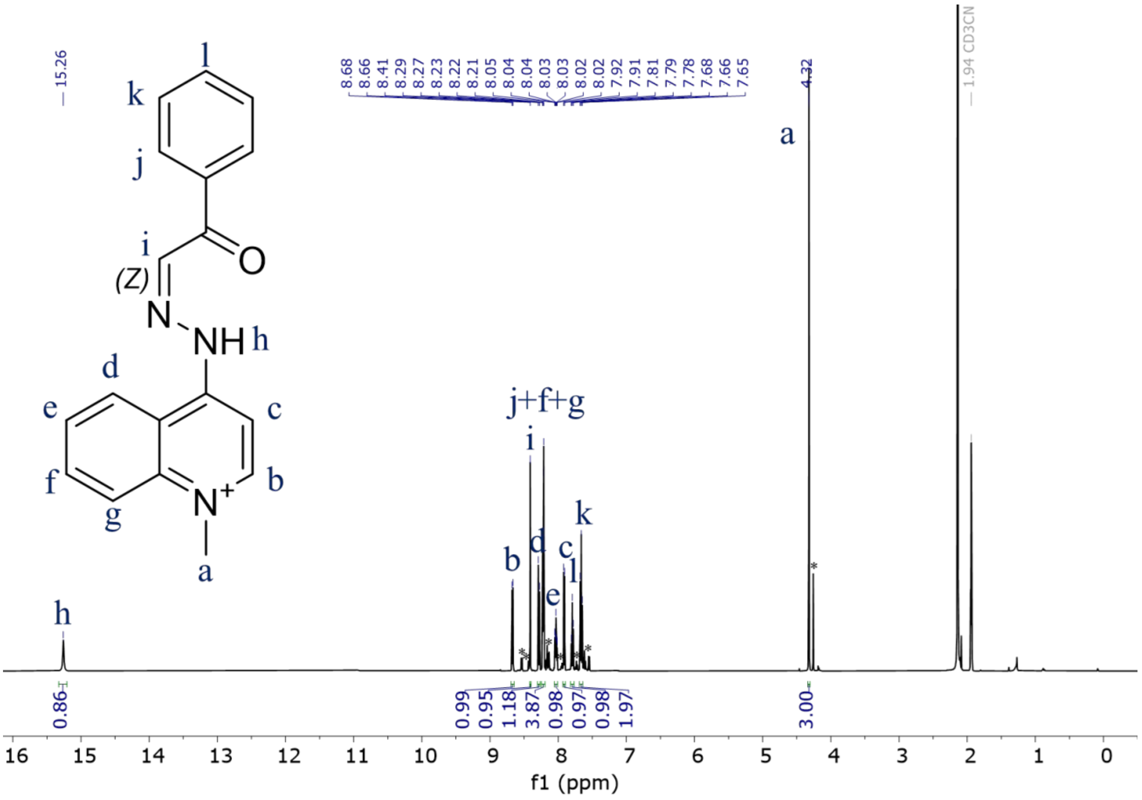

Figure S69. ^{1}H NMR (400 MHz, CD_3CN) spectrum of the 254 nm PSS for the compound **P**$_b$H·PF$_6$. Signals marked with an asterisk correspond to the *E*-isomer.

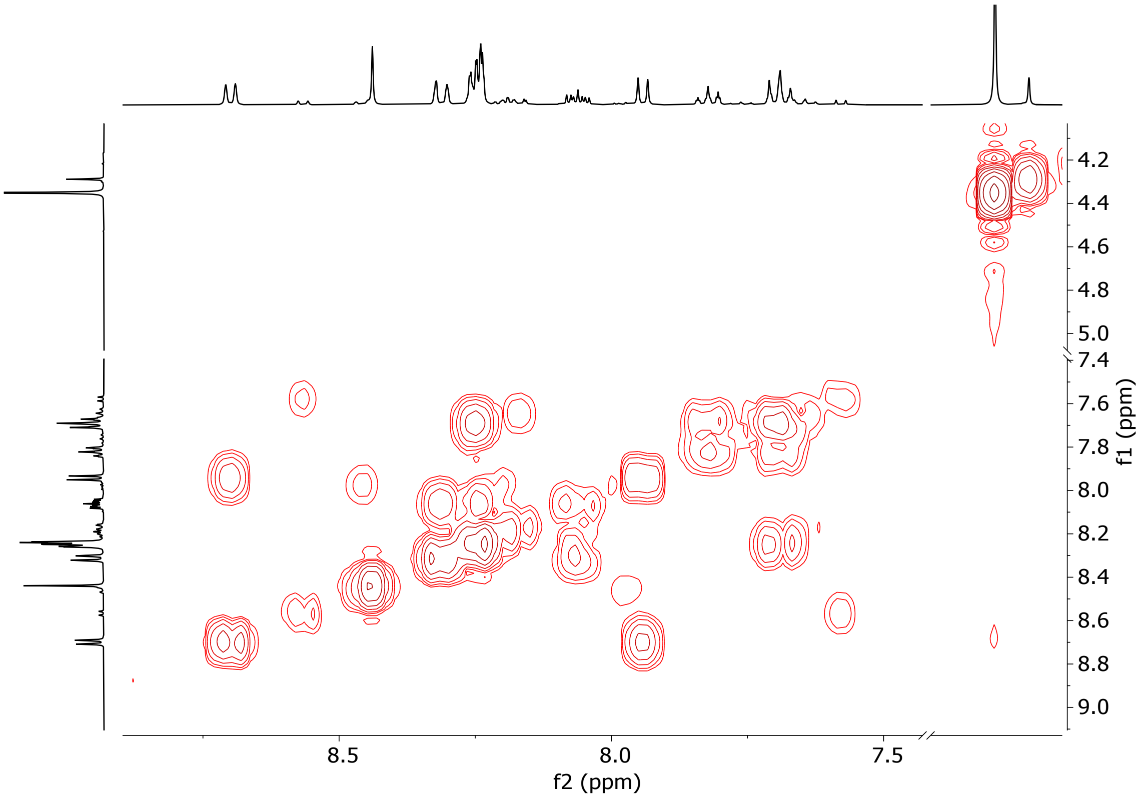

Figure S70. ^{1}H-^{1}H COSY (400 MHz, CD_3CN) partial spectrum of the 254 nm PSS for the compound **P**$_b$H·PF_6.

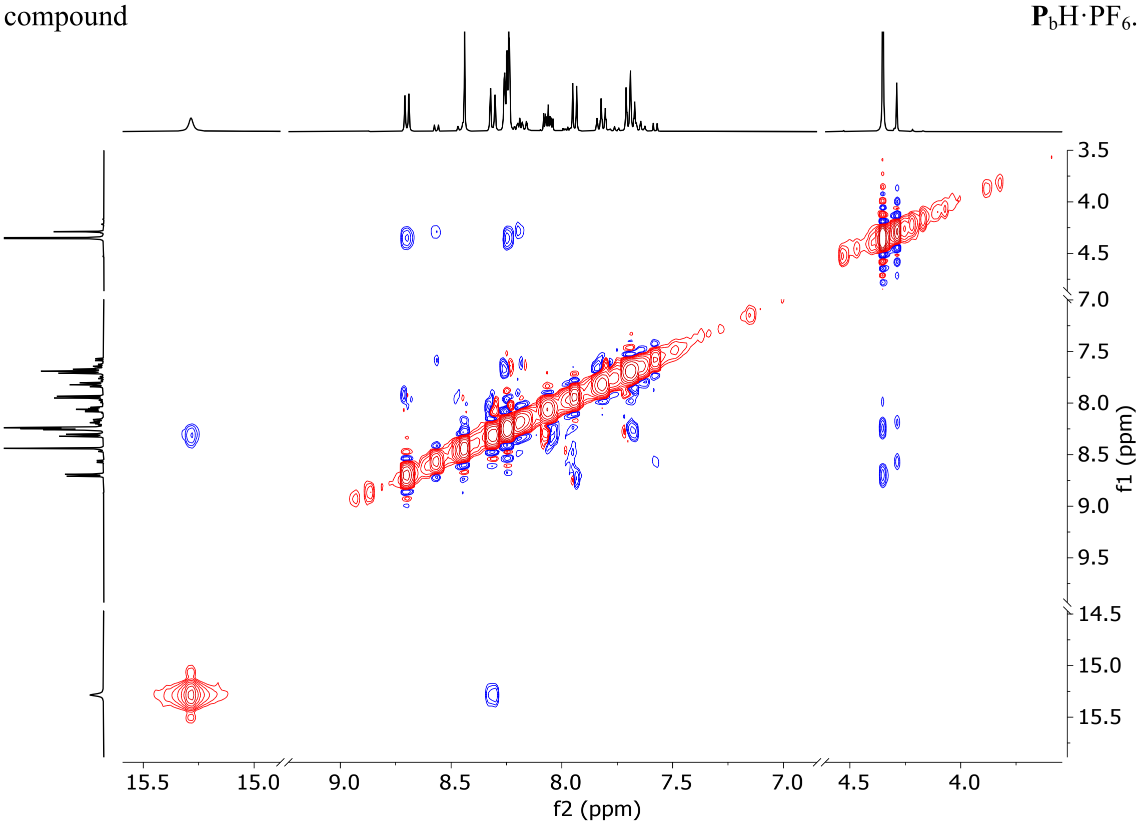

Figure S71. ^{1}H-^{1}H NOESY (400 MHz, CD_3CN) partial spectrum of the 254 nm PSS for the compound **P**$_b$H·PF_6. Blue cross peaks indicate NOE correlations. The absence of NOE coupling between NH and the imine signals is diagnostic of the *Z* isomer.

2.4.3. NMR data for the species assigned as *E*-P_b.

$P_bH \cdot Cl$ salt was dissolved at 0.5 mM in a D_2O phosphate buffer solution (20 mM, pD 13). Owing to the low solubility of the compound under basic conditions, the sample concentration was limited to 0.5 mM. Consequently, the number of scans for the ^1H NMR experiment was increased to 32.

^{1}H NMR (500 MHz, D_2O) δ (*ppm*): 8.55 (dd, *J* = 8.2, 1.4 Hz, 1H), 8.49 (s, 1H), 8.09 (dd, *J* = 8.4, 1.4 Hz, 2H), 7.99 – 7.92 (m, 2H), 7.83 (d, *J* = 8.6 Hz, 1H), 7.75 (t, *J* = 7.3 Hz, 1H), 7.68 – 7.58 (m, 3H), 7.19 (d, *J* = 7.5 Hz, 1H), 4.01 (s, 3H).

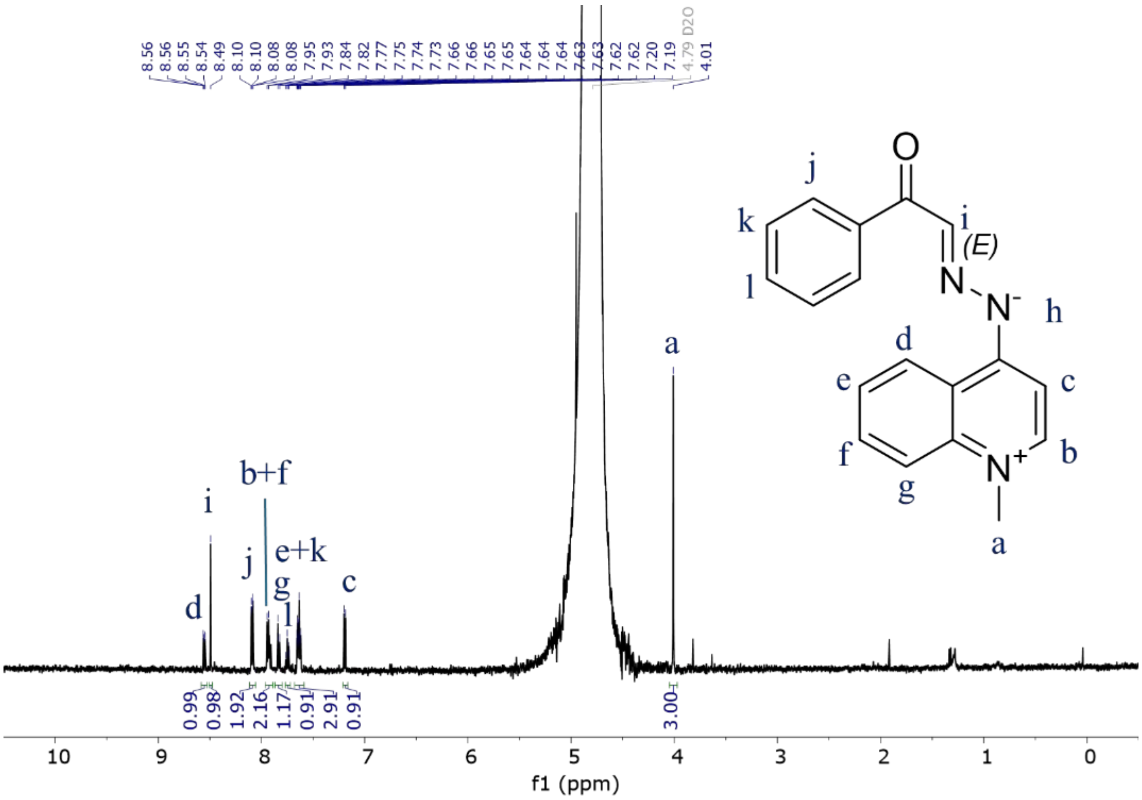

Figure S72. ^{1}H NMR (500 MHz, D_2O) spectrum of the compound **P_b**$H \cdot Cl$ as synthesized.

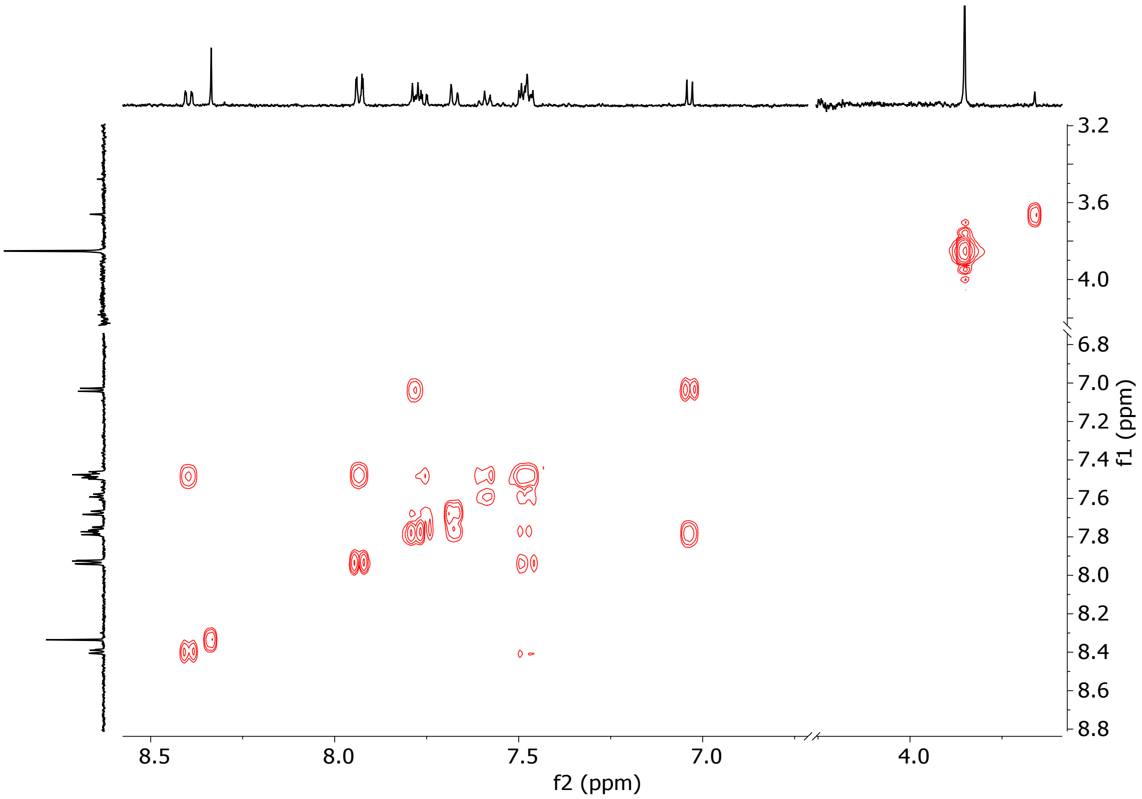

Figure S73. ^{1}H-^{1}H COSY (500 MHz, D_2O) partial spectrum of the compound **P**$_b$H·Cl as synthesized.

2.4.4. NMR data for the species assigned as *Z*-P_b.

$\mathbf{P_b}$H·Cl salt was dissolved at 5 mM in a D_2O phosphate buffer solution (20 mM, pD 5). The NMR tube containing the compound solution was irradiated for 16 h at 254 nm. Immediately before data acquisition, the sample was basified to pD 13 using phosphate buffer (40 mM), resulting in a final compound concentration of 0.5 mM. The interval between basification and acquisition was approximately 1 min. For the ^{1}H and COSY experiments, 16 and 64 scans were applied, with acquisition times of ~2 min and ~150 min, respectively.

^{1}H NMR (500 MHz, D_2O) δ (*ppm*): 7.94 (d, *J* = 7.7 Hz, 2H), 7.84 (s, 1H), 7.71 (d, *J* = 8.1 Hz, 1H), 7.69 – 7.61 (m, 3H), 7.58 (d, *J* = 8.6 Hz, 1H), 7.52 (t, *J* = 7.6 Hz, 2H), 7.25 (t, *J* = 7.8 Hz, 1H), 6.75 (d, *J* = 7.7 Hz, 1H), 3.82 (s, 3H).

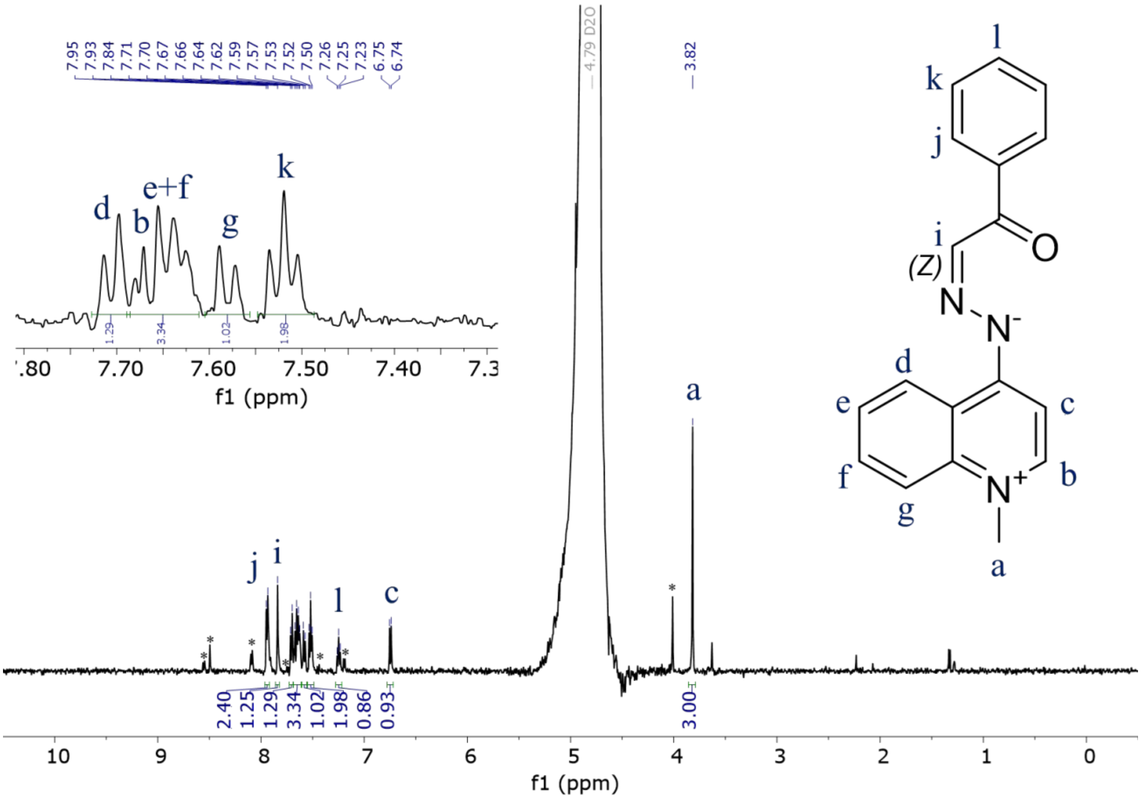

Figure S74. ^{1}H NMR (500 MHz, D_2O) spectrum of the basification after reaching the 254 nm PSS for the compound $\mathbf{P_b}$H·Cl. Signals marked with an asterisk correspond to the *E*-isomer. *Inset*: Partial region of the spectrum between 7.8-7.3 ppm.

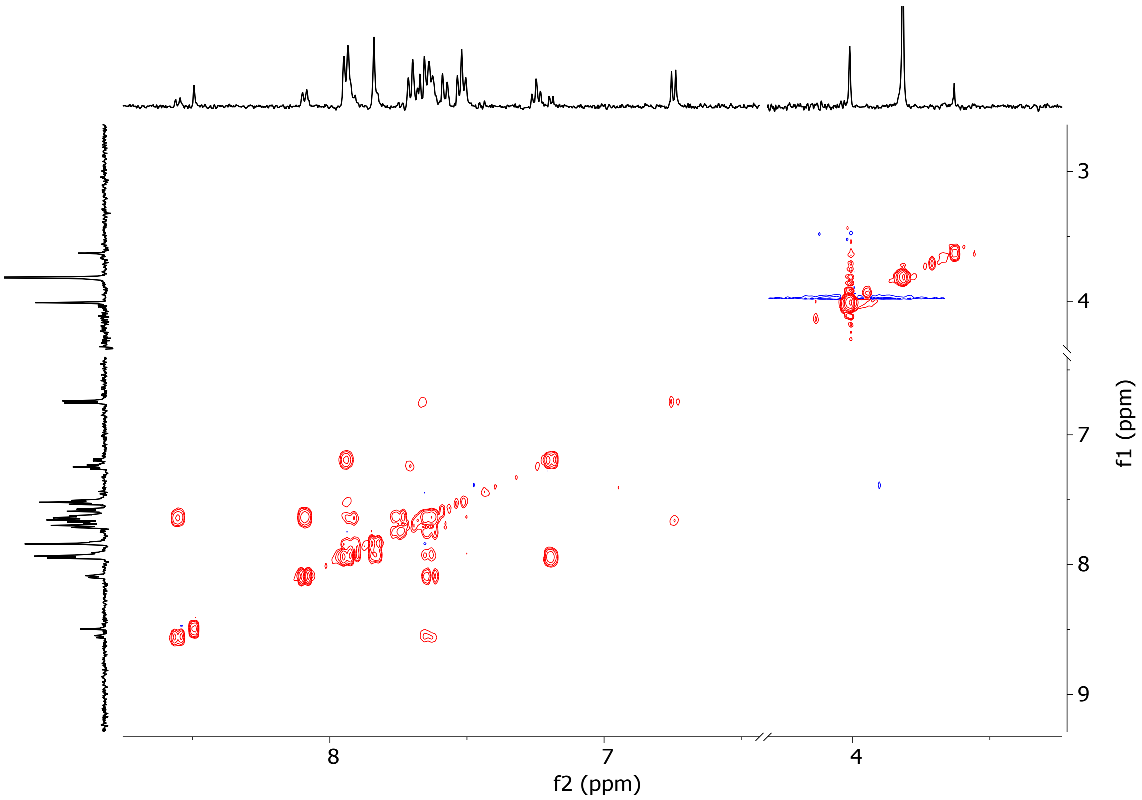

Figure S75. ^{1}H-^{1}H COSY (500 MHz, D_2O) spectrum of the basification after reaching the 254 nm PSS for the compound $\mathbf{P}_bH{\cdot}Cl$. Due to the acquisition time of the experiment and the fast thermal reversion, cross-peaks of *E*-isomer exhibit a greater intensity than observed in the ^{1}H NMR. Signals marked with an asterisk correspond to the *E*-isomer.

2.5 Synthesis and characterization data of H_c·Cl.

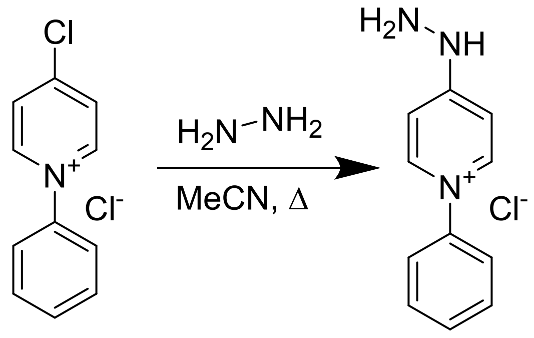

4-chloro-1-phenylpyridin-1-ium chloride (0.85 g, 3.8 mmol, 1 equiv) was dissolved in 80 mL of CH_3CN in a round bottom flask and heated at 120 ºC in a magnetic hot plate stirrer. Hydrazine hydrate (1.75 mL, 26.5 mmol, 7 equiv) was then added, and the reaction mixture was heated for 4h. Upon completion, a black solid was formed, which was filtered, and the remaining solution was concentrated under reduced pressure to afford **H_c**·Cl as a dark red sticky solid (759 mg, 92%) without further purification.

^{1}H NMR (300 MHz, DMSO-d$_6$) δ (*ppm*): 8.34 (d, J = 7.7 Hz, 1H), 8.17 (d, J = 7.6 Hz, 1H), 7.61 (d, J = 4.3 Hz, 4H), 7.57 – 7.44 (m, 2H), 7.06 (d, J = 8.1 Hz, 1H), 6.78 (d, J = 7.8 Hz, 1H), 5.73 (s, 2H).

^{13}C{^{1}H} NMR (75 MHz, DMSO-d$_6$) δ (*ppm*): 157.7 (C_{Ar}), 142.5 (CH_{Ar}), 142.1 (C_{Ar}), 140.3 (CH_{Ar}), 130.1 (CH_{Ar}), 129.1 (CH_{Ar}), 123.3 (CH_{Ar}), 107.5 (CH_{Ar}) 104.9 (CH_{Ar})

HRMS (ESI) m/z: Calcd for $C_{11}H_{12}N_3^+$ 186.1026; Found 186.1028.

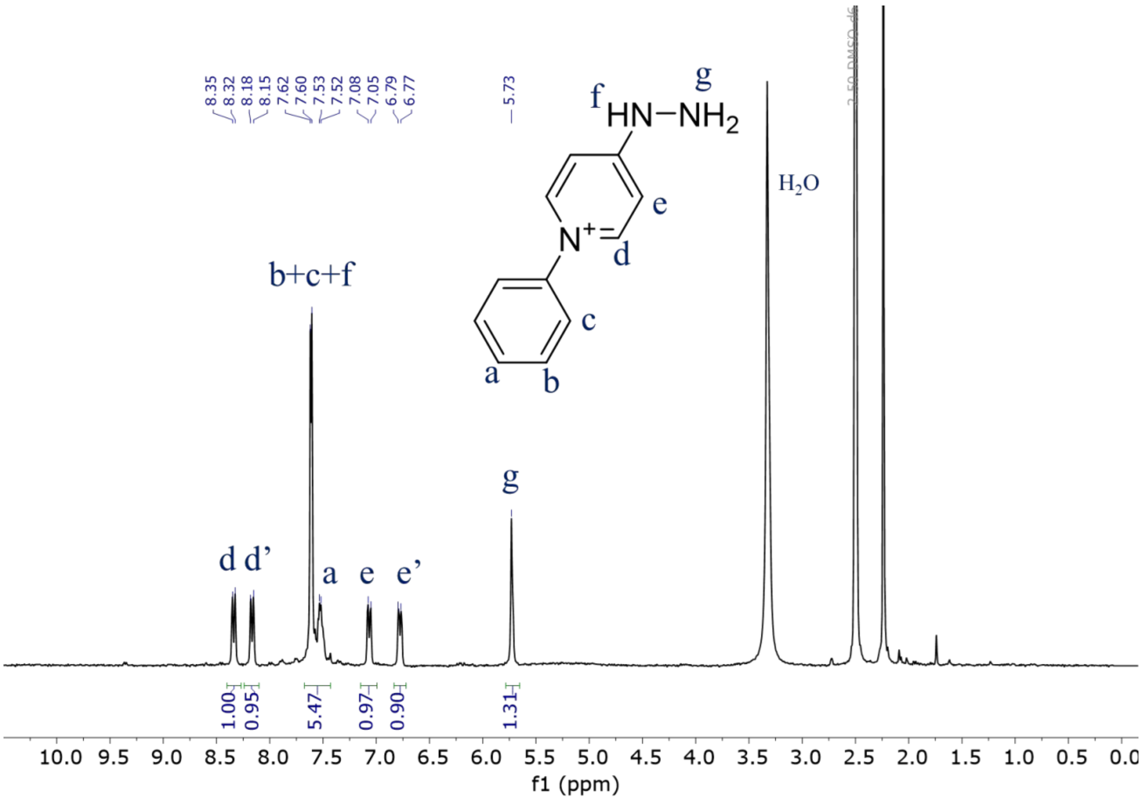

Figure S76. ^{1}H NMR (300 MHz, DMSO-d$_6$) spectrum of $\mathbf{H}_c$·Cl.

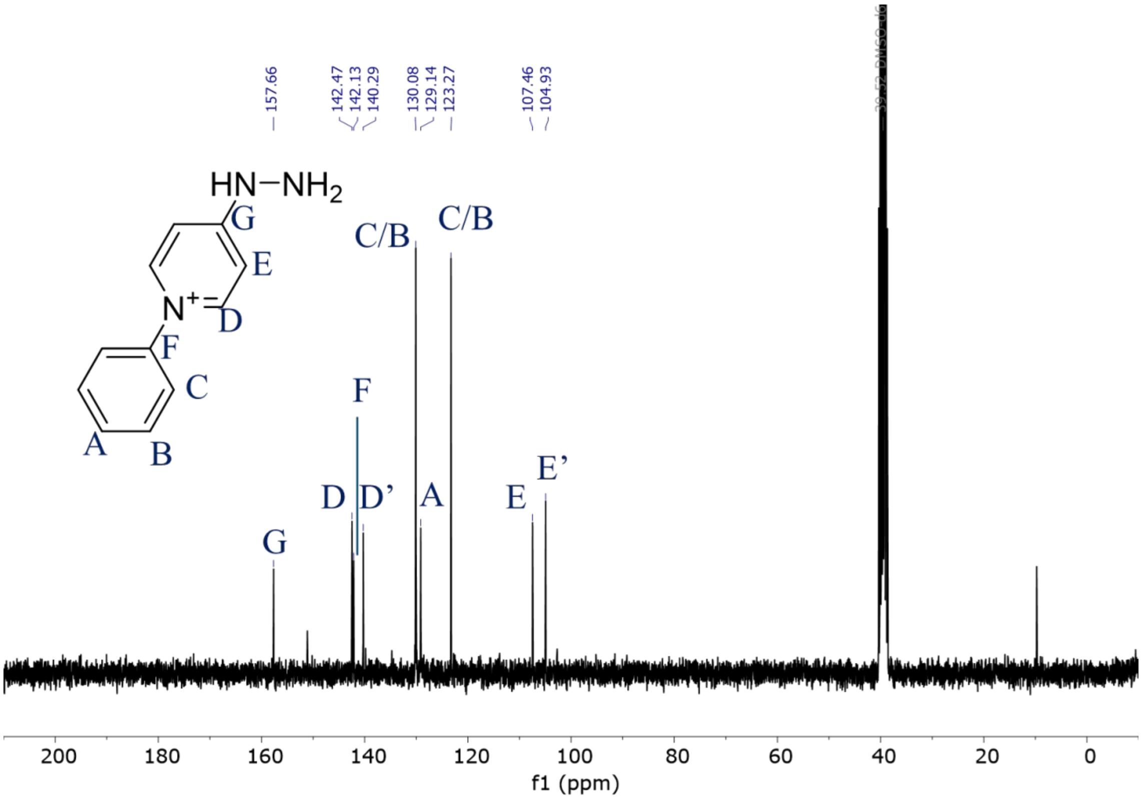

Figure S77. $^{13}C\{^{1}H\}$ NMR (75 MHz, DMSO-d$_6$) spectrum of $\mathbf{H}_c$·Cl.

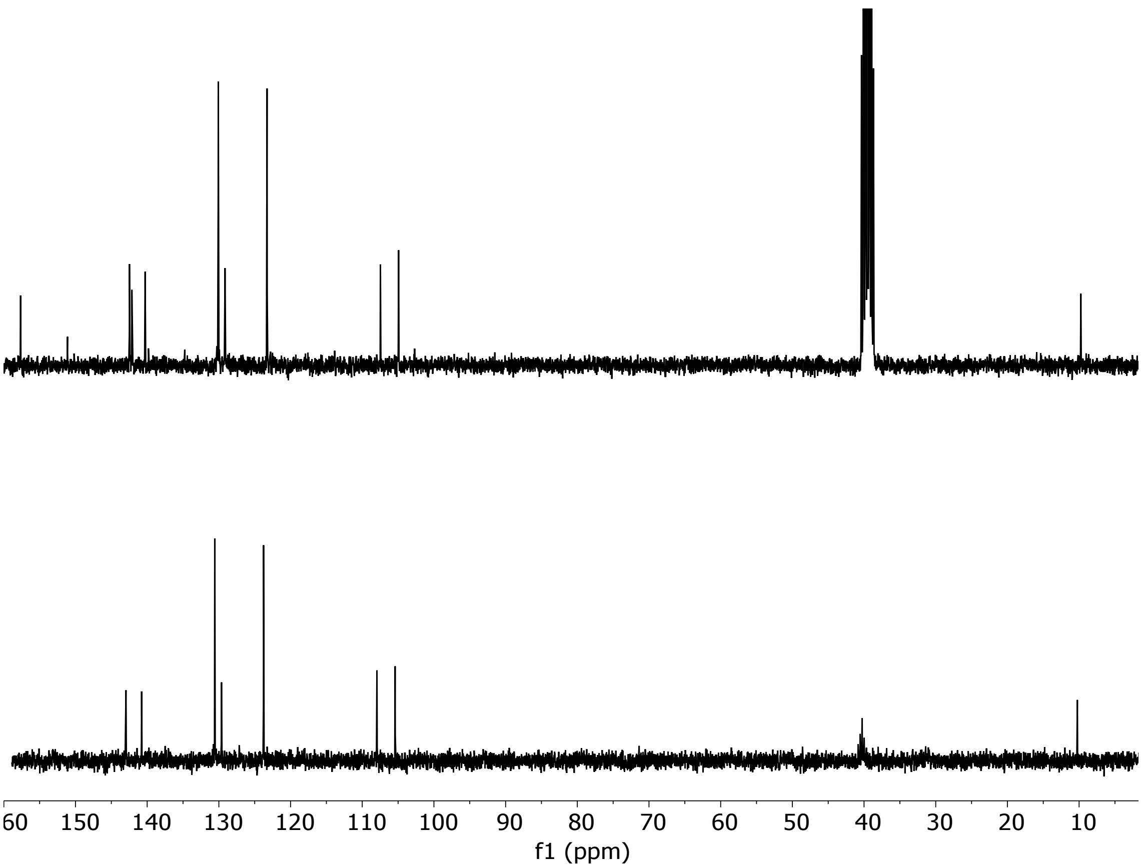

Figure S78. DEPT-135 NMR (75 MHz, DMSO-d_6) and $^{13}C\{^{1}H\}$ NMR (75 MHz, DMSO-d_6) stacked spectra of $\mathbf{H}_c$·Cl.

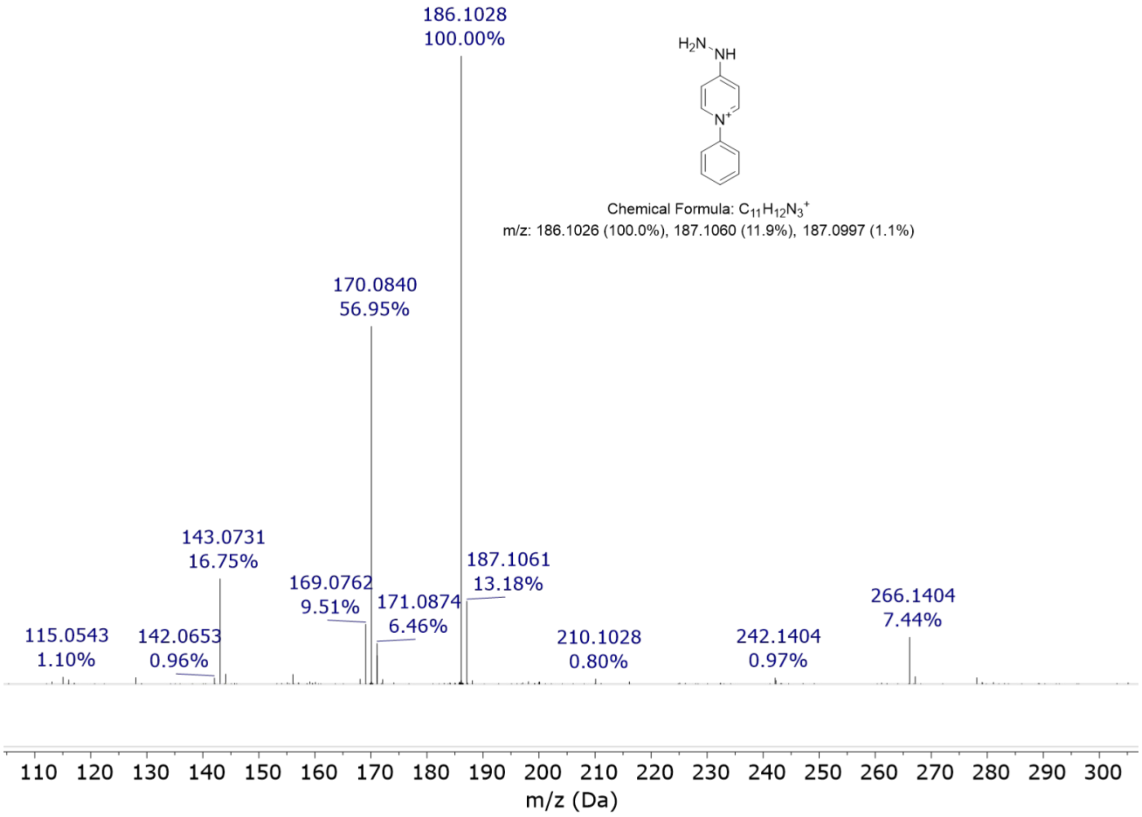

Figure S79. HRMS-ESI spectrum of $\mathbf{H}_c$·Cl.

2.6 Synthesis and characterization data of $P_cH{\cdot}Cl/PF_6$.

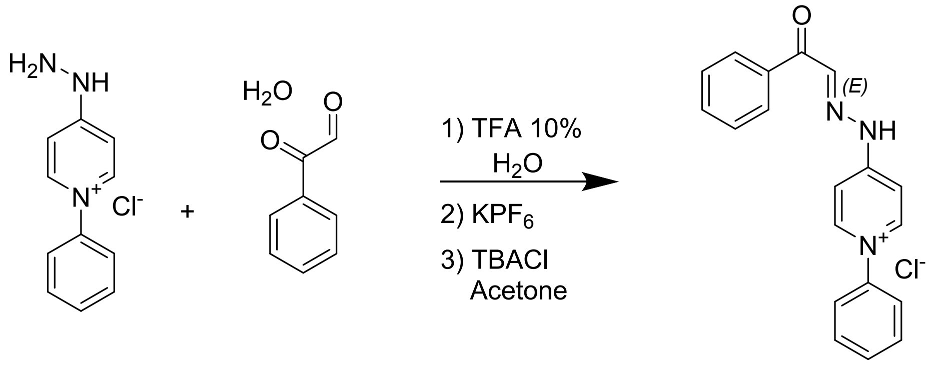

H$_c$·Cl (732 mg, 2.8 mmol, 1 eq) was dissolved in a round bottom flask in 200 mL of H_2O. Phenylglyoxal (474 mg, 3.1 mmol, 1.1 eq) and TFA (22 µL, 0.3 mmol, 0.1 eq) were then added, and the mixture was heated in a magnetic hot plate stirrer at 60ºC for 4h in the absence of light. Upon completion, KPF_6 (1.0 g, 2eq) was added to the mixture and the resulting precipitate was filtered, washed with H_2O (2 x 10 mL) and Et_2O (2 x 10 mL) and dried under vacuum to yield the product **P**$_c$H·PF_6 as a yellow solid (647 mg, 51%). The solid was dissolved in acetone and a saturated solution of TBACl in acetone was added. After 1 h under stirring, the precipitate was filtered and washed with acetone (2 x 10 mL) and Et_2O (2 x 10 mL), and dried under vacuum to yield **P**$_c$H·Cl as a yellow solid (406 mg, 32%).

HRMS (ESI) m/z: Calcd for $C_{19}H_{16}N_3O^+$ 302.1288; found 302.1280.

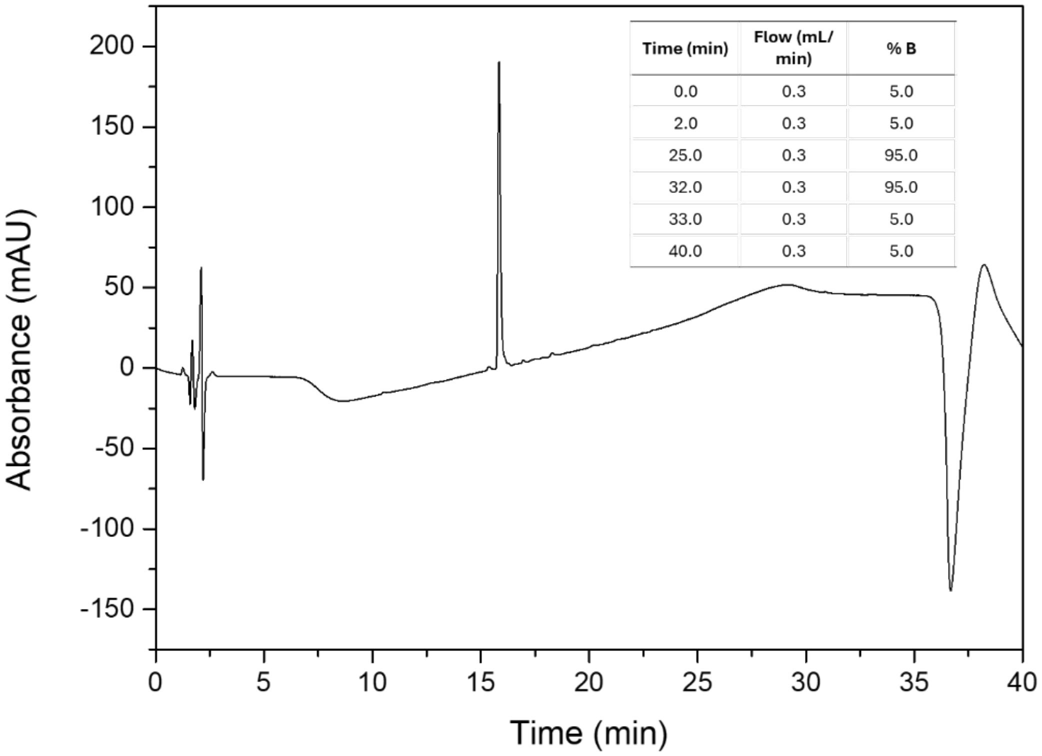

Figure S80. HPLC chromatogram (220 nm) of **P**$_c$H·Cl at t_R = 15.84 min (Inset: separation method; A = H_2O + 0.04 % TFA, B = CH_3CN + 0.04 % TFA).

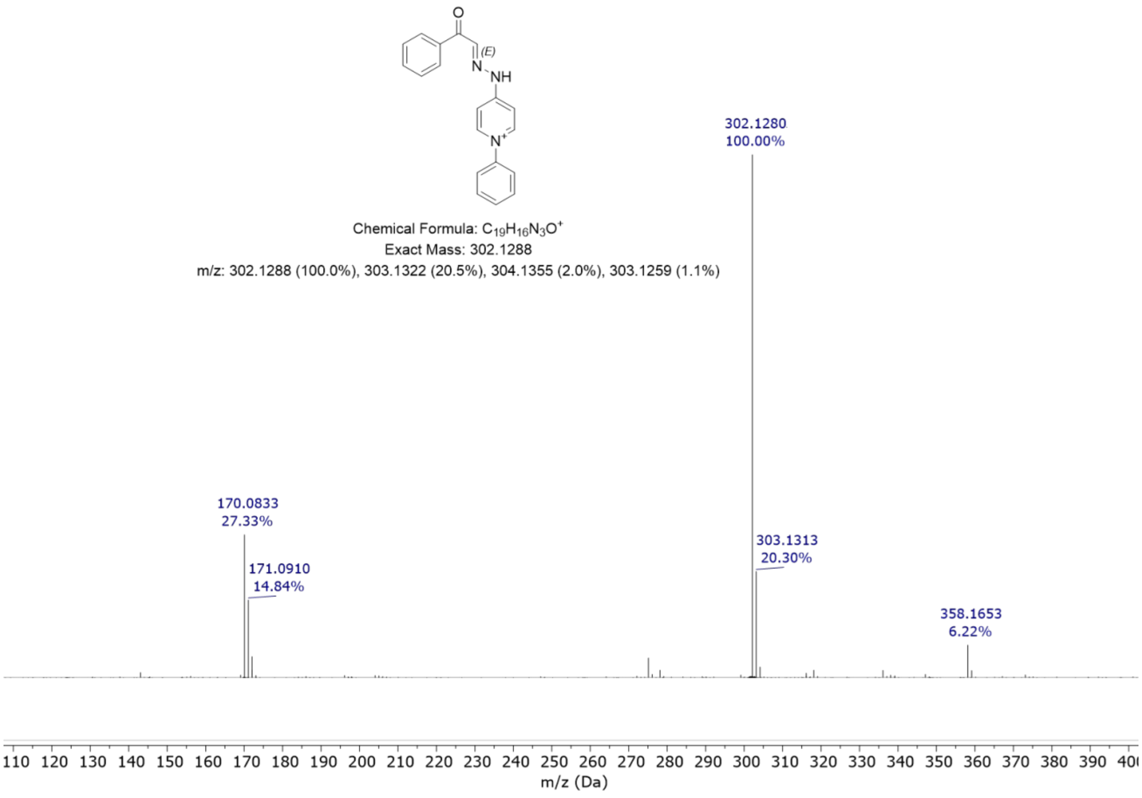

Figure S81. HRMS-ESI spectrum of **P**$_c$H·Cl.

2.6.1. NMR data for the species assigned as *E*-P_cH^+.

2.6.1.1. Aqueous medium.

P$_c$H·Cl salt was dissolved at 5 mM in a D_2O phosphate buffer solution (20 mM, pD 6).

^{1}H NMR (500 MHz, D_2O) δ (*ppm*): 8.59 (d, J = 7.3 Hz, 2H), 8.26 (s, 1H), 8.08 (d, J = 7.8 Hz, 2H), 7.76 (t, J = 7.4 Hz, 2H), 7.71 – 7.5 (m, 6H), 7.53 – 7.26 (m, 2H).

^{13}C{^{1}H} NMR (126 MHz, D_2O) δ (*ppm*): 191.2 (C=O), 155.2 (C_{Ar}), 143.6 (CH_{Ar}), 142.7 (CH=N), 142.1 (C_{Ar}), 135.5 (C_{Ar}), 134.4 (CH_{Ar}), 130.5 (CH_{Ar}), 130.3 (CH_{Ar}), 129.8 (CH_{Ar}), 128.8 (CH_{Ar}), 123.5 (CH_{Ar}), 109.5 (CH_{Ar}).

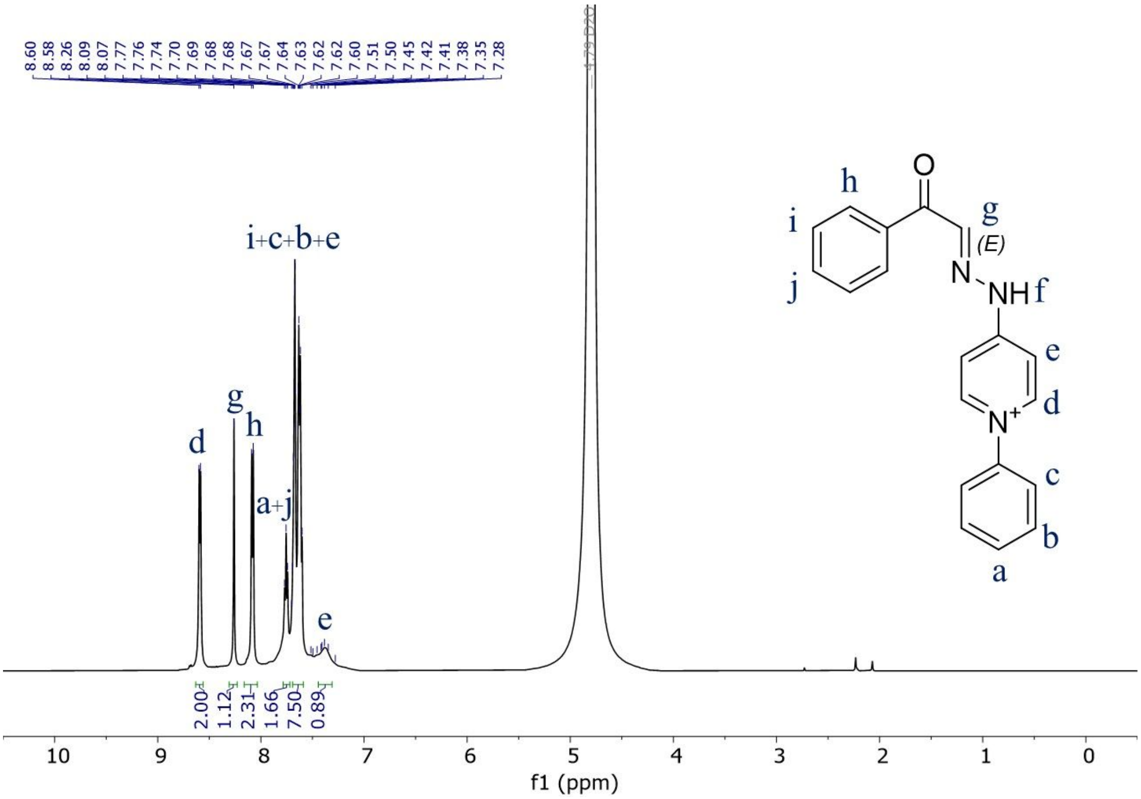

Figure S82. ^{1}H NMR (500 MHz, D_2O) spectrum of the compound **P**$_c$H·Cl as synthesized.

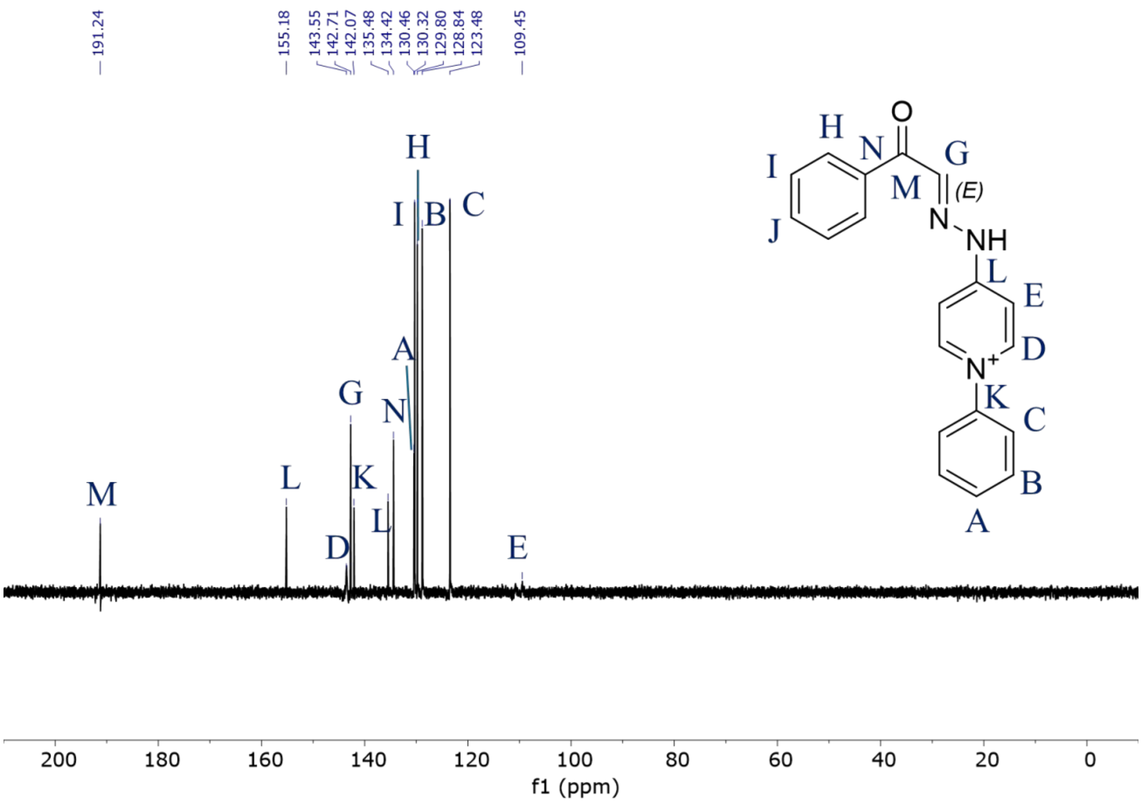

Figure S83. $^{13}C\{^{1}H\}$ NMR (126 MHz, D_2O) spectrum of the compound $\mathbf{P}_c$H·Cl as synthesized.

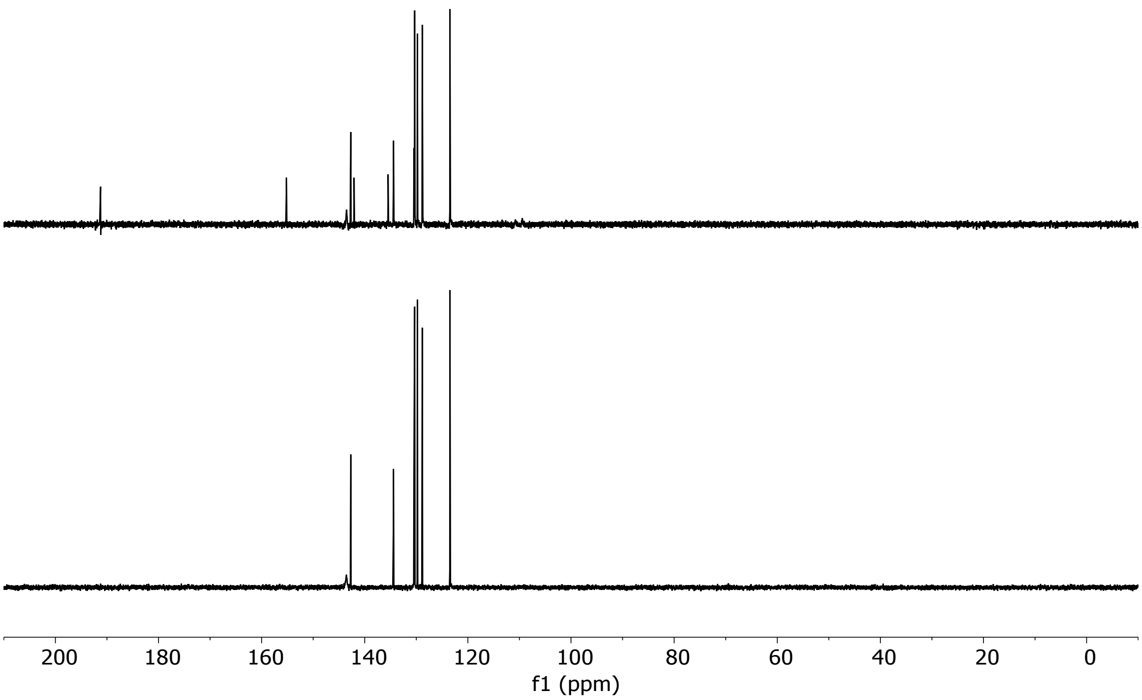

Figure S84. DEPT-135 NMR (126 MHz, D_2O) and $^{13}C\{^{1}H\}$ NMR (126 MHz, D_2O) stacked spectra of the compound $\mathbf{P}_c$H·Cl as synthesized.

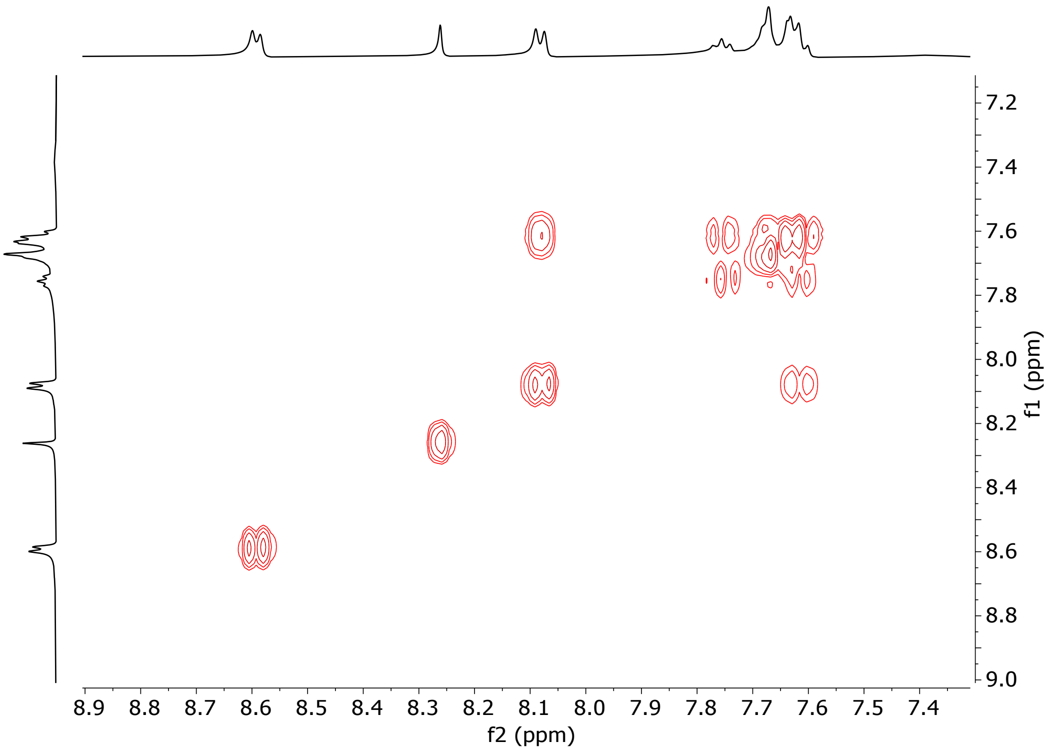

Figure S85. ^{1}H-^{1}H COSY (500 MHz, D_2O) partial spectrum of the compound $\mathbf{P}_c$H·Cl as synthesized.

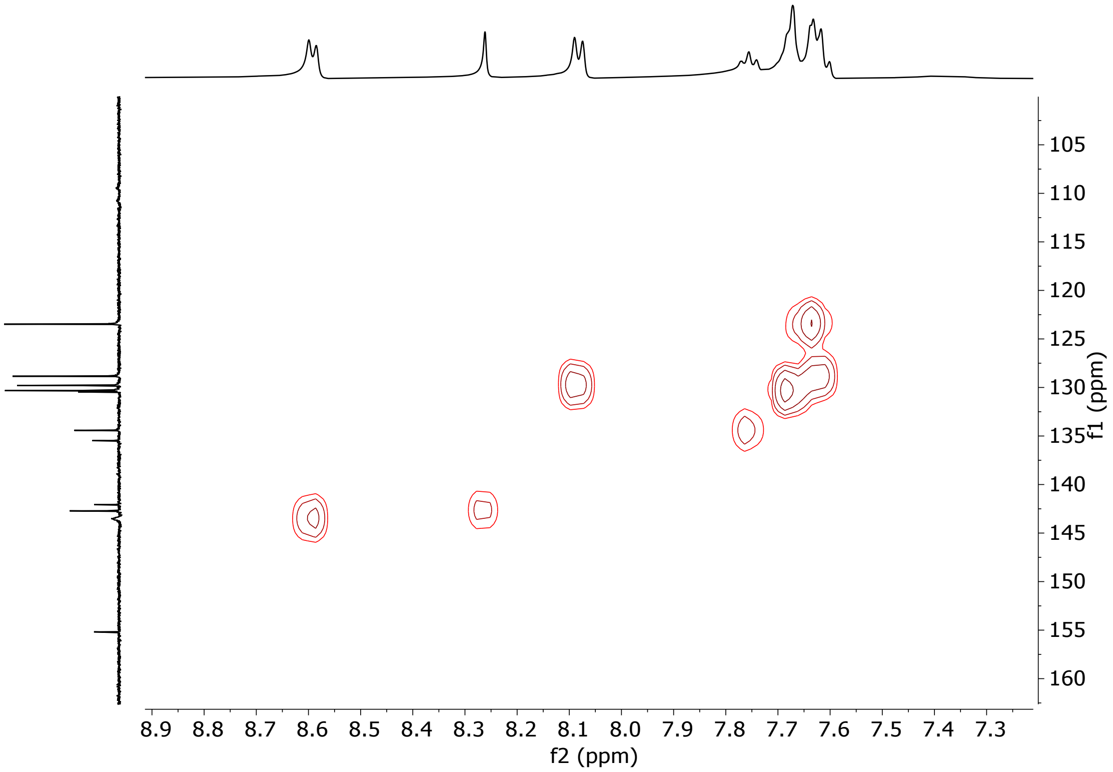

Figure S86. ^{1}H-^{13}C HSQC (500 MHz, D_2O) partial spectrum of the compound $\mathbf{P}_c$H·Cl as synthesized.

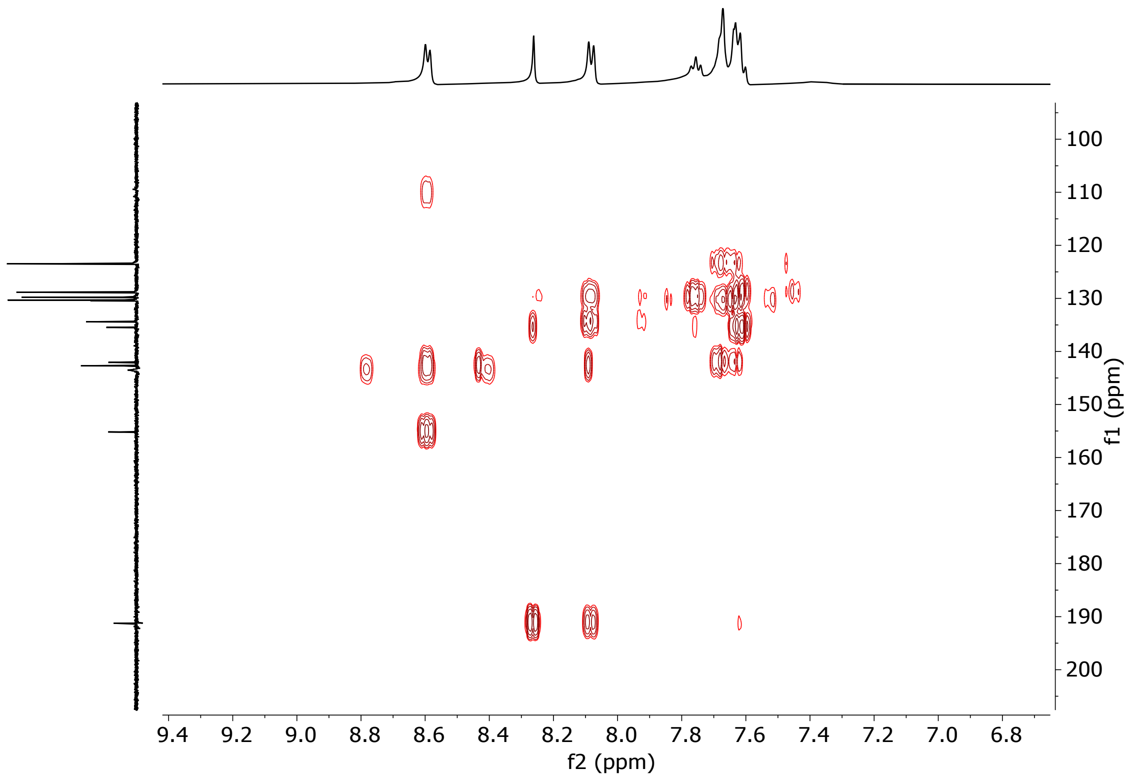

Figure S87. ^{1}H-^{13}C HMBC (500 MHz, D_2O) partial spectrum of the compound $\mathbf{P}_c$H·Cl as synthesized.

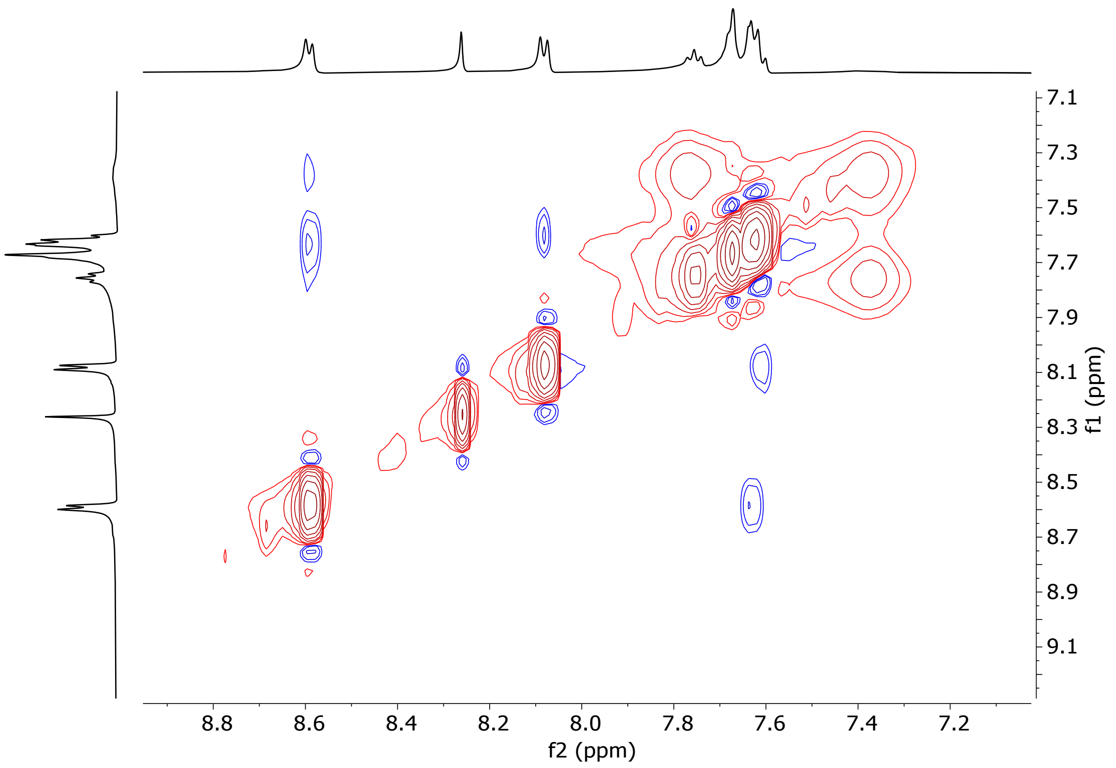

Figure S88. ^{1}H-^{1}H NOESY (500 MHz, D_2O) partial spectrum of the compound $\mathbf{P}_c$H·Cl as synthesized.

2.6.1.2. Organic medium.

P$_c$H·PF$_6$ salt was dissolved at 5 mM in CD_3CN.

^{1}H NMR (400 MHz, CD_3CN) δ (*ppm*): 11.31 – 10.33 (m, 1H), 8.45 (d, J = 7.2 Hz, 2H), 8.10 (dd, J = 8.4, 1.3 Hz, 2H), 7.98 (s, 1H), 7.71 (t, J = 7.5 Hz, 1H), 7.68 – 7.64 (m, 3H), 7.62 – 7.55 (m, 5H), 7.42 – 7.25 (m, 1H).

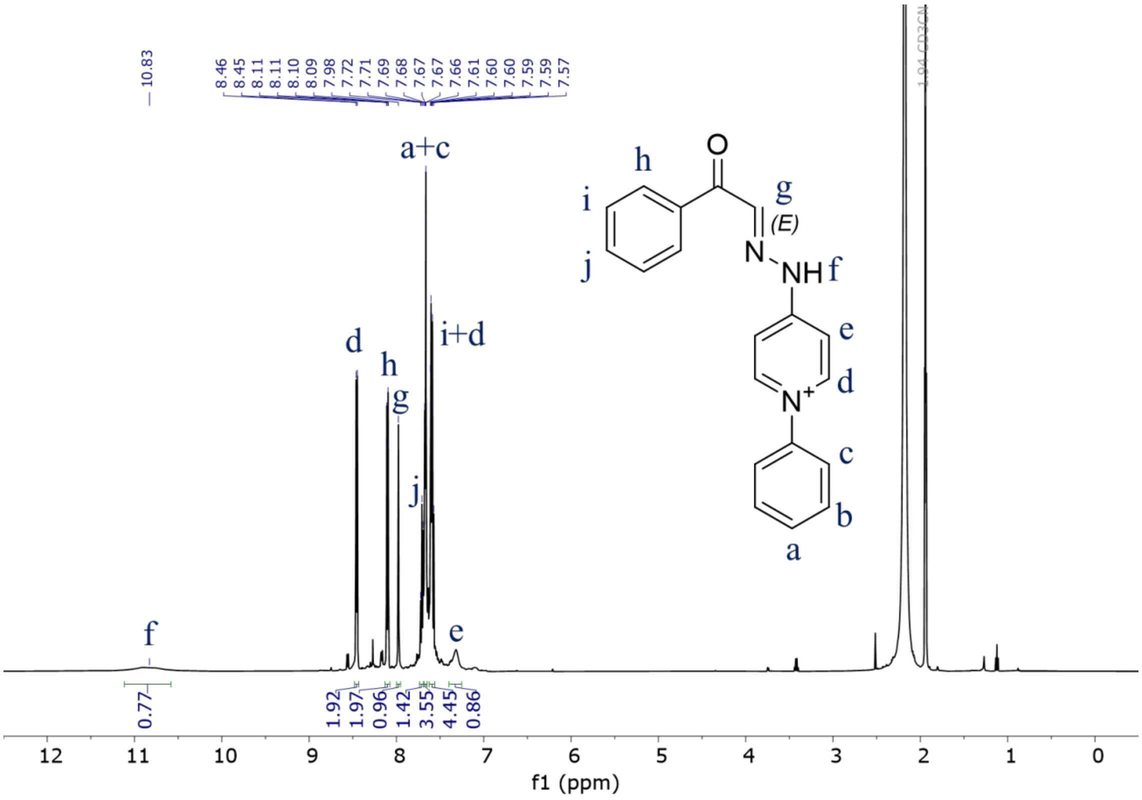

Figure S89. ^{1}H NMR (400 MHz, CD_3CN) spectrum of the compound **P**$_c$H·PF$_6$ as synthesized.

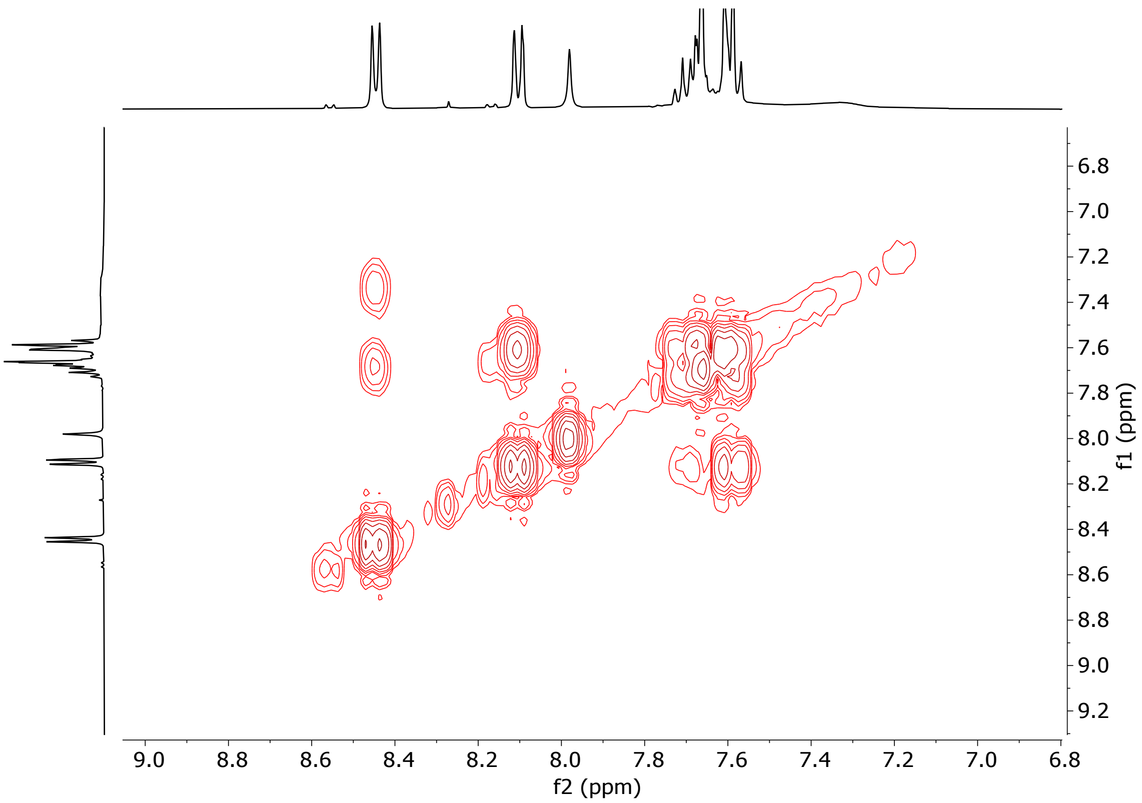

Figure S90. ^{1}H-^{1}H COSY (400 MHz, CD_3CN) partial spectrum of the compound $\mathbf{P}_c$H·PF_6 as synthesized.

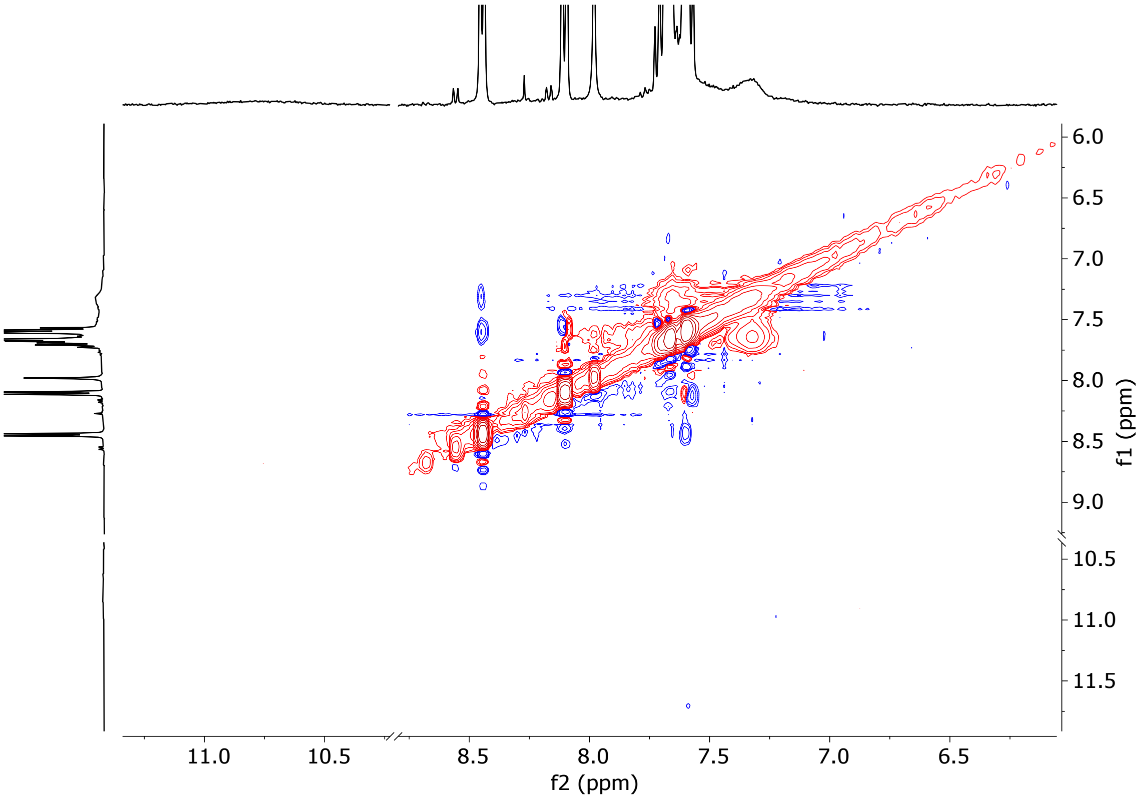

Figure S91. ^{1}H-^{1}H NOESY (400 MHz, CD_3CN) partial spectrum of the compound $\mathbf{P}_c$H·PF_6 as synthesized. Blue cross peaks indicate NOE correlations. Due to the coalescence of the NH signal, NOE is not observed with the imine, however the NH chemical shift is diagnostic of the *E*-isomer.

2.6.2 NMR data for the species assigned as *Z*-P_cH^+.

2.6.2.1. Aqueous medium.

P_cH·Cl salt was dissolved at 5 mM in a D_2O phosphate buffer solution (20 mM, pD 6). The NMR tube containing the compound solution was irradiated for 16 h at 254 nm, after which all NMR experiments were acquired.

^{1}H NMR (500 MHz, D_2O) δ (*ppm*): 8.71 (d, *J* = 7.2 Hz, 2H), 8.29 (s, 1H), 8.16 (d, *J* = 7.4 Hz, 2H), 7.90 – 7.84 (m, 2H), 7.78 (t, *J* = 7.5 Hz, 1H), 7.71 – 7.63 (m, 7H).

^{13}C{^{1}H} NMR (126 MHz, D_2O) δ (*ppm*): 188.5 (C=O), 155.4 (C_{Ar}), 143.9 (CH_{Ar}), 142.2 (C_{Ar}), 135.7 (C_{Ar}), 135.0 (CH_{Ar}), 133.2 (CH=N), 130.6 (CH_{Ar}), 130.3 (CH_{Ar}), 129.2 (CH_{Ar}), 129.0 (CH_{Ar}), 123.6 (CH_{Ar}) 110.9 (CH_{Ar}).

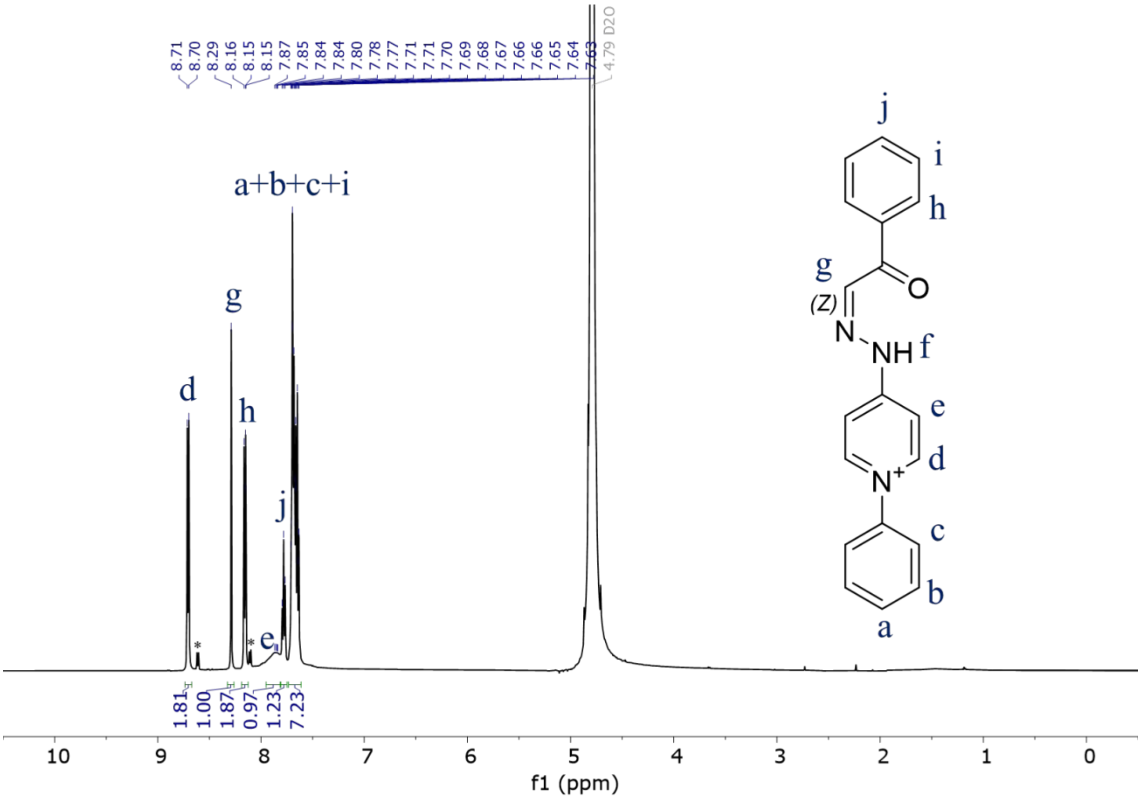

Figure S92. ^{1}H NMR (500 MHz, D_2O) spectrum of the 254 nm PSS for the compound **P_c**H·Cl. Signals marked with an asterisk correspond to the *E*-isomer.

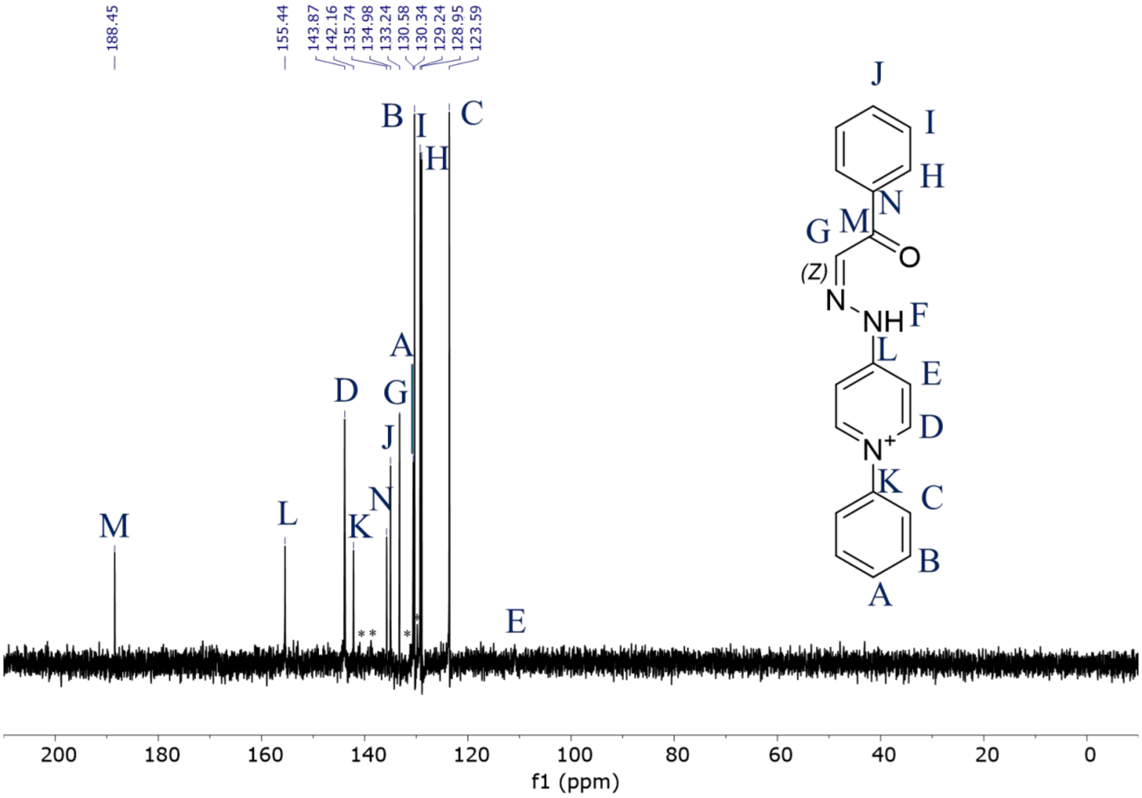

Figure S93. $^{13}C\{^{1}H\}$ NMR (126 MHz, D_2O) spectrum of the 254 nm PSS for the compound $\mathbf{P}_c$H·Cl. Signals marked with an asterisk correspond to the *E*-isomer.

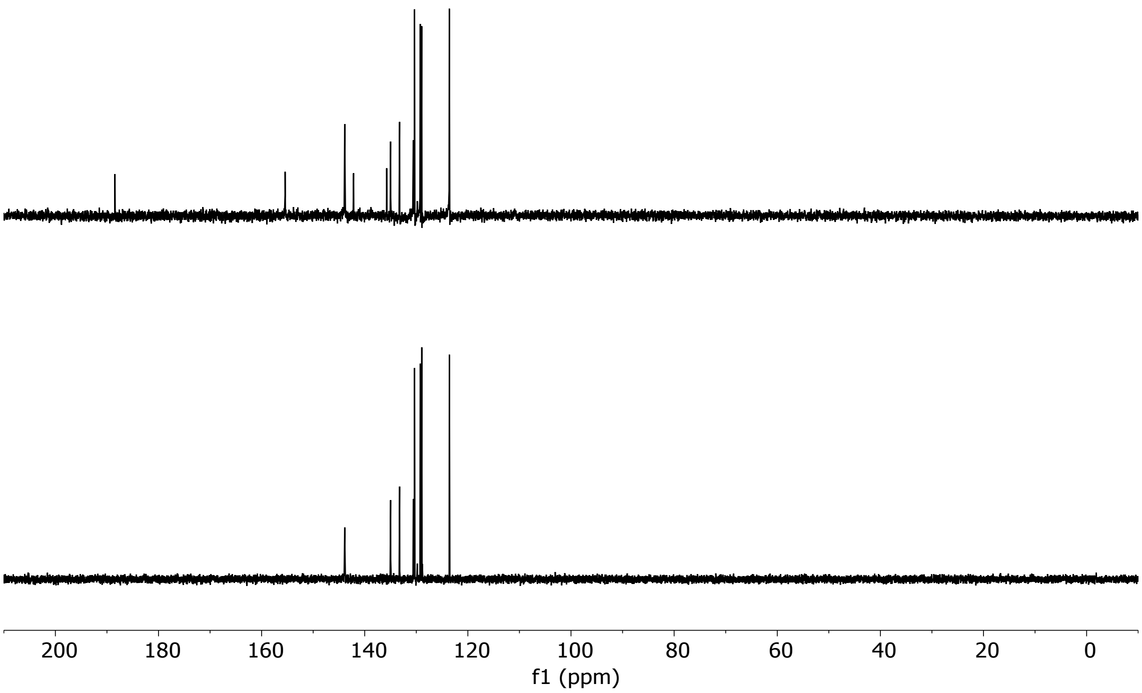

Figure S94. DEPT-135 NMR (126 MHz, D_2O) and $^{13}C\{^{1}H\}$ NMR (126 MHz, D_2O) stacked spectra of the 254 nm PSS for the compound $\mathbf{P}_c$H·Cl.

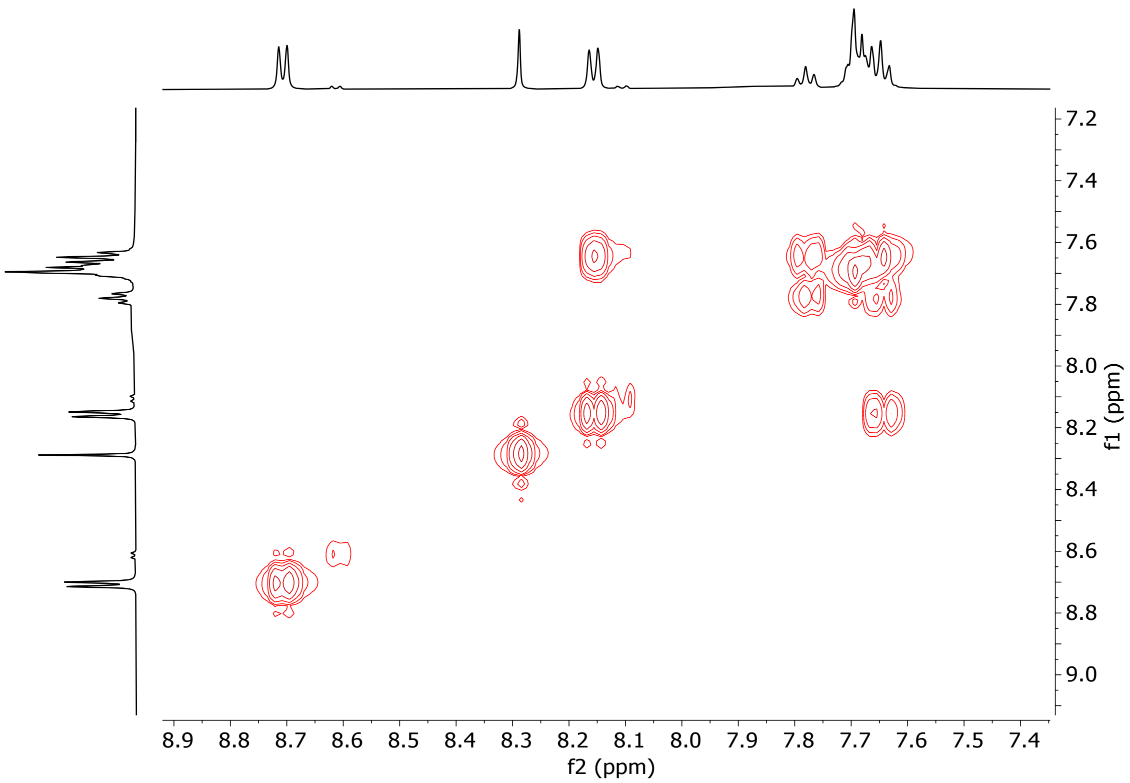

Figure S95. ^{1}H-^{1}H COSY (500 MHz, D_2O) partial spectrum of the 254 nm PSS for the compound **P**$_c$H·Cl.

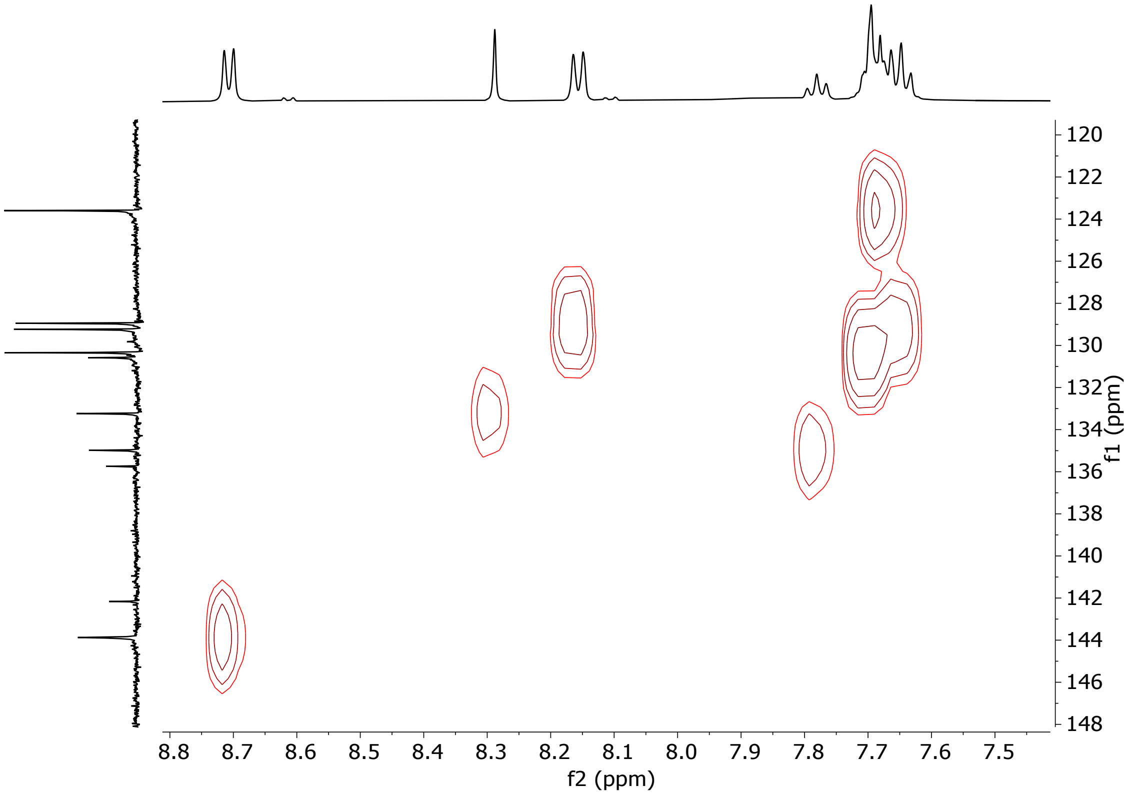

Figure S96. ^{1}H-^{13}C HSQC (500 MHz, D_2O) partial spectrum of the 254 nm PSS for the compound **P**$_c$H·Cl.

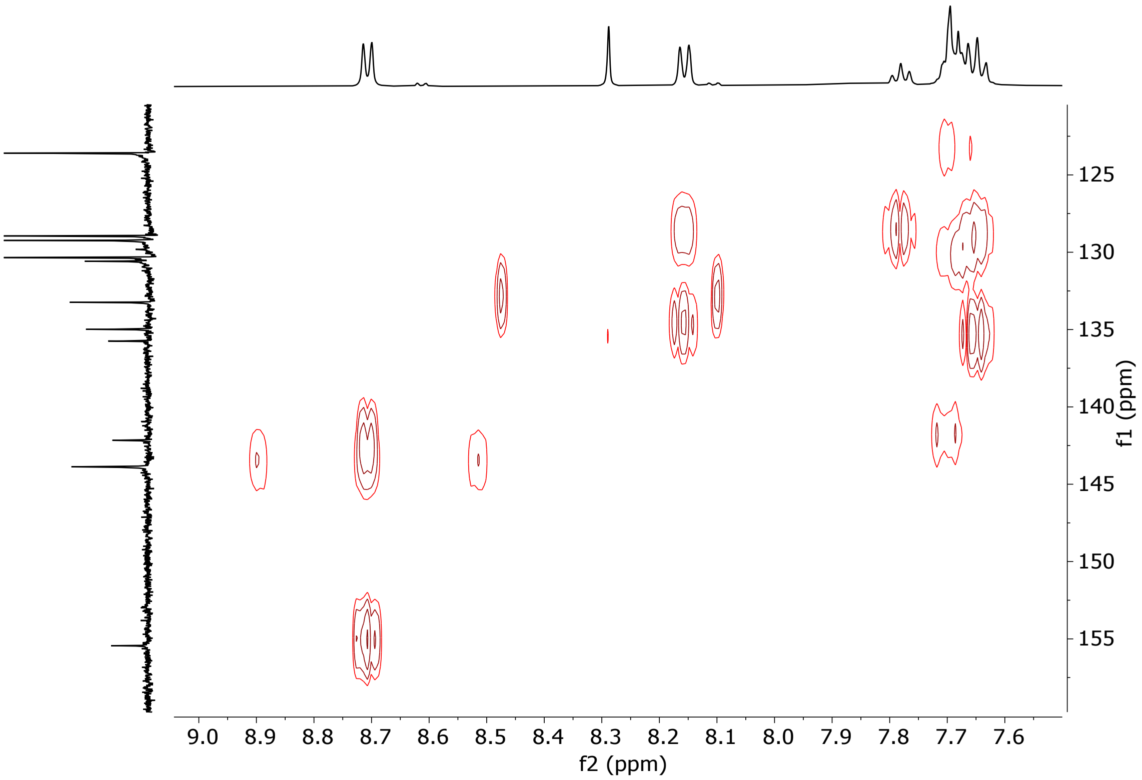

Figure S97. ^{1}H-^{13}C HMBC (500 MHz, D_2O) partial spectrum of the 254 nm PSS for the compound $\mathbf{P}_c$H·Cl.

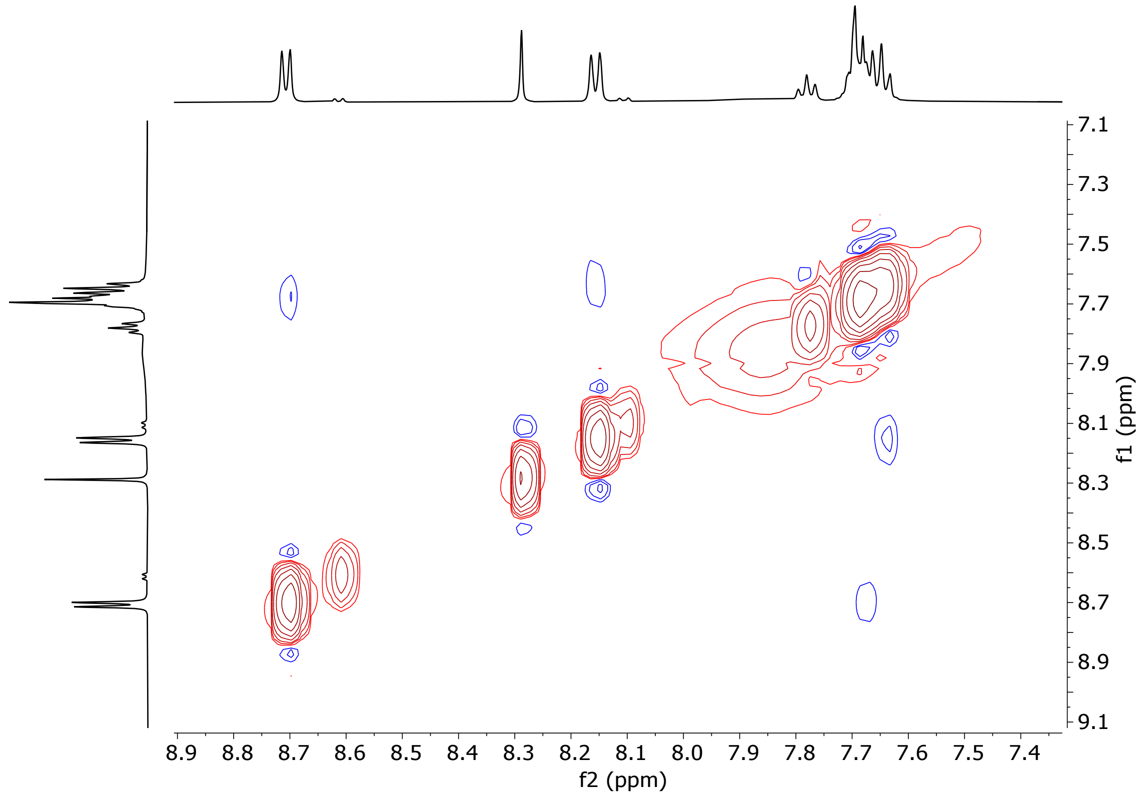

Figure S98. ^{1}H-^{1}H NOESY (500 MHz, D_2O) partial spectrum of the 254 nm PSS for the compound $\mathbf{P}_c$H·Cl.

2.6.2.2. Organic medium.

P$_c$H·PF$_6$ salt was dissolved at 5 mM in CD$_3$CN. The NMR tube containing the compound solution was irradiated for 16 h at 254 nm, after which all NMR experiments were acquired.

^{1}H NMR (500 MHz, CD$_3$CN) δ (*ppm*): 14.06 (s, 1H), 8.56 (d, *J* = 7.9 Hz, 2H), 8.27 (s, 1H), 8.17 (d, *J* = 7.0 Hz, 2H), 7.77 (t, *J* = 7.5 Hz, 1H), 7.72 – 7.66 (m, 4H), 7.66 – 7.61 (m, 5H).

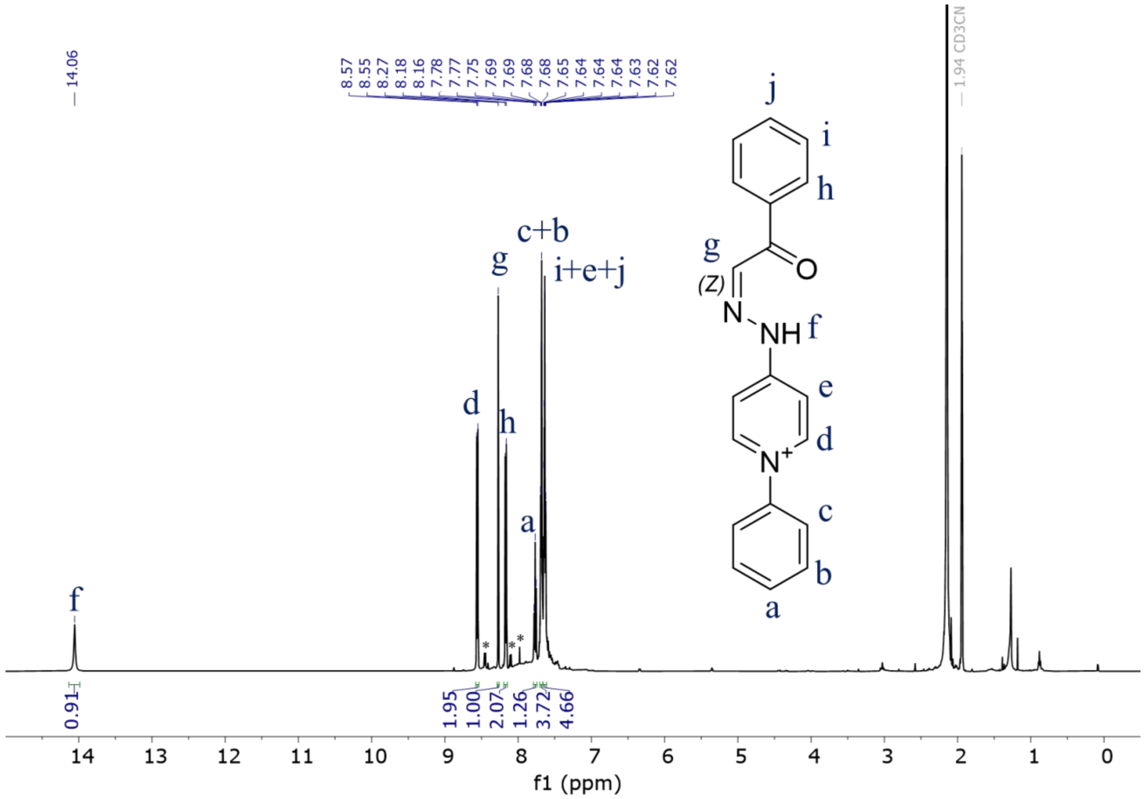

Figure S99. ^{1}H NMR (500 MHz, CD$_3$CN) spectrum of the 254 nm PSS for the compound **P**$_c$H·PF$_6$. Signals marked with an asterisk correspond to the *E*-isomer.

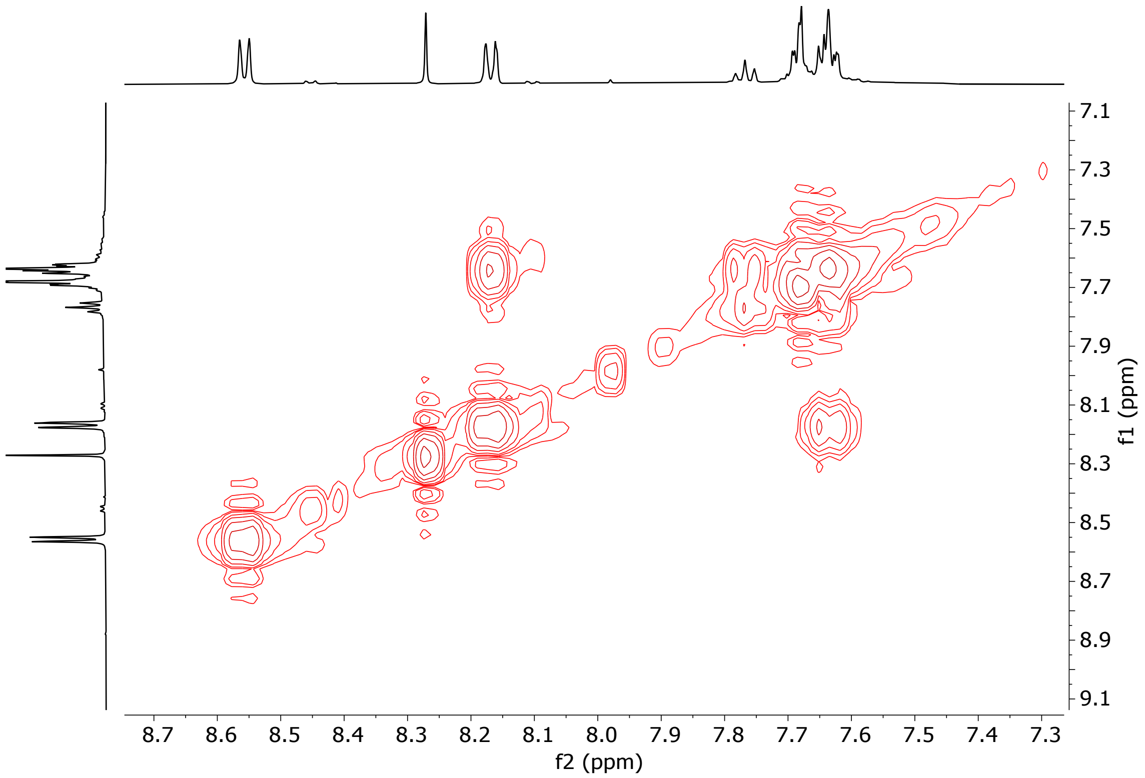

Figure S100. ^{1}H-^{1}H COSY (500 MHz, CD_3CN) partial spectrum of the 254 nm PSS for the compound **P**$_c$H·PF_6.

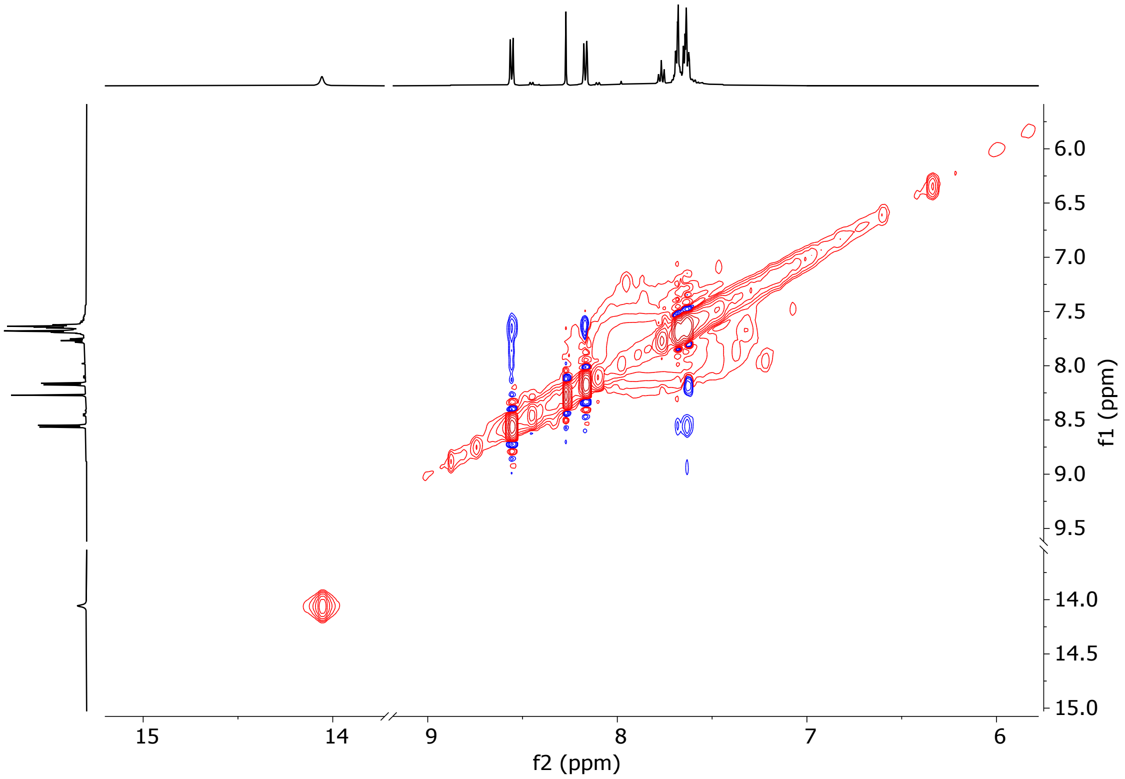

Figure S101. ^{1}H-^{1}H NOESY (500 MHz, CD_3CN) partial spectrum of the 254 nm PSS for the compound **P**$_c$H·PF_6. Blue cross peaks indicate NOE correlations The NH deshielding due to the formation of hydrogen bond is diagnostic of the *Z* isomer.

2.6.3. NMR data for the species assigned as *E*-P_c.

P_cH·Cl salt was dissolved at 5 mM in a D_2O phosphate buffer solution (20 mM, pD 13).

^{1}H NMR (500 MHz, D_2O) δ (*ppm*): 8.31 (s, 1H), 8.17 – 7.99 (m, 4H), 7.79 – 7.67 (m, 1H), 7.66 – 7.59 (m, 4H), 7.59 – 7.55 (m, 3H).

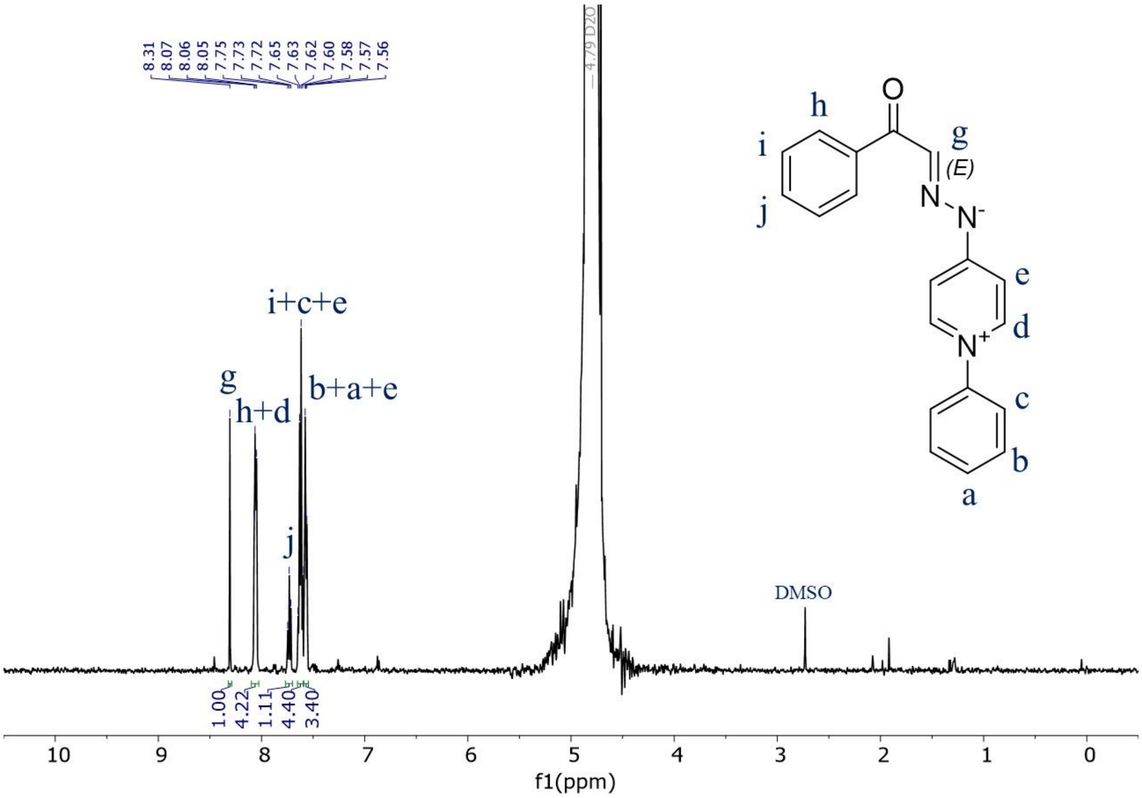

Figure S102. ^{1}H NMR (500 MHz, D_2O) spectrum of the compound P_cH·Cl as synthesized.

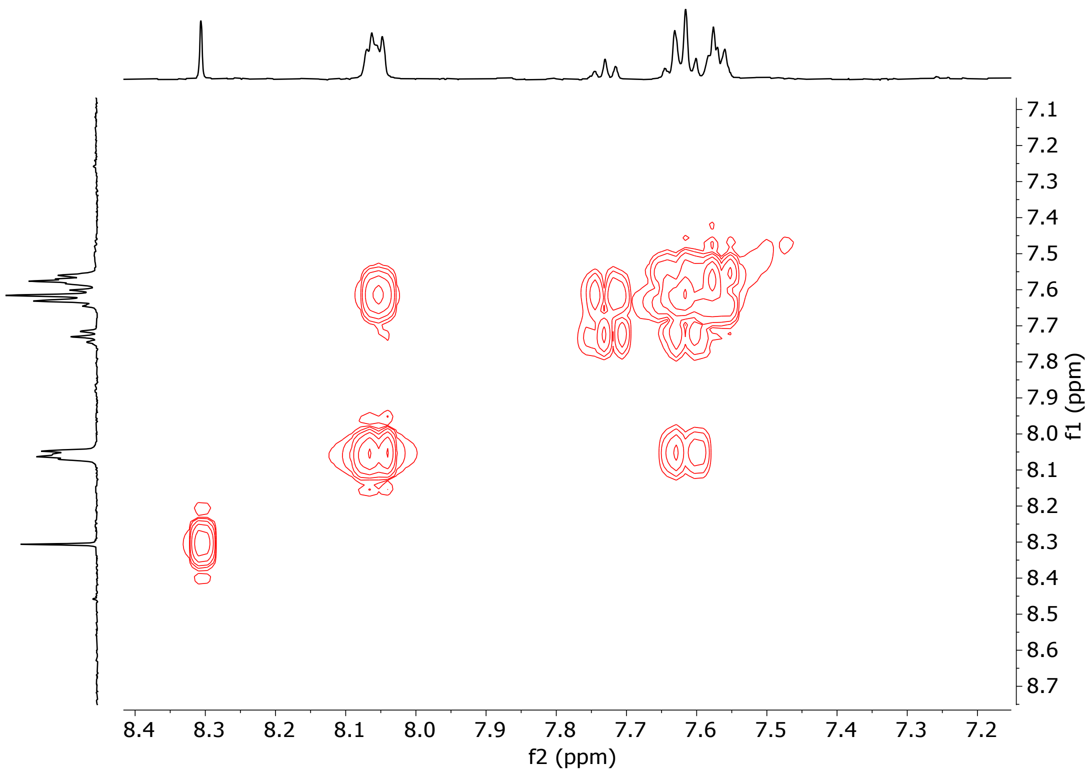

Figure S103. ^{1}H-^{1}H COSY (500 MHz, D_2O) spectrum of the compound **P**$_c$H·Cl as synthesized.

2.6.4. NMR data for the species assigned as *Z*-P_c.

P_cH·Cl salt was dissolved at 5 mM in a D_2O phosphate buffer solution (20 mM, pD 6). The NMR tube containing the compound solution was irradiated for 16 h at 254 nm. Immediately before data acquisition, the sample was basified to pD 13 using phosphate buffer (40 mM), resulting in a final compound concentration of 2.5 mM. The interval between basification and acquisition was approximately 1 min. For the ^{1}H and COSY experiments, 4 scans were applied in both cases, with acquisition times of ~1 min and ~60 min, respectively.

^{1}H NMR (500 MHz, D_2O) δ (*ppm*): 7.98 (d, J = 7.7 Hz, 2H), 7.83 (d, J = 7.5 Hz, 2H), 7.80 (s, 1H), 7.72 (t, J = 7.5 Hz, 1H), 7.67 – 7.57 (m, 4H), 7.56 – 7.46 (m, 3H), 6.97 – 6.68 (m, 2H).

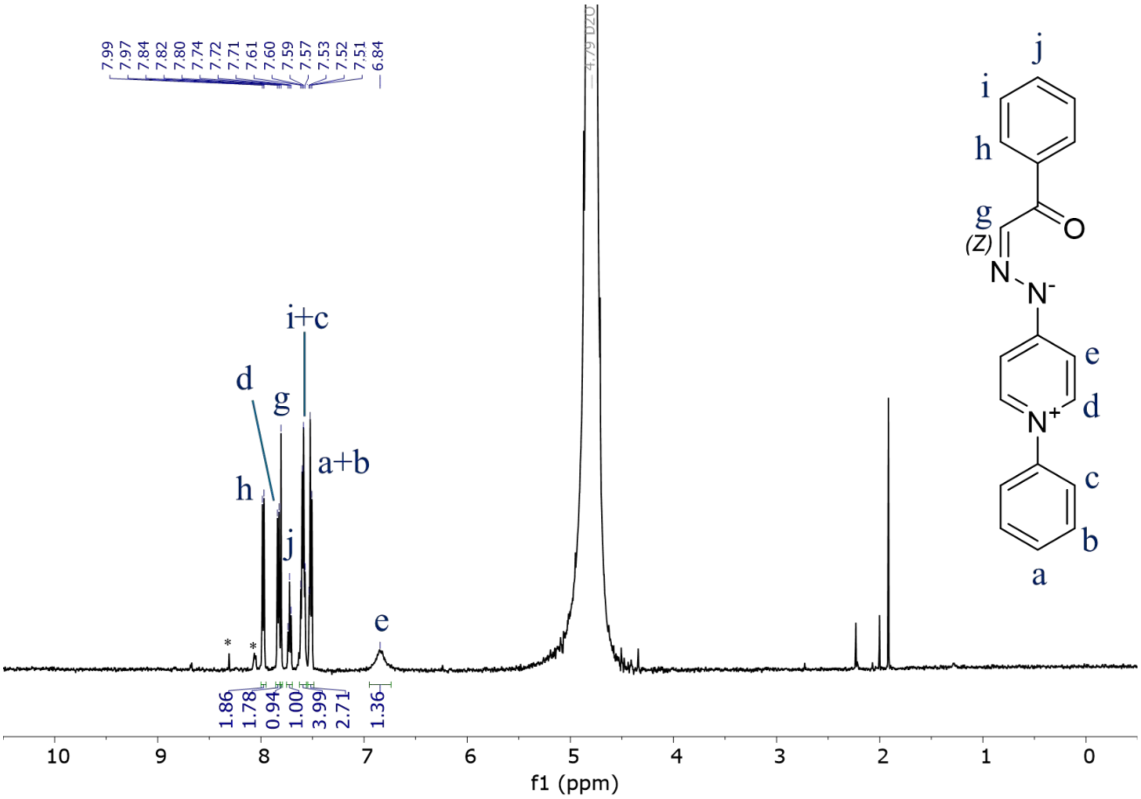

Figure S104. ^{1}H NMR (500 MHz, D_2O) spectrum of the basification after reaching the 254 nm PSS for the compound P_cH·Cl. Signals marked with an asterisk correspond to the *E*-isomer.

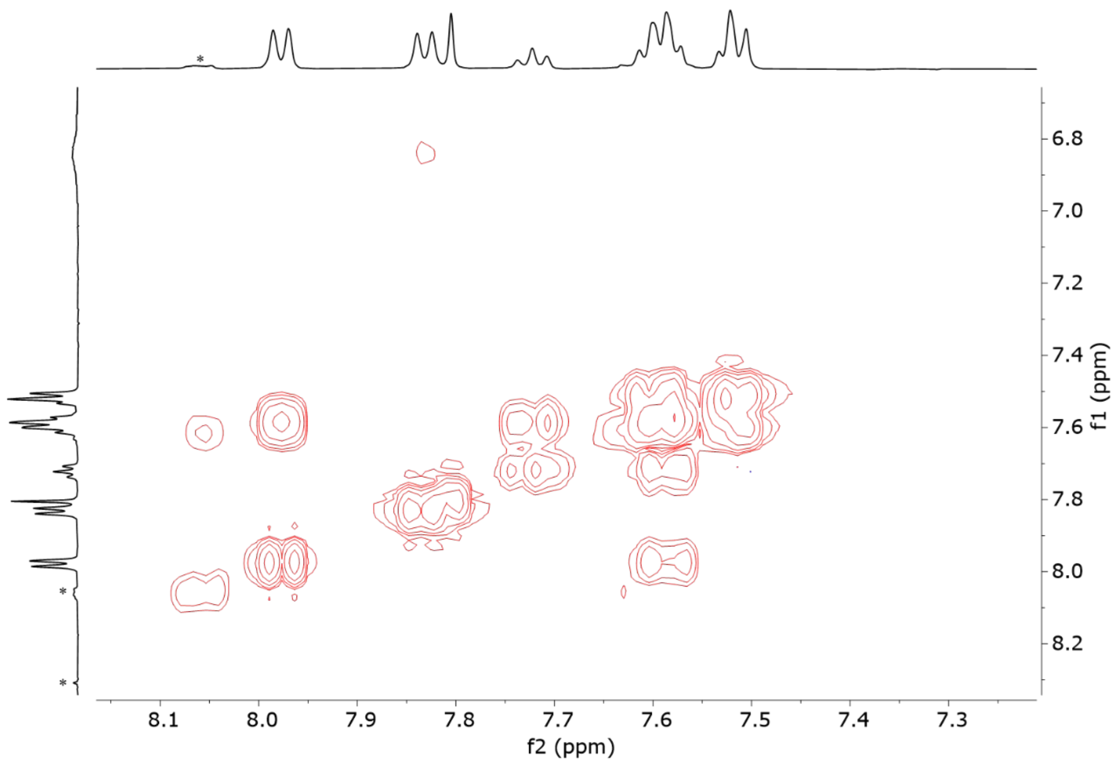

Figure S105. ^{1}H-^{1}H COSY (500 MHz, D_2O) partial spectrum of the basification after reaching the 254 nm PSS for the compound $\mathbf{P}_c$H·Cl. Due to the acquisition time of the experiment and the fast thermal reversion, cross-peaks of *E*-isomer exhibit a greater intensity than observed in the ^{1}H NMR. Signals marked with an asterisk correspond to the *E*-isomer.

2.7 Synthesis and NMR data of 2·I.

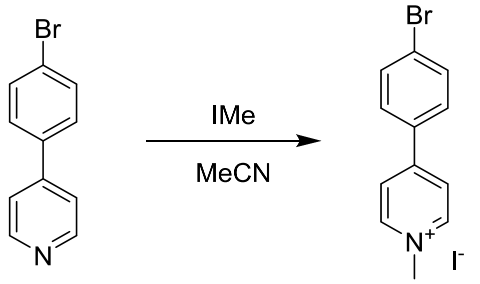

4-(4-Bromophenyl)pyridine (1 g, 4.3 mmol, 1 equiv) was dissolved in 60 mL of MeCN in a round bottom flask. Iodomethane (5.3 mL, 85.4 mmol, 20 equiv) was then added dropwise and the mixture reaction was stirred at r.t. in a magnetic hot plate stirrer for 40 h. Upon completion, the resulting precipitate was filtered, washed with CH_3CN (3 x 10 mL) and Et_2O (3 x 10 mL), and dried under vacuum to yield **2**·I as a beige solid (1.5 g, 93%).

^{1}H NMR (400 MHz, D_2O) δ (*ppm*): 8.79 (d, J = 6.9 Hz, 2H), 8.30 (d, J = 6.9 Hz, 2H), 7.90 – 7.79 (m, 4H), 4.40 (s, 3H).

^{13}C{^{1}H} NMR (101 MHz, D_2O) δ (*ppm*): 155.2 (C_{Ar}), 145.0 (CH_{Ar}), 133.0 (C_{Ar}), 132.7 (CH_{Ar}), 129.5 (CH_{Ar}), 126.4 (C_{Ar}), 124.7 (CH_{Ar}), 47.3 (CH_3).

HRMS (ESI) m/z: Calcd for $C_{12}H_{11}BrN^+$ 248.0069; found 248.0068.

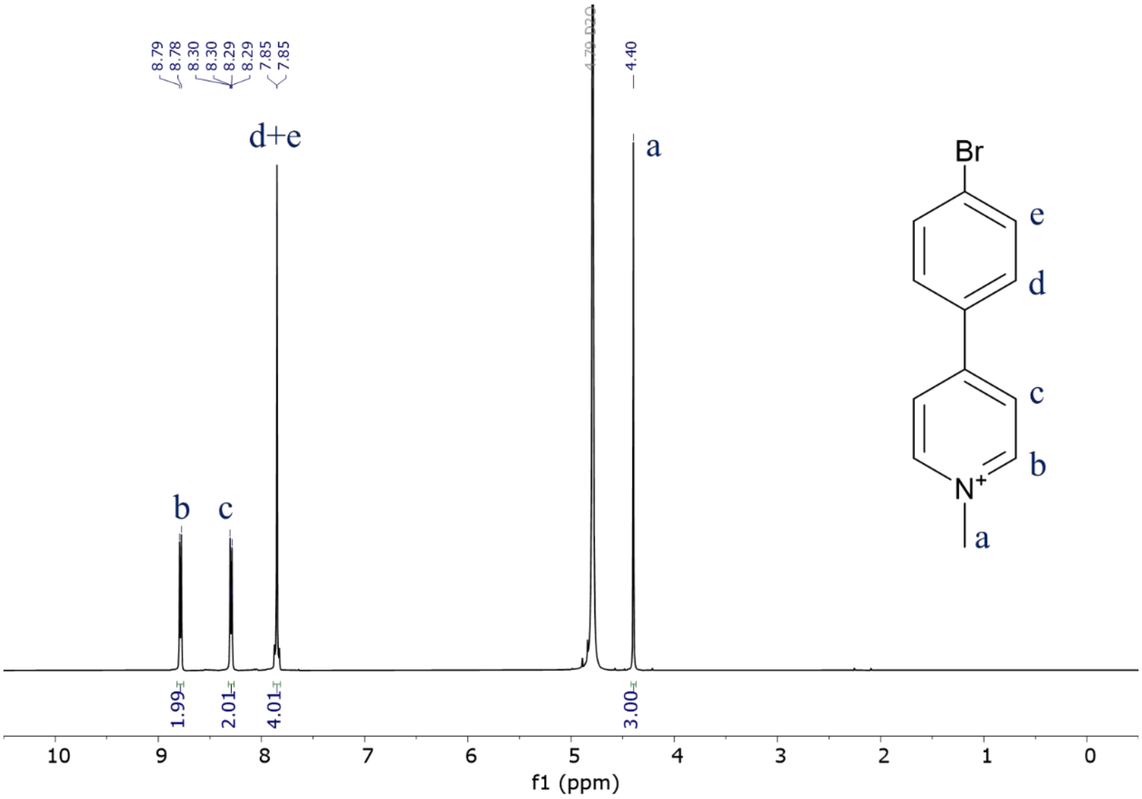

Figure S106. ^{1}H NMR (400 MHz, D_2O) spectrum of **2**·I.

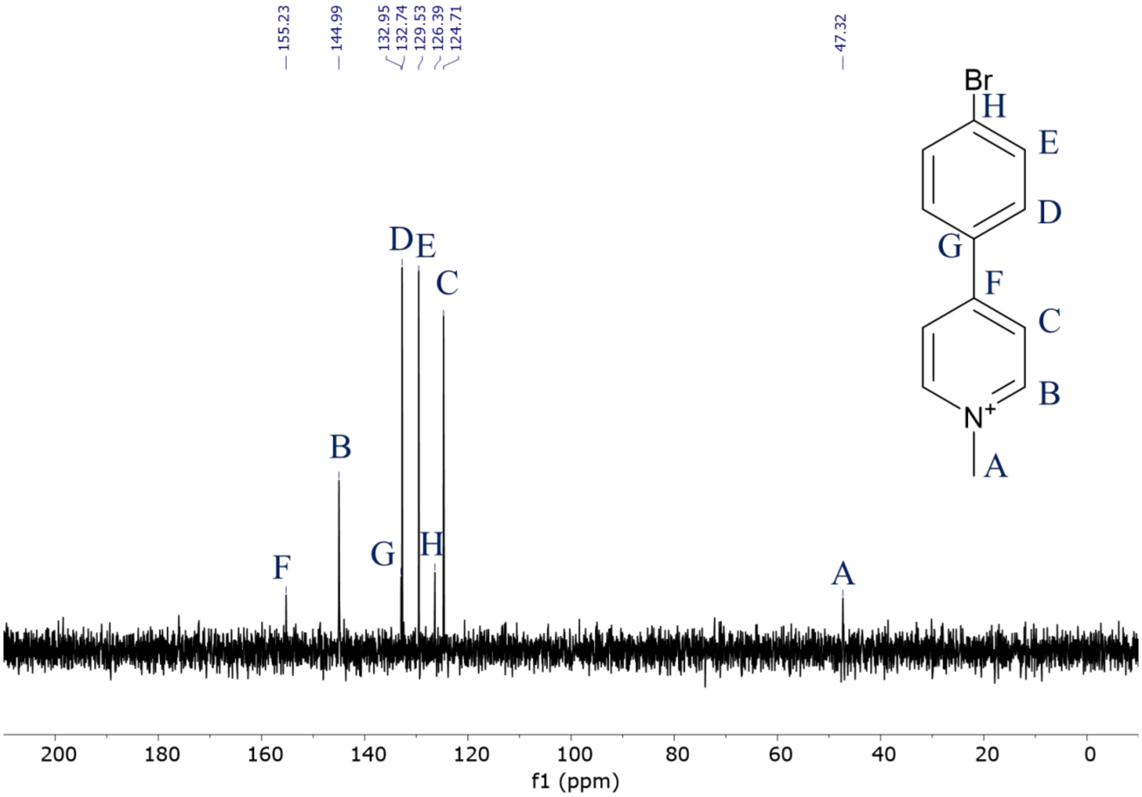

Figure S107. $^{13}C\{^1H\}$ NMR (101 MHz, D_2O) spectrum of **2**·I.

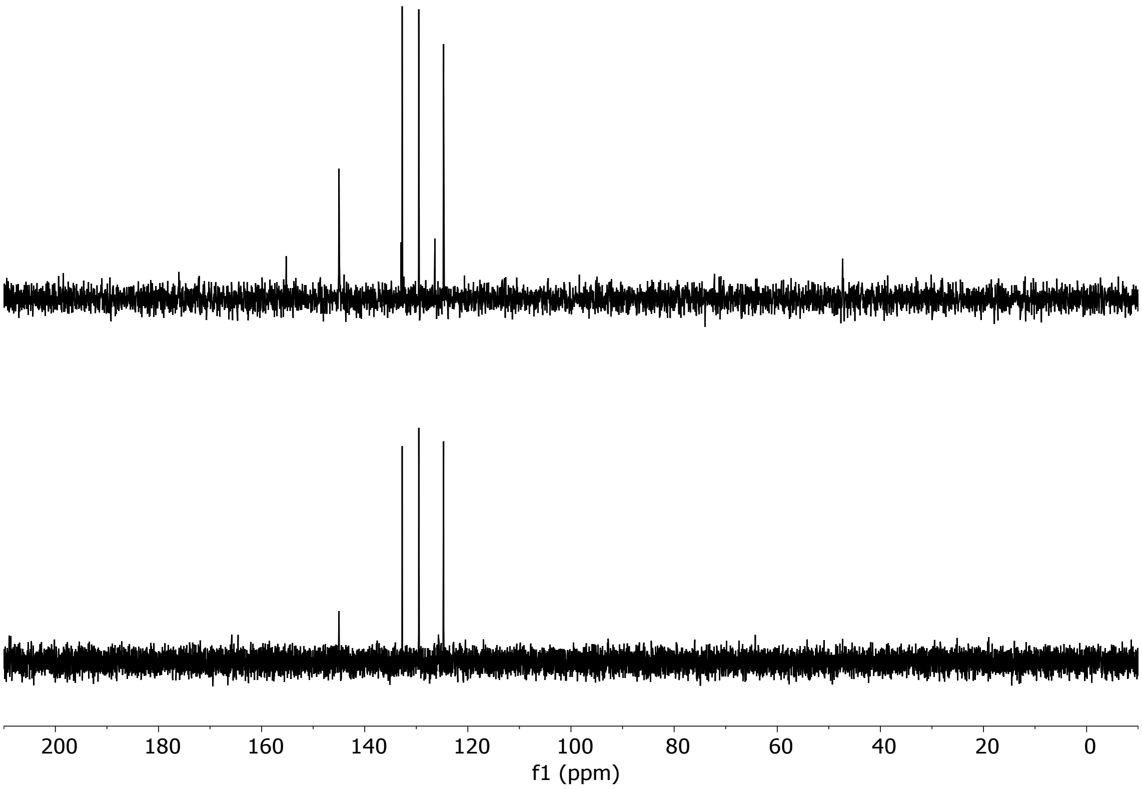

Figure S108. DEPT-135 NMR (101 MHz, D_2O) and $^{13}C\{^1H\}$ NMR (101 MHz, D_2O) stacked spectra of **2**·I.

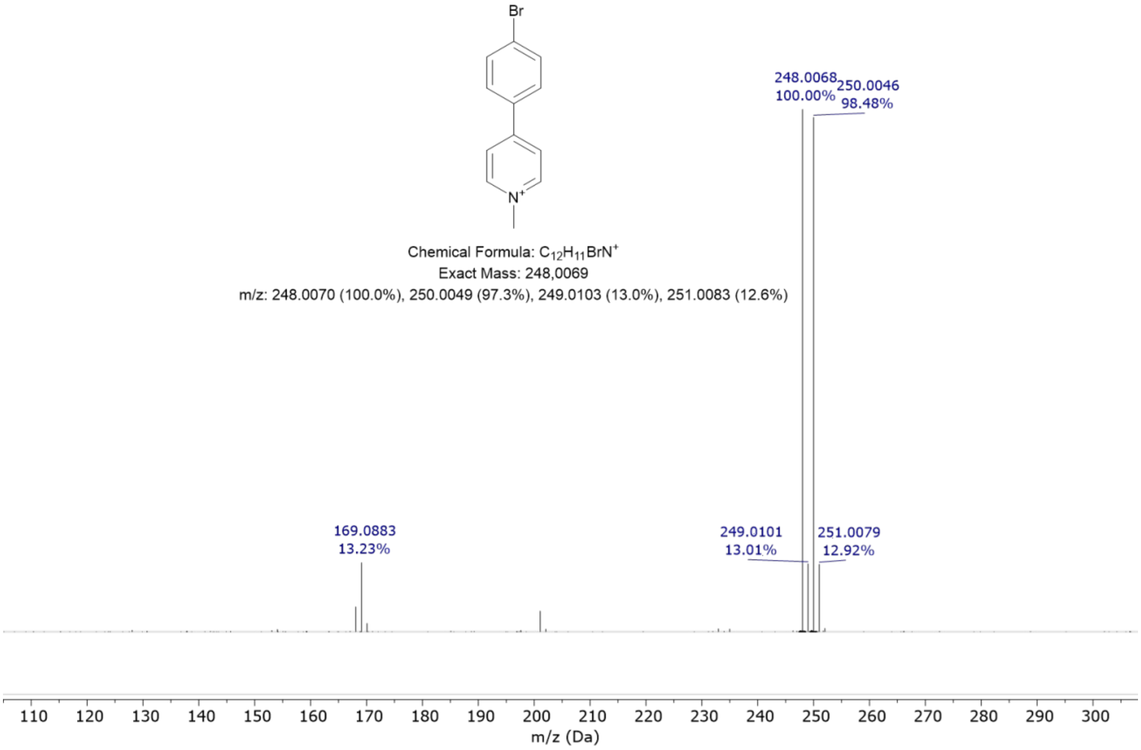

Figure S109. HRMS-ESI spectrum of **2**·I.

2.8. Synthesis and NMR data of H_d·I.

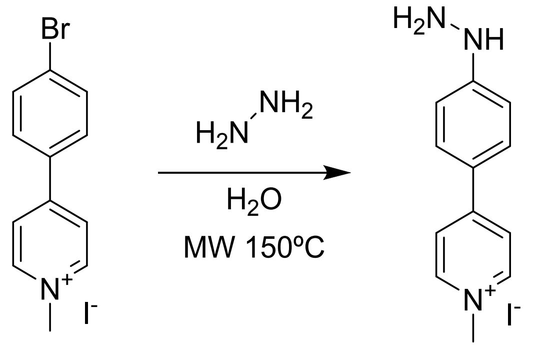

Precursor **2**·I (1.5 g, 4.0 mmol, 1 equiv) and hydrazine hydrate (2 mL, 31.92 mmol, 8 equiv) were dissolved in 17 mL of H_2O in a microwave sealed reaction flask. The reaction mixture was heated at 150ºC in a microwave reactor with stirring for 14 h. Upon completion, the resulting precipitate was filtered, washed with H_2O (2mL) and Et_2O (2 x 10 mL), and dried under vacuum to yield **H_d**·I as an orange solid (557 mg, 43%).

^{1}H NMR (400 MHz, D_2O) δ (*ppm*): 8.58 (d, J = 7.0 Hz, 2H), 8.18 (d, J = 7.0 Hz, 2H), 7.93 (d, J = 8.9 Hz, 2H), 7.10 (d, J = 8.9 Hz, 2H), 4.29 (s, 3H).

^{13}C{^{1}H} NMR (101 MHz, D_2O) δ (*ppm*): 154.4 (C_{Ar}), 144.2 (CH_{Ar}), 134.1 (C_{Ar}), 129.4 (CH_{Ar}), 123.5 (C_{Ar}), 122.5 (CH_{Ar}), 113.1 (CH_{Ar}), 46.6 (CH_3).

HRMS (ESI) m/z: Calcd for $C_{12}H_{14}N_3^+$ 200.1182; found 200.1182.

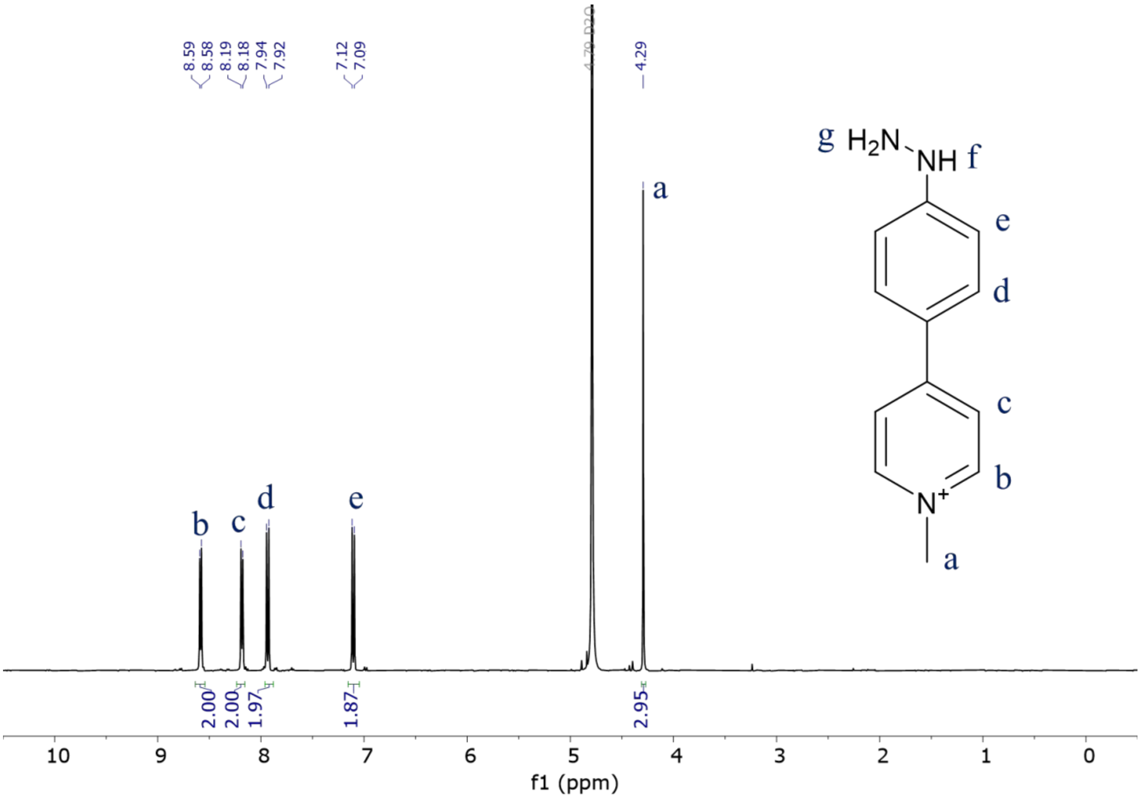

Figure S110. ^{1}H NMR (400 MHz, D_2O) spectrum of $\mathbf{H}_d$·I.

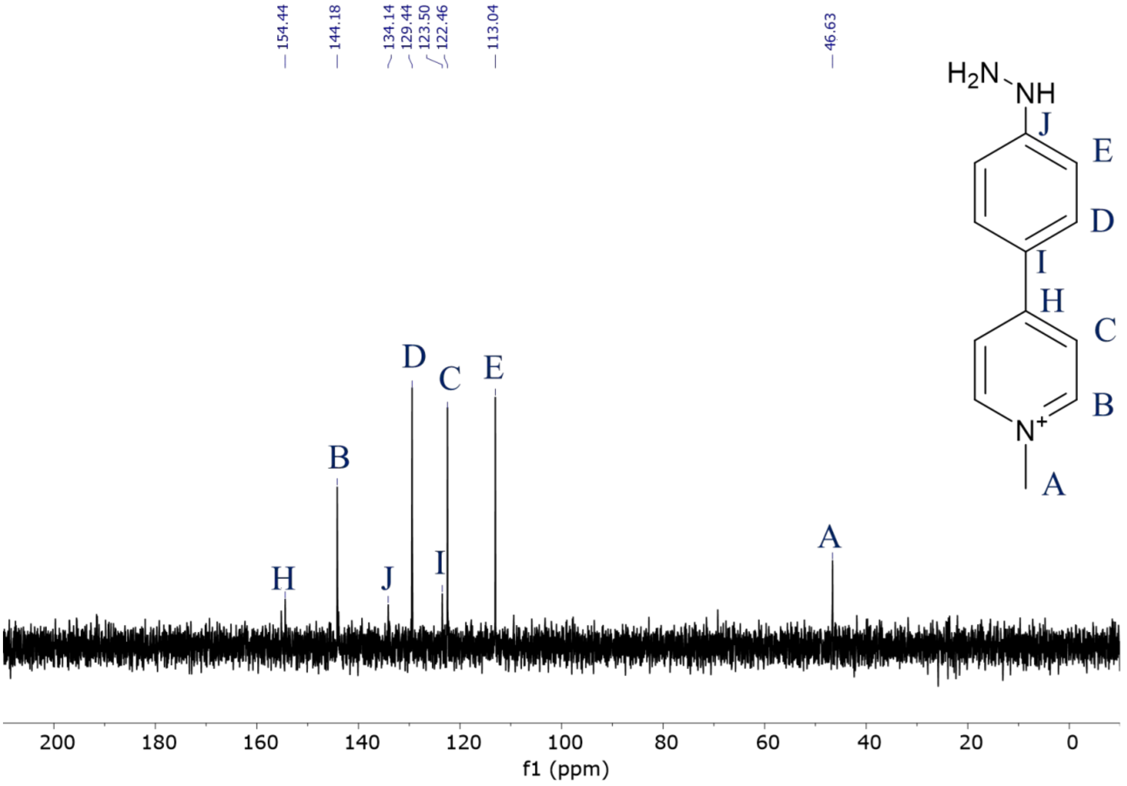

Figure S111. ^{13}C{^{1}H} NMR (101 MHz, D_2O) spectrum of $\mathbf{H}_d$·I.

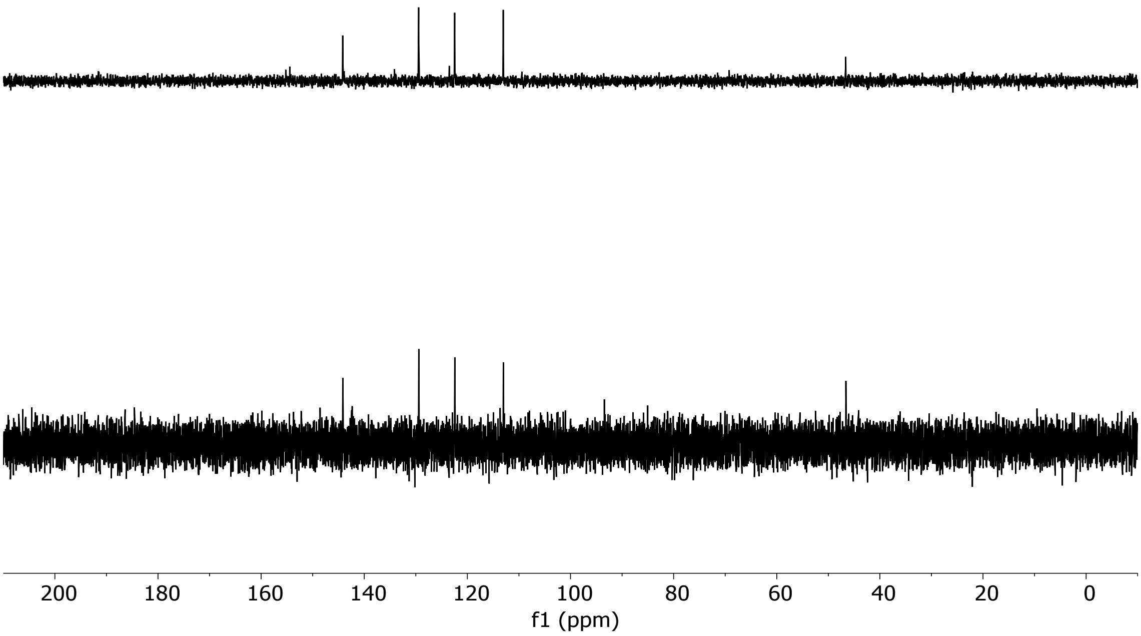

Figure S112. DEPT-135 NMR (101 MHz, D_2O) and $^{13}C\{^1H\}$ NMR (101 MHz, D_2O) stacked spectra of $\mathbf{H}_d$·I.

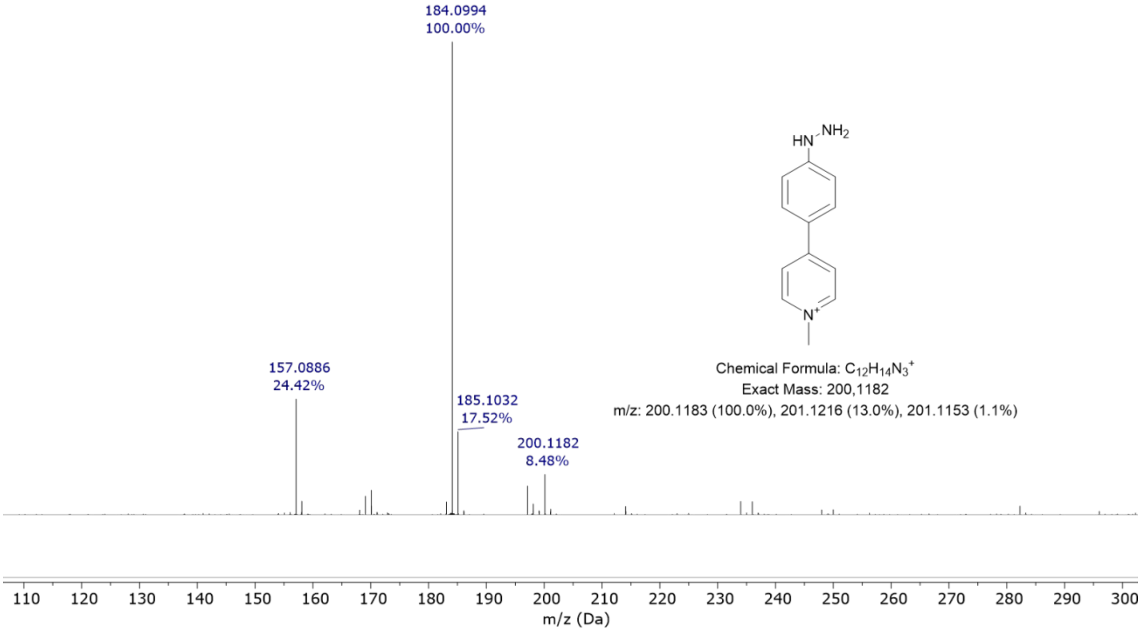

Figure S113. HRMS-ESI spectrum of $\mathbf{H}_d$·I.

2.9. Synthesis and characterization data of $P_dH{\cdot}Cl$.

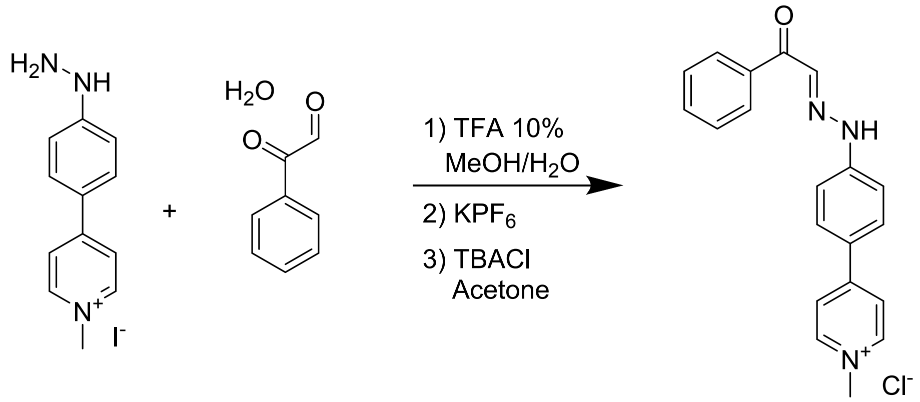

Precursor $\mathbf{H}_d{\cdot}I$ (200 mg, 0.6 mmol, 1 equiv) was dissolved in 50 mL H_2O and 50 mL EtOH in a round bottom flask. Phenylglyoxal (102 mg, 0.7 mmol, 1.1 equiv), and TFA (5 µL, 0.1 mmol, 0.1 equiv), were then added and the resulting mixture was heated at 60ºC in a magnetic hot plate stirrer during 3h in the absence of light. After the reaction was completed, the EtOH was removed in a rotary evaporator and KPF_6 (449 mg, 2.4 mmol, 4 equiv) was added to the solution. The resulting precipitate was filtered, washed with H_2O (3 x 10 mL) and Et_2O (3 × 10 mL) and dried under vacuum to $\mathbf{P}_dH{\cdot}PF_6$ as a yellow solid (227.5 mg, 81%). $\mathbf{P}_dH{\cdot}PF_6$ was dissolved in acetone and a saturated solution of TBACl in acetone was added. After 1 h under stirring, the resulting precipitate was filtered and washed with acetone (2 x 10 mL) and Et_2O (2 x 10 mL), and dried under vacuum to yield $\mathbf{P}_dH{\cdot}Cl$ (138.8 mg, 49%) as a pale yellow solid.

HRMS (ESI) m/z: Calcd for $C_{20}H_{18}N_3O^+$ 316.1444; found 316.1447.

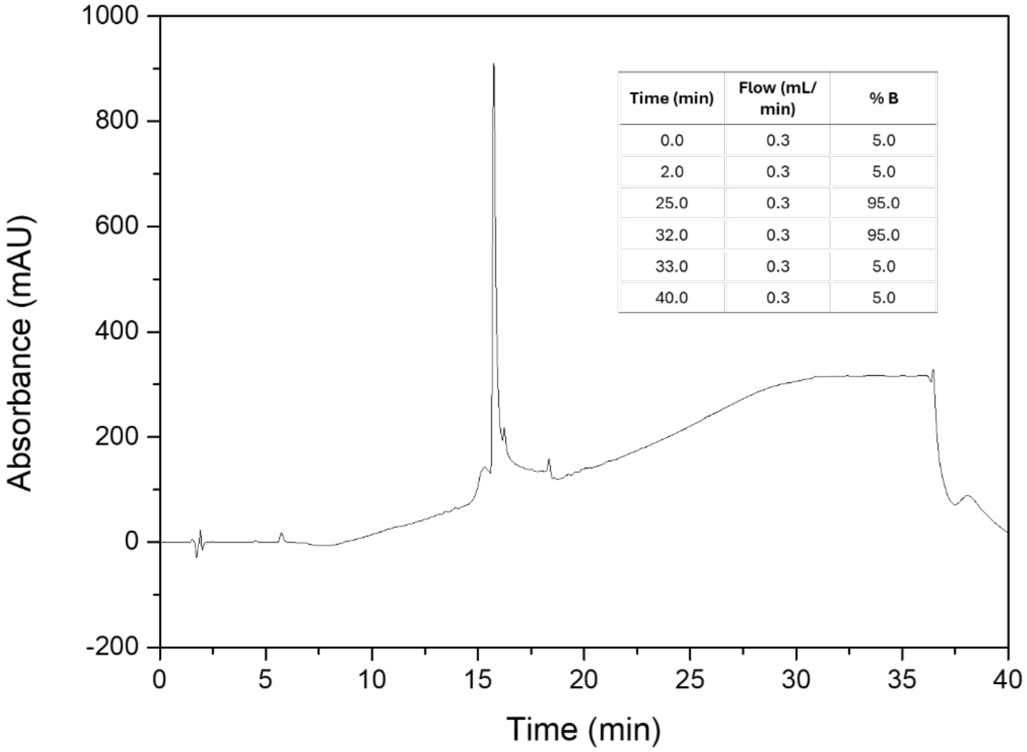

Figure S114. HPLC chromatogram (220 nm) of $\mathbf{P}_d$H·Cl at t_R = 15.75 min (Inset: separation method; A = H_2O + 0.04 % TFA, B = CH_3CN + 0.04 % TFA).

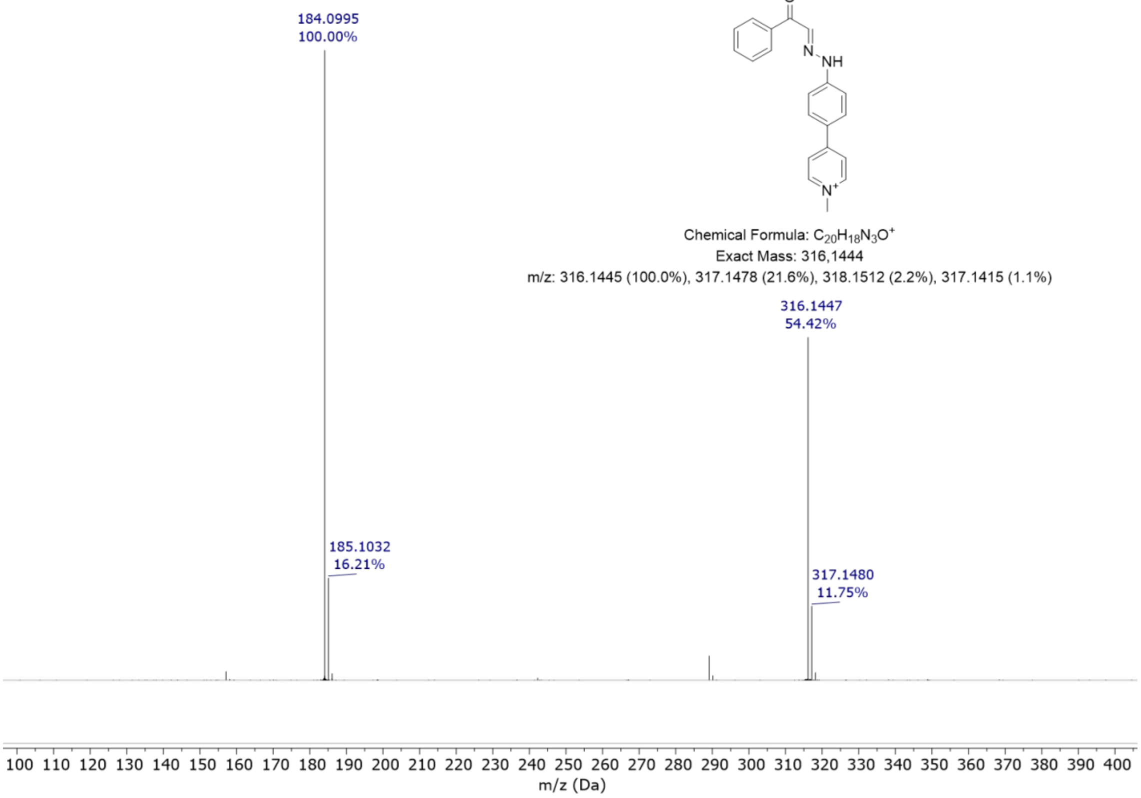

Figure S115. HRMS-ESI spectrum of $\mathbf{P}_d$H·Cl.

2.9.1. NMR data for the species assigned as *E*-P_dH^+.

2.9.1.1. Aqueous medium.

P$_d$H·Cl salt was dissolved at 5 mM in a D_2O phosphate buffer solution (20 mM, pD 6).

^{1}H NMR (500 MHz, D_2O) δ (*ppm*): 8.10 (d, J = 6.4 Hz, 2H), 7.63 (d, J = 6.4 Hz, 2H), 7.57 (d, J = 8.5 Hz, 2H), 7.51 – 7.46 (m, 3H), 7.33 – 7.25 (m, 3H), 6.98 (d, J = 8.2 Hz, 2H), 3.96 (s, 3H).

^{13}C{^{1}H} NMR (126 MHz, D_2O) δ (*ppm*): 187.7 (C=O), 153.2 (C_{Ar}), 145.9 (C_{Ar}), 143.7 (CH_{Ar}), 135.5 (C_{Ar}), 133.5 (CH_{Ar}), 130.9 (CH=N), 129.0 (CH_{Ar}), 128.7 (CH_{Ar}), 128.6 (CH_{Ar}), 125.4 (C_{Ar}), 122.0 (CH_{Ar}), 114.7 (CH_{Ar}), 46.4 (CH_3).

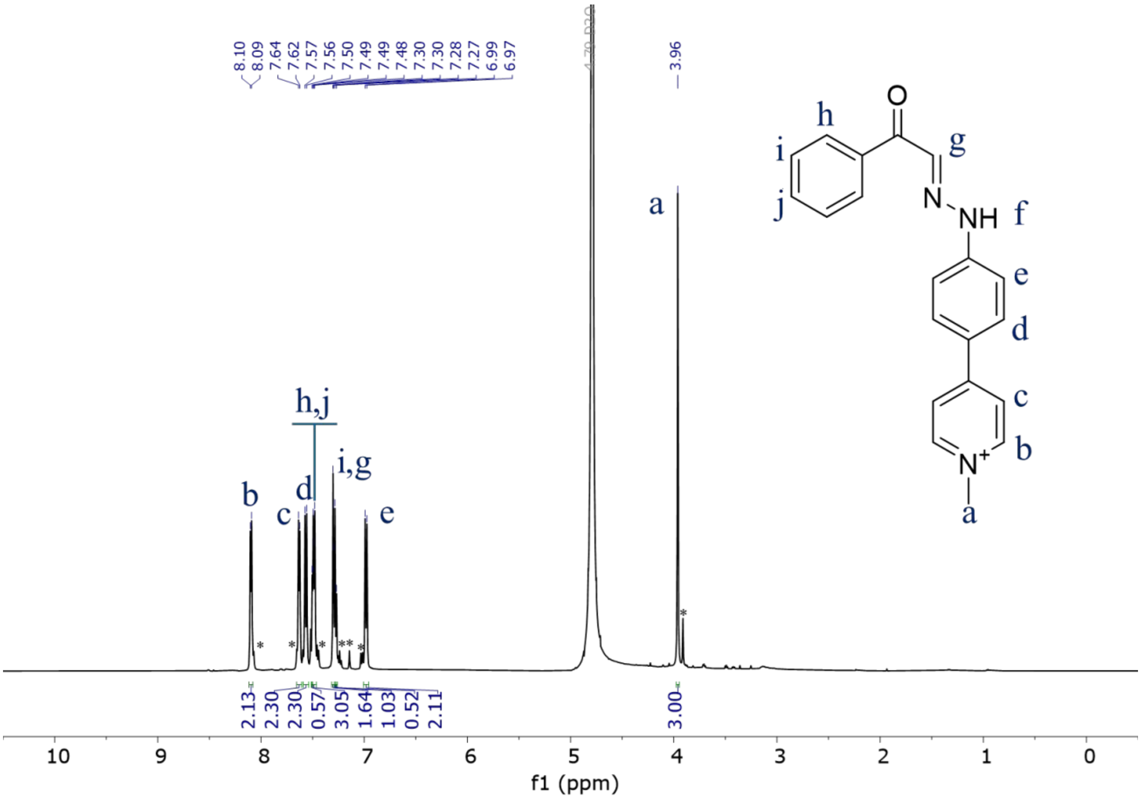

Figure S116. ^{1}H NMR (500 MHz, D_2O) spectrum of the compound **P**$_d$H·Cl as synthesized. Signals marked with an asterisk correspond to the *Z* isomer.

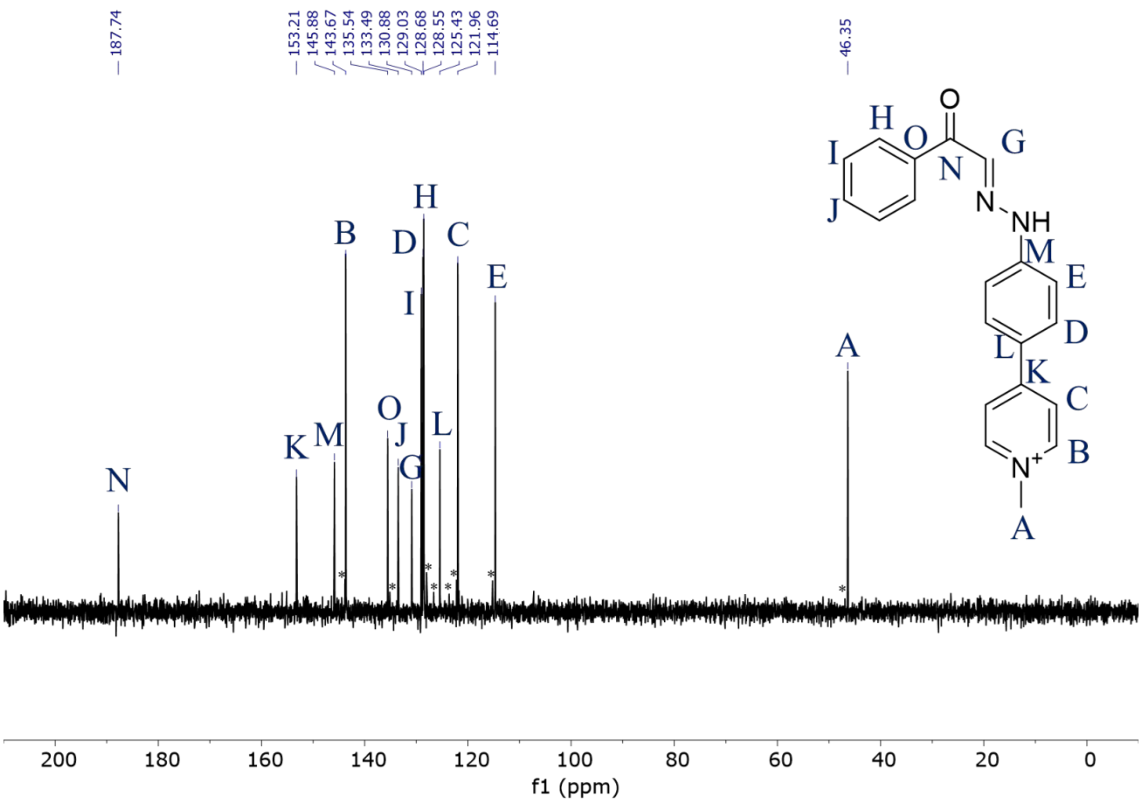

Figure S117. $^{13}C\{^{1}H\}$ NMR (126 MHz, D_2O) spectrum of the compound $\mathbf{P}_d$H·Cl as synthesized. Signals marked with an asterisk correspond to the *Z* isomer.

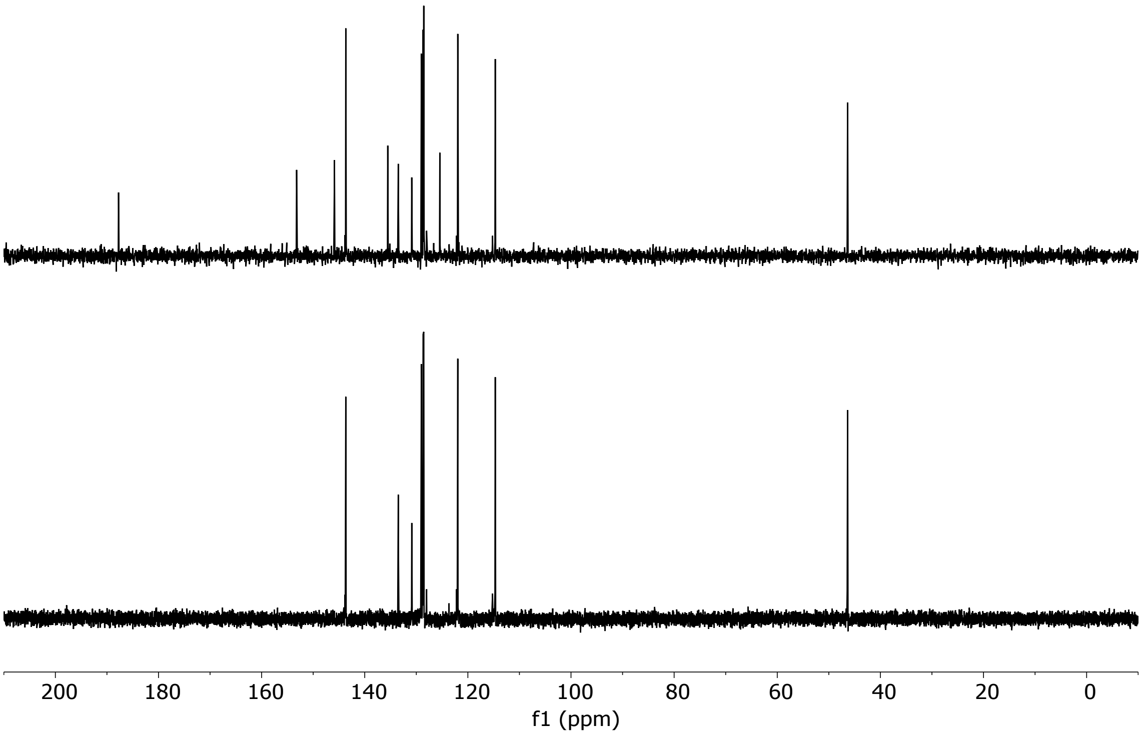

Figure S118. DEPT-135 NMR (126 MHz, D_2O) and $^{13}C\{^{1}H\}$ NMR (126 MHz, D_2O) stacked spectra of the compound $\mathbf{P}_d$H·Cl as synthesized.

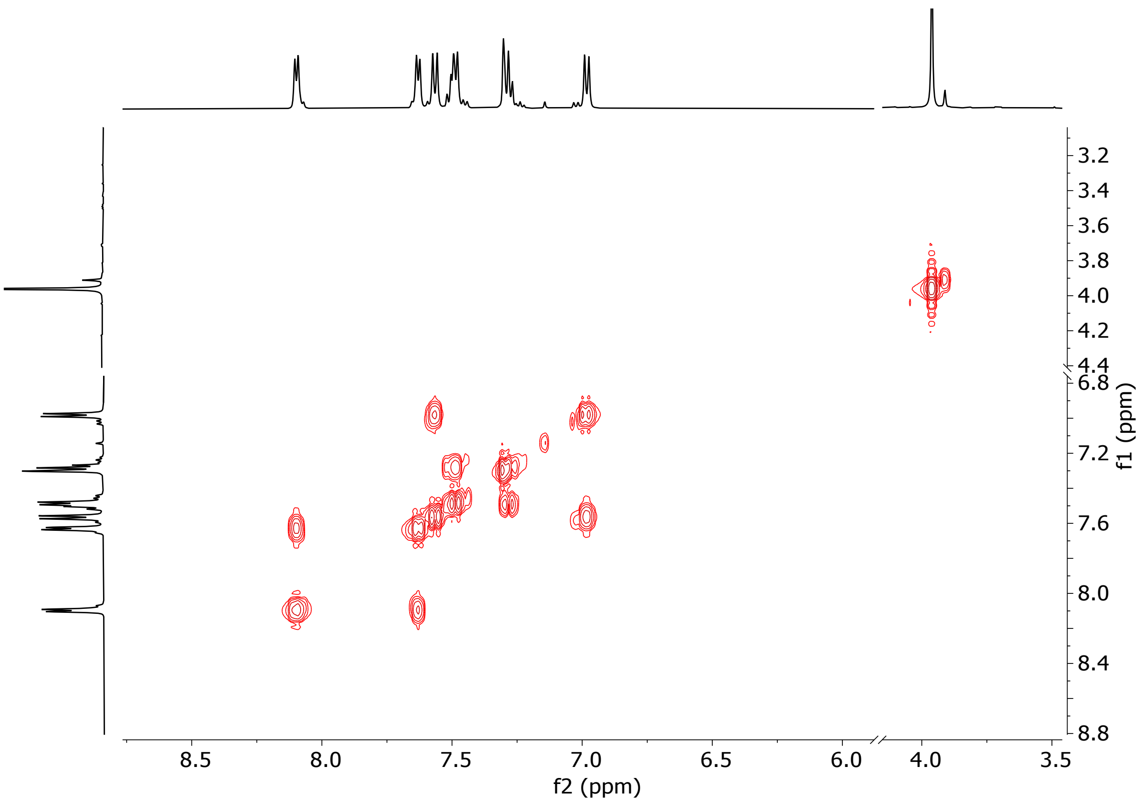

Figure S119. ^{1}H-^{1}H COSY (500 MHz, D_2O) partial spectrum of the compound $\mathbf{P}_d$H·Cl as synthesized.

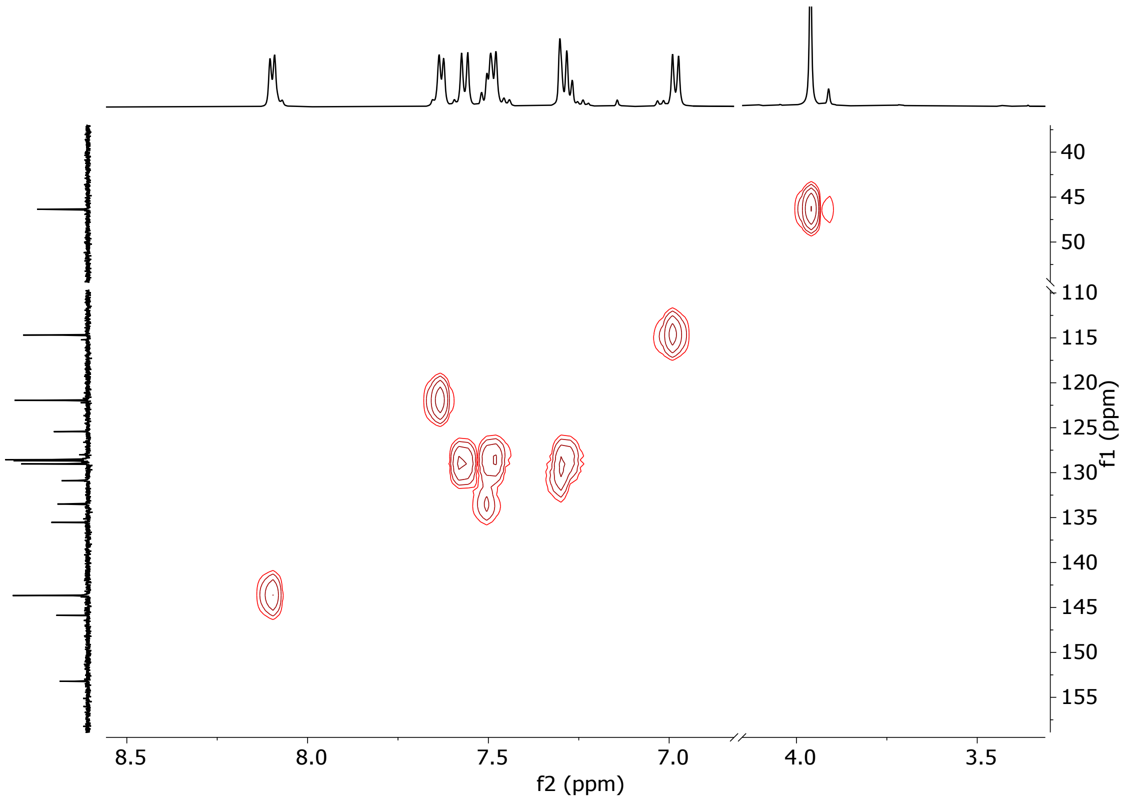

Figure S120. ^{1}H-^{13}C HSQC (500 MHz, D_2O) partial spectrum of the compound $\mathbf{P}_d$H·Cl as synthesized.

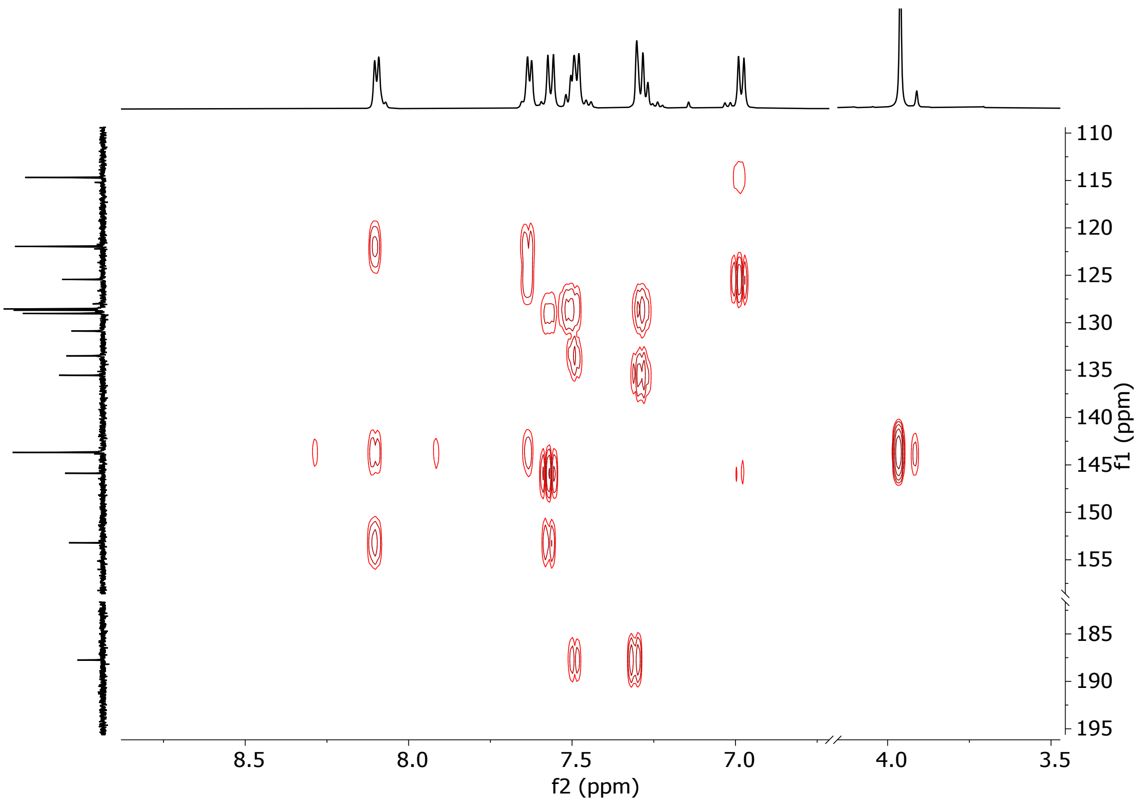

Figure S121. ^{1}H-^{13}C HMBC (500 MHz, D_2O) partial spectrum of the compound $\mathbf{P}_d$H·Cl as synthesized.

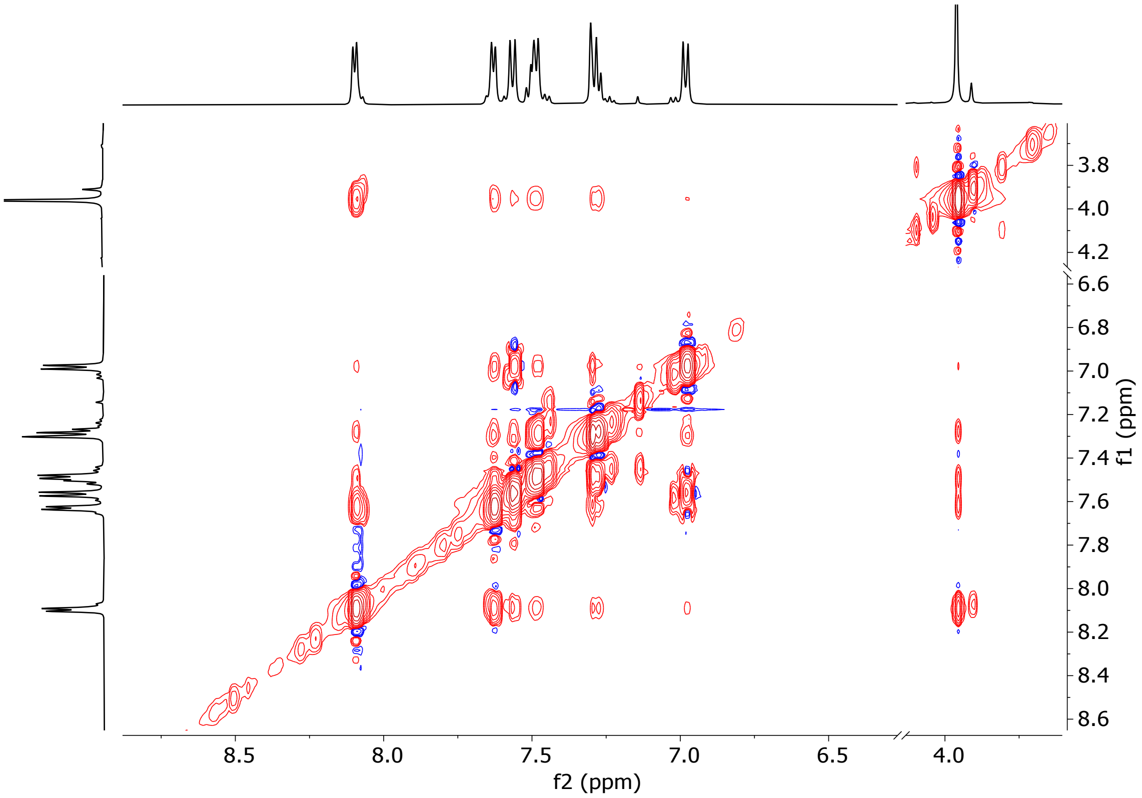

Figure S122. ^{1}H-^{1}H NOESY (500 MHz, D_2O) partial spectrum of the compound $\mathbf{P}_d$H·Cl as synthesized. Blue cross peaks indicate NOE correlations.

2.9.1.2. Organic medium.

$\mathbf{P_dH{\cdot}PF_6}$ salt was dissolved at 5 mM in CD_3CN.

^{1}H NMR (400 MHz, CD_3CN) δ (*ppm*): 9.90 (s, 1H), 8.48 (d, J = 6.9 Hz, 2H), 8.15 (d, J = 7.1 Hz, 2H), 8.10 – 8.03 (m, 2H), 7.92 (d, J = 8.9 Hz, 2H), 7.73 (s, 1H), 7.68 – 7.61 (m, 1H), 7.59 – 7.51 (m, 2H), 7.32 (d, J = 8.9 Hz, 2H), 4.21 (s, 3H).

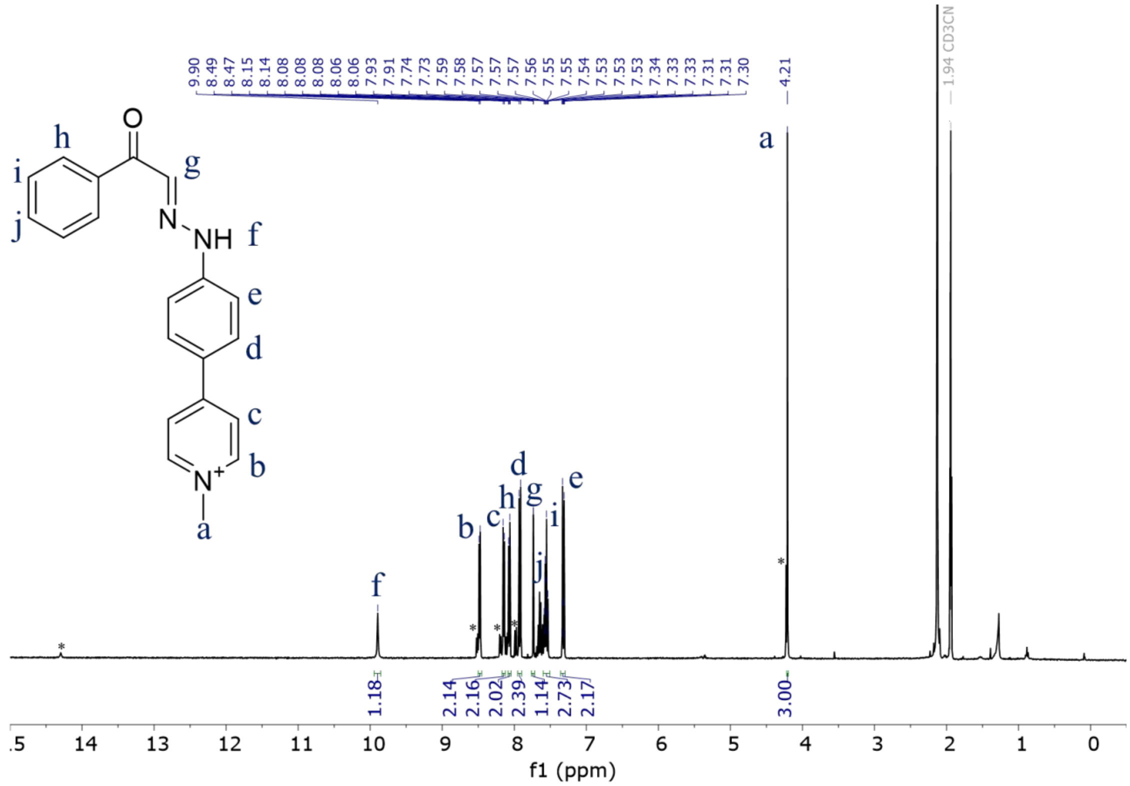

Figure S123. ^{1}H NMR (400 MHz, CD_3CN) spectrum of the compound $\mathbf{P_dH{\cdot}PF_6}$ as synthesized. Signals marked with an asterisk correspond to the *Z* isomer.

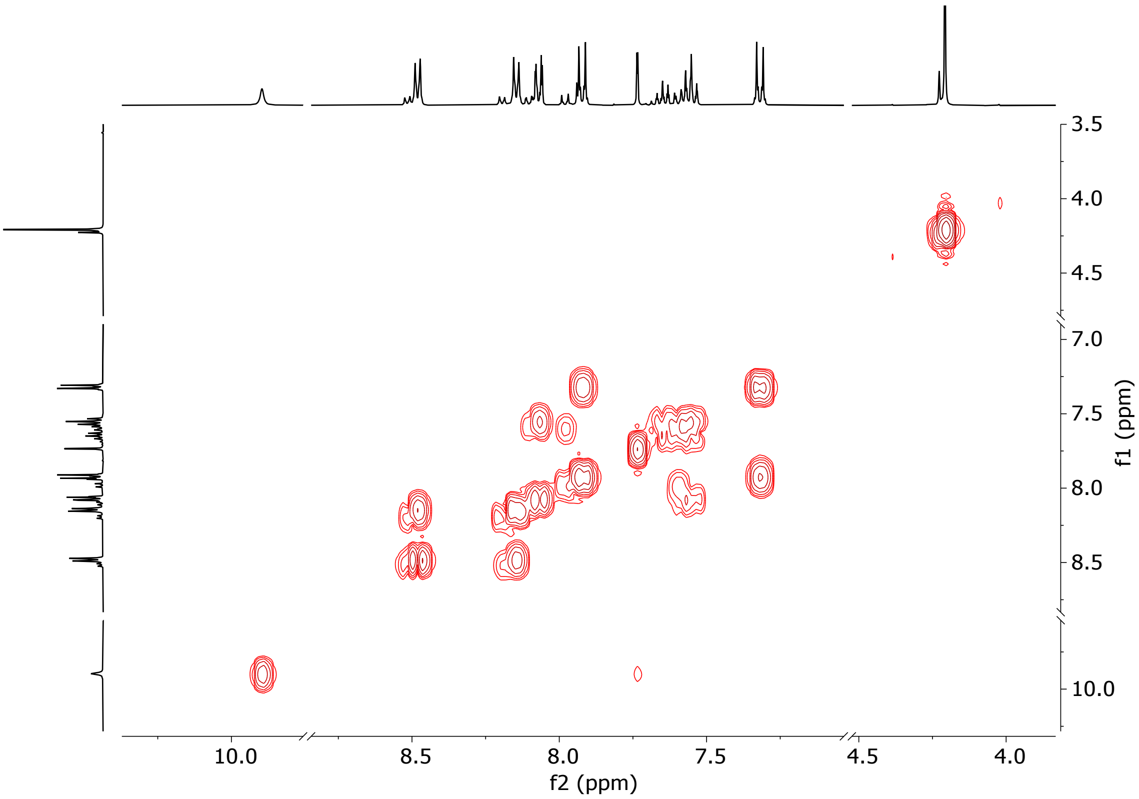

Figure S124. ^1H-^1H COSY (400 MHz, CD_3CN) partial spectrum of the compound $\mathbf{P}_d$H·PF_6 as synthesized.

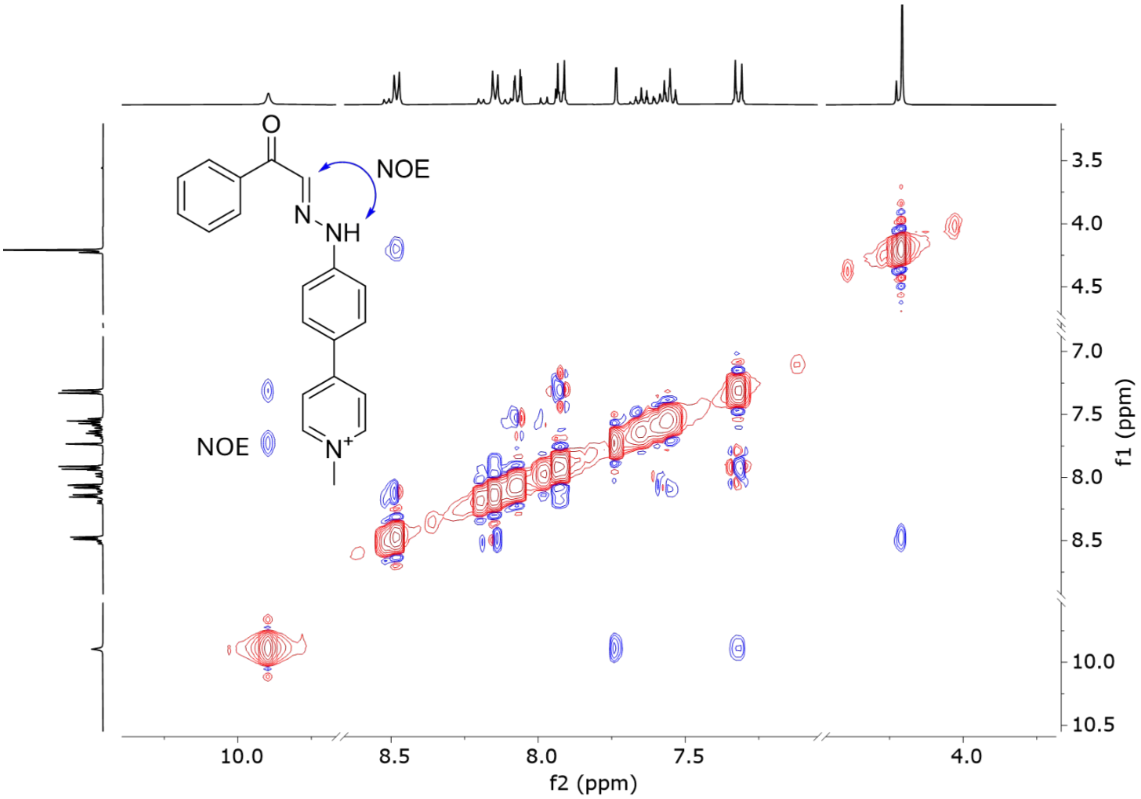

Figure S125. ^1H-^1H NOESY (400 MHz, CD_3CN) partial spectrum of the compound $\mathbf{P}_d$H·PF_6 as synthesized. Blue cross peaks indicate NOE correlations. The observed NOE between the NH and the imine proton is diagnostic of the *E*-isomer.

2.9.2. NMR data of the species assigned as *E*-$\mathbf{P_d}$.

Due to the low solubility of $\mathbf{P_d}$H·Cl in basic medium in D_2O, it was not possible to characterize the conjugate base $\mathbf{P_d}$ by NMR.

3. Hydrolytic stability of $\mathbf{P}_{a\text{-}d}$H·Cl compounds.

$\mathbf{P}_{a\text{-}d}$H·Cl salts were dissolved at 5 mM in a D_2O phosphate buffer solution (20 mM, pD 5.5). The NMR spectra of the compounds were recorded at various time points over a period of 7 days. No hydrolysis products were observed for any of the compounds during this period at room temperature and protected from light.

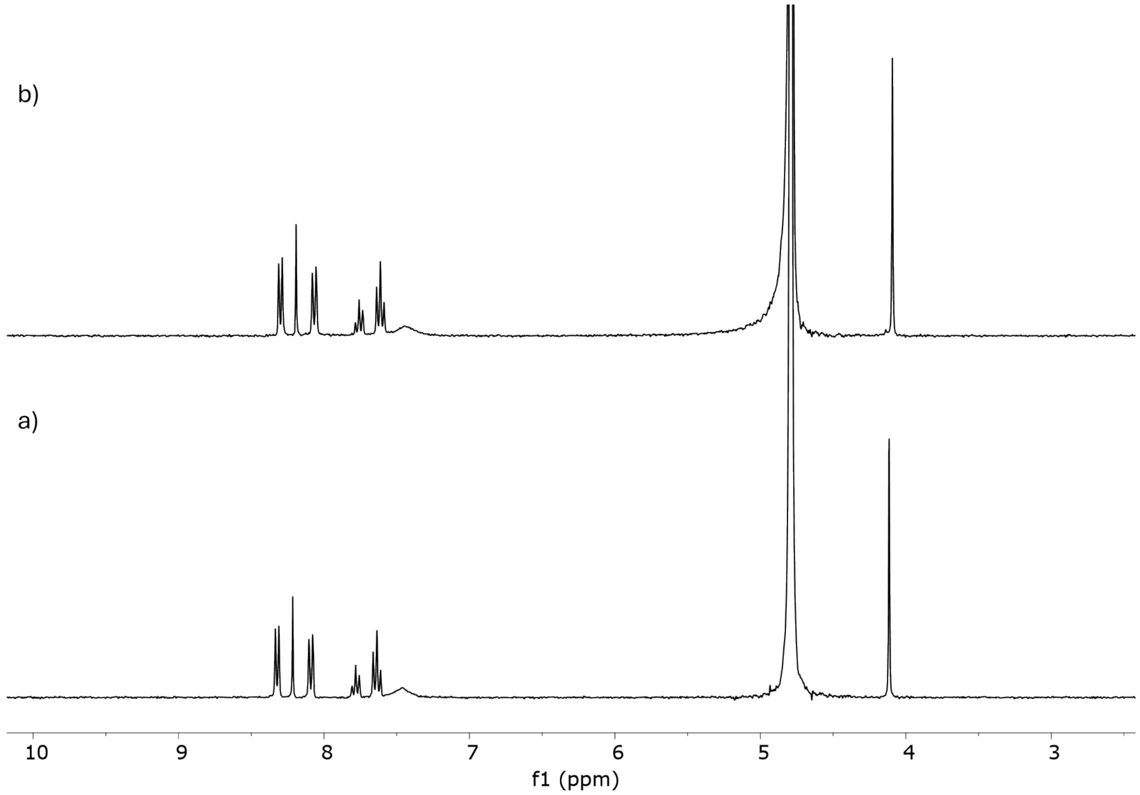

Figure S126. ^{1}H NMR stacked spectra (300 MHz, D_2O, pD 5.5) of $\mathbf{P}_a$H·Cl (5.0 mM). a) Spectrum at t = 0. b) Spectrum after seven days in solution, protected from light.

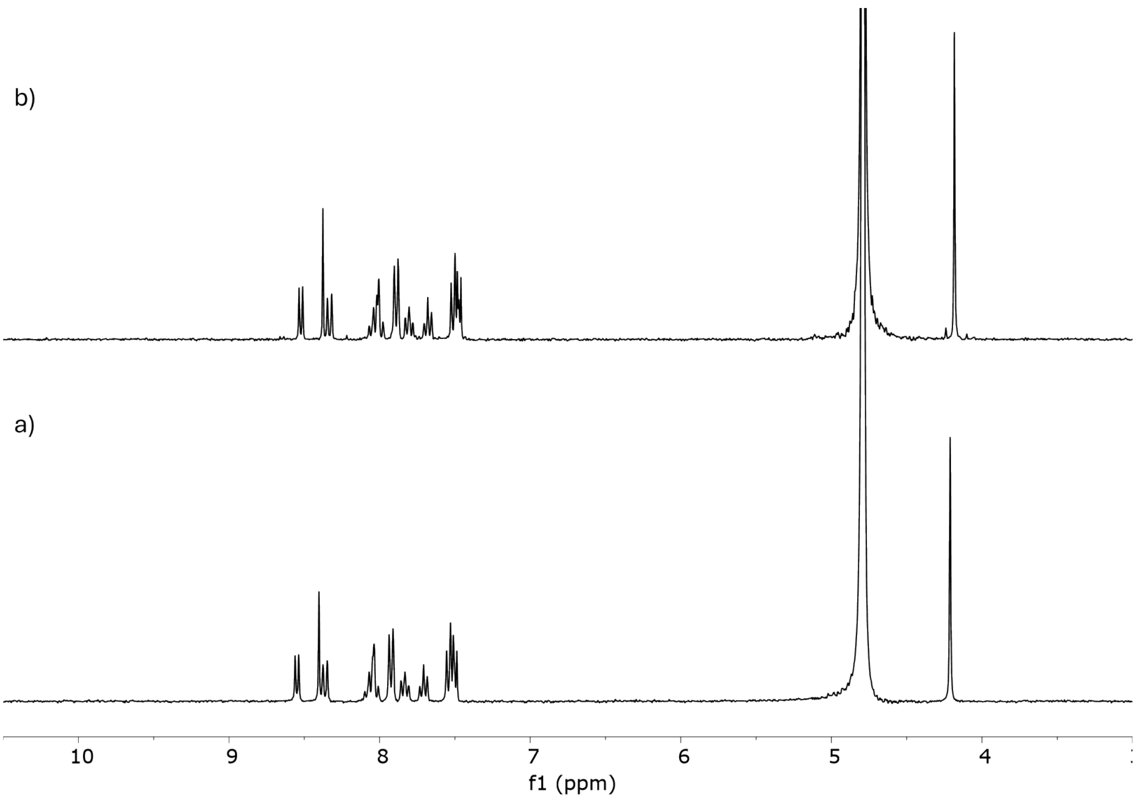

Figure S 127. ^{1}H NMR stacked spectra (300 MHz, D_2O, pD 5.5) of **P**$_b$H·Cl (5.0 mM). a) Spectrum at t = 0. b) Spectrum after seven days in solution, protected from light.

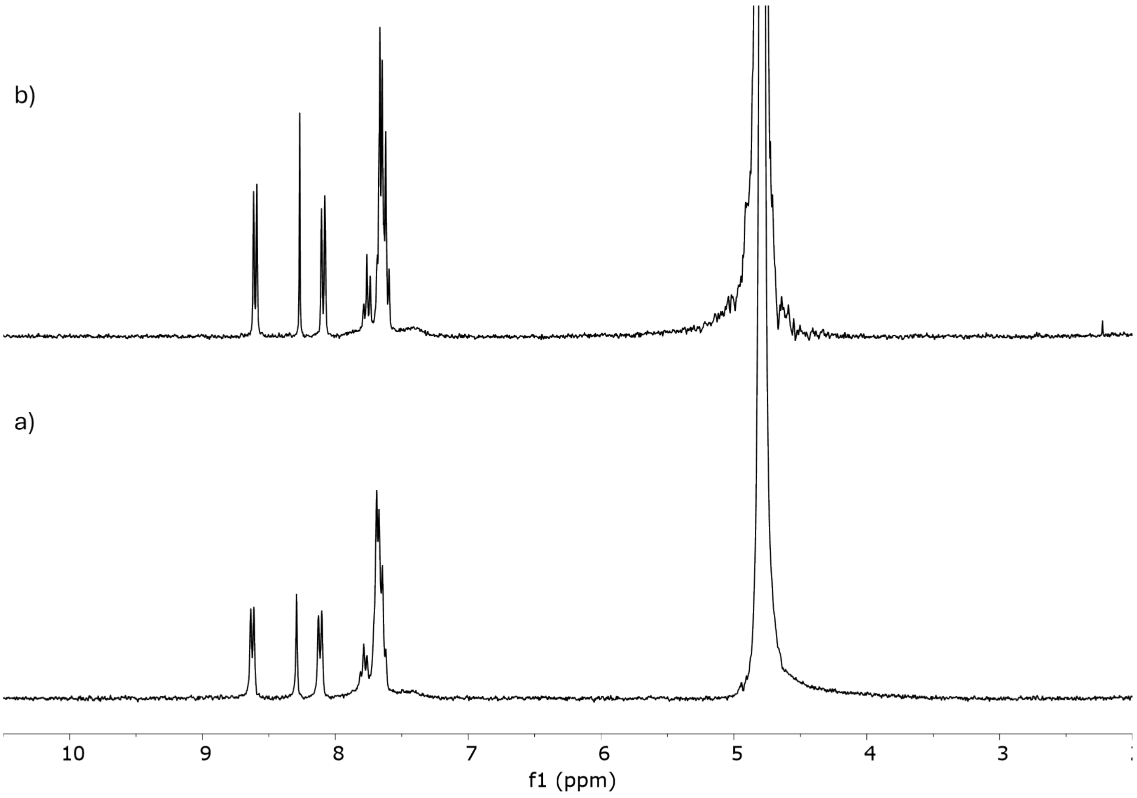

Figure S128. ^{1}H NMR stacked spectra (300 MHz, D_2O, pD 5.5) of **P**$_c$H·Cl (5.0 mM). a) Spectrum at t = 0. b) Spectrum after seven days in solution, protected from light.

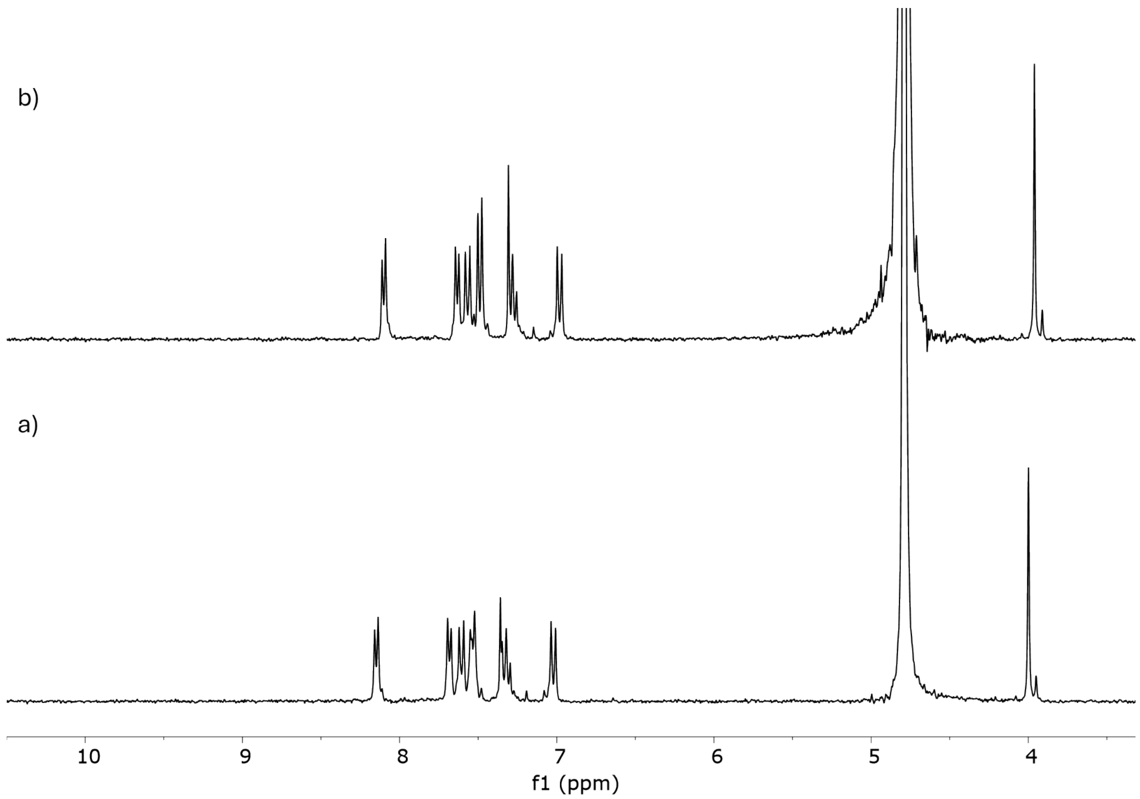

Figure S129. ^{1}H NMR stacked spectra (300 MHz, D_2O, pD 5.5) of $\mathbf{P}_d$H·Cl (5.0 mM). a) Spectrum at t = 0. b) Spectrum after seven days in solution, protected from light.

4. Determination of pK_a values for *E*-$P_{a\text{-}d}H^+$ by UV-Vis spectroscopy.

All the experiments were recorded on a Jasco V-650 spectrometer. The concentration of the compounds was kept constant at appropriate values in the 10-20 μM range, and that of the phosphate buffer at 20 mM. The absorption measurements of the corresponding air-equilibrated phosphate buffered aqueous solutions were carried out at room temperature (298 K), in quartz cuvettes with 1 cm optical pathlength. Considering the previously reported behaviour of vermellogens,[5] UV-Vis spectra were initially recorded in the 4-12 pH range, to qualitatively determine in each case the presence or absence of characteristic absorption bands for the acidic-basic forms of the compounds. From the qualitative data, UV-Vis titrations were recorded in the appropriate pH range for each compound (see below), and pK_a values derived from triplicate experiments.

[5] Barravecchia, L.; Blanco-Gómez, A.; Neira, I.; Skackauskaite, R.; Vila, A.; Rey-Rico, A.; Peinador, C.; García, M. D. ″Vermellogens″ and the development of CB[8]-based supramolecular switches using pH-responsive and non-toxic viologen analogues. *J. Am. Chem. Soc.* **2022**, *144*, 19127−19136.

4.1. E-P_aH^+.

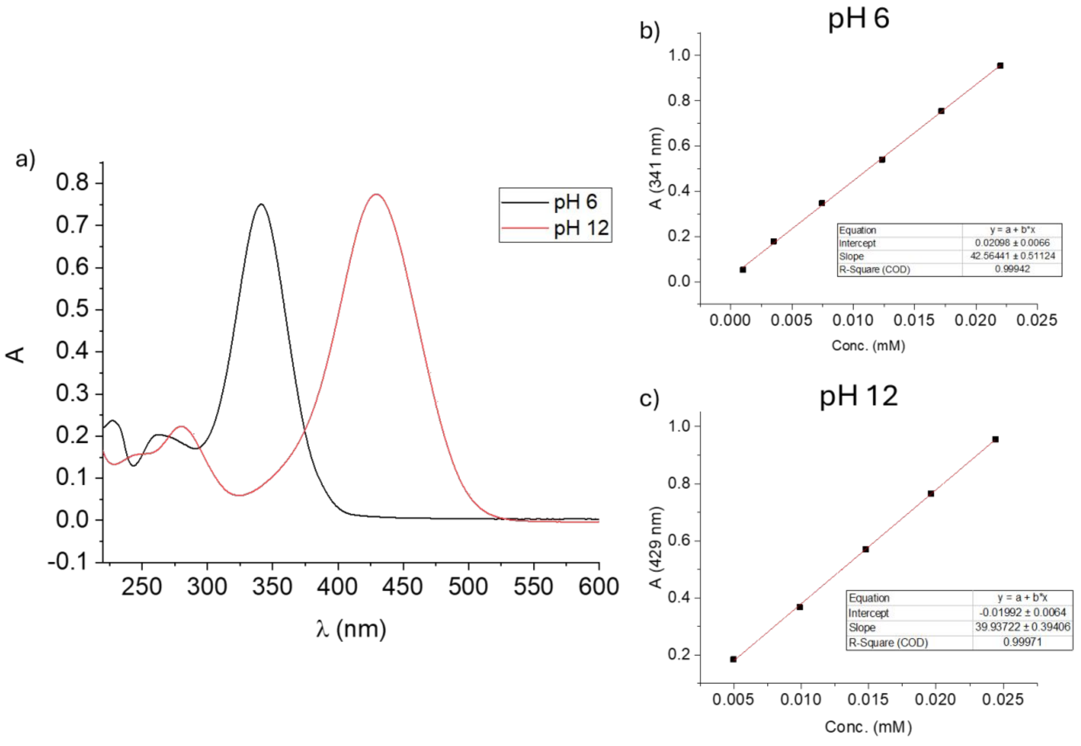

Figure S130. a) UV-Vis spectra of **P**$_a$H·Cl at pH 6 (black) and pH 11 (red) at 18 µM. b) Linear relationship at pH 6 between absorbance and concentration of **P**$_a$H·Cl (ε = 40642 ± 1838 L·mol^{-1}·cm^{-1}). c) Linear relationship at pH 12 between absorbance at 429 nm and concentration of **P**$_a$H·Cl (ε = 38820 ± 1713L·mol^{-1}·cm^{-1}).

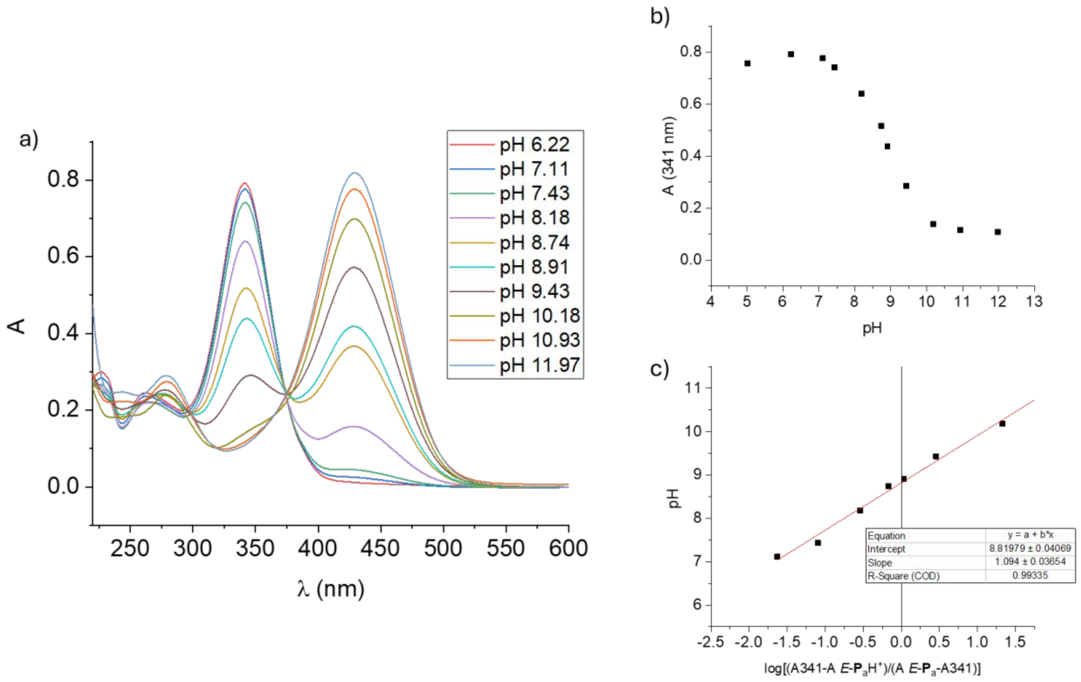

Figure S131. a) UV-Vis spectra for the titration of **P**$_a$H·Cl at 20 µM in phosphate buffers. b) Absorption of **P**$_a$H$^+$ at λ = 341 nm plotted against pH. c) Linear fitting of pH plotted against log [(A351-A **P**$_a$H$^+$)/(A **P**$_a$-A351)], where pKa mean value is 8.8 ± 0.2.

4.2. *E*-P_bH^+.

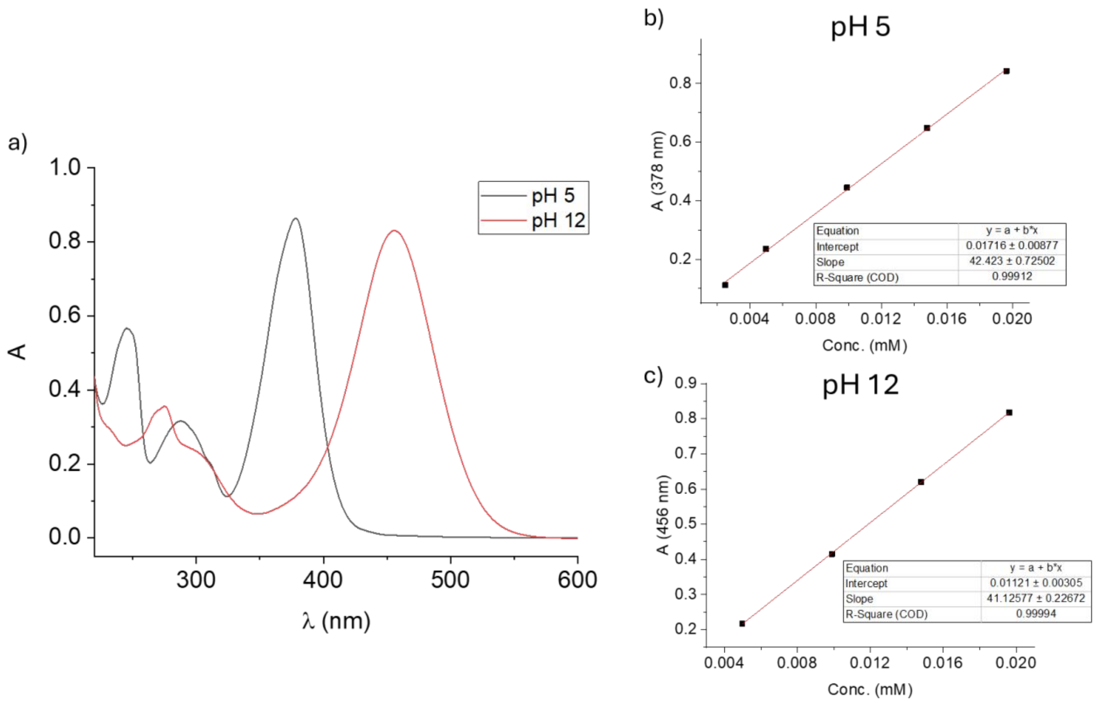

Figure S132. UV-Vis spectra of $\mathbf{P_b}$H·Cl at pH 5 (black) and pH 12 (red) at 20 µM. b) Linear relationship at pH 5 between absorbance at 378 nm and concentration of $\mathbf{P_b}$H·Cl (ε = 42265 ± 838 L·mol^{-1}·cm^{-1}). c) Linear relationship at pH 12 between absorbance at 456 nm and concentration of $\mathbf{P_b}$H·Cl (ε = 42143 ± 1044 L·mol^{-1}·cm^{-1}).

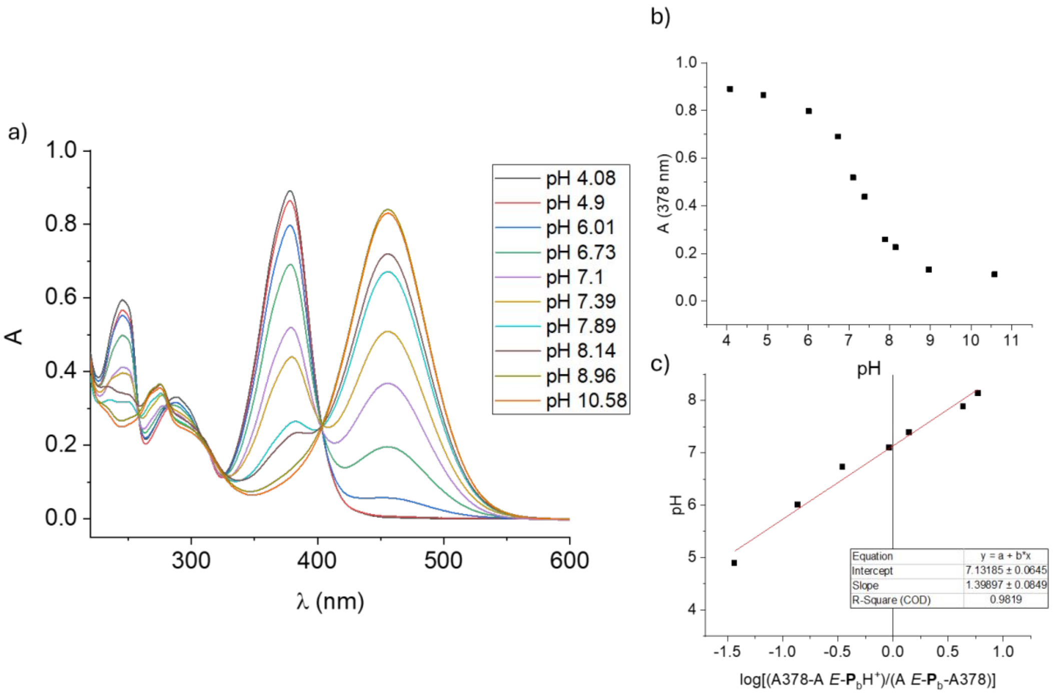

Figure S133. UV-Vis spectra for the titration of $\mathbf{P_b}$H·Cl (20 µM in phosphate buffer solutions in the 4.1-10.6 pH range). b) Absorption of $\mathbf{P_b}H^+$ at λ = 378 nm plotted against pH. c) Linear fitting of pH plotted against log [(A378-A $\mathbf{P_b}H^+$)/(A $\mathbf{P_b}$-A378)]. pK_a = 7.2±0.1 as mean value of triplicate experiments.

4.3. *E*-$\mathbf{P}_c\mathbf{H}^+$.

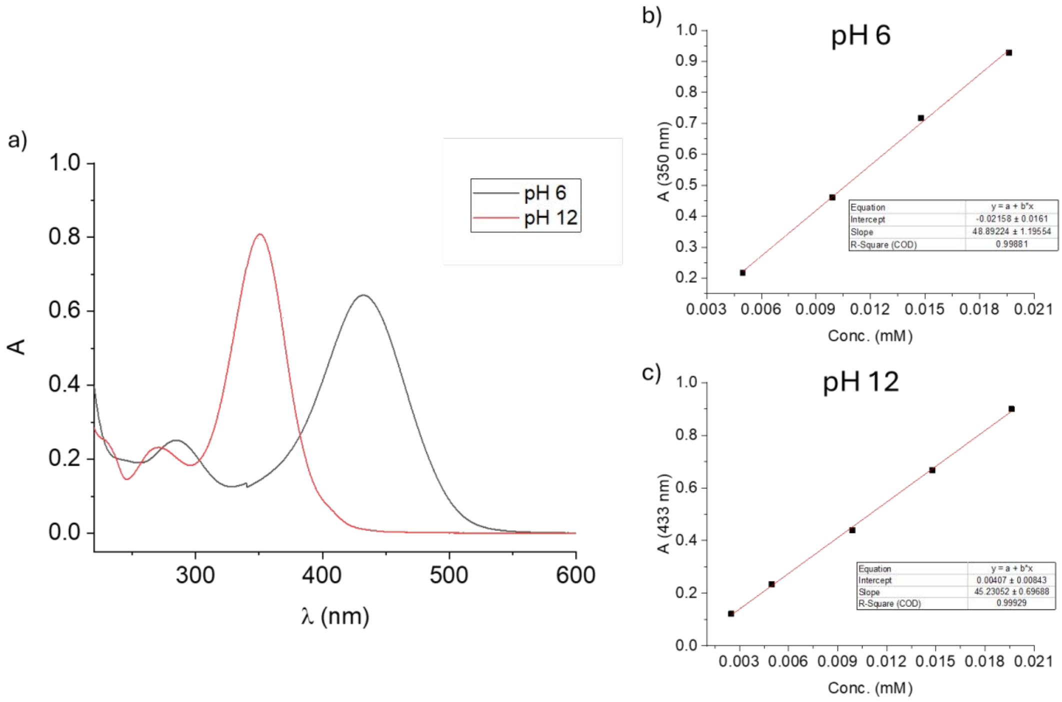

Figure S134. UV-Vis spectra of $\mathbf{P}_c$H·Cl at pH 6 (black) and pH 12 (red) at 14 µM. b) Linear relationship at pH 6 between absorbance at 350 nm and concentration of $\mathbf{P}_c$H·Cl (ε = 49266 ± 1780 L·mol^{-1}·cm^{-1}). c) Linear relationship at pH 12 between absorbance at 433 nm and concentration of $\mathbf{P}_c$H·Cl (ε = 46382 ± 1667 L·mol^{-1}·cm^{-1}).

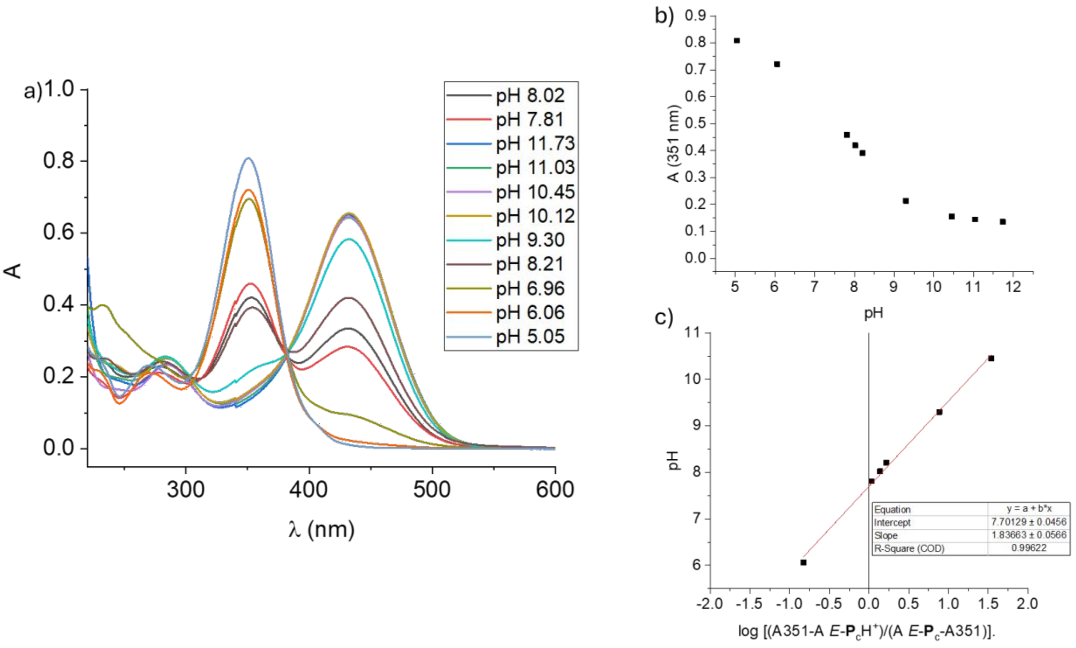

Figure S135.UV-Vis spectra for the titration of $\mathbf{P}_c$H·Cl, (14 µM in phosphate buffer solutions in the 5.1-11.7 pH range). b) Absorption of $\mathbf{P}_c$H$^+$ at λ = 351 nm plotted against pH. c) Linear fitting of pH plotted against log [(A351-A $\mathbf{P}_c$H$^+$)/(A $\mathbf{P}_c$-A351)]. pK_a = 7.8±0.1 as mean value of triplicate experiments.

4.4. *E*-$\mathbf{P_dH^+}$.

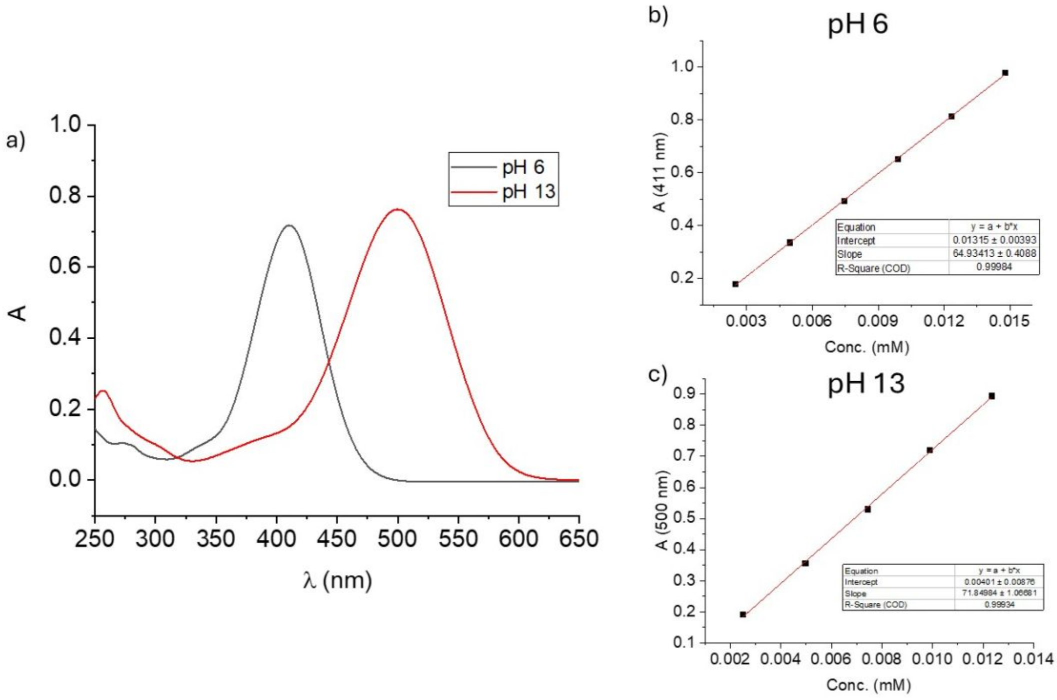

Figure S136. UV-Vis spectra of $P_dH{\cdot}Cl$ at pH 6 (red) and pH 13 (black) at 11 µM. b) Linear relationship at pH 6 between absorbance at 411 nm and concentration of $\mathbf{P_d}H{\cdot}Cl$ (ε = 65691 ± 2966 $L{\cdot}mol^{-1}{\cdot}cm^{-1}$). c) Linear relationship at pH 13 between absorbance at 500 nm and concentration of $\mathbf{P_d}H{\cdot}Cl$ (ε = 69154 ± 3100 $L{\cdot}mol^{-1}{\cdot}cm^{-1}$).

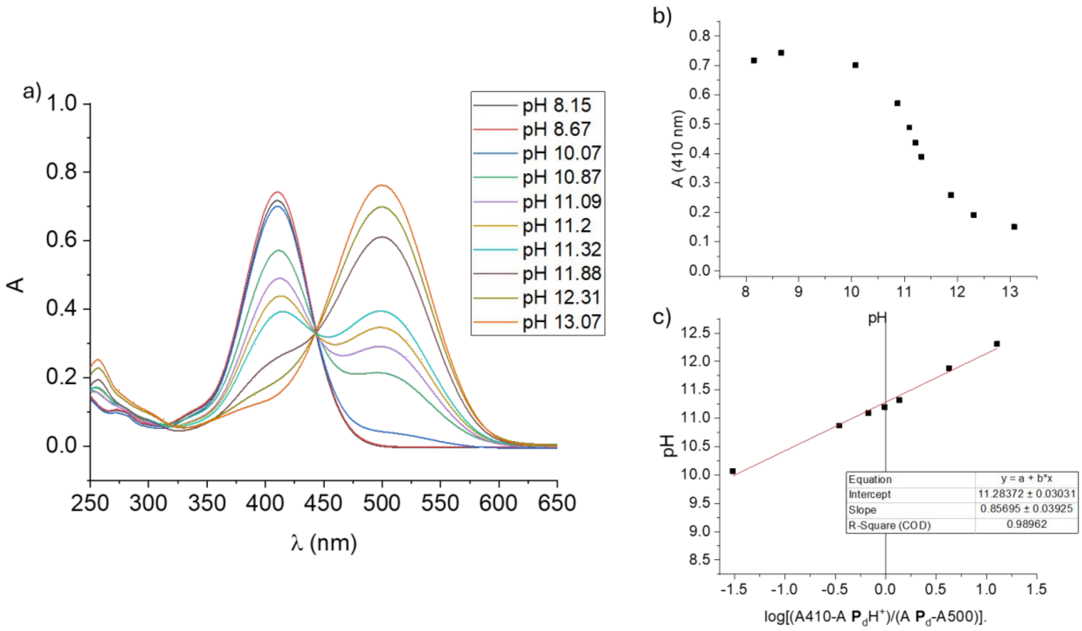

Figure S137. UV-Vis spectra for the titration of $\mathbf{P_d}H{\cdot}Cl$ (11 µM in phosphate buffer solutions in the 5.1-11.7 pH range). b) Absorption of $\mathbf{P_d}H^+$ at λ = 410 nm plotted against pH. c) Linear fitting of pH plotted against log [(A410-A$\mathbf{P_d}H^+$)/(A$\mathbf{P_d}$-A410)]. pK_a = 11.2±0.1 as mean value of triplicate experiments.

5. Isomerization Photochemical Data.

5.1. E-P$_{a\text{-}c}$H$^+$→Z-P$_{a\text{-}c}$H$^+$ photoisomerization kinetics.

5.1.1. E-P$_a$H$^+$→Z-P$_a$H$^+$

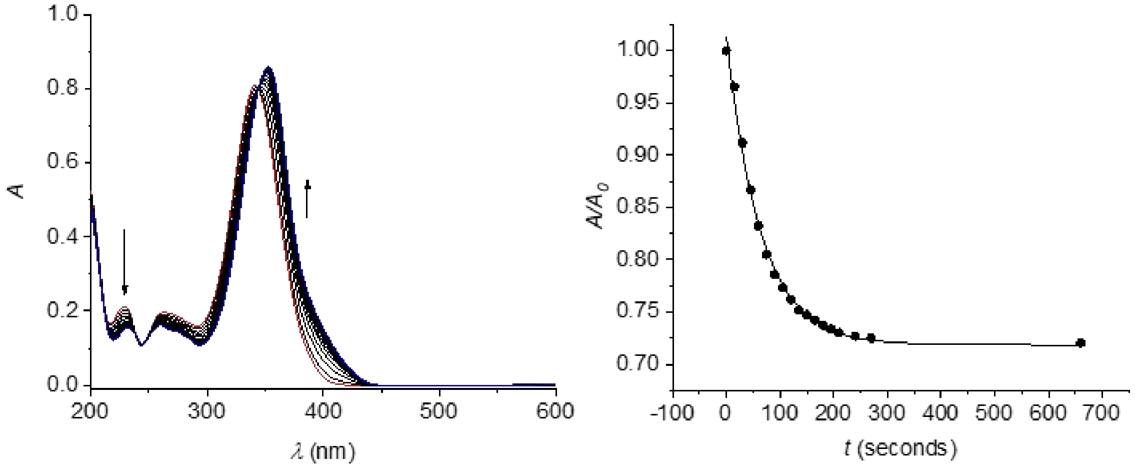

Figure S138. Left: variations in the absorption spectrum of **P**$_a$H·Cl (20.6 μM, pH = 6) upon irradiation at 254 nm; the absorption spectrum of the E-form is shown in red; the photostationary state is in blue. Right: Kinetics (monitored at 320 nm) for the E-**P**$_a$H$^+$ to Z-**P**$_a$H$^+$ photoisomerization (20.6 μM) upon irradiation at 254 nm (I_0 = 1.37 × 10^{-7} mol/s); k = 1.55 × 10^{-2} s^{-1}. $\Phi_{E\to Z}$ = 0.20.

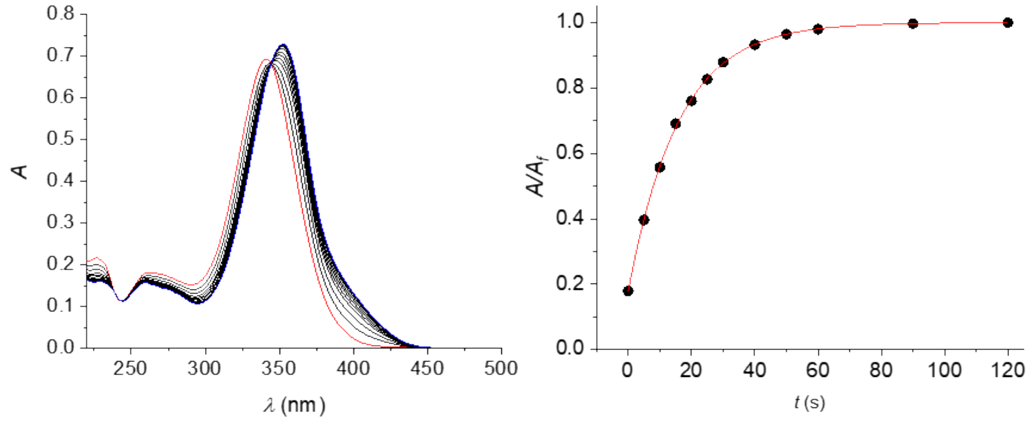

Figure S139. Left: variations in the absorption spectrum of **P**$_a$H·Cl (17.5 μM, pH = 6) upon irradiation at 365 nm; the absorption spectrum of the E-form is shown in red; the photostationary state is in blue. Right: Kinetics (monitored at 400 nm) for the E-**P**$_a$H$^+$ to Z-**P**$_a$H$^+$ photoisomerization (17.5 μM) upon irradiation at 365 nm (I_0 = 2.6 × 10^{-7} mol/s); k = 6.4 × 10^{-2} s^{-1}. $\Phi_{E\to Z}$ = 0.22.

5.1.2. E-$P_bH^+ \rightarrow Z$-P_bH^+

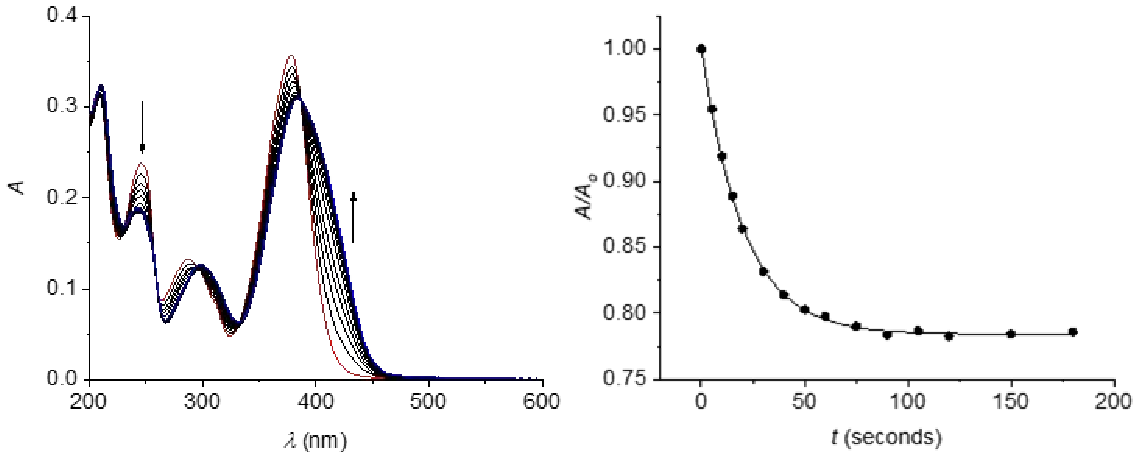

Figure S140. Left: variations in the absorption spectrum of $\mathbf{P}_b$H·Cl (7.9 µM, pH = 5) upon irradiation at 254 nm; the absorption spectrum of the E-form is shown in red; the photostationary state is in blue. Right: Kinetics (monitored at 370 nm) for the E-$\mathbf{P}_b H^+$ to Z-$\mathbf{P}_b H^+$ photoisomerization (7.9 µM) upon irradiation at 254 nm ($I_0 = 1.41 \times 10^{-7}$ mol/s); $k = 4.91 \times 10^{-2}$ s^{-1}. $\Phi_{E\rightarrow Z} = 0.38$.

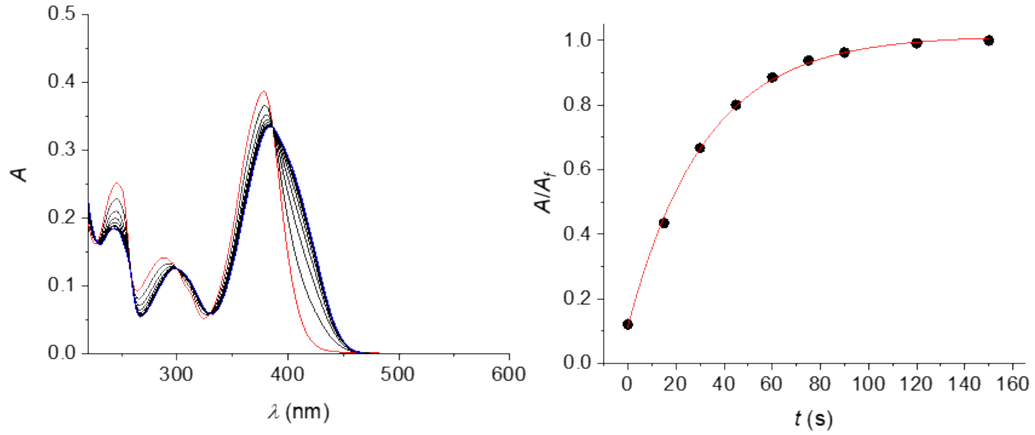

Figure S141. Left: variations in the absorption spectrum of $\mathbf{P}_b$H·Cl (10 µM, pH = 5) upon irradiation at 365 nm; the absorption spectrum of the E-form is shown in red; the photostationary state is in blue. Right: Kinetics (monitored at 420 nm) for the E-$\mathbf{P}_b H^+$ to Z-$\mathbf{P}_b H^+$ photoisomerization (10 µM) upon irradiation at 365 nm ($I_0 = 2.0 \times 10^{-7}$ mol/s); $k = 3.0 \times 10^{-2}$ s^{-1}. $\Phi_{E\rightarrow Z} = 0.35$.

5.1.3. *E*-P_cH^+→*Z*-P_cH^+

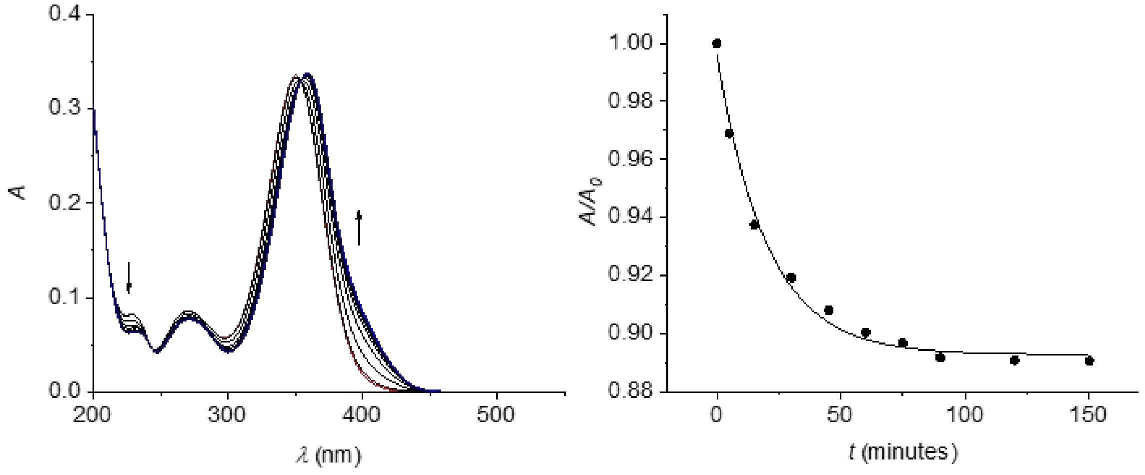

Figure S142. Left: variations in the absorption spectrum of **P**$_c$H·Cl (9 μM, pH = 6) upon irradiation at 254 nm; the absorption spectrum of the *E*-form is shown in red; the photostationary state is in blue. Right: Kinetics (monitored at 377 nm) of the *E*-**P**$_c$H$^+$ to *Z*-**P**$_c$H$^+$ photoisomerization (9 μM) upon irradiation at 254 nm ($I_0 = 1.39 \times 10^{-7}$ mol/s); $k = 4.90 \times 10^{-2}$ s^{-1}. $\Phi_{E\rightarrow Z} = 0.34$.

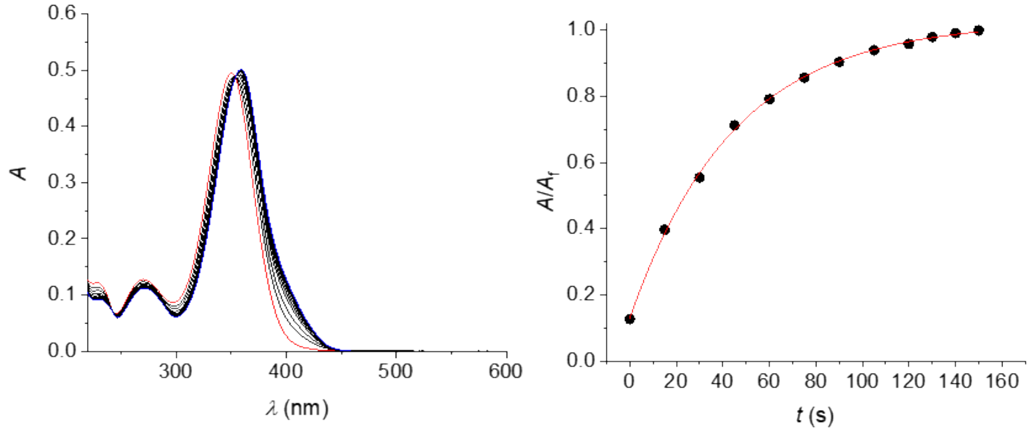

Figure S143. Left: variations in the absorption spectrum of **P**$_c$H·Cl (10 μM, pH = 6) upon irradiation at 365 nm; the absorption spectrum of the *E*-form is shown in red; the photostationary state is in blue. Right: Kinetics (monitored at 410 nm) of the *E*-**P**$_c$H$^+$ to *Z*-**P**$_c$H$^+$ photoisomerization (10 μM) upon irradiation at 365 nm ($I_0 = 2.4 \times 10^{-7}$ mol/s); $k = 2.0 \times 10^{-2}$ s^{-1}. $\Phi_{E\rightarrow Z} = 0.34$.

5.1.4. *E*-P_dH$^+$→*Z*-P_dH$^+$

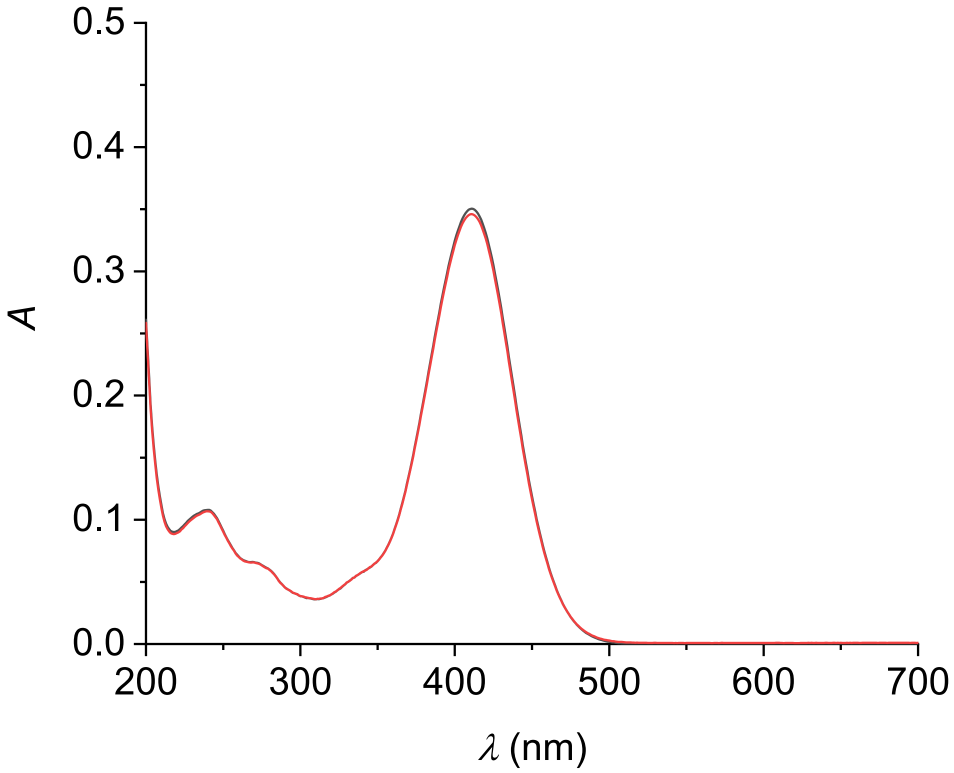

Figure S144. Absorption spectrum changes during irradiation at 254 nm of **P**$_d$H·Cl (7 µM, pH = 6). initial absorption spectrum in black; photostationary-state spectrum after irradiation in red. No changes were observed during irradiation at 254 nm or 365 nm.

5.2. *Z*-$\mathbf{P}_{a\text{-}c}\mathbf{H}^+$→*E*-$\mathbf{P}_{a\text{-}c}\mathbf{H}^+$ photoisomerization kinetics.

5.2.1. *Z*-$\mathbf{P}_a\mathbf{H}^+$→*E*-$\mathbf{P}_a\mathbf{H}^+$

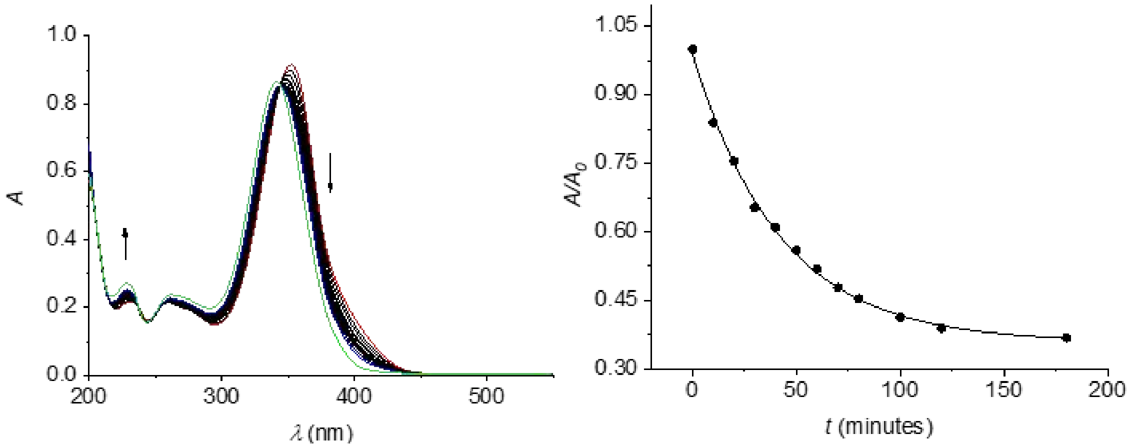

Figure S145. Left: variations in the absorption spectrum of $\mathbf{P}_a$H·Cl (20.6 µM, pH = 6) upon irradiation at > 420 nm (long-pass filter); the absorption spectrum of the 254 nm photostationary state (mainly *Z*- form) is shown in red; the absorption spectrum of the photostationary state after irradiation at >420 nm is presented in blue; the absorption spectrum of the pure *E*-isomer is shown in green. Right: Kinetics (monitored at 420 nm) of the *Z*-$\mathbf{P}_a$H$^+$ to *E*-$\mathbf{P}_a$H$^+$ photoisomerization (20.6 µM) upon irradiation at > 420 nm; $k = 3.95 \times 10^{-4}$ s^{-1}. $\Phi_{Z \to E} < 0.005$ (440-nm irradiation).

5.2.2. *Z*-$\mathbf{P}_b\mathbf{H}^+$→*E*-$\mathbf{P}_b\mathbf{H}^+$

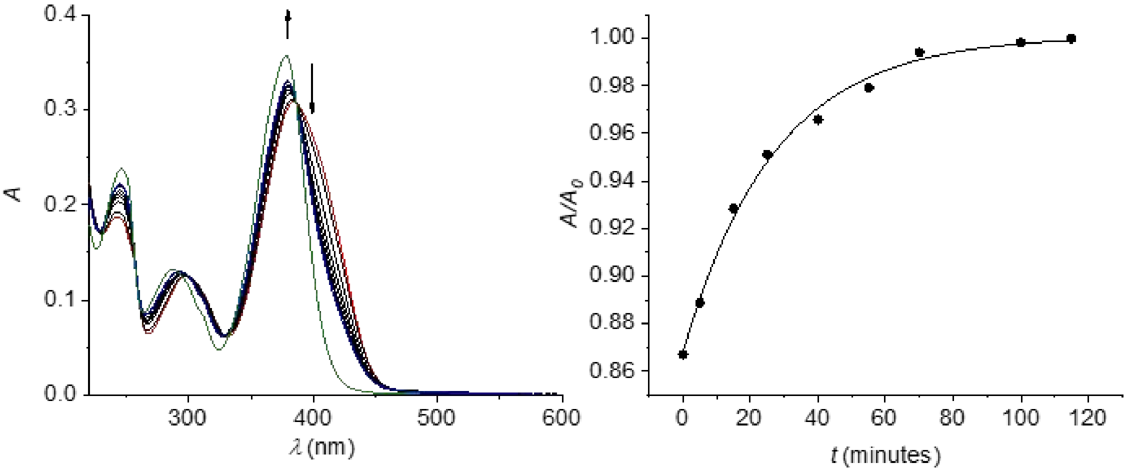

Figure S146. Left: variations in the absorption spectrum of $\mathbf{P}_b$H·Cl (7.9 µM, pH = 5) upon irradiation at >420 nm (long-pass filter); the absorption spectrum of the 254 nm photostationary state (mainly *Z* form) is shown in red; the absorption spectrum of the photostationary state after irradiation at >420 nm is presented in blue; the absorption spectrum of the pure *E*- isomer is shown in green. Right: Kinetics (monitored at 370 nm) of the *Z*-$\mathbf{P}_b$H$^+$ to *E*-$\mathbf{P}_b$H$^+$ photoisomerization (7.9 µM) upon irradiation at > 420 nm; $k = 6.17 \times 10^{-4}$ s^{-1}. $\Phi_{Z \to E} < 0.005$ (440-nm irradiation).

5.2.3. Z-$P_cH^+ \rightarrow E$-P_cH^+

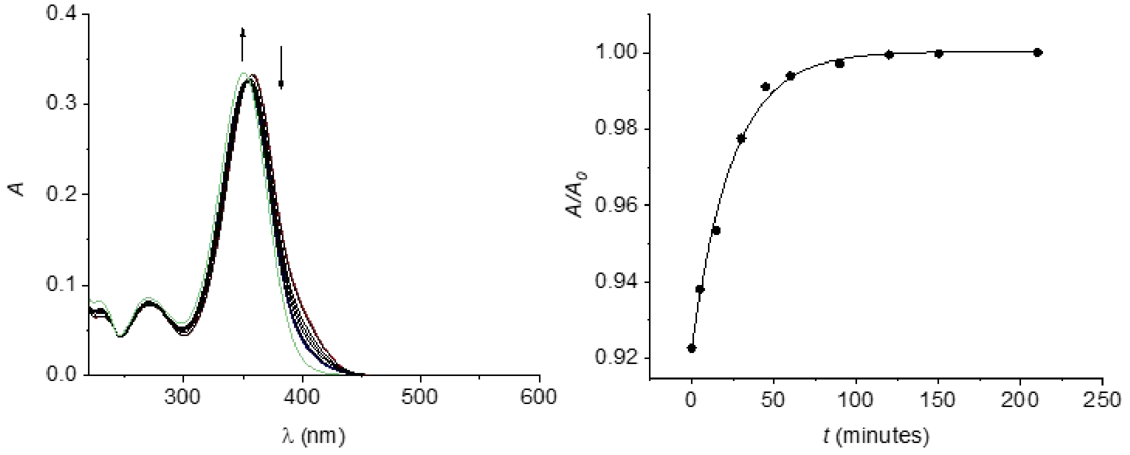

Figure S147. Left: variations in the absorption spectrum of **P**$_c$H·Cl (9.0 μM, pH = 6) upon irradiation at >420 nm (long-pass filter); the absorption spectrum of the 254 nm photostationary state (mainly *Z*- form) is shown in red; the absorption spectrum of the photostationary state after irradiation at >420 nm is presented in blue; the absorption spectrum of the pure *E* form is shown in green. Right: Kinetics (monitored at 351 nm) of the *Z*-**P**$_c$H$^+$ to *E*-**P**$_c$H$^+$ photoisomerization (9.0 μM) upon irradiation at > 420 nm; $k = 1.85 \times 10^{-3}$ s^{-1}. $\Phi_{Z\rightarrow E} = 0.008$ (440-nm irradiation).

5.3. Fatigue resistance.

Fatigue resistance experiments were performed using solutions of the respective photoswitches. Each sample was irradiated at 254 nm for 5 minutes to ensure complete isomerization from the *E*-form to the *Z*-form. The reverse isomerization (*Z* to *E*) was achieved by irradiating at >440 nm for 10 minutes. This process was repeated for each cycle.

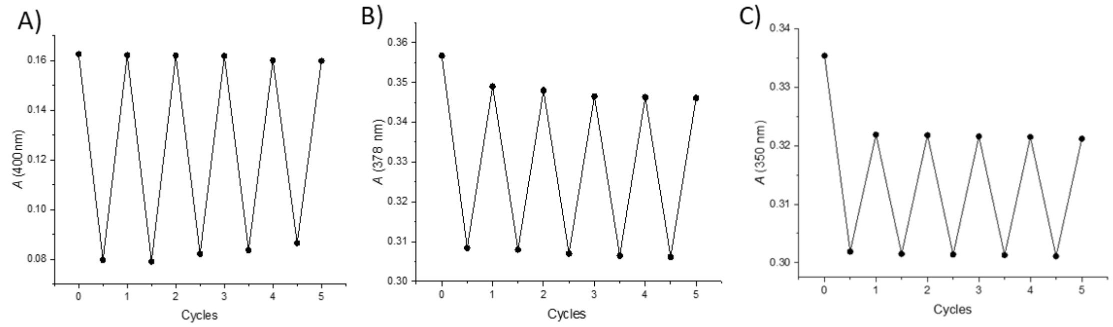

Figure S148. A) Absorbance changes at 400 nm during repeated photoisomerization cycles (alternating irradiation at > 420 nm with irradiation at 254 nm) of $\mathbf{P}_a$H·Cl (20.6 µM, pH 6). B) Absorbance changes at 378 nm during repeated photoisomerization cycles (alternating irradiation at >420 nm with irradiation at 254 nm) of $\mathbf{P}_b$H·Cl (7.9 µM, pH 5). C) Absorbance changes at 350 nm during repeated photoisomerization cycles (alternating irradiation at > 420 nm with irradiation at 254 nm) of $\mathbf{P}_c$H·Cl (9.0 µM, pH 6).

5.4. Photostationary state distributions.

5.4.1. $P_aH{\cdot}Cl$

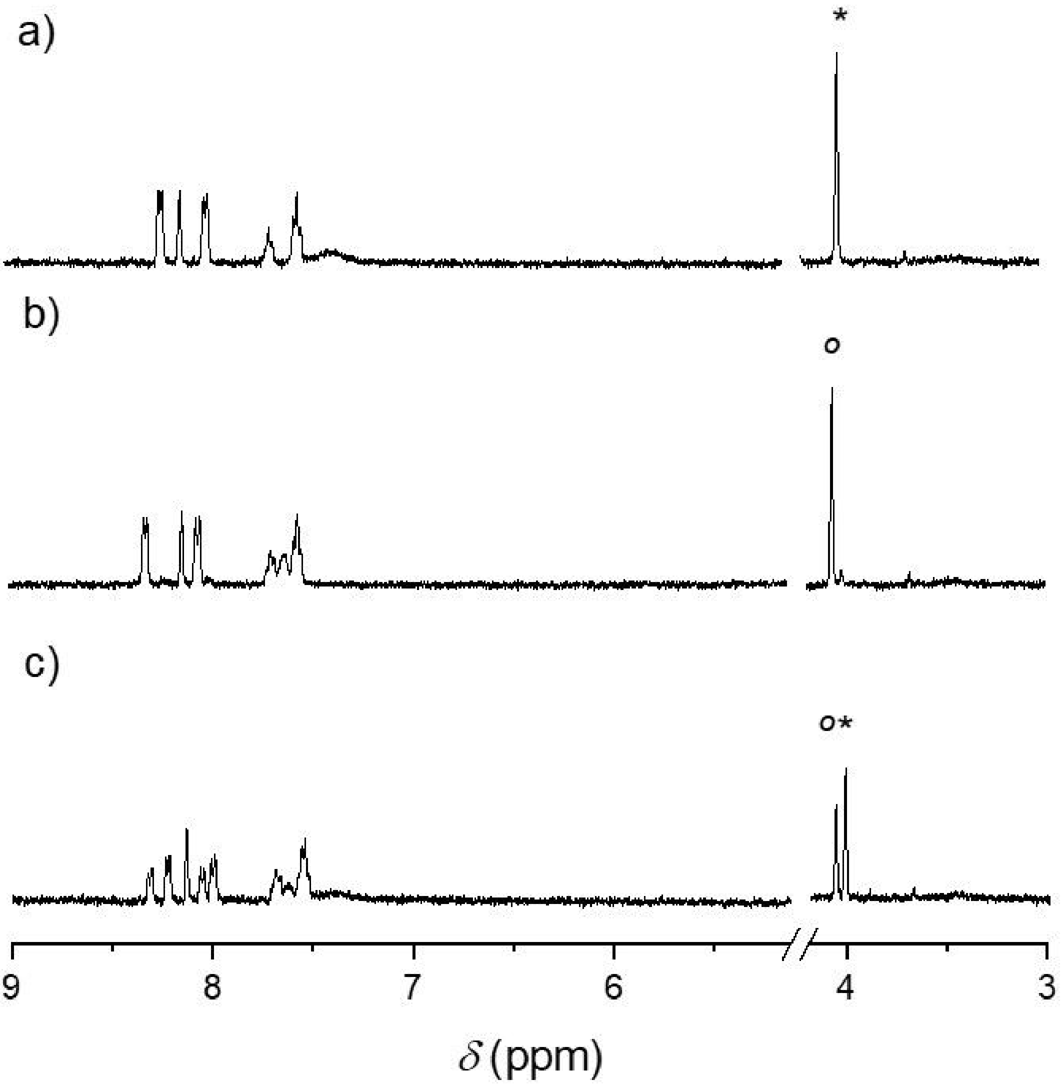

Figure S149. Partial ^1H NMR stacked spectra (400 MHz, D_2O, pD 6) of $\mathbf{P}_aH{\cdot}Cl$ (1.0 mM). a) Initial spectrum of the *E*-isomer, b) spectrum upon irradiation at 254 nm until reaching the photostationary state PSS (corresponding to 93% *Z*-isomer), and c) the spectrum upon isomerization reversion by irradiation at >420 nm, yielding back 67% of the E-isomer. (*) highlights the signal selected for quantify *E*-isomer. (º) highlights the signal selected for quantify *Z*-isomer.

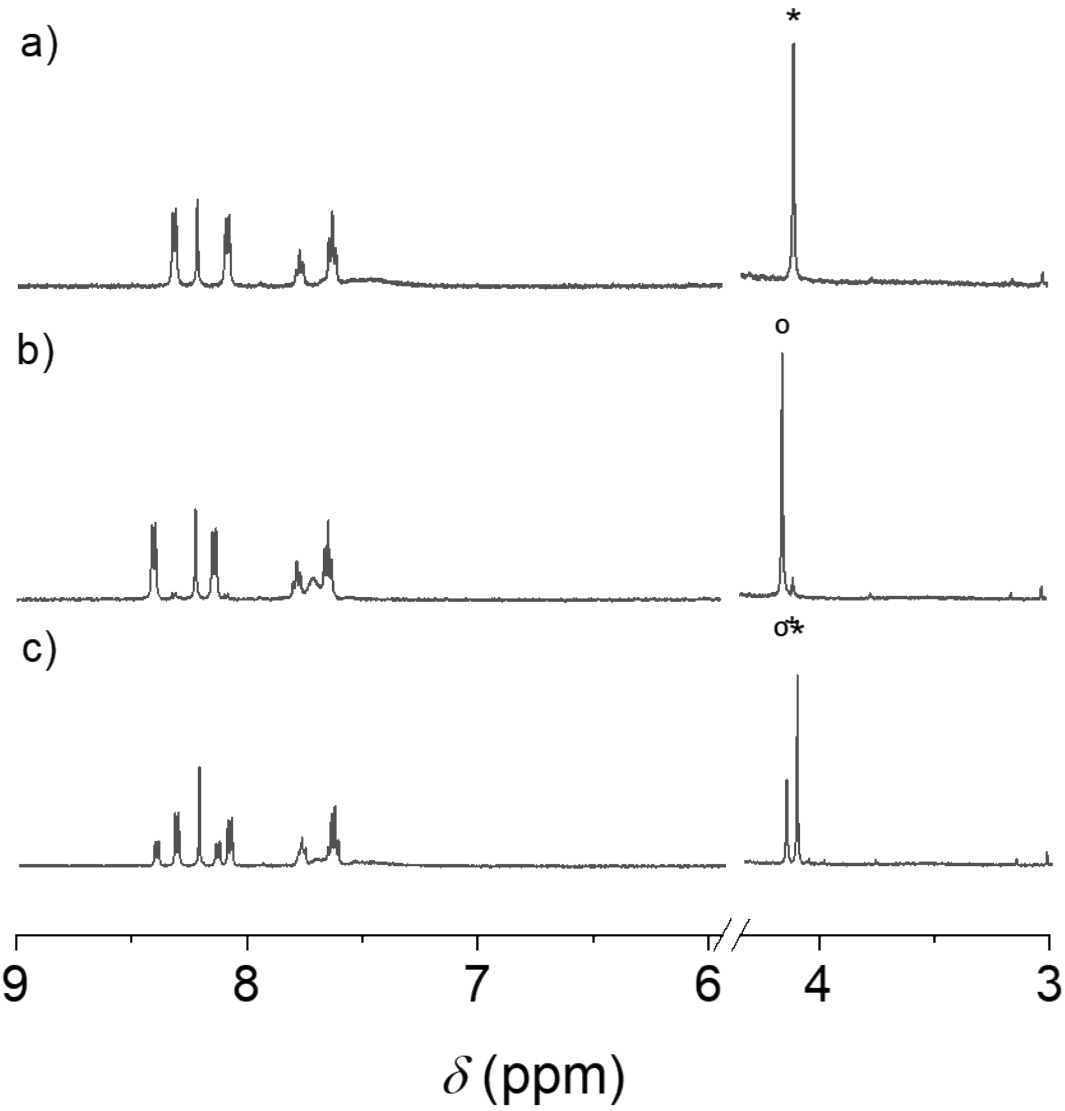

Figure S150. Partial ^{1}H NMR stacked spectra (500 MHz, D_2O, pD 6) of $\mathbf{P}_a$H·Cl (1.0 mM). a) Initial spectrum of the *E*-isomer, b) spectrum upon irradiation at 365 nm until reaching the photostationary state PSS (corresponding to 87% *Z*-isomer), and c) the spectrum upon isomerization reversion by irradiation at >420 nm, yielding back 66% of the E-isomer. (*) highlights the signal selected for quantify *E*-isomer. (º) highlights the signal selected for quantify *Z*-isomer.

5.4.2. $P_bH·Cl$

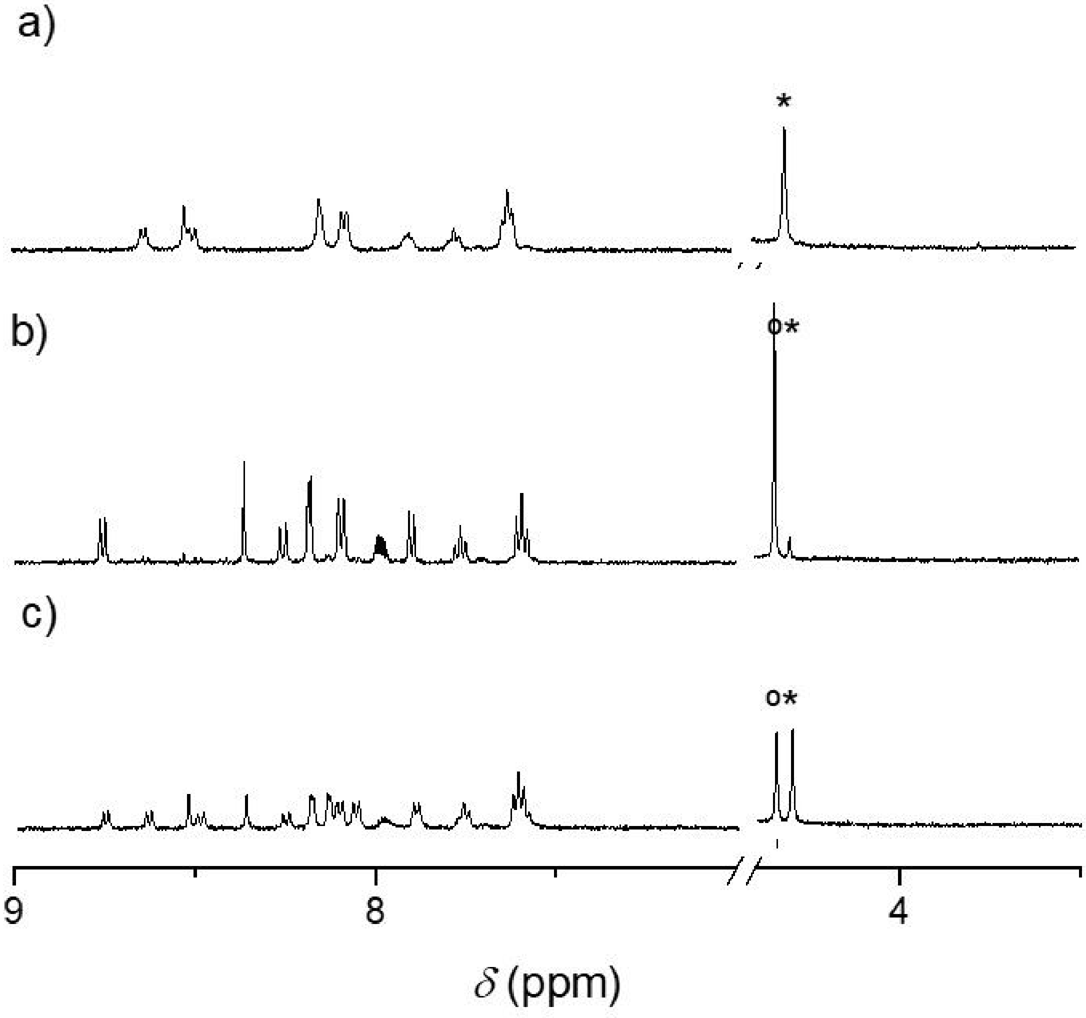

Figure S151. Partial ^1H NMR stacked spectra (400 MHz, D_2O, pD 6) of $\mathbf{P_b}$H·Cl (1.0 mM). a) Initial spectrum of the *E*-isomer, b) spectrum upon irradiation at 254 nm until reaching the photostationary state PSS (corresponding to 93% *Z*-isomer), and c) the spectrum upon isomerization reversion by irradiation at > 420 nm, yielding back 57% of the *E*-isomer. (*) highlights the signal selected for quantify *E*-isomer. (º) highlights the signal selected for quantify *Z*-isomer.

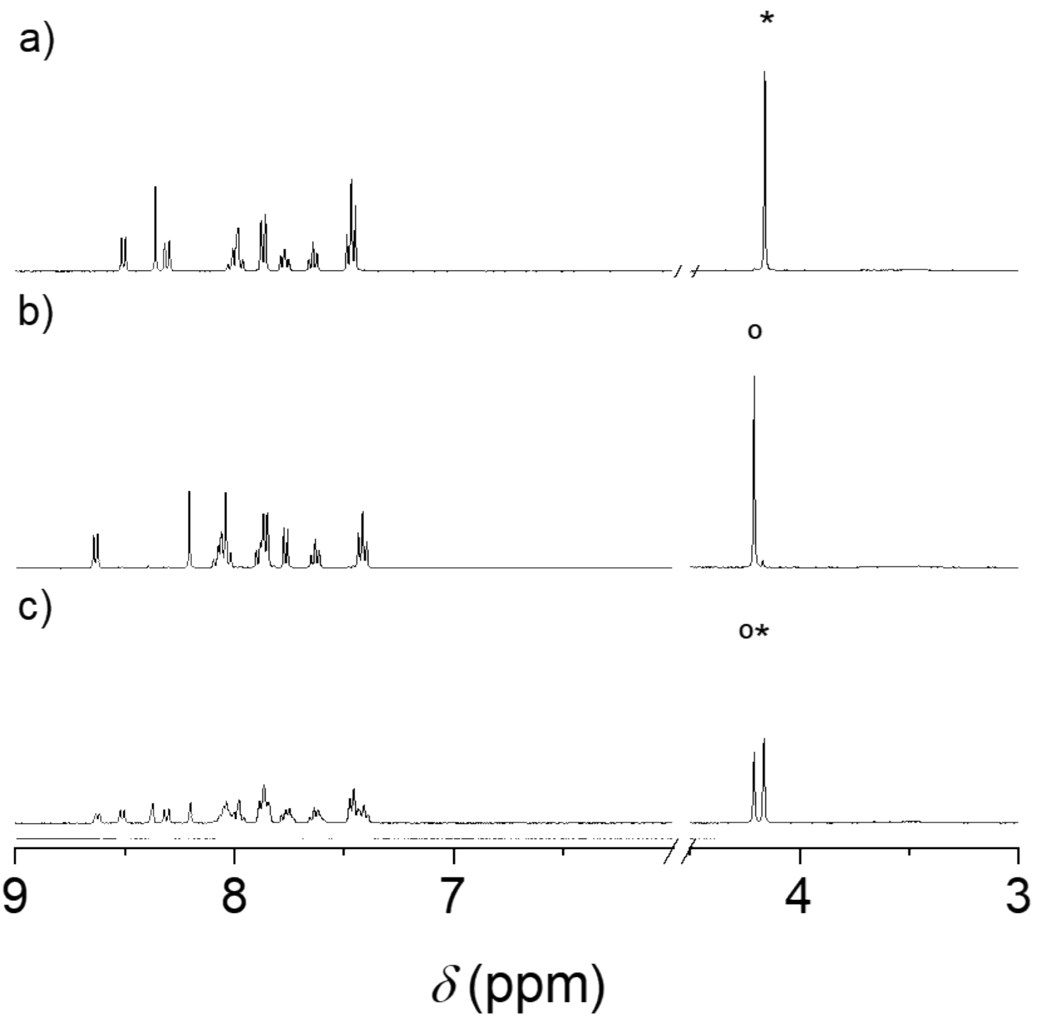

Figure S152. Partial ^{1}H NMR stacked spectra (500 MHz, D_2O, pD 6) of $\mathbf{P}_b$H·Cl (1.0 mM). a) Initial spectrum of the *E*-isomer, b) spectrum upon irradiation at 365 nm until reaching the photostationary state PSS (corresponding to 92% *Z*-isomer), and c) the spectrum upon isomerization reversion by irradiation at > 420 nm, yielding back 55% of the *E*-isomer. (*) highlights the signal selected for quantify *E*-isomer. (º) highlights the signal selected for quantify *Z*-isomer.

5.4.3. P_cH·Cl

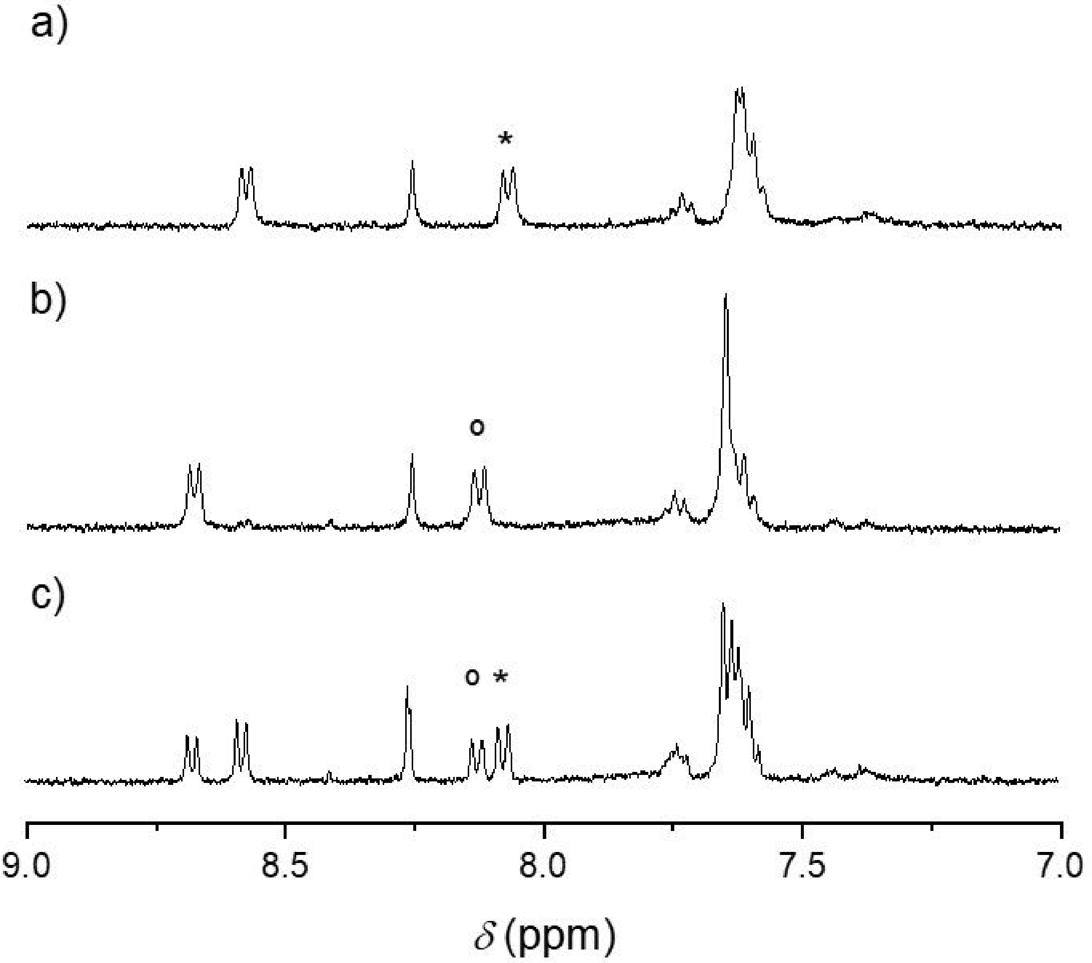

Figure S153. Partial ^{1}H NMR stacked spectra (400 MHz, D_2O, pD 6) of **P**$_c$H·Cl (1.1 mM). a) Initial spectrum of the *E*-isomer, b) spectrum upon irradiation at 254 nm until reaching the photostationary state PSS (corresponding to 98% *Z*-isomer), and c) the spectrum upon isomerization reversion by irradiation at > 420 nm, yielding back 55% of the *E*-isomer. (*) highlights the signal selected for quantify *E*-isomer. (º) highlights the signal selected for quantify *Z*-isomer.

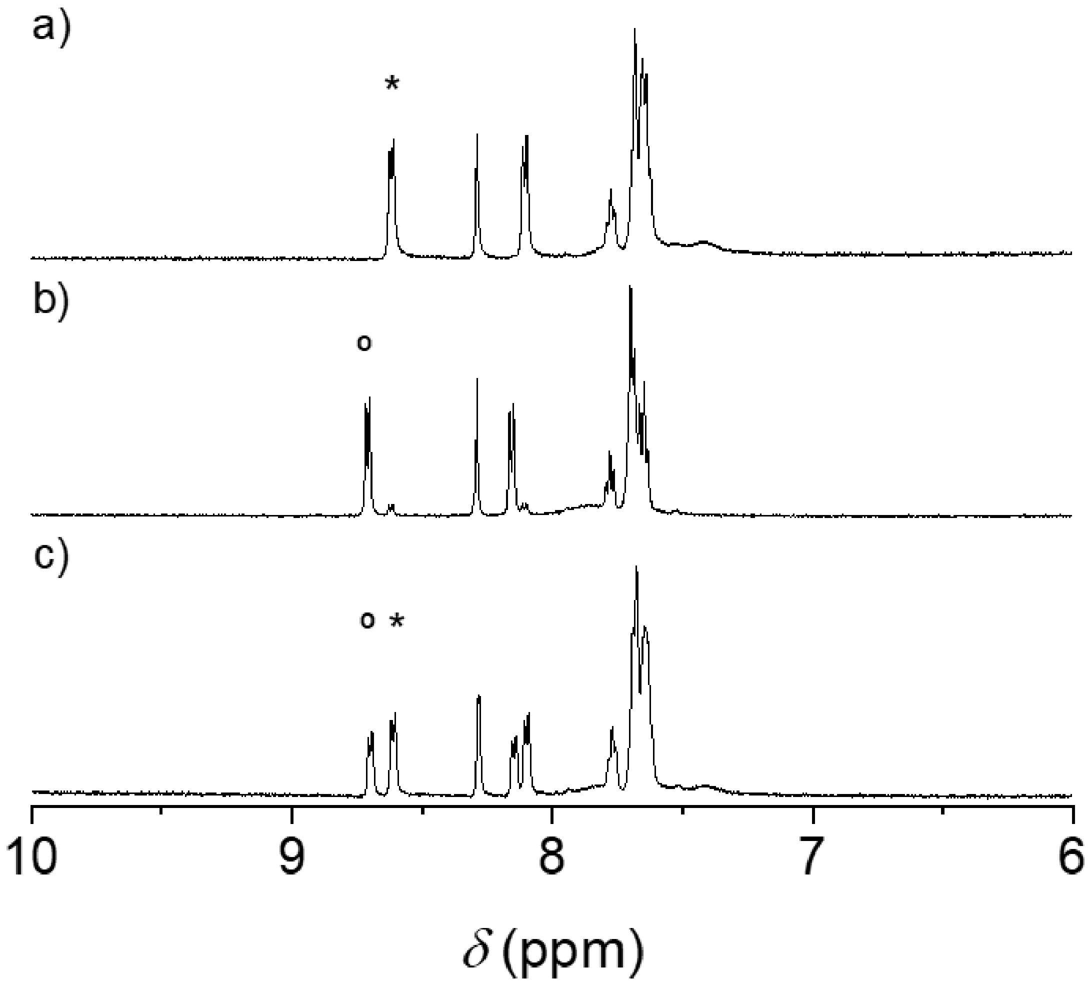

Figure S154. Partial ^{1}H NMR stacked spectra (500 MHz, D_2O, pD 6) of $\mathbf{P}_c$H·Cl (1.0 mM). a) Initial spectrum of the *E*-isomer, b) spectrum upon irradiation at 365 nm until reaching the photostationary state PSS (corresponding to 93% *Z*-isomer), and c) the spectrum upon isomerization reversion by irradiation at > 420 nm, yielding back 60% of the *E*-isomer. (*) highlights the signal selected for quantify *E*-isomer. (º) highlights the signal selected for quantify *Z*-isomer.

5.5. Z-$P_{a\text{-}c}H^+$→E-$P_{a\text{-}c}H^+$ thermal back-isomerization kinetics.

5.5.1. Z-P_aH^+→E-P_aH^+

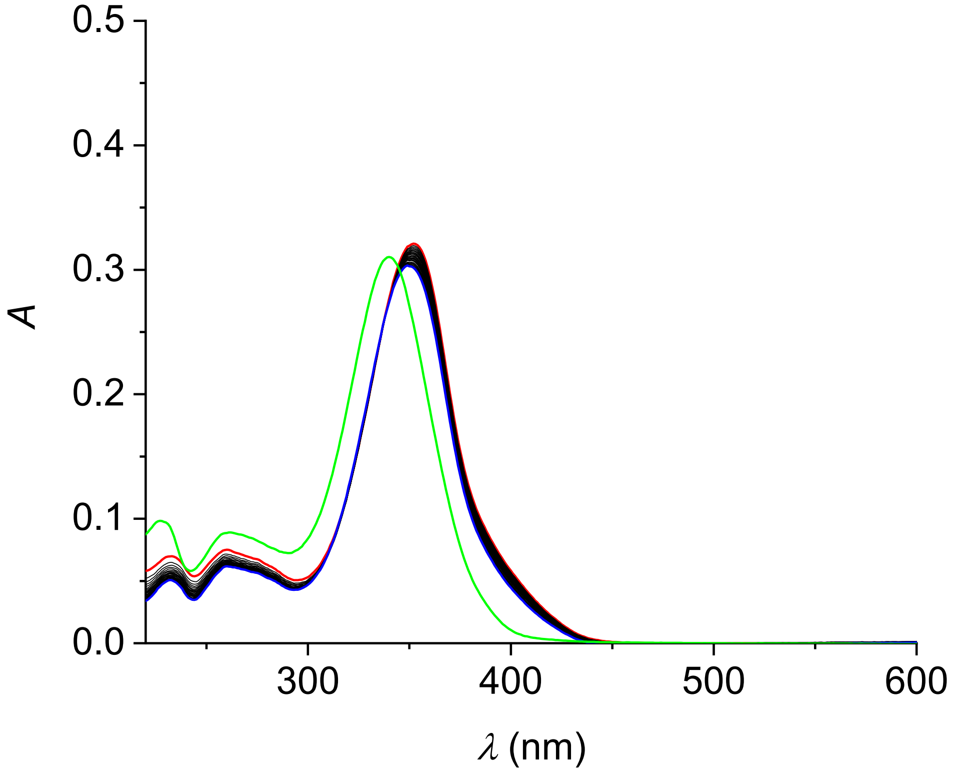

Figure S155. Absorption spectrum variation during 48h at room temperature from the PSS reached for $\mathbf{P}_a$H·Cl (7µM, pH 6, Red) state during 48h. *E*-isomer absorption spectrum in green. Even though the kinetics has not been fully completed, it can be observed that it occurs much more slowly than photochemical processes.

To obtain an estimation of $\mathbf{P}_a$H·Cl *Z*→*E* thermal half-live at room temperature, the *Z*→*E* thermal reversion kinetics were monitored starting from the 254nm photostationary state at different temperatures (47, 55 and 64ºC). By tracking the absorbance changes at 420 nm, an Arrhenius analysis was performed, allowing the determination of the activation energy (E_A) and the estimation of the thermal half-life at 25ºC.

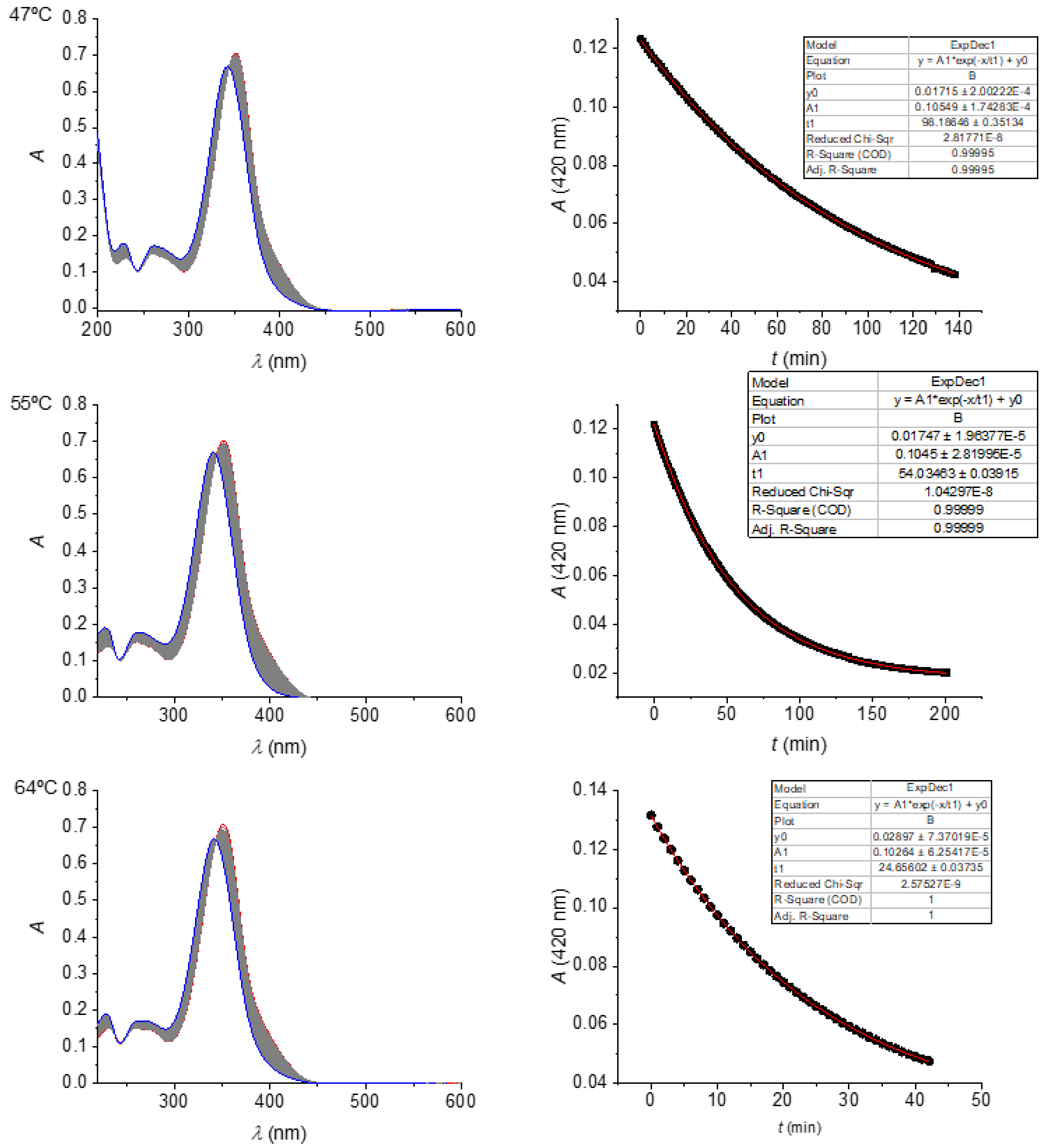

Figure S156. Left: variations in the absorption spectrum of *Z*-$\mathbf{P}_a$H·Cl (20 μM, pH 6) at 47, 55, and 64 ºC; the absorption spectrum of the *Z* form is shown in red; the final spectrum is shown in blue. Right: Kinetics for the thermal isomerization of *Z*-$\mathbf{P}_a H^+$ to *E*-$\mathbf{P}_a H^+$.

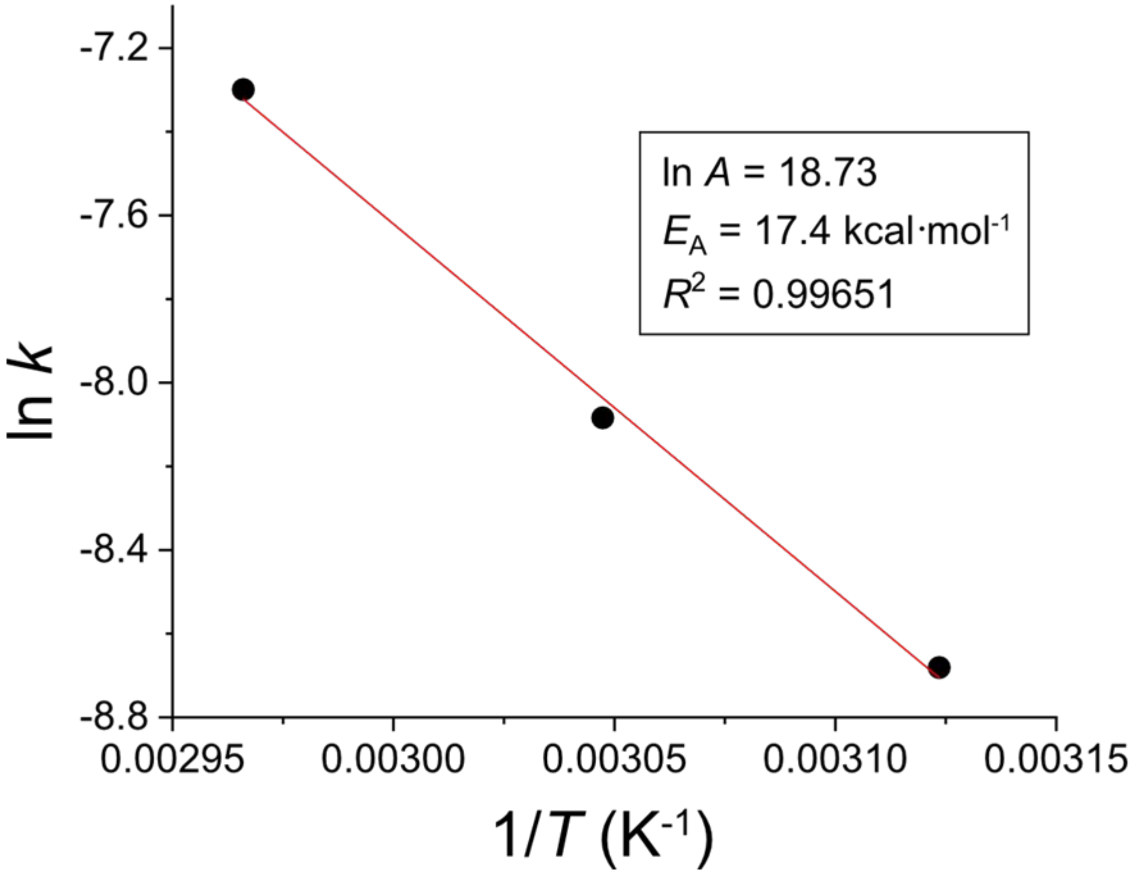

Figure S157. Arrhenius plot of the *Z*-**P**$_a$H$^+$ to *E*-**P**$_a$H$^+$ thermal isomerization process. E_A= 17.4 kcal·mol^{-1}. k_{298K} = 2.2 × 10^{-5} s^{-1}. Half-life: 525 min (ca. 9 h).

5.5.2. Z-$P_bH^+ \rightarrow E$-P_bH^+

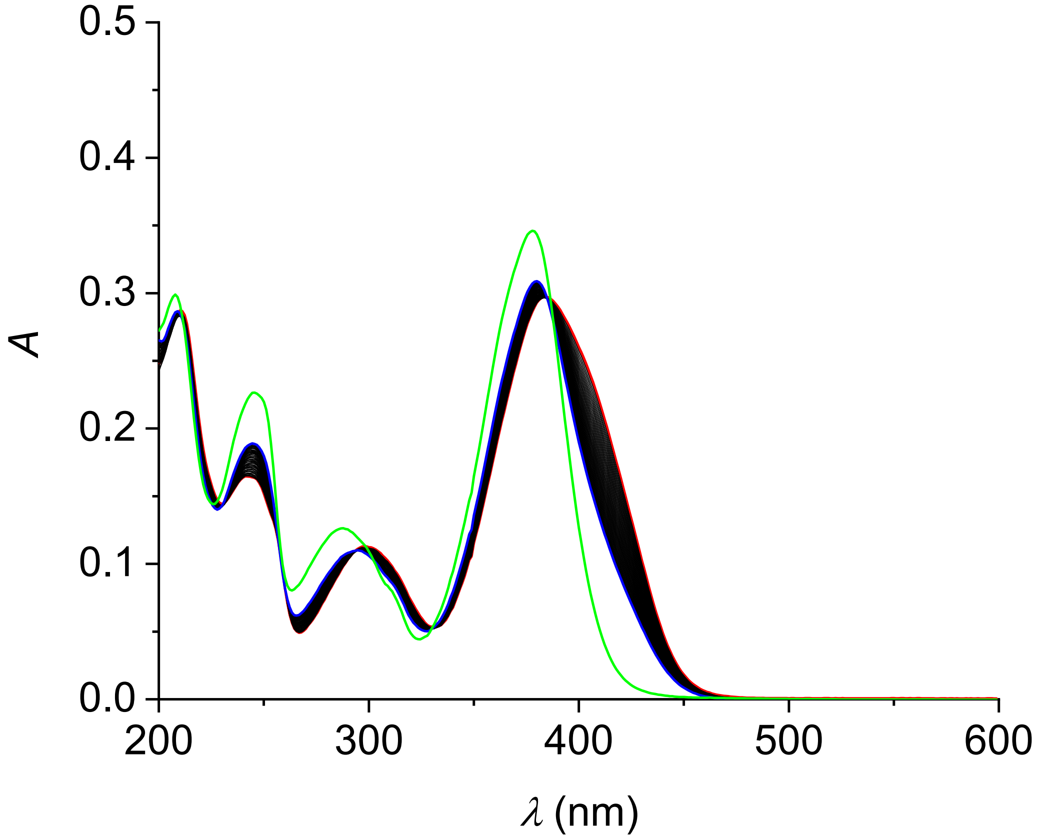

Figure S158. Absorption spectrum variation during 48h at room temperature from the PSS reached for $\mathbf{P}_b$H·Cl (8 µM, pH 5, Red). *E*-isomer absorption spectrum in green. Even though the kinetics have not been fully completed, it can be observed that it occurs much more slowly than photochemical processes.

5.5.3. *Z*-P_cH^+→*E*-P_cH^+

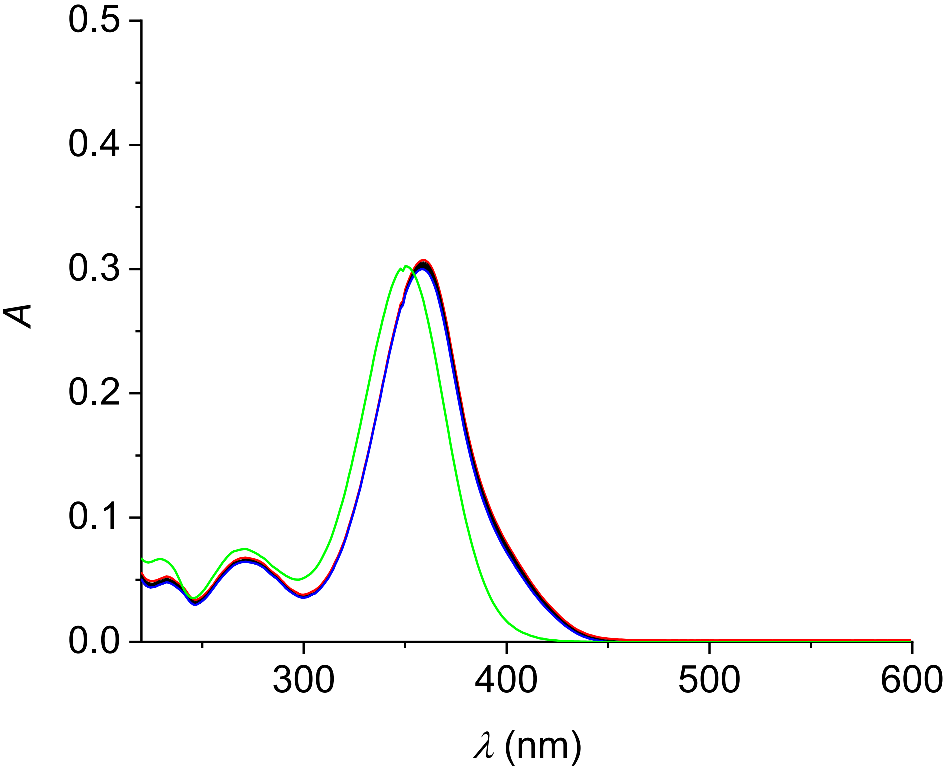

Figure S159. Absorption spectrum variation during 48h at room temperature from the PSS reached for **P**$_c$H·Cl (8 μM, pH 6, Red) for 5 days. *E*-isomer absorption spectrum in green. Even though the kinetics has not been fully completed, it can be observed that it occurs much more slowly than photochemical processes.

5.6. *E*-P_{a-c}→*Z*-P_{a-c} photoisomerization kinetics.

5.6.1. *E*-P_a→*Z*-P_a

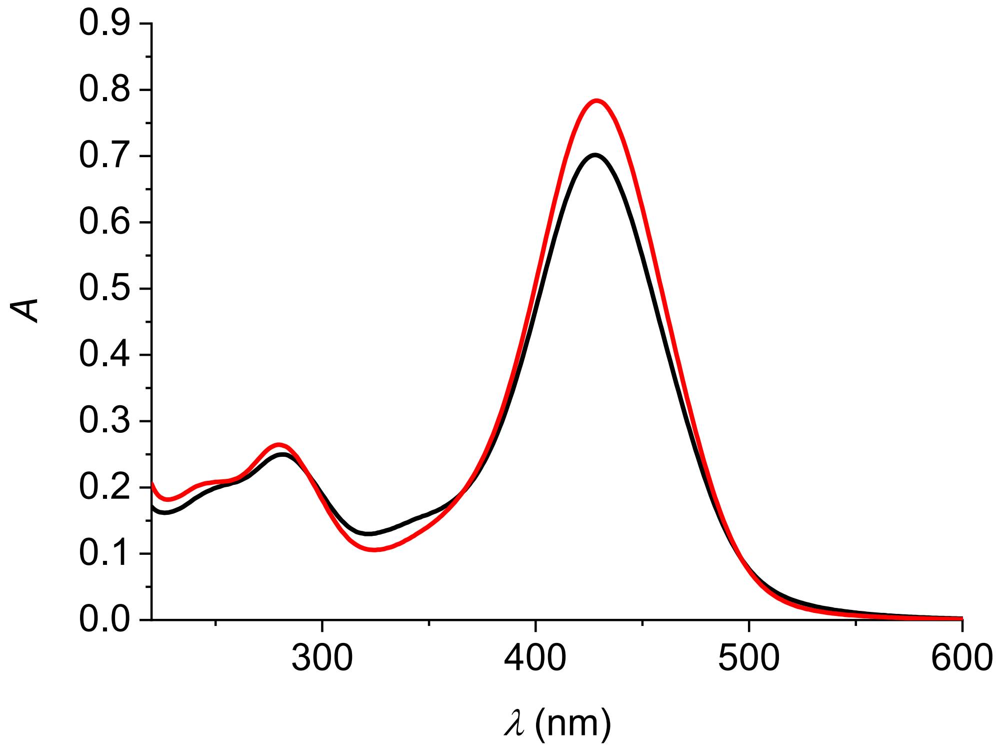

Figure S160. Absorption spectrum changes during irradiation at > 420 nm for $\mathbf{P}_a$H·Cl (20.6 μM, pH 12). *E*-isomer absorption spectrum in red; photostationary-state spectrum after irradiation (120 min) in black.

5.6.2. *E*-P_b→*Z*-P_b

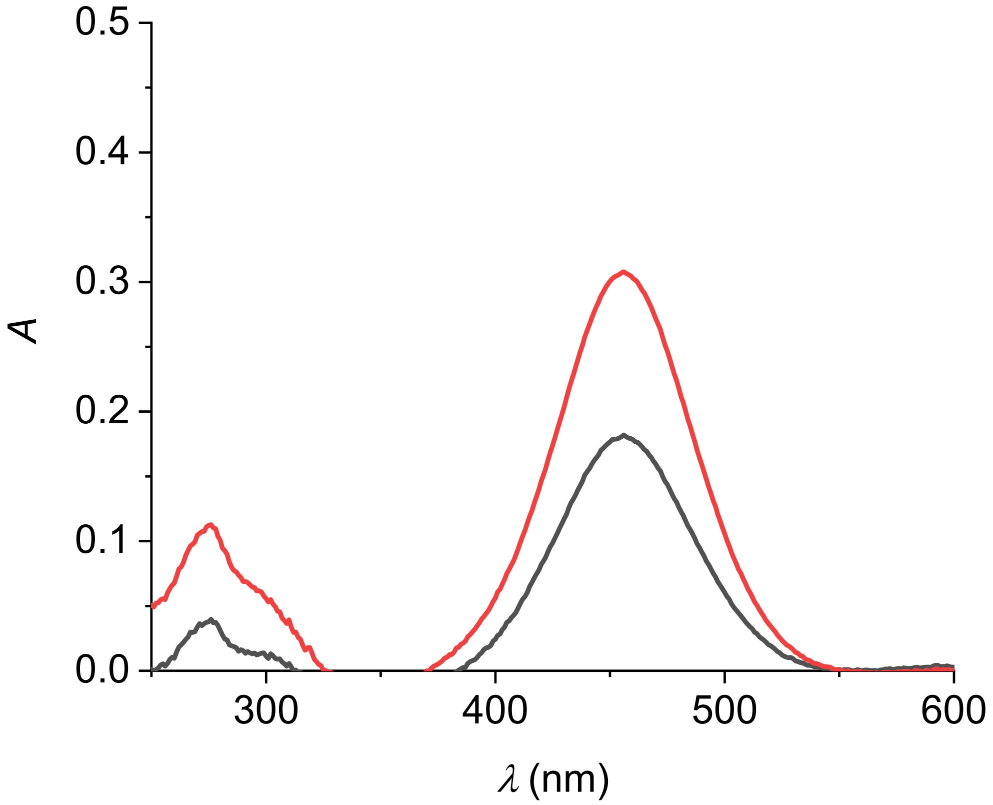

Figure S161. Absorption spectrum changes during irradiation at >420 nm of $\mathbf{P}_b$H·Cl (7.9 μM) at pH 12. *E*-isomer absorption spectrum in red; photostationary-state spectrum after irradiation (120 min) in black (probable precipitation is observed).

5.6.3. *E*-P$_c$→*Z*-P$_c$

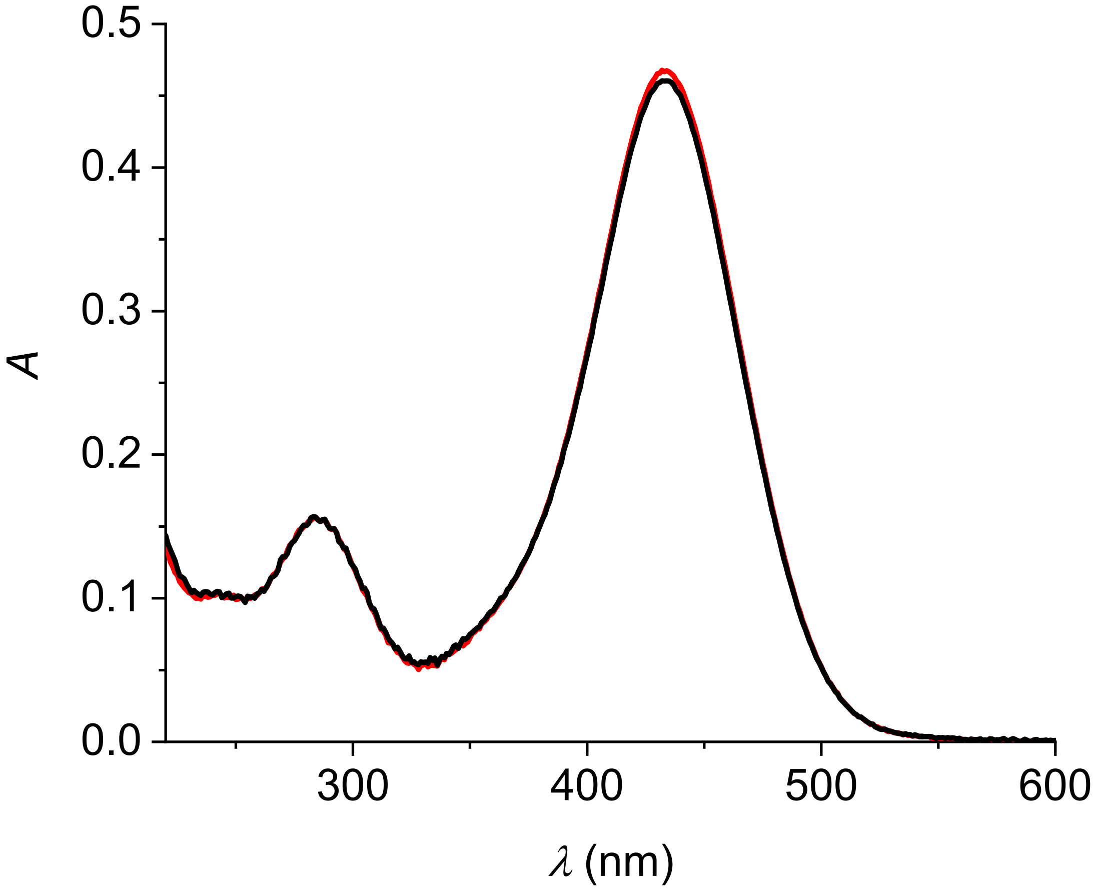

Figure S162. Absorption spectrum changes during irradiation at >420 nm of **P**$_c$H·Cl (9.0 µM, pH 12). *E*-isomer absorption spectrum in red; photostationary-state spectrum after irradiation (120 min) in black.

6. Determination of pK_a values for the PSS by UV-Vis spectroscopy.

The concentration of $\mathbf{P}_{a\text{-}c}$H·Cl was kept constant at appropriate values in the 10-20 μM range, and that of the phosphate buffer at 20 mM. The absorption measurements of the corresponding air-equilibrated phosphate buffered aqueous solutions were carried out at room temperature (298 K), in quartz cuvettes with 1 cm optical pathlength. For the kinetic trapping of the deprotonated *Z*-isomers of $\mathbf{P}_{a\text{-}c}$H·Cl, a solution of the corresponding salt in phosphate buffered solution at a conveniently acidic pH/pD was irradiated at 254 nm until reaching the PSS. Then, the pH of the solution was in each case adjusted to the required final value, by addition of 40 mM stock solutions of phosphate buffer of the required composition. The reported measurements were done as fast as possible to minimize thermal back-isomerization.

6.1. $P_aH{\cdot}Cl$.

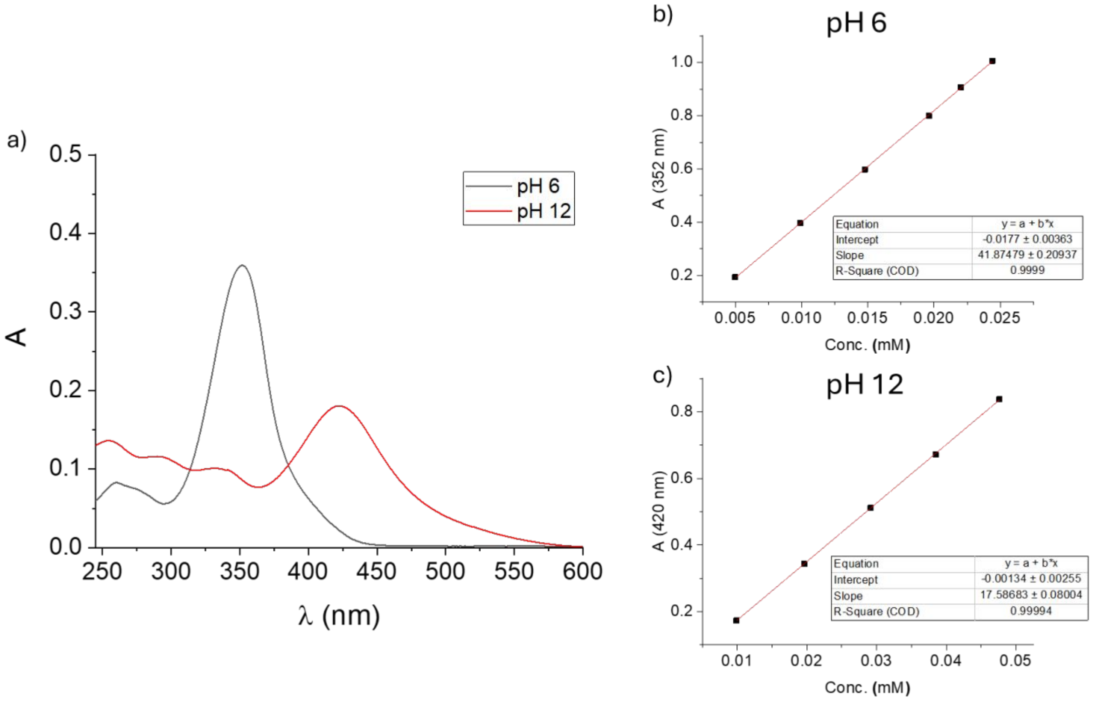

Figure S163. a) UV-Vis spectra for the PSS at pH 6 (black) and 11 (red). Samples prepared following the procedure explained above from an 8.5 µM initial concentration of **P**$_a$H·Cl. b) Linear relationship at pH 6 between absorbance and concentration of **P**$_a$H·Cl (ε = 42700 ± 1185 L·mol^{-1}·cm^{-1}). c) Linear relationship at pH 12 between absorbance at 420 nm and concentration of **P**$_a$H·Cl (ε = 17549 ± 647 L·mol^{-1}·cm^{-1}).

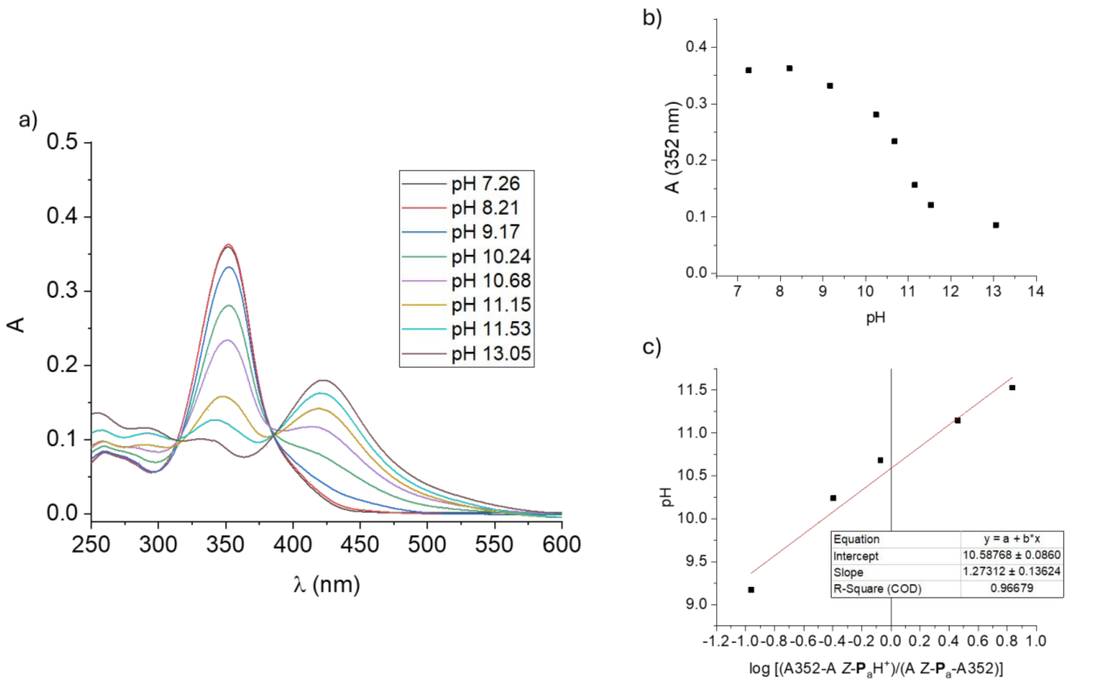

Figure S164. a) UV-Vis spectra for the titration of *Z*-enriched solutions prepared following the procedure explained above, starting from an 8.5 µM initial concentration of **P**$_a$H·Cl. b) Absorption at λ = 352 nm for the species assigned as *Z*-**P**$_a$H$^+$ plotted against pH. c) Linear fitting of pH plotted against log [(A352-A *Z*-**P**$_a$H$^+$)/(A *Z*-**P**$_a$-A352)]. pK_a = 10.4 ± 0.2 as mean value of triplicate experiments.

6.2. $P_bH·Cl$.

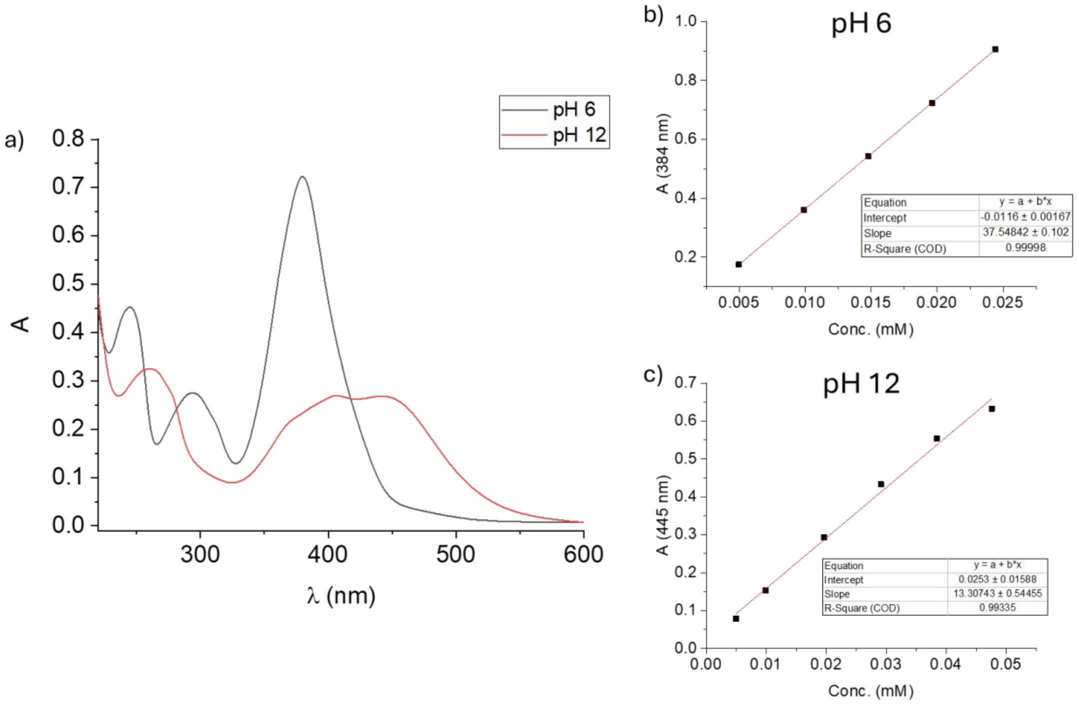

Figure S165. a) UV-Vis spectra for the PSS at pH 6 (black) and 12 (red). Samples prepared following the procedure explained above from a 20.0 µM initial concentration of $\mathbf{P_b}$H·Cl. b) Linear relationship at pH 6 between absorbance at 384 nm and concentration of $\mathbf{P_b}$H·Cl (ε = 36002 ± 1384 L·mol^{-1}·cm^{-1}). c) Linear relationship at pH 12 between absorbance at 445 nm and concentration of $\mathbf{P_b}$H·Cl (ε = 13194 ± 998 L·mol^{-1}·cm^{-1}).

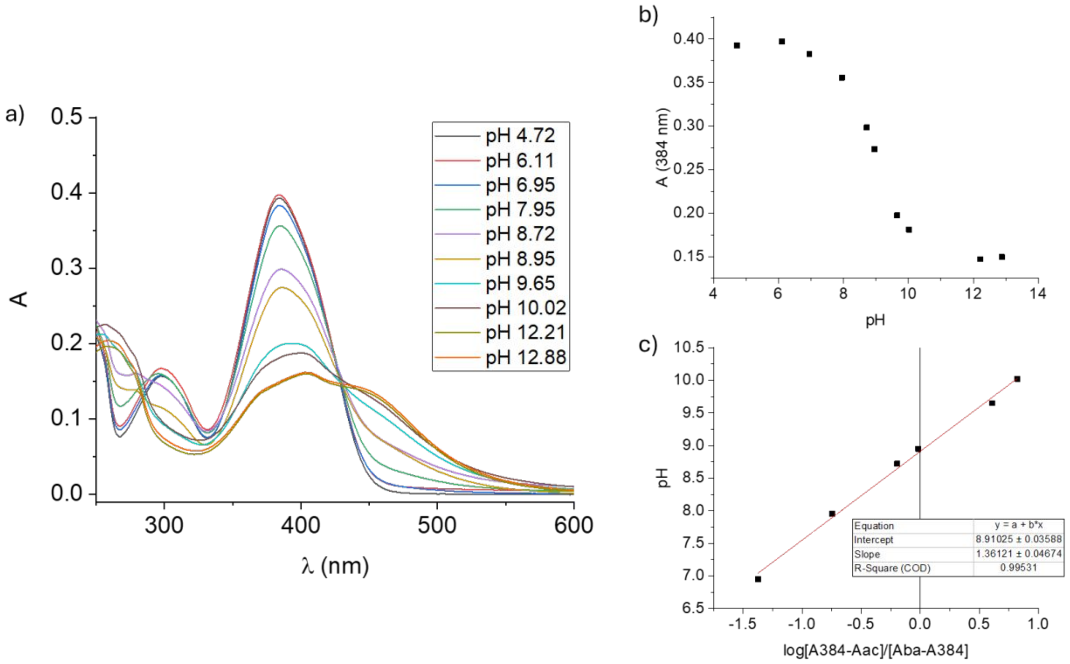

Figure S166. a) UV-Vis spectra for the titration of *Z*- enriched solutions prepared following the procedure explained above, starting from an 11.0 µM initial concentration of $\mathbf{P_b}$H·Cl. b) Absorption for the species assigned as *Z*-$\mathbf{P_b}$H$^+$ at λ = 384 nm plotted against pH. c) Linear fitting of pH plotted against log [(A384-A *Z*-$\mathbf{P_b}$H$^+$)/(A *Z*-$\mathbf{P_b}$-A384)]. pK_a = 8.8±0.2 as mean value of triplicate experiments.

6.3. $P_cH{\cdot}Cl$.

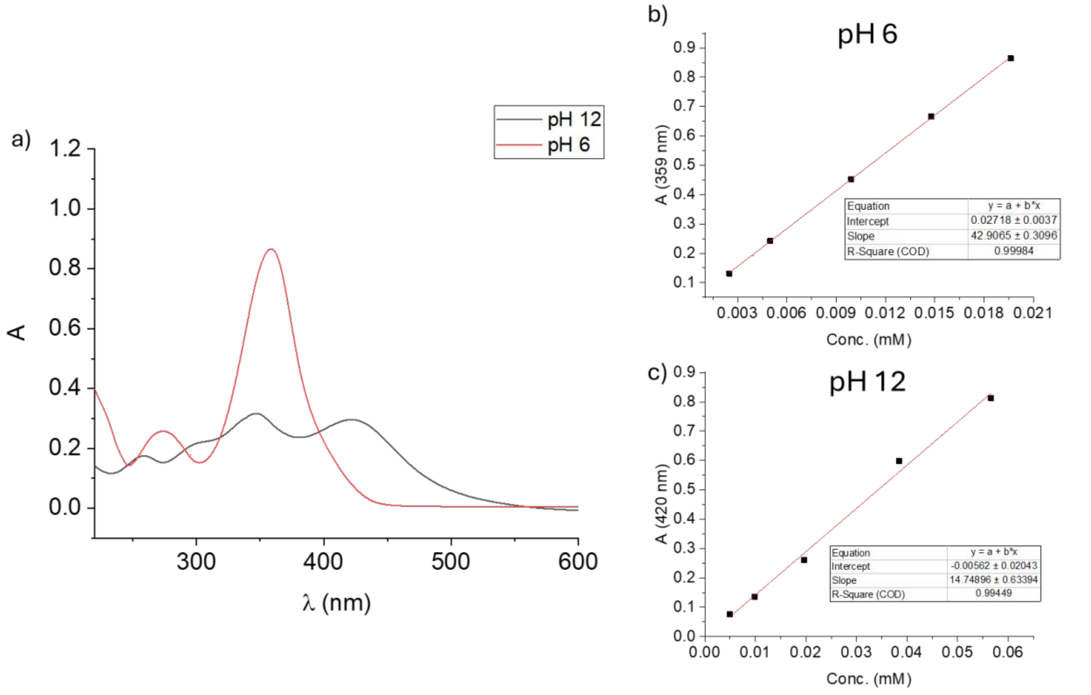

Figure S167. UV-Vis spectra for the PSS at pH 6 (black) and 12 (red). Samples prepared following the procedure explained above from a 20.0 μM initial concentration of $\mathbf{P_c}$H·Cl b) Linear relationship at pH 6 between absorbance at 359 nm and concentration of $\mathbf{P_c}$H·Cl (ε = 43131 ± 1028 L·mol^{-1}·cm^{-1}). c) Linear relationship at pH 12 between absorbance at 420 nm and concentration of $\mathbf{P_c}$H·Cl (ε = 15088 ± 297 L·mol^{-1}·cm^{-1}).

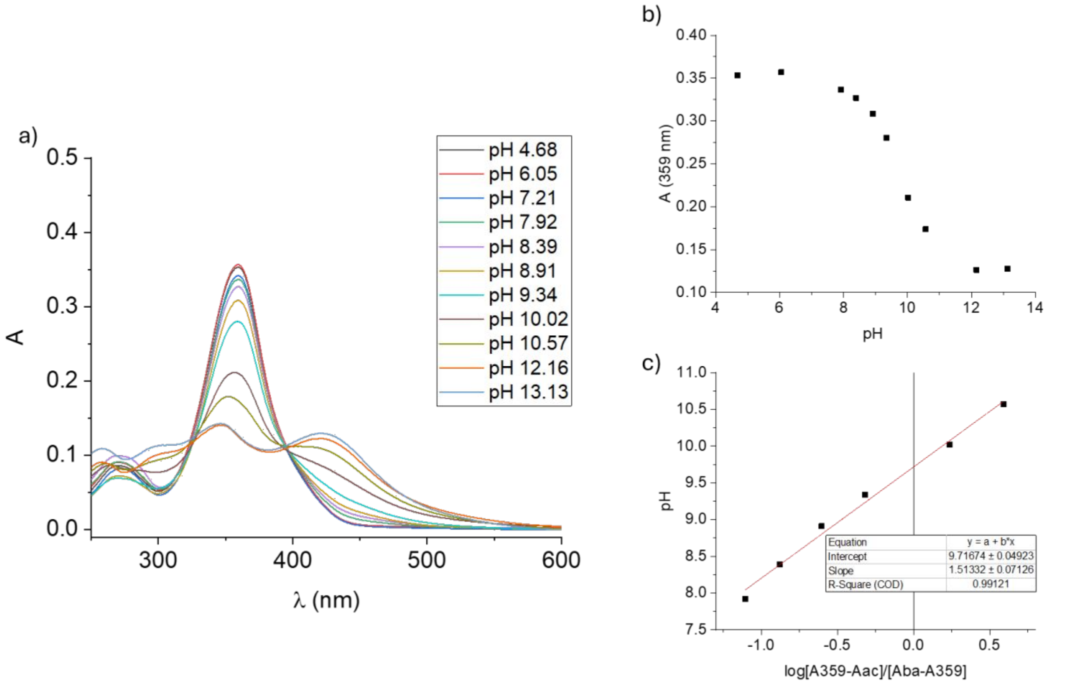

Figure S168. UV-Vis spectra for the titration of *Z*- enriched solutions prepared following the procedure explained above, starting from an 11.0 μM initial concentration of $\mathbf{P_c}$H·Cl. b) Absorption for the species assigned as *Z*-$\mathbf{P_c}$H$^+$ at λ = 359 nm plotted against pH. c) Linear fitting of pH plotted against log [(A359-A *Z*-$\mathbf{P_c}$H$^+$)/(A *Z*-$\mathbf{P_c}$-A359)]. pK_a = 9.9±0.1 as mean value of triplicate experiments.

7. Thermal Z-$\mathbf{P}_{a\text{-}c}$→E-$\mathbf{P}_{a\text{-}c}$ isomerizations from the PSS

To prepare a solution enriched in the Z-$\mathbf{P}_{a\text{-}c}$ isomer, a solution of $\mathbf{P}_{a\text{-}c}$H·Cl was first adjusted to pH 6 using 20 mM phosphate buffer (pH 5 for $\mathbf{P}_b$H·Cl). The solution was then irradiated at a wavelength of 254 nm until the photostationary state was reached, thereby maximizing the proportion of Z-$\mathbf{P}_{a\text{-}c}H^+$. Subsequently, the solution was basified to pH 13 using a NaOH 0.5 M solution and a calibrated pH meter, yielding the desired Z-$\mathbf{P}_{a\text{-}c}$ solution.

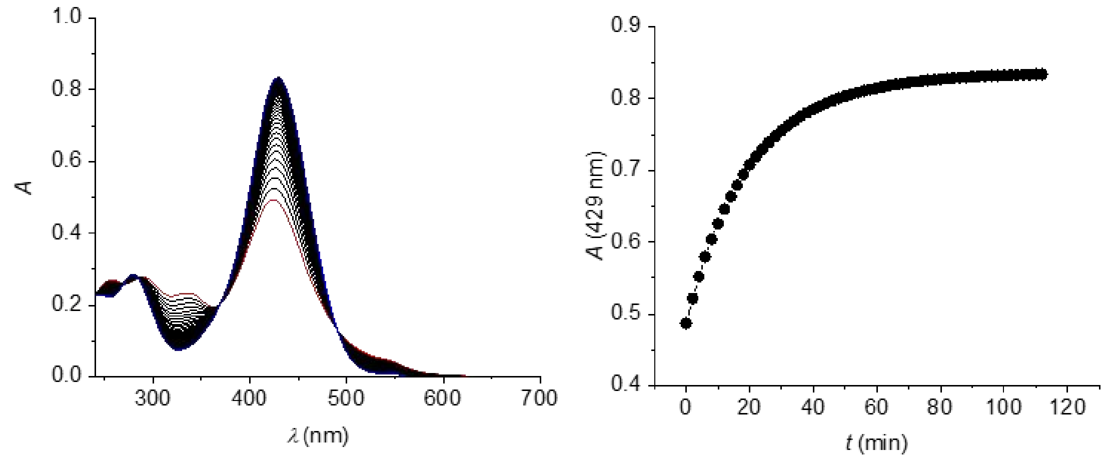

Figure S169. Left: variations in the absorption spectrum of deprotonated $\mathbf{P}_a$H·Cl (26 µM, pH=13) at room temperature; the absorption spectrum of the Z-form is shown in red; the stationary state is in blue. Right: Kinetics (monitored at 429 nm) of the isomerization of Z-$\mathbf{P}_a$ to E-$\mathbf{P}_a$ (26 µM) at room temperature. $k = 7.2 \times 10^{-4}$ s^{-1}.

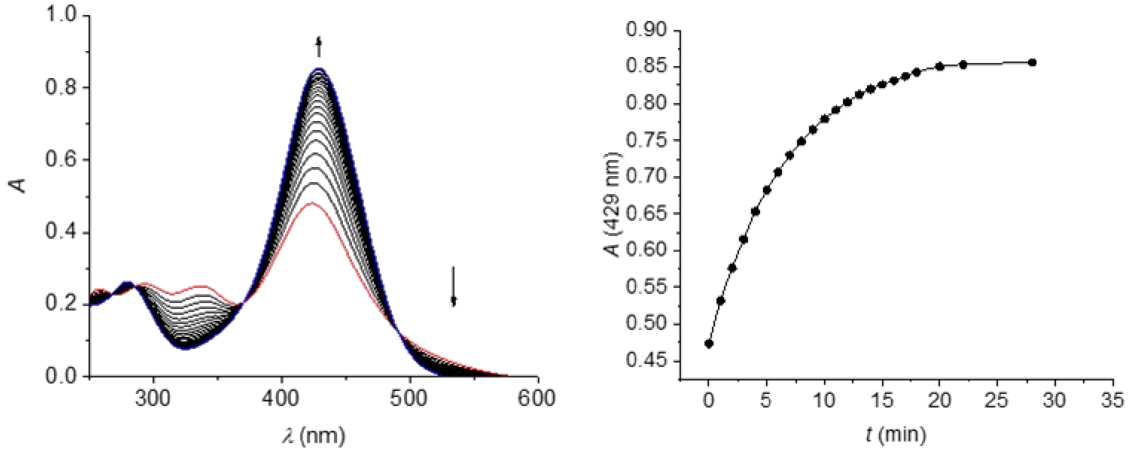

Figure S170. Left: variations in the absorption spectrum of deprotonated $\mathbf{P}_a$H·Cl (26 µM, pH=13) upon irradiation at >495 nm; the absorption spectrum of the Z-form is shown in red; the photostationary state is in blue. Right: Kinetics (monitored at 429 nm) of the photoisomerization of Z-$\mathbf{P}_a$ to E-$\mathbf{P}_a$ (26 µM) upon irradiation at >495 nm. $k = 2.5 \times 10^{-3}$ s^{-1}.

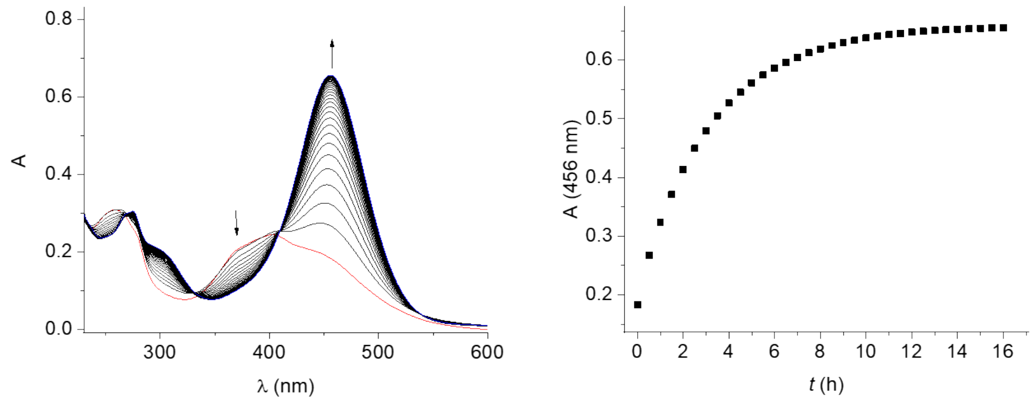

Figure S171. Left: variations in the absorption spectrum of deprotonated $\mathbf{P}_b$H·Cl (15 μM, pH=13) at room temperature; the absorption spectrum of the *Z*-form is shown in red; the stationary state is in blue. Right: Kinetics (monitored at 456 nm) of the isomerization of *Z*-$\mathbf{P}_b$ to *E*-$\mathbf{P}_b$ (15 μM) at room temperature. $k = 8.9 \times 10^{-5}$ s^{-1}.

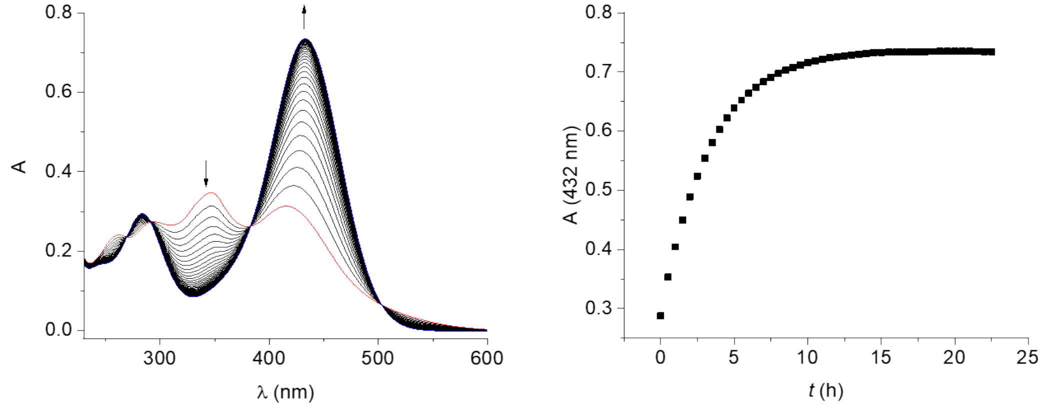

Figure S172. Left: variations in the absorption spectrum of deprotonated $\mathbf{P}_c$H·Cl (16 μM, pH=13) at room temperature; the absorption spectrum of the *Z*-form is shown in red; the stationary state is in blue. Right: Kinetics (monitored at 432 nm) of the isomerization of *Z*-$\mathbf{P}_c$ to *E*-$\mathbf{P}_c$ (16 μM) at room temperature. $k = 8.4 \times 10^{-5}$ s^{-1}.

8. X-RAY crystallographic data.

8.1. Obtention of X-Ray crystals of $\mathbf{P_a{\cdot}H{\cdot}PF_6}$, $\mathbf{P_b{\cdot}H{\cdot}I}$, $\mathbf{P_c{\cdot}H{\cdot}PF_6}$.

Single crystals suitable for X-ray diffraction were obtained via slow vapor diffusion crystallization in a closed system. Compounds $\mathbf{P}_{a,c}H{\cdot}PF_6$ or $\mathbf{P}_b{\cdot}H{\cdot}I$ (50 mg) were dissolved in the minimum volume of acetonitrile (2 mL) and placed in a small vial. This vial was then inserted into a larger sealed vial containing diethyl ether as the antisolvent. The system was stored at low temperature (refrigerated), allowing gradual diffusion of Et_2O into the acetonitrile solution. After 48 hours, well-formed single crystals appropriate for X-ray analysis were observed.

8.1.1. X-Ray crystallographic data of $\mathbf{P_a{\cdot}H{\cdot}PF_6}$ (CCDC: 2486929).

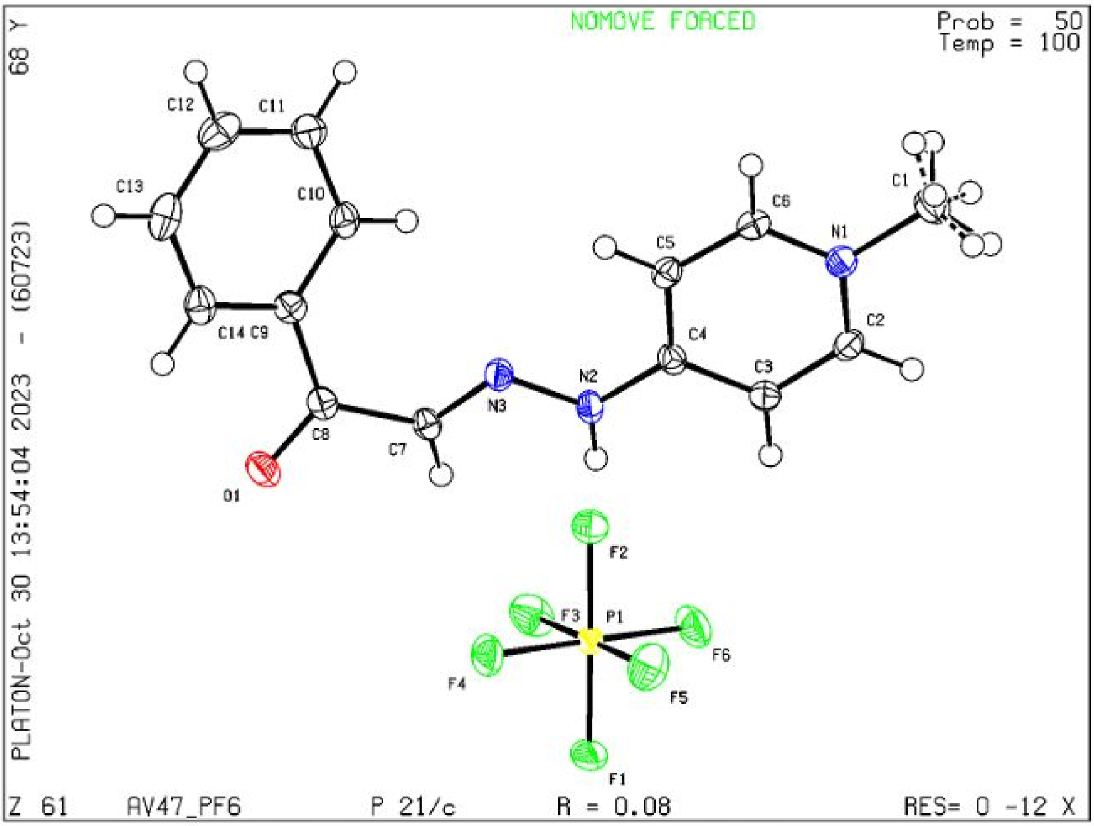

Figure S173. ORTEP plot of the X-ray structure of $\mathbf{P}_a H{\cdot}PF_6$. The displacement ellipsoids are shown at the 50% probability.

Data collection: APPEX3v2022.1-1 (BRUKER AXS, 2021); cell refinement: SAINT V8.40B (Bruker AXS LLC, 2019); data reduction: *SAINT* V8.40B (Bruker AXS LLC, 2019); program(s) used to solve structure: *SHELXT2018/2,*[6] program(s) used to refine

[6] Shelidrick, G.M. Crystal structure refinement with SHELXL. *Acta Cryst.* **2015**, *C71*, 3-8

structure: SHELXL2019/*1*,[7] molecular graphics: *ORTEP* for Windows,[8] software used to prepare material for publication: *WinGX* publication routines.[7]

Special details: All e.s.d.'s (except the e.s.d. in the dihedral angle between two l.s. planes) are estimated using the full covariance matrix. The cell e.s.d.'s are considered individually in the estimation of e.s.d.'s in distances, angles and torsion angles; correlations between e.s.d.'s in cell parameters are only used when they are defined by crystal symmetry. An approximate (isotropic) treatment of cell e.s.d.'s is used for estimating e.s.d.'s involving l.s. planes.

A summary of the crystallographic data and the structure refinement parameters is reported in **Table S1**.

TABLE S1. Crystal data and structure refinement of $\mathbf{P}_a$H·PF_6.

**Crystal data**	
Empirical formula	$C_{14}H_{14}N_3O\cdot F_6P$
Mr	385.25
Crystal system	Monoclinic, $P2_1/c$
Hall symbol	-P 2ybc
a (Å)	6.8845 (6)
b (Å)	10.6842 (9)
c (Å)	21.4382 (19)
β (°)	90.608 (3)
V (Å^3)	1576.8 (2)
Z	4
F(000)	784
Dx (Mg m^{-3})	1.623
Radiation	Mo Kα (λ = 0.71073 Å)
Temperature (K)	100
Crystal description	Plate, colourless, 0.28 × 0.04 × 0.03 mm
**Data collection**	
Diffractometer	Bruker D8 VENTURE PHOTON III-14
Radiation source	INCOATEC microfocus sealed tube, Incoatec IμS 3.0
Monochromator	Incoatec multilayer mirror

[7] Shelidrick, G.M. Aspherical scattering factors for SHELXL – model, implementation and application. *Acta Cryst*. **2019**, *A75*, 50–62
[8] Farrugia, L.J. WinGX and ORTEP for Windows: an update. *J. Appl. Cryst.* **2012**, *45*, 849–854

Detector resolution	7.3910 pixels mm^{-1}
Scan type	ω and phi
Absorption correction	multi-scan (BRUKER SADABS2016/2)
T_{min}	0.896
T_{max}	0.926
Reflections measured	69739
Independent reflections	3891
Reflections with $I > 2\sigma(I)$	3358
R_{int}	0.061
θ range (°)	2.1–28.3
h	-9→9
k	-14→14
l	-28→28
**Refinement**	
Refinement on F^2	
Least-squares matrix	Full
$R[F^2 > 2\sigma(F^2)]$	0.085
$wR(F^2)$	0.253
S	1.09
Reflections	3891
Parameters	228
Restraints	0
Constraints	0
Primary atom site location	dual
Secondary atom site location	dual
Hydrogen site location	inferred from neighbouring sites
H-atom parameters	constrained
Weighting scheme	$w = 1/[\sigma^2(Fo^2) + (0.0812P)^2 + 13.7565P]$, $P = (Fo^2 + 2Fc^2)/3$
$(\Delta/\sigma)_{max}$	< 0.001
$\Delta\rho_{max}$ (e Å^{-3})	1.09
$\Delta\rho_{min}$ (e Å^{-3})	−0.97

9.1.2 X-Ray crystallographic data of P_b·H·I (CCDC: 2487028).

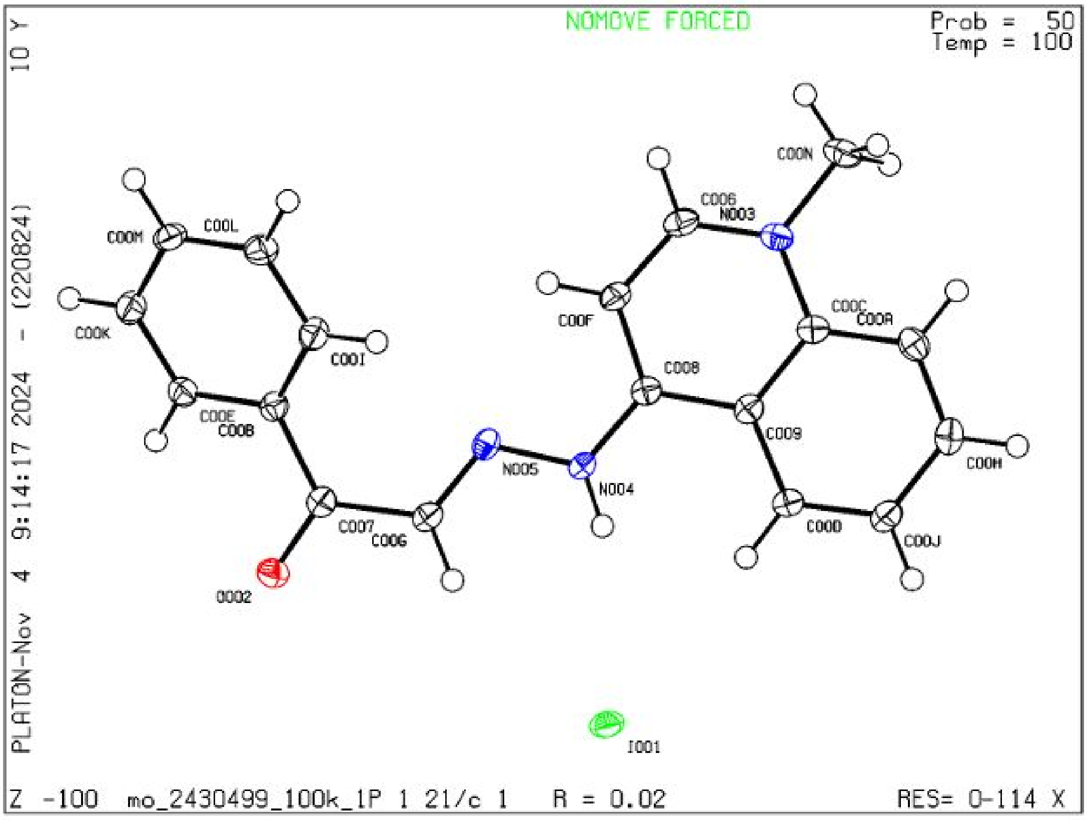

Figure S174. ORTEP plot of the X-ray structure of **P_b**H·I. The displacement ellipsoids are shown at the 50% probability.

A single crystal of **P_b**H·I was analysed by X-ray diffraction and a summary of the crystallographic data and the structure refinement parameters is reported in **Table S2**. Crystallographic data were collected at 100 K using a Bruker D8 Venture diffractometer with a Photon III C14 detector and Mo-Kα radiation (λ = 0.71073 Å) generated by an Incoatec microfocus diamond II source. The software APEX6 Version 2024.9-0 (Bruker AXS Inc., 2024) was used for collecting frames of data, indexing reflections, and determination of lattice parameters, SAINT Version 8.41 (Bruker AXS Inc., 2016) for integration of intensity of reflections, and SADABS Version 2016/2,[9] for scaling and empirical absorption correction. The structure was solved by dual-space algorithm using the program SHELXT.[10] All non-hydrogen atoms were refined with anisotropic displacement parameters by full-matrix least-squares

[9] Krause, L.; Herbst-Irmer, R.; Sheldrick, G. M.; Stalke, D. Comparison of Silver and Molybdenum Microfocus X-ray Sources for Single-Crystal Structure Determination. *J. Appl. Crystallogr.* **2015**, *48*, 3–10.
[10] Sheldrick, G. M. SHELXT Integrated Space-Group and Crystal-Structure Determination. *Acta Crystallogr., Sect. A: Found. Adv.* **2015**, *71*, 3–8.

calculations on F^2 using the program SHELXL,[5] with OLEX2.[11] Hydrogen atoms were inserted at calculated positions and constrained with isotropic displacement.

TABLE S2. Crystal data and structure refinement of $\mathbf{P_b}$H·I.

Empirical formula	$C_{18} H_{16} I N_3 O$	
Formula weight	417.24	
Temperature	100.00 K	
Wavelength	0.71073 Å	
Crystal system	Monoclinic	
Space group	P 1 21/c 1	
Unit cell dimensions	a = 8.9533(8) Å	α= 90°
	b = 7.9762(8) Å	β= 92.447(3)°
	c = 23.588(2) Å	γ = 90°
Volume	1683.0(3) Å^3	
Z	4	
Density (calculated)	1.647 Mg/m^3	
Absorption coefficient	1.910 mm^{-1}	
F(000)	824	
Crystal size	0.166 x 0.078 x 0.042 mm^3	
Theta range for data collection	2.277 to 28.308°	
Index ranges	-11<=h<=11, -10<=k<=10, -31<=l<=31	
Reflections collected	58785	
Independent reflections	4109 [R(int) = 0.0543]	
Completeness to theta = 25.242°	98.0 %	
Absorption correction	Semi-empirical from equivalents	
Max. and min. transmission	0.7457 and 0.6612	

[11] Dolomanov, O. V.; Bourhis, L. J.; Gildea, R. J.; Howard, J. A. K.; Puschmann, H. OLEX2: A Complete Structure Solution, Refinement and Analysis Program. *J. Appl. Crystallogr.* **2009**, *42*, 339–341.

Refinement method	Full-matrix least-squares on F^2
Data / restraints / parameters	4109 / 0 / 209
Goodness-of-fit on F2	1.054
Final R indices [I>2sigma(I)]	R1 = 0.0237, wR2 = 0.0500
R indices (all data)	R1 = 0.0258, wR2 = 0.0510
Extinction coefficient	n/a
Largest diff. peak and hole	0.377 and -0.599 e.$Å^{-3}$

9.1.3. X-Ray crystallographic data of **P**$_c$**·H·PF**$_6$ **(CCDC: 2486961).**

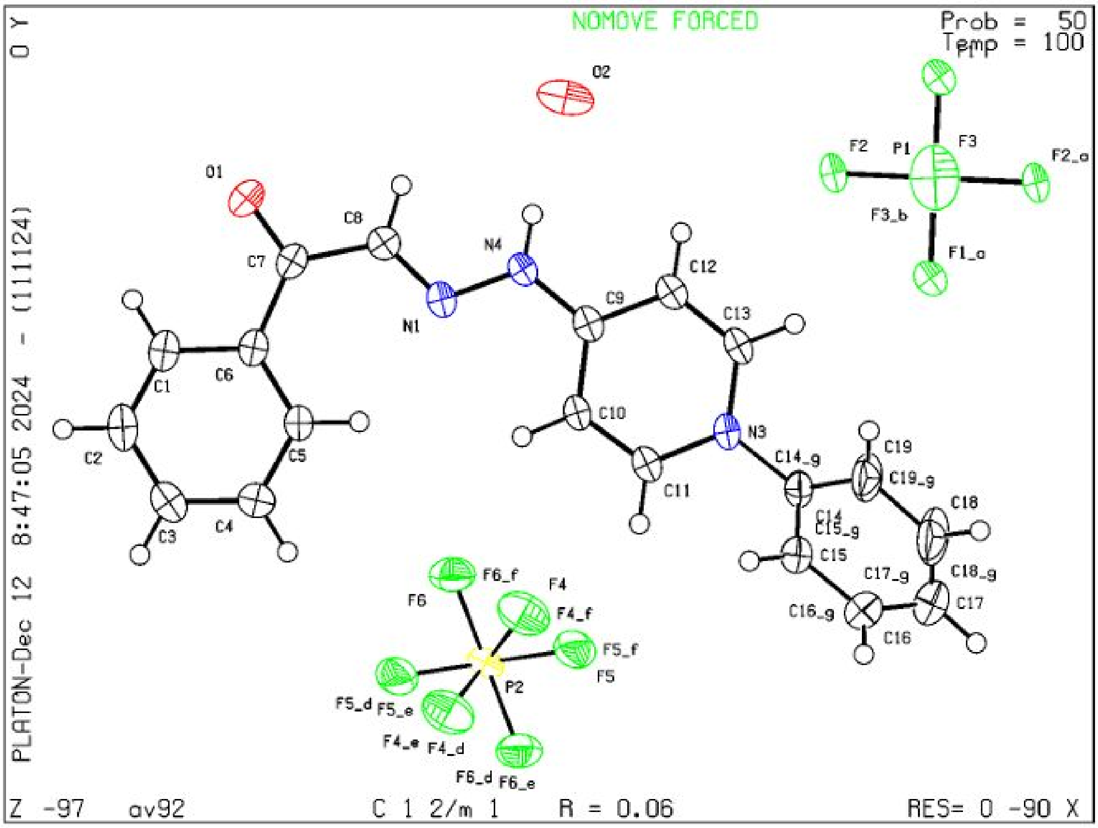

Figure S175. ORTEP plot of the X-ray structure of **P**$_c$H·PF$_6$. The displacement ellipsoids are shown at the 50% probability.

A single crystal of **P**$_c$H·PF$_6$ was analysed by X-ray diffraction and a summary of the crystallographic data and the structure refinement parameters is reported in **Table S3**. Crystallographic data were collected at 100 K using a Bruker D8 Venture diffractometer with a Photon III C14 detector and Mo-Kα radiation (λ = 0.71073 Å) generated by an Incoatec microfocus diamond II source. The software APEX6 Version 2024.9-0 (Bruker AXS Inc., 2024) was used for collecting frames of data, indexing reflections, and determination of lattice parameters, SAINT Version 8.41 (Bruker AXS Inc., 2016) for integration of intensity of reflections, and SADABS Version 2016/2,[8] for scaling and empirical absorption correction. The structure was solved by dual-space algorithm using the program SHELXT.[9] All non-hydrogen atoms were refined with anisotropic displacement parameters by full-matrix least-squares calculations on F^2 using the program SHELXL,[5] with OLEX2.[10] Hydrogen atoms were inserted at calculated positions and constrained with isotropic displacement. Attempts to locate the hydrogen atoms of the crystallized water molecules from the difference map were unsuccessful. The P atoms lie on inversion centers and mirror planes. F atoms and one aromatic ring (C14-C15-C16-C17-C18-C19) are generated by the symmetry operations.

TABLE S3. Crystal data and structure refinement of $\mathbf{P_c}H\cdot PF_6$.

Empirical formula	$C_{19} H_{16} F_6 N_3 O_2 P$	
Formula weight	463.32	
Temperature	100.00 K	
Wavelength	0.71073 Å	
Crystal system	Monoclinic	
Space group	C 1 2/m 1	
Unit cell dimensions	a = 19.354(2) Å	α= 90°
	b = 6.6707(7) Å	β= 110.446(4)°
	c = 17.034(2) Å	γ = 90°
Volume	2060.6(4) $Å^3$	
Z	4	
Density (calculated)	1.493 Mg/m^3	
Absorption coefficient	0.208 mm^{-1}	
F(000)	944	
Crystal size	0.125 x 0.074 x 0.074 mm^3	
Theta range for data collection	2.246 to 20.831°	
Index ranges	-19<=h<=19, -6<=k<=6, -17<=l<=17	
Reflections collected	20782	
Independent reflections	1203 [R(int) = 0.0713]	
Completeness to theta = 20.831°	99.8 %	
Absorption correction	Semi-empirical from equivalents	
Max. and min. transmission	0.6631 and 0.6425	
Refinement method	Full-matrix least-squares on F^2	
Data / restraints / parameters	1203 / 0 / 215	
Goodness-of-fit on F2	1.109	
Final R indices [I>2sigma(I)]	R1 = 0.0533, wR2 = 0.1423	

R indices (all data)	R1 = 0.0575, wR2 = 0.1470
Extinction coefficient	n/a
Largest diff. peak and hole	0.700 and -0.233 e.$Å^{-3}$
