# Supplementary material for: Photovermellogens: Minimalistic Pyridinium-Based Acylhydrazones with Photoswitchable Basicity for Operation in Aqueous Media
Source: J Org Chem. 2026 May 4;91(19):6559–65. doi: 10.1021/acs.joc.5c02962 (PMC13185115; doi:10.1021/acs.joc.5c02962)
Supplement: Supplementary file 2 [file jo5c02962_si_002.pdf]

Supporting Information:

Photovermellogens: Minimalistic Pyridinium-Based Acylhydrazone with Photoswitchable Basicity for Operation in Aqueous Media

Alejandro Vila[a], Francisco G. Blandón-Cumbreras[b], Mike Pauls[c], Mauro Díaz-Abellás[a], Arturo Blanco-Gómez[a], Carlos Peinador[a], Patricia Remón[b], Christoph Bannwarth*[c], Uwe Pischel*[b], and Marcos D. García*[a]

[a]CICA-Centro Interdisciplinar de Química e Bioloxía and Departamento de Química, Facultade de Ciencias, Universidade da Coruña, 15071 A Coruña, Spain.
[b]Center for Research in Sustainable Chemistry (CIQSO) and Department of Chemistry, University of Huelva, 21071 Huelva, Spain.
[c]Institute of Physical Chemistry, RWTH Aachen University, 52074 Aachen, Germany.

*Correspondence to: bannwarth@pc.rwth-aachen.de (C.B.), uwe.pischel@diq.uhu.es (U.P.), marcos.garcia1@udc.es (M.D.G.)

Contents

S1 Quantumchemical Calculations **S3**
S1.1 Generation of Structures S3
S1.2 Computation of Free Energies S4
S1.3 Simulation of UV-Vis Absorption Spectra S6
S1.4 Modelling of Thermal Isomerization S10
S1.5 Modelling of Photoisomerization S11

S1 Quantumchemical Calculations

S1.1 Generation of Structures

The computational investigation of this work focuses on the molecules $\mathbf{P}_{\mathrm{a}}\mathrm{H}^{+}$ and its conjugate base form $\mathbf{P}_{\mathrm{a}}$, whose E- and Z-isomers are shown in Figure S1.

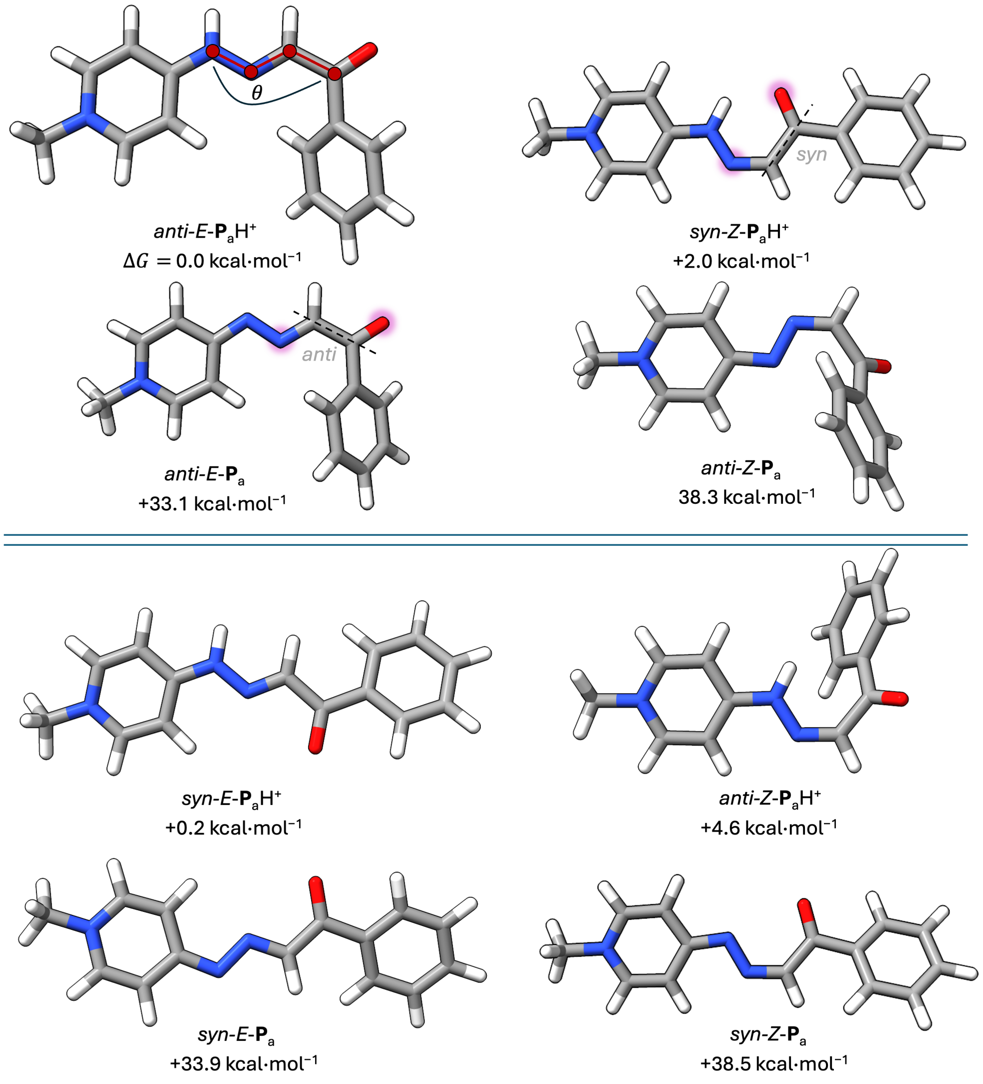

Figure S1: Top: Ground state geometries of the thermodynamically favored conformations (*anti*/*syn*) *anti*-E-$\mathbf{P}_{\mathrm{a}}\mathrm{H}^{+}$, *syn*-$Z$-$\mathbf{P}_{\mathrm{a}}\mathrm{H}^{+}$ and the conjugate bases *anti*-E-$\mathbf{P}_{\mathrm{a}}$ and *anti*-Z-$\mathbf{P}_{\mathrm{a}}$ calculated at the PBEh-3c+CPCM(H_2O) level of theory. Free energy differences refer to the same level of theory (see Tab. 1 and text below for details). The dihedral angle θ differentiating E- and Z-, and *syn*- and *anti*-isomers are defined as well. Bottom: Thermodynamically disfavored *syn*- and *anti* conformations.

We used the semiempirical electronic structure theory method GFN2-xTB[1] as implemented in the xtb program (version 6.6.1)[2] to first optimize the corresponding molecules.

Based on these geometries, we performed metadynamics-based conformational sampling with the conformer-rotamer ensemble sampling tool (CREST)[3] (version 2.11.3)[4] at the GFN2-xTB level including the analytical linearized Poisson-Boltzmann (ALPB)[5] implicit solvation model for water. From this conformational sampling, we obtained both isomers (E/Z) with *syn* or *anti* configuration of the benzaldehyde moiety in an unbiased manner. All conformers within the conformer ensemble (CE) were then reoptimized with restricted Kohn-Sham density functional theory (RKS-DFT) using the composite method PBEh-3c[6] and the conductor-like polarizable continuum (CPCM)[7, 8] implicit solvation model as available in the ORCA program (version 6.0.1).[9, 10, 11]

As a member of the 3c-family of methods, PBEh-3c corrects for some known deficiencies of DFT, while still maintaining moderate computational cost. In PBEh-3c, a modified valence double-ζ basis set ("def2-mSVP") based on the Ahlrich-type basis set def2-SV(P)[12] and a hybrid functional based on that of Perdew, Burke and Ernzerhof (PBE)[13] with 42% of non-local Fock exchange are utilized. Dispersion interactions and the basis set superposition error are addressed by means of Grimme's D3 dispersion correction[14] and a geometric counterpoise correction[15], respectively.

Using the CREGEN routine of the CREST program, we filtered out dublicates within each CE and removed energetically higher-lying conformations (3.0 $\mathrm{kcal \cdot mol^{-1}}$ with respect to the energetically lowest conformer, based on the PBEh-3c+CPCM(H_2O) energies). If not stated otherwise, the reported energy differences refer to a single minimum energy conformer, which was determined from the CE electronic energies (PBEh-3c+CPCM(H_2O)).

S1.2 Computation of Free Energies

To derive meaningful Gibbs free energies for species i, we began with a short assessment of different theory levels, following eq. 1 or 2 to compute G_i.

$$G_i = E_{\mathrm{vac},i} + G_{\mathrm{TRV},i} + \delta G_{\mathrm{solv},i} \tag{1}$$

$$G_i = E_{\mathrm{solv},i} + G_{\mathrm{TRV},i} \tag{2}$$

In eq. 1, the electronic energy in gas phase, $E_{\mathrm{vac},i}$ is accounted for, while eq. 2 implicitly contains solvation contributions to the electronic energy in $E_{\mathrm{solv},i}$, i.e. these were electronic energy calculations determined with CPCM(H_2O). The calculations of E_{vac} and E_{solv} were performed with the ORCA program. A thermostatistical correction that accounts for nuclear translational, rotational and vibrational (TRV) degrees of freedom and the zero point vibrational energy, $G_{\mathrm{TRV},i}$, is considered in both cases. When the electronic energy is determined in gas phase, a solvation correction $\delta G_{\mathrm{solv},i}$ was explicitly added (eq. 1) computed as the difference in (free) energy for the system in vacuum (dielectric environment) at the GFN2-xTB level of theory using the ALPB model, $\delta G_{\mathrm{solv},i} = G_{\mathrm{solv},i}^{\mathrm{GFN2\text{-}xTB,ALPB}} - E_{\mathrm{vac},i}^{\mathrm{GFN2\text{-}xTB}}$. In these calculations, a standard state correction for the conversion of 1 mol of an ideal gas at 1 bar to a 1 M solution (keyword 'bar1M' in xtb) was accounted for.

Table 1 lists the relative free energy differences (T = 298 K) between $\mathbf{P}_\mathrm{a}\mathrm{H}^+$ and $\mathbf{P}_\mathrm{a}$ for selected theory levels used to determine the terms associated with G_i.

Table 1: Relative free energy differences ΔG of *syn*/*anti*-E- and Z-isomers of $\mathbf{P}_{\mathrm{a}}\mathrm{H}^+$ and $\mathbf{P}_{\mathrm{a}}$ relative to *anti*-E-$\mathbf{P}_{\mathrm{a}}\mathrm{H}^+$. Throughout, G_{TRV} contributions employing the PBEh-3c+CPCM geometries and harmonic frequencies scaled by 0.95[16] were used. ^{1}: Free energy determined following eq. 1 with δG_{solv} computed using GFN2-xTB+ALPB at the PBEh-3c+CPCM geometries. ^{2}: Free energy determined following eq. 2. ^{a}: E_{vac} computed at the PW6B95-D4/def2-QZVPP level of theory. ^{b}: E_{solv} computed at the PW6B95-D4/def2-QZVPP+CPCM level of theory. ^{c}: E_{vac} computed at the ωB97X-D4/def2-QZVPP level of theory. ^{d}: E_{solv} computed at the ωB97X-D4/def2-QZVPP+CPCM level of theory. ^{e}: E_{solv} computed at the PBEh-3c+CPCM level of theory.

Molecule	ΔG kcal $\cdot$ mol^{-1}				
	^{1,a}	^{2,b}	^{1,c}	^{2,d}	^{2,e}
anti-E-$\mathbf{P}_{\mathrm{a}}\mathrm{H}^+$	+0.0	+0.0	+0.0	+0.0	+0.0
syn-E-$\mathbf{P}_{\mathrm{a}}\mathrm{H}^+$	+2.1	+0.4	2.5	0.6	0.2
anti-Z-$\mathbf{P}_{\mathrm{a}}\mathrm{H}^+$	+1.9	4.6	1.6	4.1	4.6
syn-Z-$\mathbf{P}_{\mathrm{a}}\mathrm{H}^+$	−3.3	+0.8	−2.4	+1.5	+2.0
anti-E-$\mathbf{P}_{\mathrm{a}}$	+24.7	+37.7	+26.6	+39.5	+33.1
syn-E-$\mathbf{P}_{\mathrm{a}}$	+27.1	+38.5	29.3	40.6	33.9
anti-Z-$\mathbf{P}_{\mathrm{a}}$	+32.2	43.9	32.4	43.9	38.3
syn-Z-$\mathbf{P}_{\mathrm{a}}$	+32.6	+43.8	+34.9	+45.9	+38.5

We compare a variety of theory levels aiming to answer which methodological combination is suited to describe the free energy differences for the presented molecules. This small benchmark is necessary, because the difference *anti*-E-$\mathbf{P}_{\mathrm{a}}\mathrm{H}^+ \rightarrow$ *syn*-Z-$\mathbf{P}_{\mathrm{a}}\mathrm{H}^+$ is endergonic (exergonic) when electronic energies (do not) include electrostatic contributions from CPCM.

Comparing the relative free energies that contain E_{vac} (^{1,a}) or E_{solv} (^{2,b}) at the PW6B95-D4[17, 18]/def2-QZVPP[12](+CPCM) level, we obtain a difference of -3.3 kcal $\cdot$ mol^{-1} or $+0.8$ kcal $\cdot$ mol^{-1} for *anti*-E-$\mathbf{P}_{\mathrm{a}}\mathrm{H}^+ \rightarrow$ *syn*-Z-$\mathbf{P}_{\mathrm{a}}\mathrm{H}^+$, respectively. Similarly, when using ωB97X-D4[19], Z-$\mathbf{P}_{\mathrm{a}}\mathrm{H}^+$ is predicted to be more stable (-2.4 kcal $\cdot$ mol^{-1}) when the electronic energy does not contain contributions from the dielectric environment. The opposite holds for the calculation with CPCM where $\Delta G = +1.5$ kcal $\cdot$ mol^{-1}, suggesting a slightly less favored Z-conformation than determined with PW6B95-D4/def2-QZVPP and CPCM(H_2O).

To ensure that the influence of the continuum solvation model is not an "artifact" due to a dissimilarity of the potential energy surfaces resulting from the composite approach—using a more sophisticated DFT-based theory level for single points on geometries calculated at a different theory level—we also checked for these energy differences using the PBEh-3c+CPCM(H_2O) theory level, which was used for the geometry optimizations (see also Fig. S1). Here, we again find *syn*-Z-$\mathbf{P}_{\mathrm{a}}\mathrm{H}^+$ to be disfavored by $+2.0$ kcal $\cdot$ mol^{-1} compared to *anti*-E-$\mathbf{P}_{\mathrm{a}}\mathrm{H}^+$. Given these findings, we conclude that, among other choices that follow eq. 2 to compute Gibbs energies, [ωB97X-D4/def2-QZVPP//PBEh-3c]+CPCM(H_2O) (^{2,d}) yields reasonable energetics to discriminate the relative stabilities of the studied systems. We adapt this choice for further discussions here and in the following.

The relative stability of the conjugate base forms Z-$\mathbf{P}_{\mathrm{a}}$, *anti*-E-$\mathbf{P}_{\mathrm{a}}$ is more clear for all employed theory levels. Here, *a*-*e* consistently predict the E-isomer to be more stable, e.g., $\Delta G' = (+39.5 - 43.9)$ kcal $\cdot$ mol^{-1} $= -4.4$ kcal $\cdot$ mol^{-1}(^{2,d}).

Another matter, which we inspected, is the role of the counter ion Cl^- on these relative stabilities. Using the automated interaction site screening (aISS) approach[20, 21] (GFN2-xTB+ALPB) with extended settings for the interaction site search and an otherwise analogous geometry refinement to what has been detailed above, we identifed *anti*-*E*-$\mathbf{P}_a$H·Cl and *syn*-*Z*-$\mathbf{P}_a$H·Cl geometries to further assess the isomer stabilities. With $+3.6\ \text{kcal}\cdot\text{mol}^{-1}$, the energy difference between E and Z essentially doubles compared to the $+1.5\ \text{kcal}\cdot\text{mol}^{-1}$, which further underlines that *anti*-*E*-$\mathbf{P}_a H^+$ should be the thermodynamically favored species in solution.

Considering the *syn* and *anti* conformations, we find that *anti*-*E*-$\mathbf{P}_a H^+$ is favored only by $\Delta G_{\text{conf}} = 0.6\ \text{kcal}\cdot\text{mol}^{-1}$ compared to the *syn* form. In *Z*-$\mathbf{P}_a H^+$, the *syn* configuration is favored more clearly (Tab. 1, $\Delta G_{\text{conf}} = (4.1 - 1.5)\ \text{kcal}\cdot\text{mol}^{-1} = 2.6\ \text{kcal}\cdot\text{mol}^{-1}$), which might be a result of stabilizing hydrogen bonding interactions between the NH group and carbonyl oxygen. Since these interactions are absent in the conjugate base forms $\mathbf{P}_a$, the conformational free energy differences are expected to be smaller. Here, we find that ΔG_{conf} between the *E*-forms is small with $1.1\ \text{kcal}\cdot\text{mol}^{-1}$. For *Z*-$\mathbf{P}_a$, the energy difference amounts to $2.0\ \text{kcal}\cdot\text{mol}^{-1}$.

S1.3 Simulation of UV-Vis Absorption Spectra

Time-dependent density functional theory (TD-DFT) was first used to compute UV-Vis absorption spectra and to analyze the excited states by means of their natural transition orbitals (NTOs).[22] Furthermore, we employ the density functional theory/multireference configuration interaction (DFT/MRCI)[23, 24] method to compare the computed vertical excitation energies ΔE_{0n} and oscillator strengths from the ground state to an excited singlet state n, f^{L}_{0n}. Throughout, all oscillator strength reported and the corresponding computed optical spectra use the length representation of the electric transition dipole moment.

To ease comparability between the results obtained with DFT/MRCI and TD-DFT, we employed the BHLYP[25] global hybrid functional (50% Fock exchange) and a def2-TZVP[12] basis set consistently. The choice of this exchange–correlation functional is linked to the fact that the employed R2018[26] DFT/MRCI Hamiltonian by Marian *et al.* is parametrized for this specific functional. We used an energy selection criterion of 0.8 E_h and the corresponding short parameter set (denoted as "short settings" in the following) for all DFT/MRCI calculations. For the post-SCF resolution-of-the-identity (RI) procedure (RI-C), the def2-TZVP/C[27] basis set was used.

For the computation of the reference configuration for both excited state methods, the RI-J[28, 29] approximation with the def2/J[30] auxiliary basis for Coulomb and the chain-of-spheres for exchange (COSX)[31, 32] integral approximation were employed. Implicit solvation effects were accounted for using the CPCM model for water. This further included use of non-equilibrium excited state solvation conditions via the linear-response CPCM (LR-CPCM)[33] in case of TD-DFT.

In Figure S2, the absorption spectra for $\mathbf{P}_a H^+$ are collected alongside the hole (h^+) and particle (e^-) NTOs with leading singular value (called "dominant NTOs" for brevity). Throughout this work, all stick spectra were broadened with Gaussians using a full width at half the maximum (FWHM) of 0.40 eV. Whenever the computed optical spectra were shifted in energy, this is given explicitly.

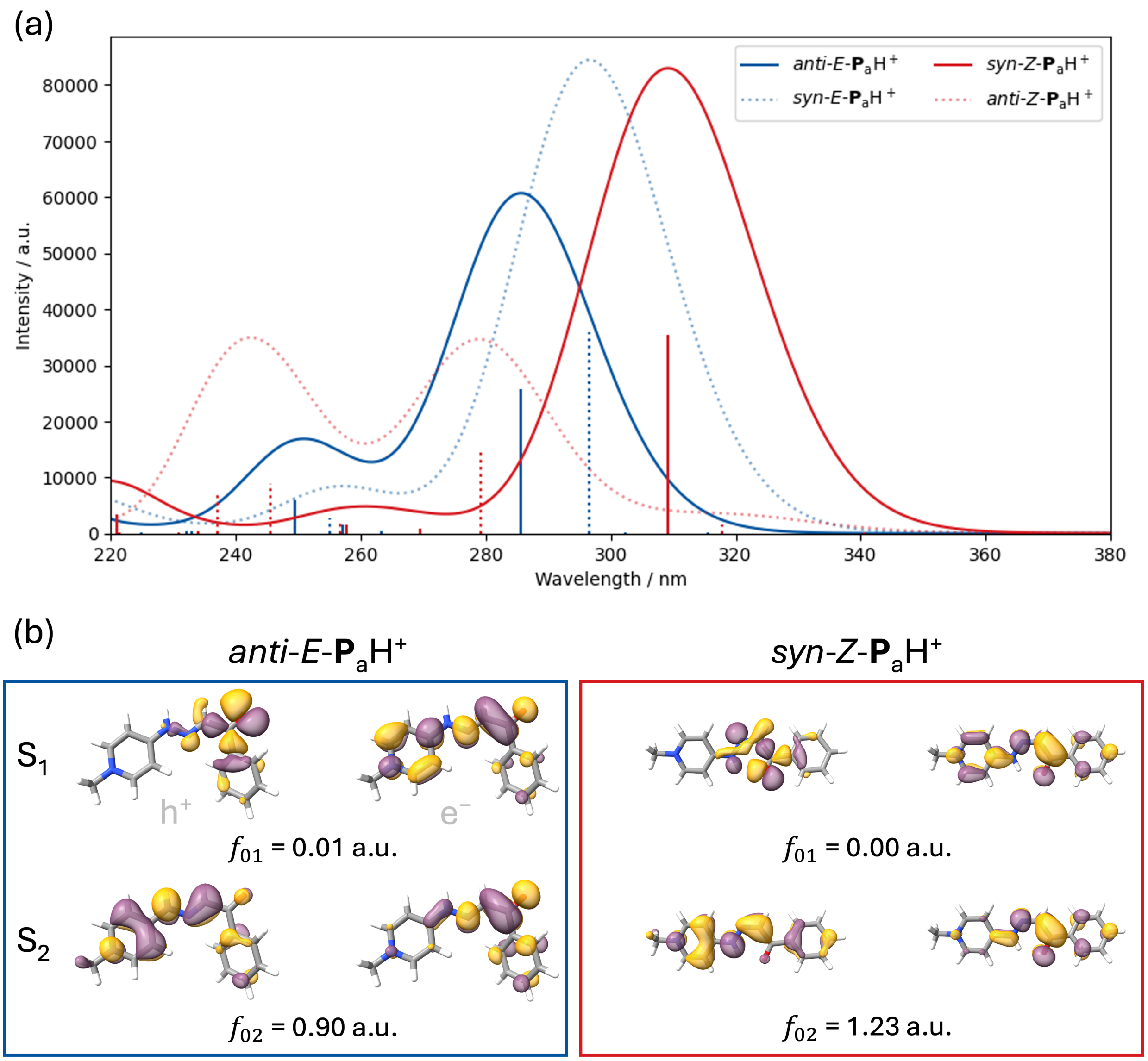

Figure S2: (a): Computed UV-Vis absorption spectra (TD-BHLYP/def2-TZVP+(LR-)CPCM) for *E*- (blue) and *Z*-$\mathbf{P}_a$H$^+$ (red). The species were further divided into (*syn* and *anti* (cf. Fig. S1), where the energetically favored form is always displayed with solid lines. (b): S_1 and S_2 dominant hole and particle NTOs of *anti*-*E*-$\mathbf{P}_a$H$^+$ and *syn*-*Z*-$\mathbf{P}_a$H$^+$ and corresponding oscillator strength f_{0n}. A contour value of ± 0.03 was used to display all isosurfaces.

Focusing first on the thermodynamically favored *syn*/*anti* form, namely *anti*-*E*-$\mathbf{P}_a$H$^+$and *syn*-*Z*-$\mathbf{P}_a$H$^+$, we observe that the *Z*-isomer is more red-shifted ($\lambda_{max} \approx 310$ nm) than the *E*-form ($\lambda_{max} \approx 286$ nm), which aligns with the experimental results. The first broadened band in the absorption spectrum (Fig. S2a) is due to the S_2 state showing a noticeable oscillator strength for both the *E*- and *Z*-isomer. Inspection of the S_1 and S_2 NTOs (Fig. S2b) shows that the former resembles an $n \rightarrow \pi^*$-type transition involving the n orbital of the carbonyl

group. This state is essentially dark ($f_{0n} \approx 0.00$ a.u.) in the optical spectra having an oscillator strength of only $f_{01} = 0.01$ a.u. (*anti*-*E*-$\mathbf{P}_a H^+$) or $f_{01} = 0.00$ a.u. (*syn*-*Z*-$\mathbf{P}_a H^+$). The S_2 state is of $\pi \to \pi^*$ character and features an oscillator strength $f_{02} = 0.90$ a.u. and $f_{02} = 1.23$ a.u. for *anti*-*E*-$\mathbf{P}_a H^+$and *syn*-*Z*-$\mathbf{P}_a H^+$, respectively.

Rotation of the benzylic substituent does not alter the ordering nor the character of the S_1 and S_2 state. However, a direct comparison of *syn*- and *anti*-*E*-$\mathbf{P}_a H^+$(Fig. S2a, blue lines) shows that the *syn*-form features a brighter S_2 state ($f_{02} = 1.25$ a.u.). This conformational change also increases the red-shift compared to the *anti*-form.

In Table 2, we compare the computed vertical excitation energies and oscillator strength of TD-DFT with those obtained using DFT/MRCI.

Table 2: Vertical excitation energies ΔE_{0n} and oscillator strength f_{0n} for S_1 and S_2 computed using TD-DFT and DFT/MRCI with a BHLYP/def2-TZVP+CPCM(H_2O) reference configuration. For DFT/MRCI calculations, the R2018 Hamiltonian and short settings were used. [a]: The excited state character was derived analysing the corresponding oscillator strength and NTOs or molecular orbitals.

		TD-DFT			DFT/MRCI		
		ΔE_{0n} (eV)	f_{0n} (a.u.)	State Character[a]	ΔE_{0n} (eV)	f_{0n} (a.u.)	State Character[a]
anti-*E*-$\mathbf{P}_a H^+$	S_1	4.10	0.01	$n \to \pi^*$	3.53	0.01	$n \to \pi^*$
	S_2	4.34	0.90	$\pi \to \pi^*$	3.95	0.83	$\pi \to \pi^*$
syn-*E*-$\mathbf{P}_a H^+$	S_1	3.93	0.01	$n \to \pi^*$	3.41	0.00	$n \to \pi^*$
	S_2	4.18	1.25	$\pi \to \pi^*$	3.79	1.17	$\pi \to \pi^*$
syn-*Z*-$\mathbf{P}_a H^+$	S_1	3.64	0.00	$n \to \pi^*$	3.23	0.00	$n \to \pi^*$
	S_2	4.01	1.23	$\pi \to \pi^*$	3.61	1.14	$\pi \to \pi^*$
anti-*Z*-$\mathbf{P}_a H^+$	S_1	3.90	0.05	$n \to \pi^*$	3.43	0.04	$n \to \pi^*$
	S_2	4.44	0.50	$\pi \to \pi^*$	4.00	0.43	$\pi \to \pi^*$
anti-*E*-$\mathbf{P}_a$	S_1	3.53	1.00	$\pi \to \pi^*$	3.09	0.24	$n \to \pi^*$
	S_2	3.67	0.02	$n \to \pi^*$	3.13	0.74	$\pi \to \pi^*$
syn-*E*-$\mathbf{P}_a$	S_1	3.34	1.20	$\pi \to \pi^*$	2.91	1.10	$\pi \to \pi^*$
	S_2	3.57	0.00	$n \to \pi^*$	3.06	0.01	$n \to \pi^*$
syn-*Z*-$\mathbf{P}_a$	S_1	2.85	0.01	$n \to \pi^*$	2.42	0.01	$n \to \pi^*$
	S_2	3.35	1.16	$\pi \to \pi^*$	2.93	1.06	$\pi \to \pi^*$
anti-*Z*-$\mathbf{P}_a$	S_1	3.47	0.29	$\pi \to \pi^*$	2.98	0.23	$\pi \to \pi^*$
	S_2	3.88	0.11	$n \to \pi^*$	3.38	0.11	$n \to \pi^*$

ΔE_{0n} as determined using DFT/MRCI are considerably more red-shifted ($\sim$ 0.5 eV) than the quantities obtained with TD-DFT. The oscillator strength for the S_1 and S_2 states are in good agreement for both methods. Compared to the experimental spectra measured in aqueous buffer solution, this qualitative shift is appreciable and already aligns reasonably well with the experimental spectrum (see Fig. 1c in the main article).

Next, we inspected how the situation changes for the conjugate base form. In Figure S3, the computed spectra for all $\mathbf{P}_a$ isomers are compared against one another.

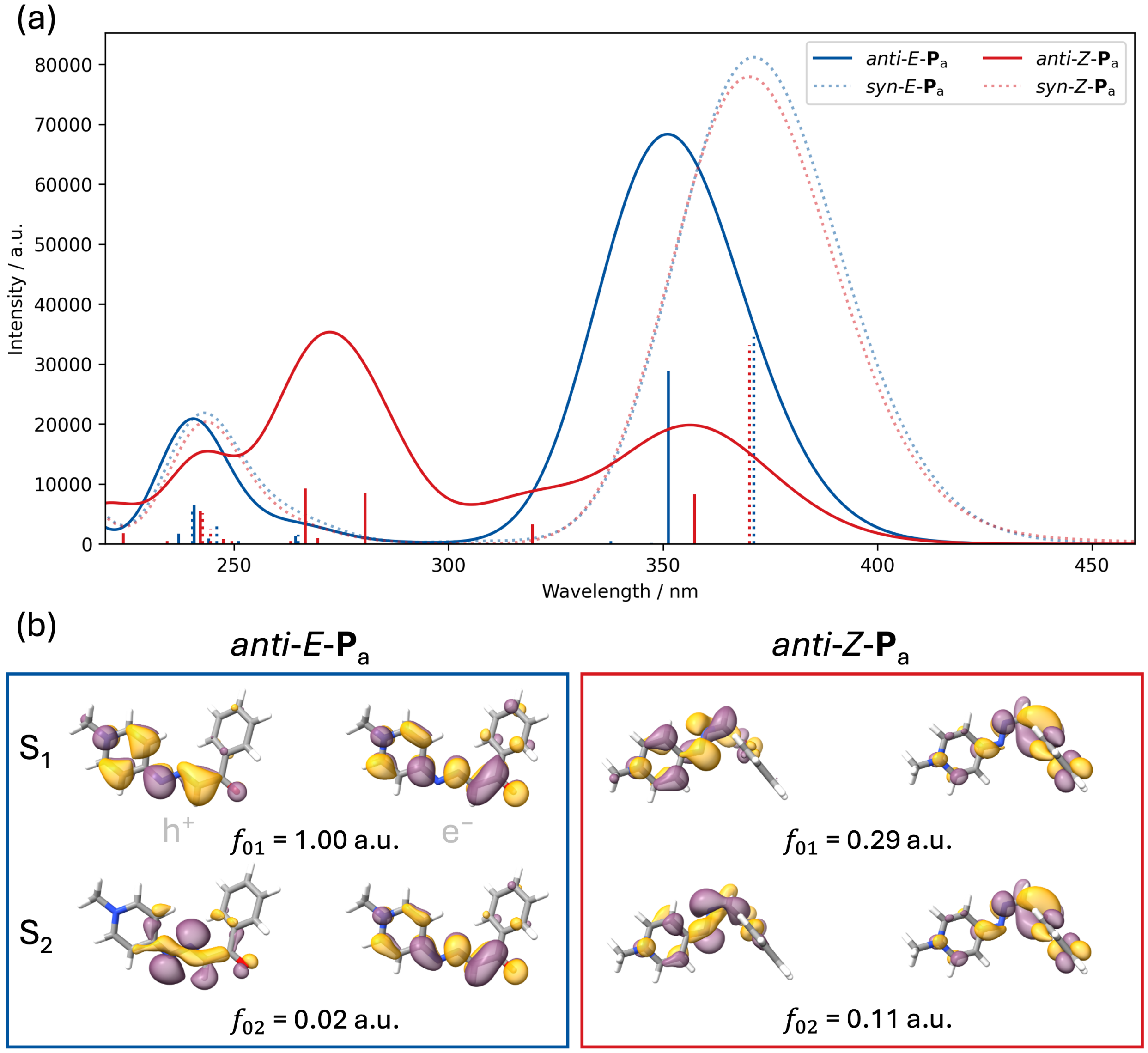

Figure S3: (a): Computed UV-Vis absorption spectra (TD-BHLYP/def2-TZVP+(LR-)CPCM) for *E*- (blue) and *Z*-$\mathbf{P}_a$ (red). The species were further divided into *syn*/*anti*-isomers, where the energetically favored one is illustrated with solid lines. (b): S_1 and S_2 dominant hole and particle NTOs for *anti*-*E*-$\mathbf{P}_a$ and *anti*-*Z*-$\mathbf{P}_a$ and corresponding oscillator strength f_{0n}.

We now find the lowest excited singlet state in the thermodynamically favored *anti*-*E*-$\mathbf{P}_a$ to be of $\pi \rightarrow \pi^*$-type showing a noticable oscillator strength of $f_{01} = 1.00$ a.u. The interchanged type of transition for S_1 ($\pi \rightarrow \pi^*$) and S_2 ($n \rightarrow \pi^*$) as compared to *anti*-*E*-$\mathbf{P}_a H^+$ (S_1 $n \rightarrow \pi^*$, S_2 $\pi \rightarrow \pi^*$) leads to a further red-shifted absorption band from 290 nm in *E*-$\mathbf{P}_a H^+$ to 350 nm in the deprotonated *anti*-*E*-$\mathbf{P}_a$. The state character of S_1 and S_2 between the *Z*-isomers of *syn*-$\mathbf{P}_a H^+$ and *anti*-$\mathbf{P}_a$ is altered. The latter shows notable oscillator strength of $f_{01} = 0.29$ a.u. and $f_{02} = 0.11$ a.u., respectively. These are, however, less bright compared to the $S_0 \rightarrow S_2$ excitation in *syn*-*Z*-$\mathbf{P}_a H^+$. The computed oscillator strength of *syn*-*Z*-$\mathbf{P}_a$ resemble the situation found for *syn*-*Z*-$\mathbf{P}_a H^+$ more closely. For both deprotonated species *syn*/*anti*-*Z*-$\mathbf{P}_a$, there exists, however, still a clear red-shift from 310 nm in *syn*-*Z*-$\mathbf{P}_a H^+$ to 357 nm in *anti*-*Z*-$\mathbf{P}_a$ or 370 nm in *syn*-*Z*-$\mathbf{P}_a$.

Comparing the results obtained with TD-DFT to the DFT/MRCI data (Tab. 2), we find a similar energetic shift of -0.5 eV than that seen for $\mathbf{P}_a H^+$. The oscillator strength for all $\mathbf{P}_a$-isomers except *anti*-*E*-$\mathbf{P}_a$ are again in close agreement. For *anti*-*E*-$\mathbf{P}_a$, DFT/MRCI yields two energetically proximate singlet excited states, S_1 and S_2, sharing roughly the combined oscillator strength of the S_1 state reported for TD-DFT.

S1.4 Modelling of Thermal Isomerization

Starting from the respective *syn*-*E*-isomers of $\mathbf{P}_a H^+$ and $\mathbf{P}_a$, GFN2-xTB+ALPB dihedral angle scans (30 structures, $\theta \in [176, 0]^\circ$) along the *E*/*Z*-isomerization coordinate θ were performed (Fig. S1). For these calculations, we used an open-shell GFN2-xTB electronic configuration with two unpaired electrons (keyword 'uhf 2' in xtb). Since the GFN2-xTB Hamiltonian does not contain spin-discriminating terms, S_1/T_1 are indistinguishable at the chosen level of theory. The interested reader is refered to Ref. 34 and 35 to read about spin-polarized versions of GFN methods.

This theory level effectively describes an S_1/T_1 excited state and yielded a "smooth" energy profile along the reaction coordinate. These geometries were reoptimized at the PBEh-3c+CPCM(H_2O) level using unrestricted Kohn-Sham (UKS) DFT and constraining θ. ORCA's functionality to converge to a broken-symmetry (BS) singlet solution was used in these calculations.

To evaluate whether thermal *E*/*Z* isomerization is feasible, we used these constraint geometries and the nuclear Hessians as guesses for BS-DFT transition state (TS) optimizations. To ensure that the identified TS connect the *E*- and *Z*-isomers, we further performed intrinsic reaction coordinate (IRC)[36] calculations. The identified TS are presented in Figure S4 with the computed barrier for isomerization and some geometrical features.

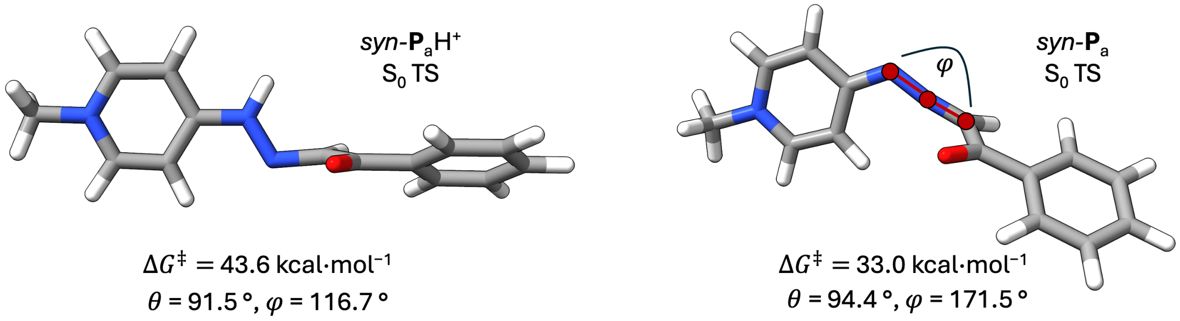

Figure S4: Transition states for the E/Z isomerization of *syn*-$\mathbf{P}_a$H$^+$ (left) and *syn*-$\mathbf{P}_a$ (right) computed at the PBEh-3c+CPCM(H_2O) level of theory. A N-N-C bend angle φ, torsion angle θ and the barrier from *syn*-Z-$\mathbf{P}_a$H$^+$ or *syn*-Z-$\mathbf{P}_a$to the corresponding TS, $\Delta G^‡$ is given as well (see section S1.2 and Tab. 1 (^{2,d}) for the theory level employed).

The barriers for the thermal back isomerization $Z \rightarrow E$ amount to 43.6 kcal · mol^{-1} for *syn*-$\mathbf{P}_a$H$^+$ and 33.0 kcal · mol^{-1} for *syn*-$\mathbf{P}_a$, respectively. Hence, our calculations suggest that thermal isomerization is essentially unfeasible for both species.

Comparing these barriers to the structurally similar photoswitch azobenzene, where the E/Z-isomerization coordinate is a C-N-N-C dihedral angle (N-N-C-C for $\mathbf{P}_a$H$^+$ and $\mathbf{P}_a$), a comparison can be made. Recently, Kaupp *et al.* inspected a rotational and inversion mechanisms for isomerization in azobenzene.[37] Evident from θ and φ in Fig. S4, the TS for Z-$\mathbf{P}_a$H$^+$ resembles a rotational-type mechanism, whereas the TS for Z-$\mathbf{P}_a$ corresponds to an inversion mechanism ($\varphi \approx 180°$) as also supported from only the former showing spin contamination and converging to a symmetry-broken solution.

Compared to what has been proposed in Ref. 37 for azobenzene, we suspect that DFT (PBEh-3c+CPCM(H_2O)) rather overestimates the barrier for thermal isomerization in the cases of $\mathbf{P}_a$H$^+$ and $\mathbf{P}_a$, since the experimental results render thermal $Z \rightarrow E$ reversion feasible. We will inspect this matter in the following further analyzing the potential energy curves along θ.

It should be made clear at this point already that the accuracy of the employed method (UKS-DFT) used to assess the barrier height for thermal isomerization is unfeasible. Potentially better agreement could be obtained resorting to multiconfigurational treatments[37] or even considering more sophisticated sampling strategies.[38] Since we are mainly interested in understanding the photoisomerization mechanism of $\mathbf{P}_a$ and $\mathbf{P}_a$H$^+$ in the present study, we will leave the task of deriving more accurate (lower) barriers for future work.

S1.5 Modelling of Photoisomerization

Potential Energy Curves

In Figure S5 below, we begin by comparing the potential energy curves for the isomerization *syn*-E-$\mathbf{P}_a$H$^+$$\rightarrow$*syn*-$Z$-$\mathbf{P}_aH^+$ using different methods.

We employed the hole-hole Tamm-Dancoff approximation (hh-TDA)[39] based on an N electron fractional occupation molecular orbital (FOMO) reference configuration.[40]

This level of theory was specifically chosen due to earlier work of Yu *et al.* on nonadiabatic molecular dynamics simulations dealing with the photoisomerization mechanism of azobenzene.[40] hh-TDA operates in a configuration space that only accounts for excitations into the lowest unoccupied molecular orbital (LUMO), however from all occupied molecular orbitals. This way, electron correlation effects, which are relevant for double bond isomerization[37, 40], and a reasonable description of minimum energy conical intersections (MECIs), are included.[41, 42] We used a development version of the TeraChem code[43, 44] (based on version 1.9.2) that features hh-TDA and an implementation of some 3c-composite methods.[45]

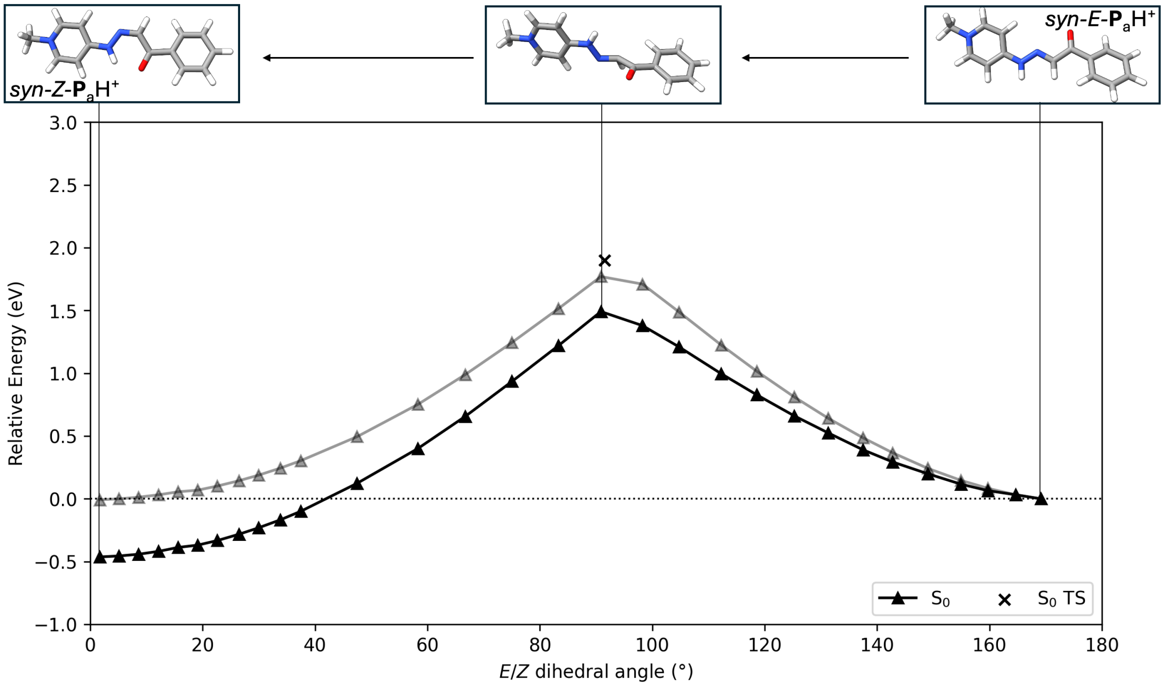

Figure S5: Potential energy curve of *syn*-E-$\mathbf{P}_a$H$^+$→ *syn*-Z-$\mathbf{P}_a$H$^+$ as obtained using hh-TDA at the constrained UKS-DFT geometries at the PBEh-3c+CPCM(H_2O) level of theory. The hh-TDA S_0 (black) and UKS S_0 (light gray, upper triangles) curves are shown for comparison. The identified S_0 TS (black cross, cf. Fig. S4) is displayed relative to the constrained UKS S_0 E-$\mathbf{P}_a$H$^+$ minimum.

UKS-DFT shows a comparable course of the S_0 potential energy curve compared to hh-TDA, but shifted to slightly higher energies. It should be noted that upon geometric relaxation of all degrees of freedom, *i.e.*, removing the constrain in θ, the anticipated destabilization of *syn*-Z-$\mathbf{P}_a$H$^+$ in comparison to *syn*-E-$\mathbf{P}_a$H$^+$ (Tab. 1) is obtained with UKS-DFT, but not so with hh-TDA.

Having offered a brief comparison to UKS-DFT, we decided, for the purpose of this work, to reside with hh-TDA for the description of states relevant for E/Z photoisomerization. This is due to its multistate nature letting us evaluate the role of several low-lying excited states more conveniently.

In the main article, we inspected the potential energy curves using the S_1/T_1 GFN2-xTB geometries obtained from a dihedral angle scan (Fig. 3).

There, we examined which of the S_0/S_1 or S_1/S_2 MECIs are relevant to transition from *syn*-*E*-$\mathbf{P}_a H^+$ to *syn*-*Z*-$\mathbf{P}_a H^+$ below.

An analogous investigation can be made for the deprotonated species $\mathbf{P}_a$ to hightlight differences to $\mathbf{P}_a H^+$(Fig. S6).

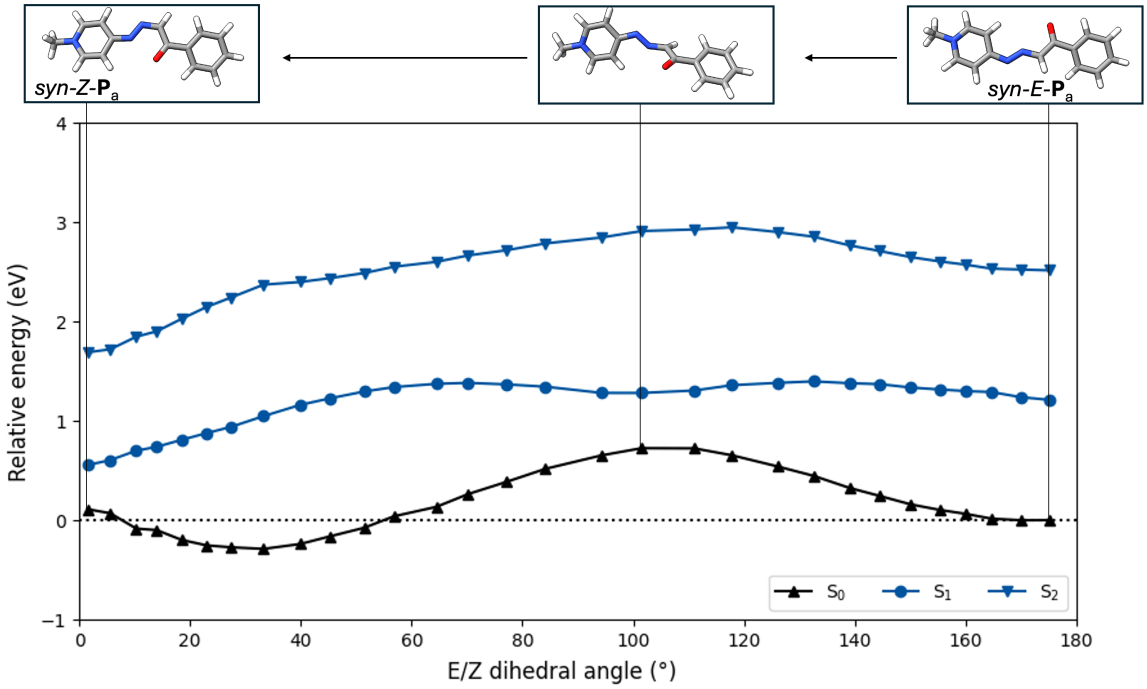

Figure S6: hh-TDA-PBEh-3c+CPCM//GFN2-xTB+ALPB potential energy curve for the $E \rightarrow Z$-isomerization of $\mathbf{P}_a$. The S_0 (black, upper triangle), S_1 (blue, circle), S_2 (blue, lower triangle) states are shown.

As pointed out in Fig. 3 in the main article and Tab. 2 above, the S_1 and S_2 excited states of *syn*-*E*-$\mathbf{P}_a$ are now of $\pi \rightarrow \pi^*$ and $n \rightarrow \pi^*$ character, respectively. The potential energy curves of the S_0 and $\pi \rightarrow \pi^*$ S_1 state approach one another around $\theta \approx 100°$, which again suggests that this isomerization could proceed via a S_0/S_1 MECI. It is interesting to see that hh-TDA-PBEh-3c separates the $n \rightarrow \pi^*$ quiet substantially in energy, while it appears close in energy with DFT/MRCI at the fully relaxed ground state minimum (Tab. S2).

Identification of Crossing Points

MECIs were computed at the hh-TDA-PBEh-3c level (without CPCM(H_2O) due to implementation restrictions) in TeraChem. An energetic barrier to the MECI ΔE_{MECI} is estimated from the electronic energy of a local excited state minimum $E_{\mathrm{S}n}$ and the energy of the MECI, E_{MECI} as $\Delta E_{\mathrm{MECI}} = E_{\mathrm{MECI}} - E_{\mathrm{S}n}$. Taking a S_0/S_1 MECI, where $\theta \geq 90°$ as example, $E_{\mathrm{S}n}$ refers to the energetically higher-lying state (here: S_1) minimum resembling either the *E*- or *Z*-isomer more closely. To be more specific, we list the selected S_0/S_1 and S_1/S_2 MECIs for

$\mathbf{P}_\mathrm{a}\mathrm{H}^+$ and $\mathbf{P}_\mathrm{a}$ as well as the geometries associated with $E_{\mathrm{S}n}$ and used to derive ΔE_MECI in Figure S7 below.

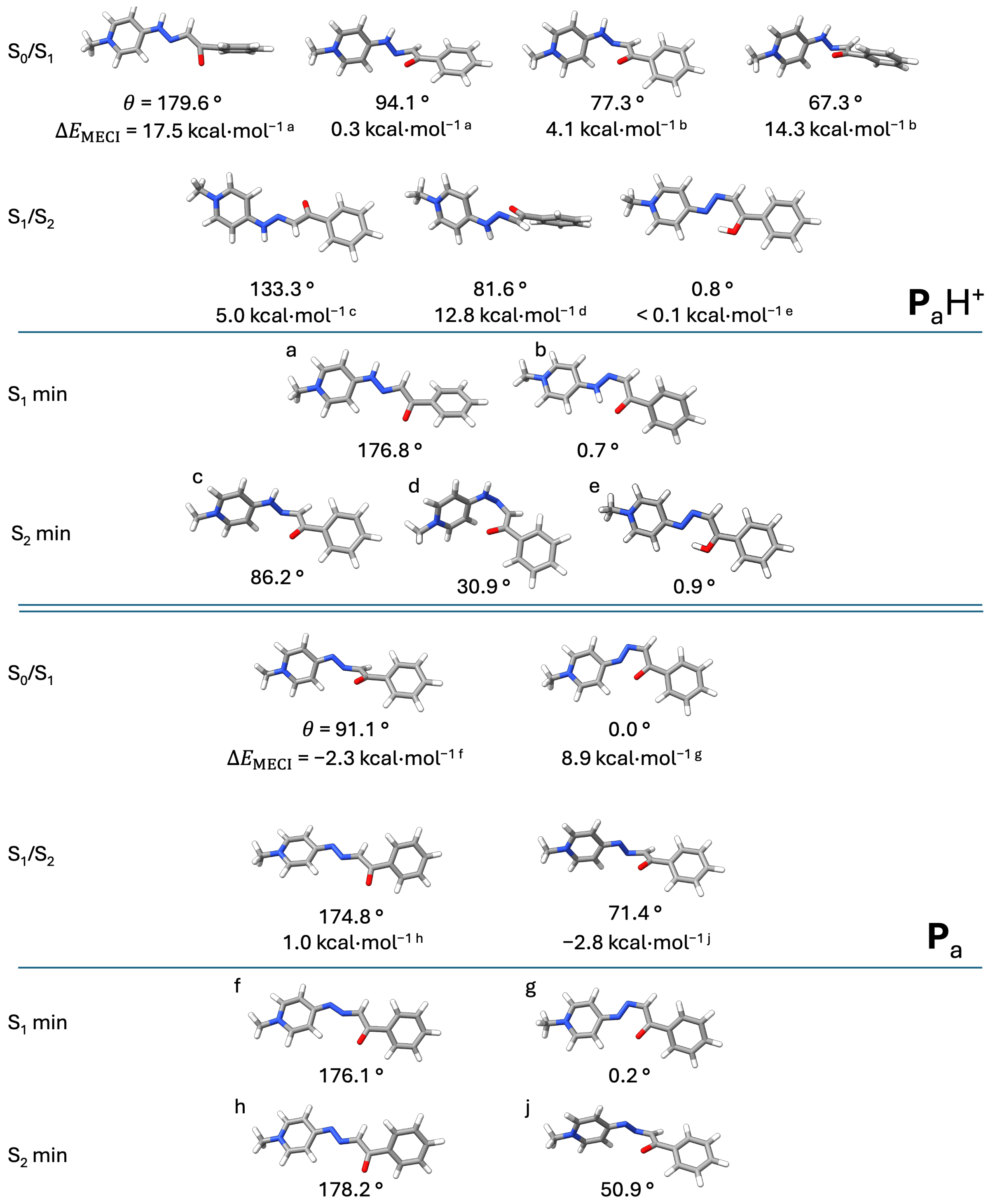

Figure S7: Selected hh-PBEh-3c S_0/S_1 (first row) and S_1/S_2 minimum energy conical intersections (second row) of $\mathbf{P}_a H^+$ obtained optimizing the geometries from the S_1/T_1 GFN2-xTB+ALPB potential energy curves along the E/Z dihedral angle θ (see above for further details). Dihedral angles θ and energy barriers ΔE_{MECI} as estimated from the proximate S_1 (third row) or S_2 excited state minima (fourth row) to the MECI are given as well. Divided by a double horizontal line, the same data points aquired for $\mathbf{P}_a$ are presented.

E-$\mathbf{P}_a H^+$ features a S_1/S_2 MECI with $\theta = 133.3°$ that is higher in energy by only 5.0 $\text{kcal} \cdot \text{mol}^{-1}$ with respect to the local energetic minimum (structure "c"). We expect that this barrier can be overcome within the lifetime of the excited state given that this state should be primarily populated at the chosen irradiation conditions ($\lambda = 254$ nm$\hat{=}$4.88 eV). This MECI allows to reach the less visible S_1 $n \rightarrow \pi^*$ state from which an S_0/S_1 MECI leads to the Z-isomer essentially barrierless (0.3 $\text{kcal} \cdot \text{mol}^{-1}$). There exist other S_0/S_1 MECIs, which we have identified, that could possibly facilitate a photochemical back isomerization. Likewise, however, Z-$\mathbf{P}_a H^+$ seems to be able to undergo intramolecular hydrogen transfer to the proximate carbonyl oxygen. This mechanism would hinder direct Z/E-backisomerization.

Inspection of the corresponding MECIs of $\mathbf{P}_a$ renders E/Z-isomerization feasible for this system as well. Here, the "barrier" to the S_0/S_1 MECI is negative with -2.3 $\text{kcal} \cdot \text{mol}^{-1}$, which suggests that the proximate local minimum describes another excited state. Similarly, the barrier to a S_1/S_2 MECI amounts to -2.8 $\text{kcal} \cdot \text{mol}^{-1}$, potentially for the same reason.

References

[1] Bannwarth, C.; Ehlert, S.; Grimme, S. GFN2-xTB – An Accurate and Broadly Parametrized Self-Consistent Tight-Binding Quantum Chemical Method with Multipole Electrostatics and Density-Dependent Dispersion Contributions. *J. Chem. Theory Comput.* **2019**, *15*, 1652–1671.

[2] Semiempirical Extended Tight-Binding Program Package (`xtb`). `https://github.com/grimme-lab/xtb`.

[3] Pracht, P.; Bohle, F.; Grimme, S. Automated exploration of the low-energy chemical space with fast quantum chemical methods. *Phys. Chem. Chem. Phys.* **2020**, *22*, 7169–7192.

[4] Conformer-Rotamer Ensemble Sampling Tool (CREST). `https://github.com/grimme-lab/crest`.

[5] Ehlert, S.; Stahn, M.; Spicher, S.; Grimme, S. Robust and Efficient Implicit Solvation Model for Fast Semiempirical Methods. *J. Chem. Theory Comput.* **2021**, *17*, 4250–4261.

[6] Grimme, S.; Brandenburg, J. G.; Bannwarth, C.; Hansen, A. Consistent structures and interactions by density functional theory with small atomic orbital basis sets. *J. Chem. Phys.* **2015**, *143*, 054107.

[7] Barone, V.; Cossi, M. Quantum Calculation of Molecular Energies and Energy Gradients in Solution by a Conductor Solvent Model. *J. Phys. Chem. A* **1998**, *102*, 1995–2001.

[8] Garcia-Ratés, M.; Neese, F. Effect of the Solute Cavity on the Solvation Energy and its Derivatives within the Framework of the Gaussian Charge Scheme. *J. Comput. Chem.* **2020**, *41*, 922–939.

[9] Neese, F. The ORCA program system. *WIREs Comput. Mol. Sci.* **2012**, *2*, 73–78.

[10] Neese, F.; Wennmohs, F.; Becker, U.; Riplinger, C. The ORCA quantum chemistry program package. *J. Chem. Phys.* **2020**, *152*, 224108.

[11] Neese, F. Software Update: The ORCA Program System—Version 6.0. *WIREs Comput. Mol. Sci.* **2025**, *15*, e70019.

[12] Weigend, F.; Ahlrichs, R. Balanced basis sets of split valence, triple zeta valence and quadruple zeta valence quality for H to Rn: Design and assessment of accuracy. *Phys. Chem. Chem. Phys.* **2005**, *7*, 3297–3305.

[13] Perdew, J. P.; Burke, K.; Ernzerhof, M. Generalized Gradient Approximation Made Simple. *Phys. Rev. Lett.* **1996**, *77*, 3865–3868, erratum *Phys. Rev. Lett.* **78**, 1396 (1997).

[14] Grimme, S.; Antony, J.; Ehrlich, S.; Krieg, H. A consistent and accurate ab initio parametrization of density functional dispersion correction (DFT-D) for the 94 elements H-Pu. *J. Chem. Phys.* **2010**, *132*, 154104.

[15] Kruse, H.; Grimme, S. A geometrical correction for the inter- and intra-molecular basis set superposition error in Hartree-Fock and density functional theory calculations for large systems. *J. Chem. Phys.* **2012**, *136*, 154101.

[16] Spicher, S.; Grimme, S. Efficient Computation of Free Energy Contributions for Association Reactions of Large Molecules. *J. Phys. Chem. Lett.* **2020**, *11*, 6606–6611.

[17] Zhao, Y.; Truhlar, D. G. Design of Density Functionals That Are Broadly Accurate for Thermochemistry, Thermochemical Kinetics, and Nonbonded Interactions. *J. Phys. Chem. A* **2005**, *109*, 5656–5667.

[18] Caldeweyher, E.; Ehlert, S.; Hansen, A.; Neugebauer, H.; Spicher, S.; Bannwarth, C.; Grimme, S. A generally applicable atomic-charge dependent London dispersion correction. *J. Chem. Phys.* **2019**, *150*, 154122.

[19] Najibi, A.; Goerigk, L. DFT-D4 counterparts of leading meta-generalized-gradient approximation and hybrid density functionals for energetics and geometries. *J. Comput. Chem.* **2020**, *41*, 2562–2572.

[20] Plett, C.; Grimme, S. Automated and Efficient Generation of General Molecular Aggregate Structures. *Angew. Chem. Int. Ed.* **2023**, *62*, e202214477.

[21] Grimme, S.; Bannwarth, C.; Caldeweyher, E.; Pisarek, J.; Hansen, A. A general intermolecular force field based on tight-binding quantum chemical calculations. *J. Chem. Phys.* **2017**, *147*, 161708.

[22] Martin, R. L. Natural transition orbitals. *J. Chem. Phys.* **2003**, *118*, 4775–4777.

[23] Grimme, S.; Waletzke, M. A combination of Kohn–Sham density functional theory and multi-reference configuration interaction methods. *J. Chem. Phys.* **1999**, *111*, 5645–5655.

[24] Marian, C. M.; Heil, A.; Kleinschmidt, M. The DFT/MRCI method. *WIREs Comput. Mol. Sci.* **2019**, *9*, e1394.

[25] Becke, A. D. A new mixing of Hartree–Fock and local density-functional theories. *J. Chem. Phys.* **1993**, *98*, 1372–1377.

[26] Heil, A.; Kleinschmidt, M.; Marian, C. M. On the performance of DFT/MRCI Hamiltonians for electronic excitations in transition metal complexes: The role of the damping function. *J. Chem. Phys.* **2018**, *149*, 164106.

[27] Hellweg, A.; Hättig, C.; Höfener, S.; Klopper, W. Optimized Accurate Auxiliary Basis Sets for RI-MP2 and RI-CC2 Calculations for the Atoms Rb to Rn. *Theor. Chem. Acc.* *117*, 587–597.

[28] Vahtras, O.; Almlöf, J.; Feyereisen, M. W. Integral approximations for LCAO-SCF calculations. *Chem. Phys. Lett.* **1993**, *213*, 514–518.

[29] Neese, F. An improvement of the resolution of the identity approximation for the formation of the Coulomb matrix. *J. Comput. Chem.* **2003**, *24*, 1740–1747.

[30] Weigend, F. Accurate Coulomb-fitting basis sets for H to Rn. *Phys. Chem. Chem. Phys.* **2006**, *8*, 1057.

[31] Neese, F.; Wennmohs, F.; Hansen, A.; Becker, U. Efficient, approximate and parallel Hartree–Fock and hybrid DFT calculations. A 'chain-of-spheres' algorithm for the Hartree–Fock exchange. *Chem. Phys.* **2009**, *356*, 98–109.

[32] Helmich-Paris, B.; de Souza, B.; Neese, F.; Izsák, R. An improved chain of spheres for exchange algorithm. *J. Chem. Phys.* **2021**, *155*, 104109.

[33] Cammi, R.; Mennucci, B.; Tomasi, J. Fast Evaluation of Geometries and Properties of Excited Molecules in Solution: A Tamm-Dancoff Model with Application to 4-Dimethylaminobenzonitrile. *J. Phys. Chem. A* **2000**, *104*, 5631–5637.

[34] Neugebauer, H.; Bädorf, B.; Ehlert, S.; Hansen, A.; Grimme, S. High-throughput screening of spin states for transition metal complexes with spin-polarized extended tight-binding methods. *J. Comput. Chem.* **2023**, *44*, 2120–2129.

[35] Moradi, S.; Tomann, R.; Hendrix, J.; Head-Gordon, M.; Stein, C. J. Spin parameter optimization for spin-polarized extended tight-binding methods. *J. Comput. Chem.* **2024**, *45*, 2786–2792.

[36] Ishida, K.; Morokuma, K.; Komornicki, A. The intrinsic reaction coordinate. An ab initio calculation for HNC→HCN and H^- + CH4→CH4 + H^-. *J. Chem. Phys.* **2008**, *66*, 2153–2156.

[37] Reimann, M.; Teichmann, E.; Hecht, S.; Kaupp, M. Solving the Azobenzene Entropy Puzzle: Direct Evidence for Multi-State Reactivity. *J. Phys. Chem. Lett.* **2022**, *13*, 10882–10888.

[38] Muždalo, A.; Saalfrank, P.; Vreede, J.; Santer, M. Cis-to-Trans Isomerization of Azobenzene Derivatives Studied with Transition Path Sampling and Quantum Mechanical/Molecular Mechanical Molecular Dynamics. *J. Chem. Theory Comput.* **2018**, *14*, 2042–2051.

[39] Bannwarth, C.; Yu, J. K.; Hohenstein, E. G.; Martínez, T. J. Hole–hole Tamm–Dancoff-approximated density functional theory: A highly efficient electronic structure method incorporating dynamic and static correlation. *J. Chem. Phys.* **2020**, *153*, 024110.

[40] Yu, J. K.; Bannwarth, C.; Hohenstein, E. G.; Martínez, T. J. Ab Initio Nonadiabatic Molecular Dynamics with Hole–Hole Tamm–Dancoff Approximated Density Functional Theory. *J. Chem. Theory Comput.* **2020**, *16*, 5499–5511.

[41] Teh, H.-H.; Subotnik, J. E. The Simplest Possible Approach for Simulating S0–S1 Conical Intersections with DFT/TDDFT: Adding One Doubly Excited Configuration. *J. Phys. Chem. Lett.* **2019**, *10*, 3426–3432.

[42] Levine, B. G.; Ko, C.; Quenneville, J.; MartÍnez, T. J. Conical intersections and double excitations in time-dependent density functional theory. *Mol. Phys.* **2006**, *104*, 1039–1051.

[43] Seritan, S.; Bannwarth, C.; Fales, B. S.; Hohenstein, E. G.; Kokkila-Schumacher, S. I. L.; Luehr, N.; Snyder, J. W.; Song, C.; Titov, A. V.; Ufimtsev, I. S.; Martínez, T. J. TeraChem: Accelerating electronic structure and ab initio molecular dynamics with graphical processing units. *J. Chem. Phys.* **2020**, *152*, 224110.

[44] Seritan, S.; Bannwarth, C.; Fales, B. S.; Hohenstein, E. G.; Isborn, C. M.; Kokkila-Schumacher, S. I. L.; Li, X.; Liu, F.; Luehr, N.; Snyder Jr., J. W.; Song, C.; Titov, A. V.; Ufimtsev, I. S.; Wang, L.-P.; Martínez, T. J. TeraChem: A graphical processing unit-accelerated electronic structure package for large-scale ab initio molecular dynamics. *WIREs Comput. Mol. Sci.* **2021**, *11*, e1494.

[45] Steinbach, P.; Bannwarth, C. Combining low-cost electronic structure theory and low-cost parallel computing architecture. *Phys. Chem. Chem. Phys.* **2024**, *26*, 16567–16578.
